# Selecting Chiral BINOL‐Derived Phosphoric Acid Catalysts: General Model To Identify Steric Features Essential for Enantioselectivity

**DOI:** 10.1002/chem.201702019

**Published:** 2017-09-14

**Authors:** Jolene P. Reid, Jonathan M. Goodman

**Affiliations:** ^1^ Centre for Molecular Informatics, Department of Chemistry University of Cambridge Lensfield Road Cambridge CB2 1EW United Kingdom

**Keywords:** asymmetric catalysis, binaphthol, catalyst choice, chirality, phosphoric acid

## Abstract

Choosing the optimal catalyst for a new transformation is challenging because the ideal molecular requirements of the catalyst for one reaction do not always simply translate to another. Large groups at the 3,3′ positions of the binaphthol rings are important for efficient stereoinduction but if they are too large this can lead to unusual or poor results. By applying a quantitative steric assessment of the substituents at the 3,3′ positions of the binaphthol ring, we have systematically studied the effect of modulating this group on enantioselectivity for a wide range of reactions involving imines, and verified this analysis using ONIOM calculations. We have shown that in most reactions, the stereochemical outcome depends on both proximal and remote sterics. Summarising detailed calculations into a simple qualitative model identifies and explains the steric features required for high selectivity. This model is consistent with seventy seven papers reporting reactions (over 1000 transformations in total), and provides a straightforward decision tree for selecting the best catalyst.

## Introduction

Chiral phosphoric acids have become popular in organic synthesis, and are recognised as broadly applicable Brønsted acid catalysts for many important transformations.^1^, ^2^, ^3^ The addition of nucleophiles to imines is a major class of reactions catalysed by chiral phosphoric acids.^4^ A number of nucleophiles participate efficiently including N‐heterocycles,^5^ transfer hydrogenation reagents,^6^, ^7^ enols,^8^ enamides,^9^ thiols,^10^ alcohols^11^ and amines,^12^ amongst others. Substituents at the 3,3′ position on the binaphthol ring are central to the selectivity, and different groups are optimal for different applications. Generally, large steric bulk is required for high enantioselectivity. However, if the substituents are too large this may stop reactions altogether,^13^ or, in some cases, reverse the sense of stereoinduction.^14^ Hence, the ability to select a catalyst rationally to achieve a desired selectivity is both necessary and difficult. Despite the numerous computational and experimental studies dedicated to this important area of catalysis, the selectivity trends are not well understood and this makes the strategic selection of catalyst challenging.^15^

**Figure 1:**
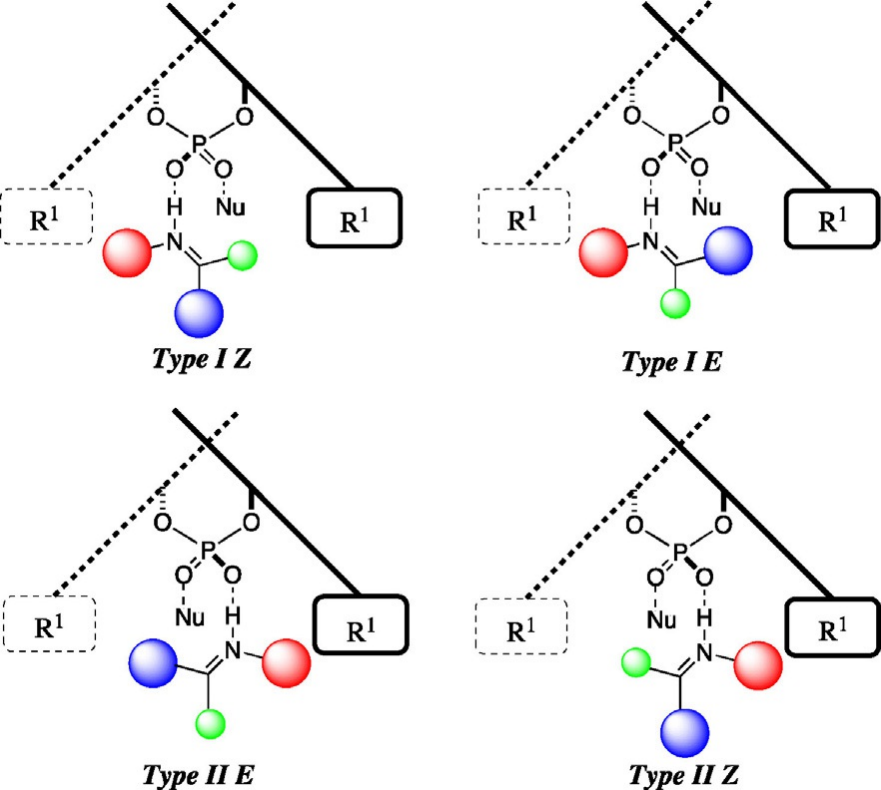
Transition state models for the prediction of stereoselectivity.

Computational investigations have shown that phosphoric acids often catalyse reactions through a bifunctional mechanism in which the catalyst simultaneously activates both the electrophile and the nucleophile, shown in Figure 1.^16^ The catalyst binds to the substrate via the catalyst hydroxyl group and there is a second interaction from the phosphoryl oxygen to the nucleophile's proton. The C_2_ symmetry of the phosphate anion allows us to place the imine at the front without loss in generality. The N‐substituent can be directed away from the front of the 3,3′ groups which we label Type I or towards which we label Type II. The imine can adopt an *E* or *Z* configuration, leading to a total of four possible pathways. The mechanistic choice between the four pathways is dependent on a number of factors,^4^ and the role of the 3,3′ groups remains elusive.

**Figure 2:**
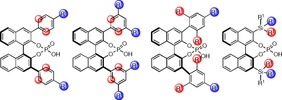
Commonly used phosphoric acid structures. The most popular scaffolds still retain the BINOL backbone with a bulky substituent at the 3,3**′**. Red indicates proximal and blue indicates remote sterics. For alkyl**‐**derived 3,3**′** groups with identical R^1^ substituents (see example on far right) the substituent that points up and towards is described as proximal sterics, down and back, remote.

The synthetic literature contains a large structural variety of catalysts and it has been found empirically that selectivity varies widely. Figure 2 shows the basic design of the most popular BINOL‐derived catalysts. Most have an aromatic ring at the 3,3′ positions, which can be further functionalised. Substitution at the 4‐ and the 3,5‐positions allows modulation of remote sterics, highlighted in blue. 2,4,6 Tri‐substitution allows placing large groups proximal and remote from the phosphoric acid. TRIP, developed by List,^17^ which has isopropyl groups at the 2,4,6 positions of the aromatic ring is the most versatile and selective catalyst known to date for the addition of nucleophiles to imines. Less frequently seen are alkyl and silyl derived 3,3′ substituents. The most successful catalyst of this class which places large triphenylsilane groups in the 3,3′ positions is commercially known as TIPSY, and was developed by MacMillan.^6^

**Figure 3:**
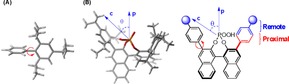
Parameters used for modelling chiral phosphoric acid structure. Steric parameter (A) is the energy required for rotation about a C−C bond, which measures proximal bulk. Steric parameter (B), AREA (θ), measures steric effects distant from the phosphoric acid. In both cases a 3D structure of TRIP is shown in a wire frame model as an example.

**Figure 4:**
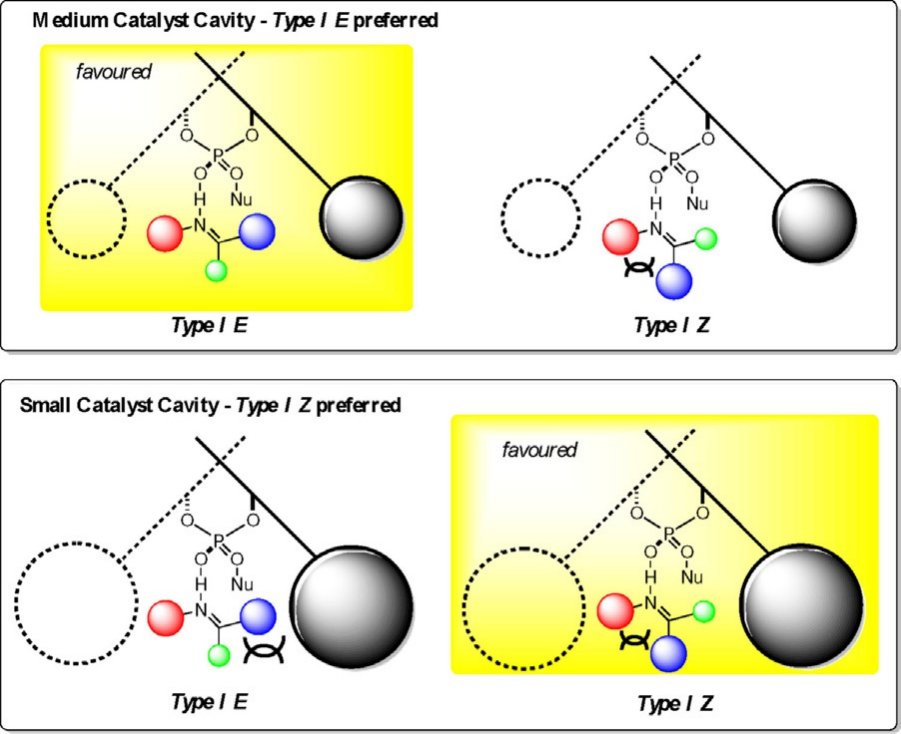
Qualitative model describing the preference for *Z* transition state with small AREA(θ) catalysts.

We recently reported a study on the influence of substituent structure at the 3,3**′** positions on the selectivity.^18^ Our strategy involved developing correlations between catalyst descriptors (Figure 3) and enantioselectivity to assess how altering the catalysts molecular features affect the transition state. The 3,3**′** group can be split into two steric regions; proximal (quantified by a rotation barrier) and distant (quantified by a remote environment angle, AREA(θ)) as described in Figure 2 and 3. In our theoretical study of the transfer hydrogenation of imines by Hantszch esters, we discovered that proximal sterics control the orientation (Type I or Type II) and the remote sterics control the configuration of the imine (*E* or *Z*). Large substituents proximal to the phosphoric acid reinforce Type I selectivity. However, moving to catalysts that have large steric demands remote from the phosphoric acid gradually reduces the energy difference between the Type I *E* and Type I *Z* pathways and ultimately leads to a preference for the *Z* pathway, a crossover point likely to occur for ketimines where the *Z* is energetically more feasible (Figure 4).

The model is consistent with a number of similar reactions in which the correct catalyst for a Type I *E* is not too large or too small but just right. However for a Type I *Z* reaction pathway small AREA(θ) catalysts are highly selective. Examples of reactions that follow this trend are given in Table 1.^6^, ^7^, ^10^, ^11^, 14b, 19, 20, 21, 22, 23

**Table 1:** Examples of the addition of symmetrical nucleophiles to imines. For each catalyst classification, we have chosen to compare the best performer from the catalyst screen of a model substrate under the same reaction conditions.

Reaction	Mechanism	AREA(θ) >70 [% *ee*]	medium AREA(θ) [% *ee*]	AREA(θ) <36 [% *ee*]
addition of diazophosphonates to *N*‐Boc imines^19^	Type I *E*	0	94	27
addition of diazoacetimides to N‐Boc imines^20^	Type I *E*	–	80	30
Strecker reaction with *N*‐Bn imines^21^	Type I *E*	–	93	0
addition of alcohols to *N*‐acyl imines^11^	Type I *E*	–	94	52
addition of thiols to *N*‐acyl imines^10^	Type I *E*	–	91	11
peroxidation of N‐acyl imines^22^	Type I *E*	–	84	3
reduction of alkynyl esters14b	Type I *E*/*Z*	−22	90	−85
reductive amination using benzothiazoline^23^	Type I *Z*	6	97	90
reductive amination using Hantzsch ester^6^	Type I *Z*	7	65	87
reduction of N‐Ar imines^7^	Type I *Z*	11	92	84

The large structural diversity coupled with unusual enantioselectivity trends complicate catalyst choice. Part of the reason for this disparity stems from the wide variation in reaction pathway choice (Type I or Type II, *E* or *Z*). Building on our recent contributions to understanding the effects of substituent at the 3,3′ positions on enantioselectivity, in this investigation we use a quantitative assessment of catalyst sterics, focusing on the bifunctional mode of activation of imines. We study theoretically a number of reactions in which we determine the factors that control the selectivity. We present our findings in a new qualitative model to understand and predict the steric features required for efficient stereoinduction. These studies provide a synthetic guide to strategic choice of catalyst based on substrate structure and reaction pathway.

## Results and Discussion

### Addition of symmetrical nucleophiles to imines

**Figure 5:**
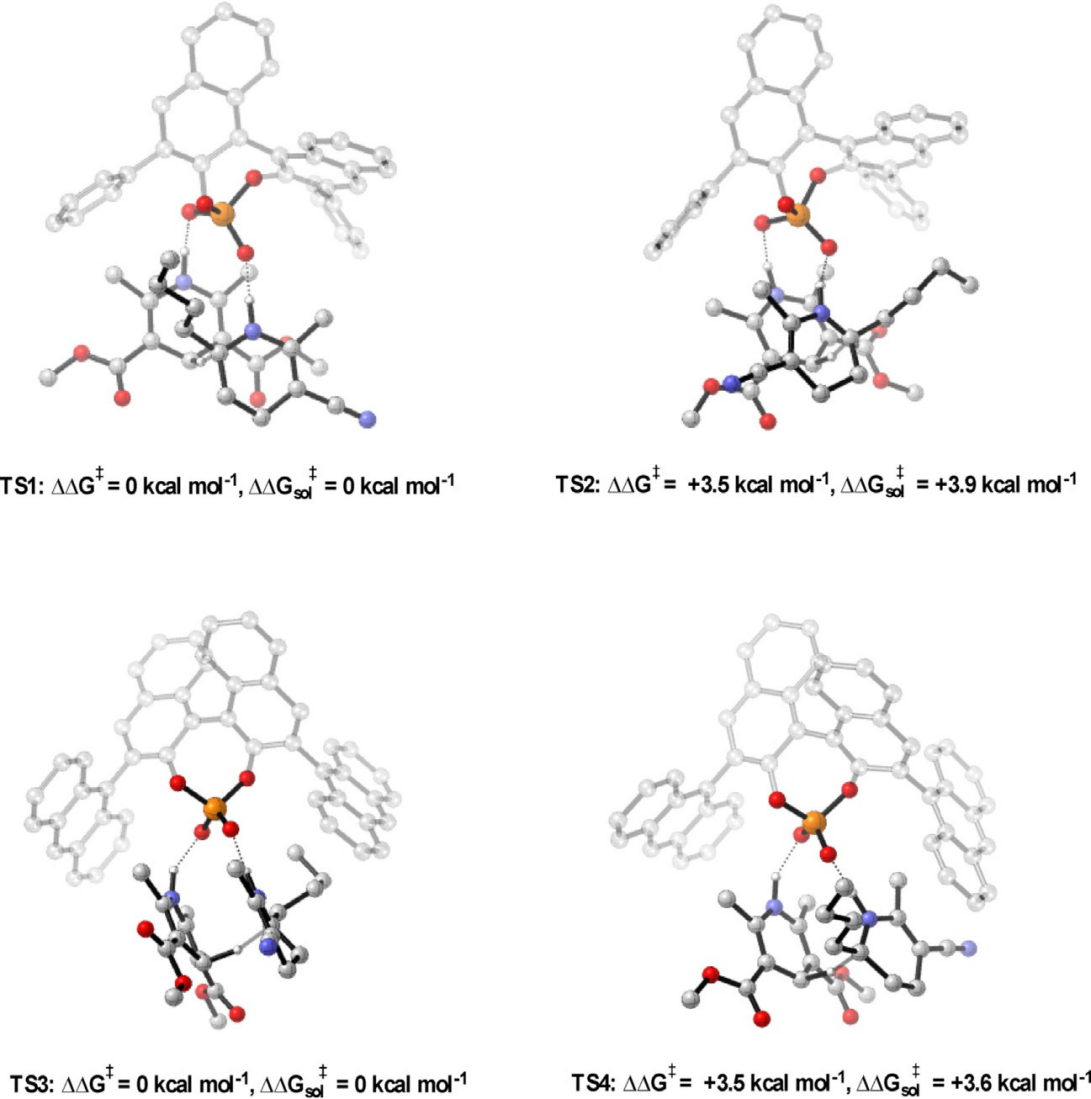
Competing TSs for the 9‐anthryl and phenyl derived phosphoric acid catalysed transfer hydrogenation reaction of cyclic imines. ONIOM (B3LYP/6‐31G**:UFF), single‐point energy M06‐2X/6‐31G**. Greyed‐out regions were treated with UFF, and the full‐colour regions were treated B3LYP/6‐31G**.

These trends from our transfer hydrogenation results led us to examine related reactions. Acyclic imines can equilibrate between the *E* and *Z* forms but cyclic imines are fixed in a *Z* configuration. The model (Figure 4) suggests that in reactions involving such substrates, only proximal sterics will affect the enantioselectivity to an appreciable extent. This is shown in the transfer hydrogenation reaction of pyridines catalysed by BINOL‐derived chiral phosphoric acids reported by Rueping et al. (Table 2).^24^ The examination of both rotation barrier and AREA(θ) as a function of enantioselectivity indicates that, enantioselectivity is almost independent of AREA(θ) but is proportional to rotation barrier. For instance, substituents that crowd access to the phosphoric acid such as 4‐PhC_6_H_4_, AREA(50), have smaller AREA(θ) values than less sterically demanding substituents such as phenyl, AREA(70), even though the rotational barrier is identical and the *ee* values are similar (−44 % vs. −46 %). It may appear, however, that the rotation barrier could overestimate the proximal steric effect of the 1‐naphthyl derived phosphoric acid. Alternatively, this failure of the steric description could represent the electronic nature of the catalyst as a significant influence on enantioselectivity. Assuming the reaction proceeds via a 1,4‐addition followed by tautomerisation to afford the imine, which is then further reduced, as proposed by Rueping et al. we computationally studied the enantio‐determining step. Detailed geometric and energetic information of the TS structures for the 9‐anthryl and phenyl derived phosphoric acids were obtained using ONIOM (B3LYP/6‐31G**:UFF) in line with Monte Carlo conformational searching, single‐point energies M06‐2X/6‐31G** were then calculated for the low energy reaction pathways (see Supporting Information for further details). In our calculations, we simplify both the cyclic substrate, the *n*‐pentyl group is replaced by a *n‐*butyl, such a small structural modification has been experimentally determined not to affect the enantioselectivity to an appreciable extent and the Hantszch ester, dimethyl is used instead of its diethyl counterpart. For the phenyl‐derived phosphoric acid catalysed reaction the lowest calculated transition state is **TS1** (Type II), which is in good agreement with the experimentally observed outcome. For the 9‐anthryl derived phosphoric acid catalysed reaction the lowest energy transition state is **TS3** (Type I), which accounts for the experimentally observed reversal in stereoinduction. The results are summarized in Figure 5, below. Although the calculated energy values are overestimated than compared to experiment (computed *ee* −99 %), the reproduction of qualitative trends is accurately predicted. The ONIOM (M06‐2X/6‐31G**:UFF) method indicates that the discrepancy between calculation and experiment traces back to the B3LYP component of the optimization in line with our findings from a previous transfer hydrogenation study.^18^ ONIOM (M06‐2X/6‐31G**:UFF) gave a smaller preference for the Type II pathway than ONIOM (B3LYP/6‐31G**:UFF). The energy difference between **TS1** and **TS2** re‐evaluated using ONIOM (M06‐2X/6‐31G**:UFF) was calculated to be 0.7 kcal mol^−1^ (computed *ee* −48 %). The Supporting Information provides a comparison of energy differences between the TSs in Figure 5 computed using both ONIOM methods. Both Type I and Type II reaction pathways followed different arrangements on comparing the catalysts. On analysing the TS pathways with varying 3,3′ substituent the most important difference is the absolute location of the reagents with respect to the 3,3′ substituents. In **TS1** (Type II) small proximal bulk creates a large cone of empty space at the front right hand side of the catalyst which can accommodate the cyclic imine, the butyl substituent is pointed away from the front of the 3,3′ groups and the Hantszch ester experiences little steric hindrance from the catalyst. A similar lack of steric interactions between imine and catalyst is present in **TS2** (Type I) but due to the increased steric interactions between the phenyl catalyst substituent and the Hantszch ester leads to a preference for **TS1**. Increasing the proximal catalyst sterics increases the steric interactions between the cyclic imine and the 3,3′ group forcing the imine to adopt a tilted disposition, **TS4**. The increased steric interactions destabilises the Type II pathway relative to Type I, which allows placing most of the substrate steric bulk furthest away from the catalyst. In line with the model presented in Figure 4, proximal sterics reinforce Type I selectivity due to unfavourable interactions with the N‐substituent and the 3,3′. Our computations predict that remote sterics exert little stereocontrolling effect; this correlation is consistent with the reduction of all known cyclic imines involving Hantzsch esters, in which proximal sterics dictate the level of stereoinduction. An optimal catalyst for such a reaction has large proximal sterics, some examples where this general trend is observed are given in Table 3.^25^, ^26^, ^27^, ^28^ The catalyst entries 6 and 7 (Table 2) present similar proximal steric environments but yet the enantioselectivities are very different. We explored a potential source of this systematic error. On calculation of the TS′s using these additional two catalysts we found that ONIOM (M06‐2X/6‐31G**:UFF) leads to consistent agreement between experiment and calculation. Inspection of the TSs suggest that increased non‐covalent interactions between the 1‐naphthyl derived phosphoric acid and the reagents are involved in lowering the energy of the Type II TS pathway (see Supporting Information). In most cases, the enantioselectivity is primarily steric in origin and the steric model works exceptionally well.

**Table 2:** Catalyst screening results for Rueping's transfer hydrogenation reaction. Type I and Type II are explained in Figure 1. Only BINOL‐derived catalysts are included to simplify analysis.

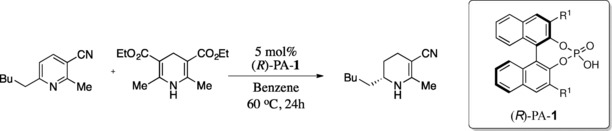					
Entry	Catalyst R_1_	AREA(θ)	Rotation barrier [kcal mol^−1^]	*ee* [%]	Mechanism
1	4‐PhC_6_H_4_	50	2.01	−44	Type II
2	3,5‐(CF_3_)_2_C_6_H_4_	62	2.02	−36	Type II
**3**	**Ph**	**70**	**2.05**	−**46**	**Type II**
4	2‐naphth	49	2.13	−32	Type II
5	3,5‐(*t*Bu)_2_‐4‐OMeC_6_H_3_	35	2.51	−56	Type II
6	1‐naphth	62	13.43	35	Type I
7	9‐phenanthryl	48	14.25	75	Type I
**8**	**9‐anthryl**	**61**	**26.53**	**89**	**Type I**

**Table 3:** Examples of cyclic imines reduced by Hantzsch ester catalyzed by chiral phosphoric acids. All energies in kcal mol^−1^. For each catalyst classification, we have chosen to compare the best performer from the catalyst screen of a model substrate under the same reaction conditions.

Substrate	Rotation barrier <3 [% *ee*]	Medium rotation barrier [% *ee*]	Rotation barrier >26 [% *ee*]
benzoxazine^25^	36	81	94
indoles^26^	42	72	97
quinoxalines^27^	10	64	90
3‐(trifluoromethyl)quinolones^28^	−35	72	97

### Addition of displaced nucleophiles to imines

**Figure 6:**
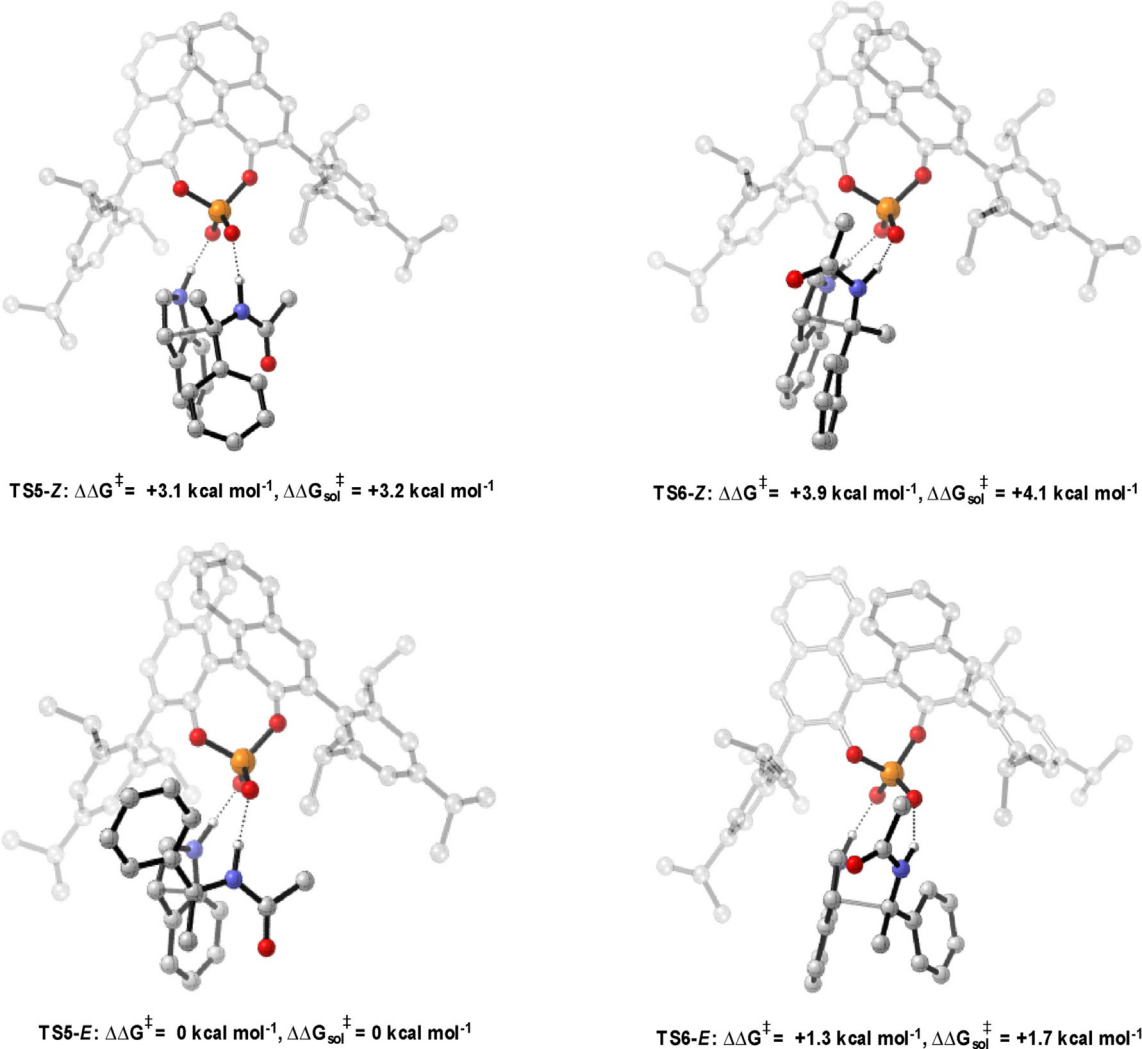
Competing TSs for the TRIP catalysed Friedel–Crafts. ONIOM (B3LYP/6‐31G**:UFF), single‐point energy M06‐2X/6‐31G**. Greyed‐out regions were treated with UFF, and the full‐colour regions were treated B3LYP/6‐31G**.

**Figure 7:**
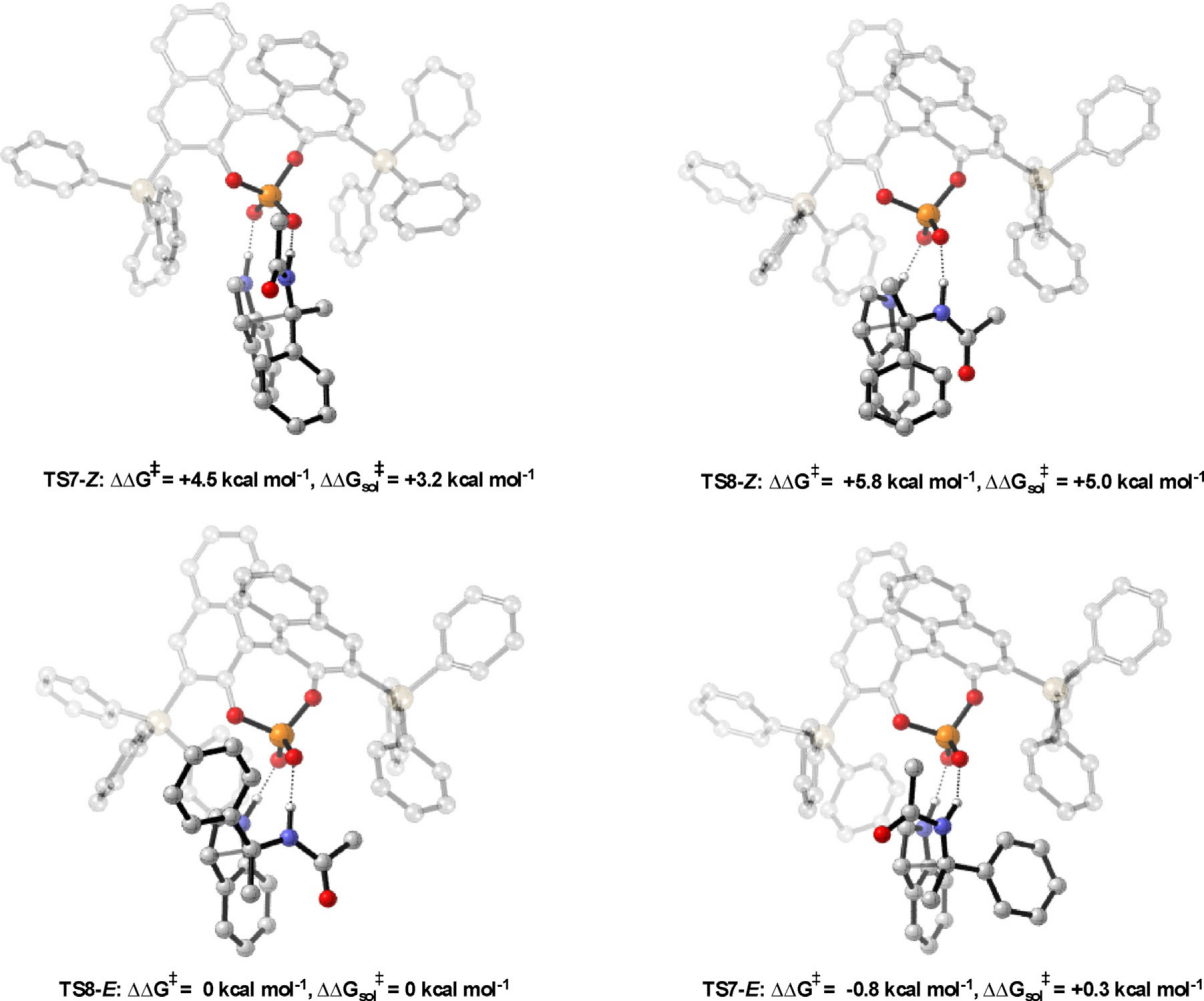
Competing TSs for the SiPh_3_ derived phosphoric acid catalysed Friedel–Crafts. ONIOM (B3LYP/6‐31G**:UFF), single‐point energy M06‐2X/6‐31G**. Greyed‐out regions were treated with UFF, and the full‐colour regions were treated B3LYP/6‐31G**.

**Figure 8:**
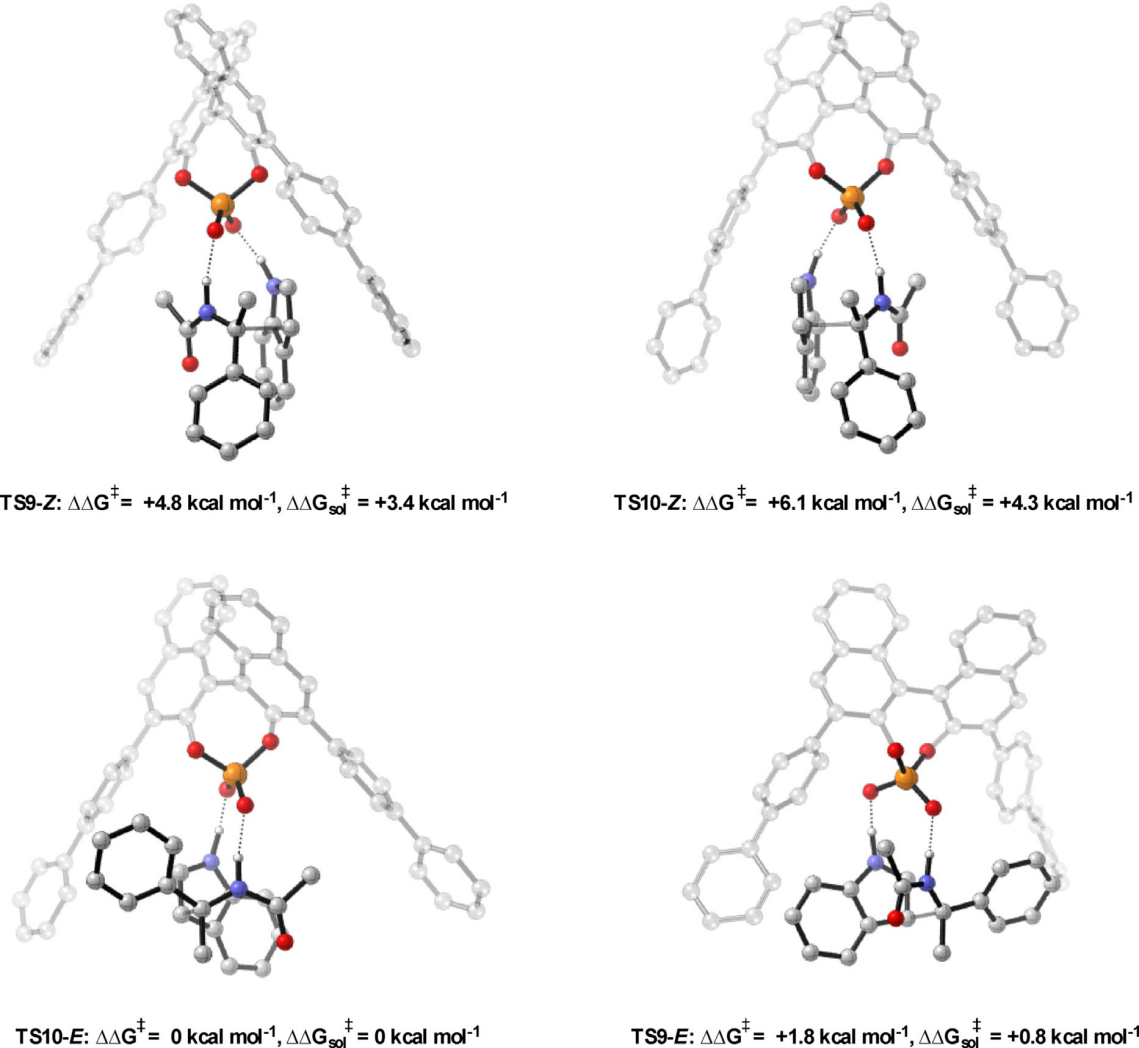
Competing TSs for the 4‐PhC_6_H_4_ derived phosphoric acid catalysed Friedel–Crafts. ONIOM (B3LYP/6‐31G**:UFF), single‐point energy M06‐2X/6‐31G**. Greyed‐out regions were treated with UFF, and the full‐colour regions were treated B3LYP/6‐31G**.

The addition of displaced nucleophiles (the nucleophilic carbon is not in line with the H‐bond that binds to the catalyst) to imines also show a similar “Goldilocks” effect, in which the correct catalyst is one that is not too small or too large but somewhere in between (Table 4).^29^ Experimentally, it was found that large proximal sterics were required for high levels of enantioselectivity. To understand the reasons for poor selectivity at both ends of the steric spectrum, and the requirement for large proximal sterics, the mechanism was computationally investigated. Transition states for the full catalyst system were located using the general method described above, the results are summarised in Figure 6, Figure 7 and Figure 8. For all catalysts the lowest energy TS pathway was Type II *E*, which is in good agreement with experiment. The *Z* pathways were higher in energy due to the internal steric substrate steric interactions. Changing the 3,3′ substituents from 2,4,6‐triisopropylphenyl (medium AREA(θ)) to SiPh_3_ (small AREA(θ)) leads to a 1.4 kcal mol^−1^ decrease in energy between the Type II *E* and the Type I *E* TS pathways, which translates experimentally to low levels of enantioselectivity. Visual inspection of the Type II *E* TSs shows that the indole pushes towards the imine forcing the benzene into free space, leaving empty space at the back right hand side of the catalyst. The imine N‐substitutent is placed in this empty catalyst pocket, with this in combination with a tilted disposition allows the large phenyl group to be placed furthest away from the catalyst bulk. Increasing the sterics remote from the phosphoric acid increases the steric interactions between the N‐substituent and one of the 3,3′ groups. This leads to a large energetic penalty and increases the energy of the Type II TS′s relative to that of the Type I. Proximal sterics were determined experimentally to be necessary for efficient stereoinduction, but it was not altogether clear how this catalyst feature imparted such strong enantioselectivity. Calculations on a 4‐biphenyl derived phosphoric acid showed that the catalyst created empty space at the front and therefore reduces the effect of the phenyl group leading to a reduction in the energetic preference for the Type II TS′s. This serves to explain the discrepancies in selectivity between catalysts with similar distal bulk, which we quantify using AREA(θ), but varying proximal bulk. This physical factor is not explicitly accounted for by the ligand AREA(θ) but can be described by a proximal bulk steric parameter, such as the rotation barrier.

**Table 4:** Effect of 3,3′ groups on the enantioselectivity of the Friedel–Crafts reaction.

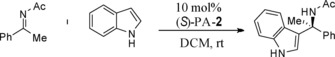				
Entry	Catalyst R_1_	AREA (θ)	Rotation barrier [kcal mol^−1^]	*ee* [%]
1	H	107	0.00	4
2	Ph	70	2.05	32
3	3,5‐(CF_3_)_2_C_6_H_4_	62	2.02	38
4	9‐anthryl	61	28.31	66
**5**	**2,4,6‐(*i*Pr)_3_C_6_H_3_**	**51**	**28.40**	**91**
**6**	**4‐PhC_6_H_4_**	**50**	**2.01**	**35**
7	2‐naphthyl	49	2.13	10
8	9‐phenanthryl	48	14.45	22
9	3,5‐(*t*Bu)_2_C_6_H_4_	41	2.65	72
**10**	**SiPh_3_**	**29**	**1.35**	**25**

**Figure 9:**
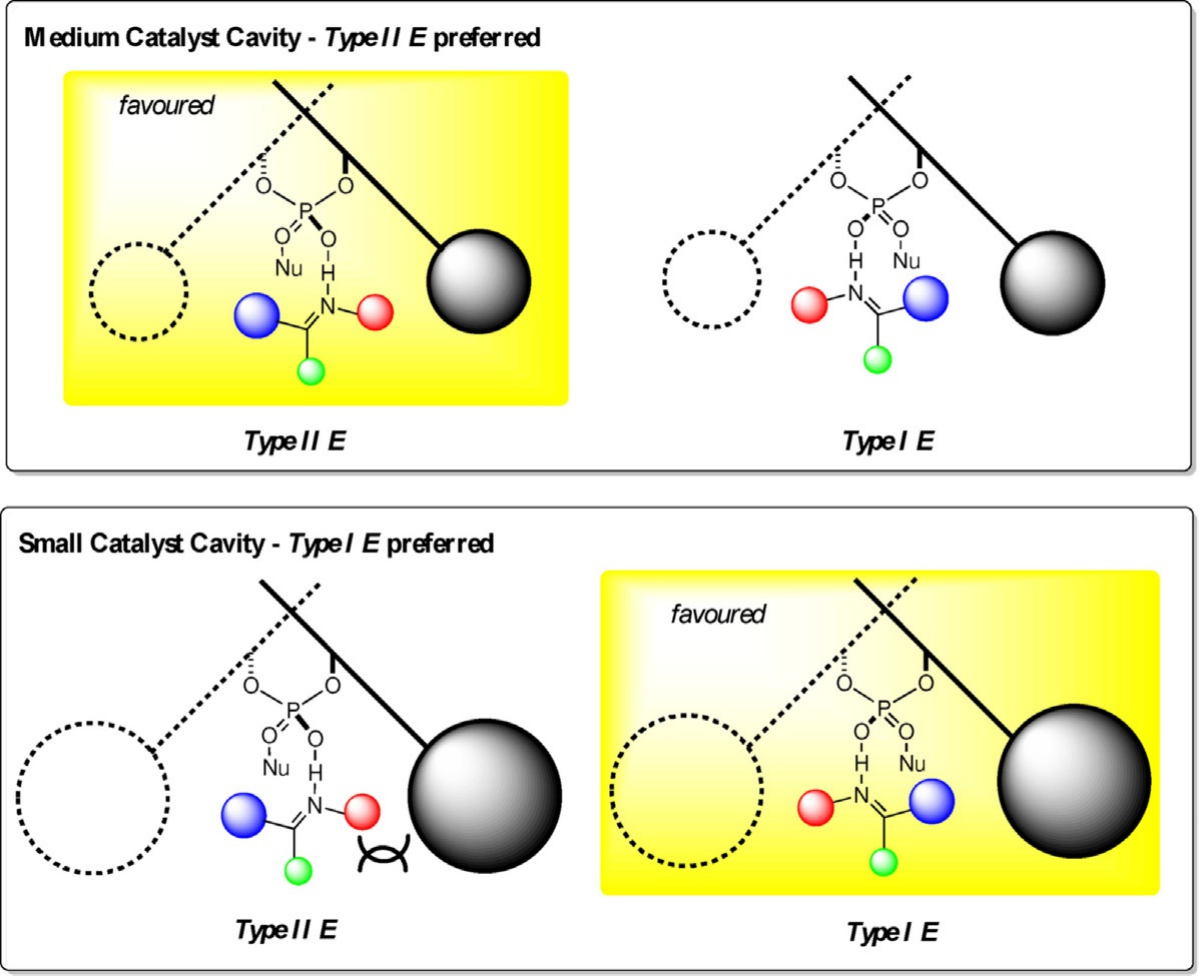
Qualitative model describing the preference for Type I *E* transition state with small AREA(θ) catalysts. In the Type II conformation, the imine adopts a tilted disposition to minimize steric interactions with the 3,3′ groups. Increasing sterics remote from the phosphoric acid increases the interaction between the N‐substituent and the 3,3′ group.

We have summarised the calculations into a simple accessible qualitative model which explains enantioselectivity trends with varying 3,3′ substituents (Figure 9). This model implies that reactions involving displaced nucleophiles proceeding via Type II *E* pathways will not be suitable for a reaction catalysed by small ligand AREA(θ) catalysts. Such catalysts bias towards a Type I transition states leading to little stereoinduction. An optimal catalyst for such a reaction would be one that is neither too big (raises Type I relative to Type II), nor too small (cannot differentiate between Type I or Type II), but somewhere in between. Although small AREA(θ) catalysts will not be selective for Type II reactions, they are expected to proceed with high levels of selectivity for Type I reaction pathways. Examples where this general trend is observed are given in Table 5 in support of the mechanistic model.^30^, ^31^, ^32^, ^33^, ^34^, ^35^

**Table 5:** Examples of the addition of displaced nucleophiles to imines. For each catalyst classification, we have chosen to compare the best performer from the catalyst screen of a model substrate under the same reaction conditions.

Reaction	Mechanism	AREA(θ) >70 [% *ee*]	Medium AREA(θ) [% *ee*]	AREA(θ) <29 [% *ee*]
addition of indole to *N*‐Ts imines^30^	Type I *E*	0	93	73
addition of dihydroindole to *N*‐Ts imines^31^	Type I *E*	–	97	99
Bignelli^32^	Type I/II *E*	–	80	−96
addition of indole to *N*‐Boc imines^33^	Type II *E*	–	92	2
addition of enamides to *N*‐acyl imines^34^	Type II *E*	4	96	9
Povarov^35^	Type II *E*	–	92	27

**Figure 10:**
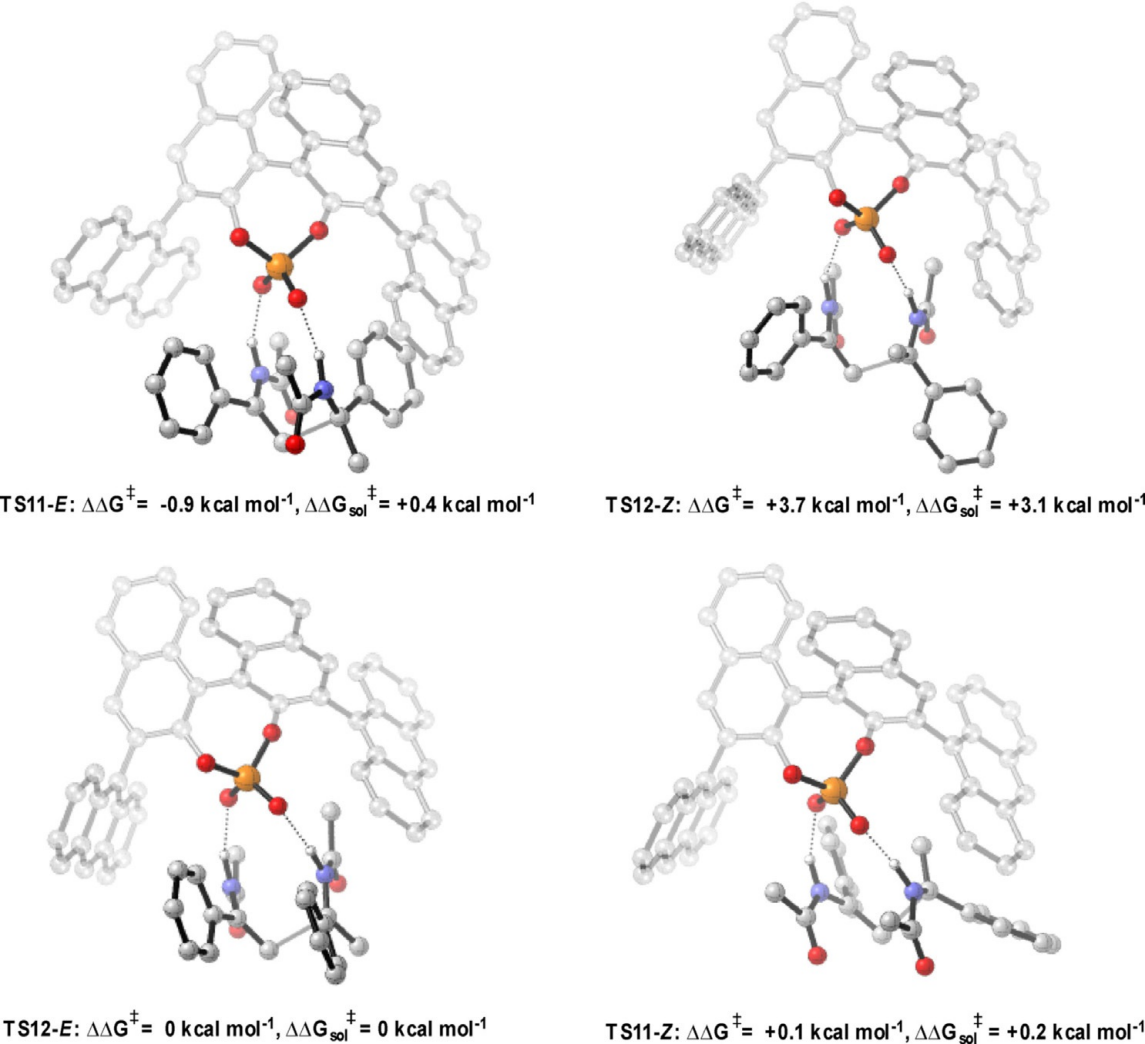
Competing TSs for the 9‐anthryl derived phosphoric acid catalysed addition of enamides. ONIOM (B3LYP/6‐31G**:UFF), single‐point energy M06‐2X/6‐31G**. Greyed‐out regions were treated with UFF, and the full‐colour regions were treated B3LYP/6‐31G**.

Whether the reaction between a displaced nucleophile and an imine proceeding via a Type II pathway benefits from large or small proximal sterics depends on the nucleophile. In the following reaction reported by Tsogoeva et al. both proximal and remote sterics played important sterochemical roles (Table 6).^34^ Increasing the remote sterics increases the enantioselectivity until a point it then turns and changes to a decline. Additionally, it was experimentally observed that large proximal sterics was detrimental to the reaction (entry 4). Calculations were performed on three catalysts; 4‐NO_2_C_6_H_4_, 9‐anthryl and SiPh_3_ derived phosphoric acids to assess the roles of the proximal and remote sterics. The reaction proceeds via a Type II  *E* pathway and as predicted by the model (Figure 9), increasing the remote sterics by changing the 3,3′ from 4‐NO_2_C_6_H_4_, AREA(64), to SiPh_3_, AREA(29), increases the energy of the Type II pathway relative to the Type I (see Supporting Information). This leads to small energy differences between the diastereomeric TSs and low levels of enantioselectivity, observing the same trends as with the Friedel–Crafts study. Despite the differing electronic nature between the catalysts there was no obvious electronic factors attenuating the selectivity. For the 9‐anthryl derived phosphoric acid the lowest energy TS was **TS12‐E** (Type II *E*), in this TS the phenyl substituent on the enamide prefers to be located in the empty catalyst pocket at the front left, this forces the imine to the right hand side of the catalyst. The phenyl substituent of the imine now experiences significant steric interactions with the 3,3′ group leading to a large energetic penalty and raising of the Type II TS′s relative to the Type I. The small energy difference between the Type II *E* and the Type I *E* (0.4 kcal mol^−1^) agrees with the poor levels of enantioselectivity observed experimentally (Figure 10). The calculations suggest that the reason for the enantioselectivity trends with more sterically demanding displaced nucleophiles is a consequence of the size of the substituent attached to the same carbon as the heteroatom that binds to the phosphoryl oxygen of the catalyst. If this substituent is large, the correct catalyst has small proximal sterics; too large raises the energy of Type II TS′s relative to Type I. Although, large proximal sterically derived catalysts are predicted not to be selective for reactions involving large displaced nucleophiles, they should proceed with high levels of enantioselectivity with small displaced nucleophiles. Examples of reactions exhibiting this trend are summarized in the Table 4.^32^, ^36^, ^37^, ^38^, ^39^

**Table 6:** Effect of 3,3′ groups on the enantioselectivity of the addition of enamides to imines.

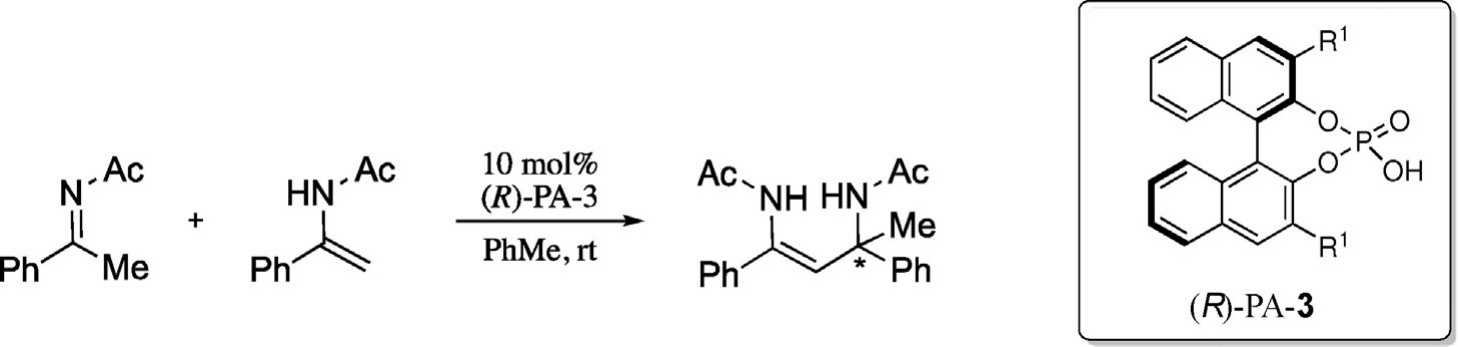				
Entry	Catalyst R^1^	AREA (θ)	Rotation barrier [kcal mol^−1^]	*ee* [%]
1	H	107	0.00	4
2	Ph	70	2.05	56
**3**	**4‐NO_2_C_6_H_4_**	**64**	**2.03**	**96**
**4**	**9‐anthryl**	**61**	**28.31**	**25**
**5**	**SiPh_3_**	**29**	**1.35**	**9**

### Qualitative model for catalyst choice

**Figure 11:**
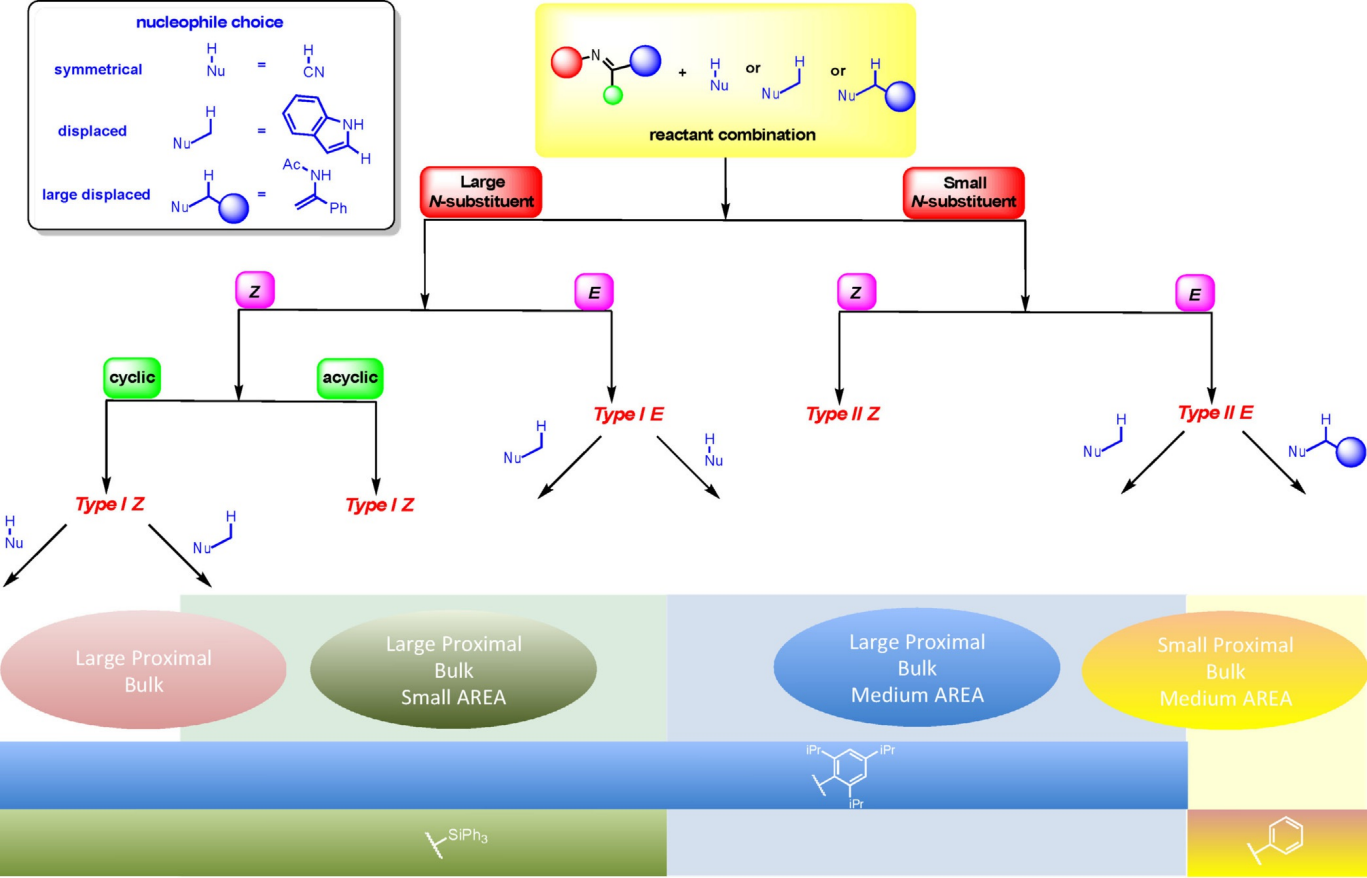
Qualitative model for catalyst choice.

By studying a number of reactions for this class, we have been able to observe broad trends. This has provided unprecedented insight into the steric factors that affect the enantioselectivity. Precise tuning of the steric environment of the 3,3′ can destabilize particular TS pathways, and by exploiting this finding we were able to construct a simple qualitative model to determine the steric features necessary for efficient stereoinduction (Figure 11). The model categorizes all reported imine/nucleophile reactant combinations and provides firstly a starting reference point for the design of selective phosphoric acid catalyzed reactions of this type, and secondly the foundations for new selective catalysts. The TS pathway in operation (Type I/II *E*/*Z*) in combination with the identity of the nucleophile determines which catalysts are selective. The reactants can be combined in different ways leading to eight different outcomes and four different catalyst choices, controlled by the factors listed: size of N‐substituent, imine configuration, acyclic or cyclic imine, size of the displaced nucleophile. The reactants follow either right‐handed or left‐handed pathways depending on the size of N‐substituent. Reactions with imines bearing large N‐substituents favor Type I pathways; a preference for *E* or *Z* depends on how accessible the configuration is. If the *Z* configuration is energetically inaccessible the reaction proceeds via a Type I *E* pathway, the correct catalyst has large proximal and medium AREA(θ).^10^, ^11^, ^12^, 13c, 14b, 19, 20, 21, 22, 40, 41, 42, 43, 44, 45, 46, 47 However, reactions involving displaced nucleophiles the best choice of catalyst has large proximal and small AREA(θ).^5^, ^8^, ^9^, ^30^, ^31^, ^48^, ^49^, ^50^, ^51^, ^52^, ^53^ Higher enantioselectivities with small proximal bulk catalysts are not uncommon with displaced nucleophiles. In these cases the competing Type II TS is likely to adopt a tilted disposition meaning the remote sterics are predominantly responsible for the enantioselectivity. For Type I *Z* reactions the correct catalyst is dependent on the nature of the imine and nucleophile. Reactions involving acyclic imines require a catalyst that has large proximal sterics to disfavor Type II pathways and small AREA(θ) to disfavor competing Type I *E* pathway.^6^, ^7^, 14b, 23, 54, 55, 56, 57, 58, 59, 60 If the imine is cyclic, the optimal catalyst is dependent on the nature of the nucleophile. For symmetrical nucleophiles like Hantszch esters only proximal sterics affect the TS to an appreciable extent and so the optimal catalyst has large proximal sterics disfavoring the Type II competing pathway.^24^, ^25^, ^26^, ^27^, ^28^, ^61^, ^62^, ^63^, ^64^, ^65^, ^66^, ^67^, ^68^ For Type I pathways with displaced nucleophiles the competing Type II TS arranges the imine towards a tilted disposition due to a greater steric accessibility, as a consequence both proximal and remote sterics play important stereochemical roles. The optimal catalyst has large proximal and small AREA(θ) disfavouring the Type II pathway.14a, 69, 70, 71 In a similar manner, reactions involving imine with small substituents favor Type II pathways and can exist as the *E* or *Z* isomers. For Type II *E*, a selective catalyst for such a reaction is one that has small AREA(θ) disfavoring Type I pathways. However, whether the reaction benefits from large proximal sterics or small depends on the sterics of the nucleophile. For smaller displaced nucleophiles, large proximal sterics are advantageous, disfavouring Type I *E* pathways.^29^, ^33^, ^35^, ^36^, ^37^, ^38^, ^72^, ^73^, ^74^, ^75^, ^76^, ^77^, ^78^ However, if the nucleophile is large, small proximal sterics are essential.^32^, ^34^, ^39^, ^79^, ^80^ The corresponding *Z* pathway is not known with acyclic imines. Unlike Type I reactions involving cyclic substrates and symmetrical nucleophiles in which only proximal sterics had a major influence on stereoselectivity, both proximal and remote sterics will play important stereochemical roles if the reaction proceeds via a Type II pathway. For such a pathway to be favoured the imine must adopt a tilted disposition regardless of whether the nucleophile employed is symmetrical or displaced and so the correct catalyst has large proximal and medium AREA(θ), showing the same enantioselectivity trends as observed with the Friedel–Crafts study (Table 4).^81^, ^82^, ^83^, ^84^ The only examples of Type II reactions and displaced nucleophiles employ sterically small nucleophiles such as indoles, whether the dependence on size of proximal sterics correlates inversely to size of displaced nucleophile as observed with the acyclic imines (Table 7) remains an open question but we would expect to observe similar trends.

**Table 7:** Examples of the addition of displaced nucleophiles to imines in which the sterics of the nucleophile dictate whether large or small proximal setrics are optimal. All reactions proceed via Type II *E* pathways. For each catalyst classification, we have chosen to compare the best performer from the catalyst screen of a model substrate under the same reaction conditions.

Reaction	Nu carbon substituent	Rotation barrier <3 [% *ee*]	Medium rotation barrier [% *ee*]	Rotation barrier >26 [% *ee*]
addition of enamides to *N*‐Boc imines^36^	H	79	–	91
Petasis Ferrier^37^	H	–	–	99
addition of vinyl indoles to *N*‐Boc imines^38^	H	21	54	92
Bignelli^32^	alkyl	99	64	1
Mannich of *N*‐Ar imines^39^	Ph	76	51	–

Although the choice of catalyst depends on the individual substrate combinations the model has supplied some insight into the correct selection of catalyst for a given process and we have included some suggestions. TRIP works well for seven of the eight reaction combinations explaining why it is so general. It should be avoided if the reaction employs large displaced nucleophiles and imines as these generally proceed via Type II *E* pathways. Useful catalysts for this reaction category will contain hydrogens at the 2 and 6 positions of the aromatic ring, for example, phenyl. If TRIP provides less than satisfactory enantioselectivities for reactions requiring large proximal bulk and small AREAs we suggest that the triphenylsilane derived phosphoric acid will be more appropriate.

## Conclusion

A general model to guide the best choice of phosphoric acid catalyst for nucleophilic addition to imines has been developed. These rules are consistent for seventy seven papers containing over 1000 examples, explaining enantioselectivity trends with varying 3,3′ groups and correctively identifying steric features essential for efficient stereoinduction. The literature presented may not be exhaustive but we have yet to find a reaction that does not fit the model. Although some of the pathways have yet to be experimentally explored, extension of the principles presented here will allow the results of any BINOL‐phosphoric acid catalysed nucleophilic addition to imines to be predicted. The insights gained in this study can be generalized to countless situations in which our molecular parameters can effectively describe the steric features required for efficient stereoinduction. The purpose of this study was to evaluate the steric effects of the catalyst for a wide range of reactions as such we did not include an electronic parameter in our analysis. As shown in cases where the parameters defect (Table 2), can lead to exposure of the catalyst electronic features important for asymmetric induction. The fact that this class of reactions although varied (over 1000 examples) behave generally allows the potential for applying a multivariate parameterization approach not just for one reaction but for many. This could allow a single model capable of predicting with a quantitative output the correct catalyst for a given transformation. These goals are on going in our research program.

## Computational Methods

For the QM/MM hybrid calculations on the full catalyst and reagent system, transition states were located first, by conformational search in MacroModel (version 9.9)^85^ using the OPLS‐2005 force field.^86^, ^87^, ^88^ Selected conformers within 10 kJ mol^−1^ of the minimum were optimized using the ONIOM method implemented in Gaussian 09 (revision D.01).^89^ The B3LYP density functional,^90^, ^91^ and split‐valence polarized 6‐31G** basis set,^92^, ^93^ were used for the high‐layer, and the force field UFF,^94^ was used for the low‐layer unless stated otherwise. The reactants and the phosphoric acid moiety of the catalyst were included in the high‐layer, and the remaining regions of the catalyst were treated as the low‐layer. This method has previously been shown to give excellent results when used to describe reactions catalyzed by chiral phosphoric acids.^4^, ^16^, ^18^, ^34^, ^35^, ^36^, ^37^

The position of the partition within the catalyst was chosen as the phosphoric acid binds directly to the reagents, whereas the remaining catalyst acts as steric bulk and can be adequately described by molecular mechanics. All calculations were performed with the (*S*)‐catalyst for model consistency with the connectivity shown in the corresponding reaction Scheme. We use the Kekulé bonding structure for all catalysts ensuring that the connectivity in the catalyst backbone is consistent between the structures allowing for accurate energy and geometry comparisons. We have re‐optimized the lowest energy TS structures using the delocalized bonding arrangement and have concluded that it does not affect the relative energies to an appreciable extent and the data is available for comparison in the Supporting Information. Single point energy calculations were performed on the resulting structures using M06‐2X density functional,^99^ and the 6‐31G** basis set, using non‐default convergence criteria (fine grid density, ultrafine accuracy level) as implemented in the Jaguar program (version 7.9).^100^ This energy was used to correct the gas‐phase energy derived from the ONIOM calculations. Free energies in solution were derived from structures optimized in the gas phase at the ONIOM (B3LYP/6‐31G**:UFF), level of theory by means of a single point calculation using M06‐2X/6‐31G** with the polarizable continuum model (PCM) as implemented in the Jaguar program (version 7.9), using benzene (probe radius=2.60 Å) for the transfer hydrogenation study, DCM (probe radius=2.33 Å) for the Friedel–Crafts study and toluene (probe radius=2.76 Å) for the addition of enamides, as the solvent.^101^ These values were used to correct the Gibbs free energy derived from the ONIOM calculations.

The quantitative parameters were calculated as described previously.^18^ Structures are illustrated using CYLview.^102^

Supporting information for this article: Complete list of authors in the Gaussian 09 reference, Cartesian coordinates of all the catalyst structures, Cartesian coordinates, energies, and values of imaginary frequencies of all the transition state structures.

## Conflict of interest

The authors declare no conflict of interest.

## Supporting information

As a service to our authors and readers, this journal provides supporting information supplied by the authors. Such materials are peer reviewed and may be re‐organized for online delivery, but are not copy‐edited or typeset. Technical support issues arising from supporting information (other than missing files) should be addressed to the authors.

Supplementary
