# Supplementary material for: Selecting Chiral BINOL‐Derived Phosphoric Acid Catalysts: General Model To Identify Steric Features Essential for Enantioselectivity
Source: Chemistry. 2017 Sep 14;23(57):14248–60. doi: 10.1002/chem.201702019 (PMC5656902; doi:10.1002/chem.201702019)
Supplement: Supplementary file 1 — Supplementary [file CHEM-23-14248-s001.pdf]

CHEMISTRY
A European Journal

Supporting Information

Selecting Chiral BINOL-Derived Phosphoric Acid Catalysts: General Model To Identify Steric Features Essential for Enantioselectivity

Jolene P. Reid and Jonathan M. Goodman*[a]

chem_201702019_sm_miscellaneous_information.pdf

Selecting Chiral BINOL-derived Phosphoric Acid Catalysts: General Model to Identify Steric Features Essential For Enantioselectivity

Jolene P. Reid and Jonathan M. Goodman[*]

Centre for Molecular Informatics, Department of Chemistry, University of Cambridge, Lensfield Road, Cambridge CB2 1EW, United Kingdom

Supporting Information

List of contents:

- Full list of authors in the Gaussian09 reference. Page S2.
- Computational methods. Pages S2-S3
- Cartesian coordinates of all the catalyst structures. Pages S4-S19
- Transfer hydrogenation study. Cartesian coordinates, energies, number and values of imaginary frequencies of all the transition state structures. Comparison of ONIOM methods and TS structures for additional catalysts. Pages S19-S51
- Friedel-Crafts study. Cartesian coordinates, energies, number and values of imaginary frequencies of all the transition state structures. S52-S76
- Addition of enamides study. Cartesian coordinates, energies, number and values of imaginary frequencies of all the transition state structures. S76-S99
- Re-optimization of TS structures using delocalised bonding model. S99-137
- References. Page S138-139

Full list of authors in the Gaussian09 reference

M. J. Frisch, G. W. Trucks, H. B. Schlegel, G. E. Scuseria, M. A. Robb, J. R. Cheeseman, G. Scalmani, V. Barone, B. Mennucci, G. A. Petersson, H. Nakatsuji, M. Caricato, X. Li, H. P. Hratchian, A. F. Izmaylov, J. Bloino, G. Zheng, J. L. Sonnenberg, M. Hada, M. Ehara, K. Toyota, R. Fukuda, J. Hasegawa, M. Ishida, T. Nakajima, Y. Honda, O. Kitao, H. Nakai, T. Vreven, J. A. Montgomery, Jr., J. E. Peralta, F. Ogliaro, M. Bearpark, J. J. Heyd, E. Brothers, K. N. Kudin, V. N. Staroverov, T. Keith, R. Kobayashi, J. Normand, K. Raghavachari, A. Rendell, J. C. Burant, S. S. Iyengar, J. Tomasi, M. Cossi, N. Rega, J. M. Millam, M. Klene, J. E. Knox, J. B. Cross, V. Bakken, C. Adamo, J. Jaramillo, R. Gomperts, R. E. Stratmann, O. Yazyev, A. J. Austin, R. Cammi, C. Pomelli, J. W. Ochterski, R. L. Martin, K. Morokuma, V. G. Zakrzewski, G. A. Voth, P. Salvador, J. J. Dannenberg, S. Dapprich, A. D. Daniels, O. Farkas, J. B. Foresman, J. V. Ortiz, J. Cioslowski, and D. J. Fox, Gaussian, Inc., Wallingford CT, 2013.

Computational Methods

For the QM/MM hybrid calculations on the full catalyst, transition states were located first, by a conformational search in MacroModel (version 9.9)[1] using the OPLS-2005 force field.[2-4] Selected conformers within 10 kJ mol^{-1} of the minimum were optimized using the ONIOM method implemented in Gaussian 09 (revision D.01).[5] The B3LYP density functional,[6-7] and split-valence polarized 6-31G** basis set,[8-9] were used for the high-layer, and the force field UFF,[10] was used for the low-layer unless stated otherwise. The reactants and the phosphoric acid moiety of the catalyst were included in the high-layer, and the remaining regions of the catalyst were treated as the low-layer. This method has previously been shown to give excellent results when used to describe reactions catalyzed by chiral phosphoric acids.[11-15] The position of the partition within the catalyst was chosen as the phosphoric acid binds directly to the reagents, whereas the remaining catalyst acts as steric bulk and can be adequately

described by molecular mechanics. We use the Kekulé bonding structure for all catalysts ensuring that the connectivity in the catalyst backbone is consistent between the structures allowing for accurate energy and geometry comparisons.[16] Single point energy calculations were performed on the resulting structures using M06-2X density functional,[17] and the 6-31G** basis set, using non-default convergence criteria (fine grid density, ultrafine accuracy level) as implemented in the Jaguar program (version 7.9).[18] This energy was used to correct the gas-phase energy derived from the ONIOM calculations. Free energies in solution were derived from structures optimized in the gas phase at the ONIOM (B3LYP/6-31G**:UFF), level of theory by means of a single point calculation using M06-2X/6-31G** with the polarizable continuum model (PCM) as implemented in the Jaguar program (version 7.9), using benzene (probe radius = 2.60 Å) for the transfer hydrogenation study, DCM (probe radius = 2.33 Å) for the Friedel-Crafts study and toluene (probe radius = 2.76 Å) for the addition of enamides, as the solvent.[19] These values were used to correct the Gibbs free energy derived from the ONIOM calculations. To further validate the results for the transfer hydrogenation study, the lowest energy conformation from this process was re-optimized using the ONIOM method implemented in Gaussian 09 (revision D.01), M06-2X/6-31G** was used for the high-layer, and the force field UFF, was used for the low-layer. The free energies obtained from this process were corrected by a single point calculation in the same manner as above. This combination of DFT and UFF has previously been shown to give better results than ONIOM (B3LYP/6-31G**:UFF) for difficult cases.[15]

The quantitative parameters were calculated as described previously.[15] Structures are illustrated using CYLview.[20]

(*S*)-1,1’-Binaphthyl-2,2’-diyl hydrogenphosphate

MM (OPLS-2005) Geometry

C	-0.20990	-2.11200	0.17720	C	-1.58770	-0.09460	0.93880
C	1.44670	-3.41350	-1.73650	C	-1.21820	0.66340	-0.20030
C	-0.63220	-3.29520	-0.47720	C	-1.64310	1.99670	-0.35350
H	2.06420	-3.91060	-2.47060	C	-2.45370	2.59500	0.62670
C	-2.86130	-0.21280	3.03170	C	-2.84850	1.85340	1.75450
C	-1.67010	-2.17400	2.21420	H	-0.60410	0.23420	-0.97790
C	-1.15060	-1.44100	1.11560	H	-1.34610	2.55930	-1.22680
H	-3.50140	0.24650	3.77080	H	-2.78010	3.61830	0.50950
C	1.11110	-1.63860	-0.07590	H	-3.48280	2.31560	2.49690
C	1.92570	-2.28550	-1.04490	P	-1.98370	-4.61190	1.30130
C	3.22620	-1.81230	-1.31780	O	-1.82090	-3.86800	-0.13240
C	3.73860	-0.70370	-0.62040	O	-1.40800	-3.50850	2.34790
C	2.95270	-0.06950	0.35770	O	-1.40650	-5.97880	1.39870
C	1.65260	-0.53580	0.62990	O	-3.57570	-4.55170	1.55210
H	3.84120	-2.30300	-2.05830	H	-3.91280	-5.42340	1.67180
H	4.73670	-0.34660	-0.82900	C	-2.49520	-1.55960	3.17380
H	3.34670	0.77590	0.90320	H	-2.86430	-2.13190	4.01230
H	1.08030	-0.03080	1.39370	C	0.17510	-3.92760	-1.43990
C	-2.42870	0.51560	1.90880	H	-0.17330	-4.82370	-1.93270

(*R*)-3,3’-Bis(phenyl)-1,1’-binaphthyl-2,2’-diyl hydrogenphosphate

MM (OPLS-2005) Geometry

C	-0.88470	-0.76620	0.73750	C	-2.55220	1.02810	0.96990
C	-3.50530	0.23230	0.31020	C	-2.91650	2.31940	1.40580
C	-3.19450	-1.07760	-0.09980	C	-1.99050	3.12260	2.09510
C	-1.88840	-1.56650	0.13280	C	-0.69910	2.63210	2.35600
H	-4.49880	0.61880	0.13490	C	-0.33110	1.34600	1.91770
C	3.04340	-2.44870	1.38840	H	-3.91150	2.69790	1.22100
C	1.90560	-3.08450	1.91970	H	-2.27360	4.10980	2.43100
C	0.63510	-2.52690	1.65120	H	0.01230	3.24160	2.89440
C	0.48230	-1.33080	0.90570	H	0.66620	0.99890	2.14290
H	4.02050	-2.86500	1.58610	C	2.92040	-1.30200	0.58300
C	-1.24390	0.53060	1.20340	C	1.64150	-0.74400	0.32430

C	1.55740	0.38490	-0.52770	C	-3.98100	-2.59590	-1.94460
C	2.71670	0.96080	-1.08120	H	-3.01520	-2.50460	-2.42120
C	3.97980	0.41220	-0.79800	C	2.81490	-5.40130	2.23130
C	4.08050	-0.71970	0.03080	C	2.96500	-6.57720	2.99220
H	0.60270	0.82640	-0.77190	H	3.54150	-7.40270	2.60080
H	2.63430	1.82430	-1.72530	C	1.60000	-5.60400	4.75720
H	4.87000	0.85360	-1.22260	H	1.12970	-5.67820	5.72680
H	5.05360	-1.14300	0.23400	C	2.35770	-6.67920	4.25740
P	-1.38710	-3.99020	0.96740	H	2.46940	-7.58170	4.84090
O	-1.60790	-2.86640	-0.18300	C	-6.50110	-2.85150	-0.72970
O	-0.48580	-3.19330	2.05600	H	-7.46850	-2.94850	-0.25850
O	-0.86600	-5.30370	0.50450	C	-4.97240	-3.39730	-2.54330
O	-2.82920	-4.06180	1.68540	H	-4.76210	-3.91300	-3.46920
H	-3.21410	-4.91000	1.54350	C	-6.23490	-3.52580	-1.93570
C	2.05650	-4.31410	2.71960	H	-6.99680	-4.14030	-2.39360
C	-4.23230	-1.91440	-0.73240	C	-5.50550	-2.05190	-0.13510
C	1.45240	-4.43100	3.99150	H	-5.71930	-1.54680	0.79580
H	0.86800	-3.61150	4.38430	H	3.27580	-5.34000	1.25610

(*R*)-3,3’-Bis(1-naphthyl)-1,1’-binaphthyl-2,2’-diyl hydrogenphosphate

MM (OPLS-2005) Geometry

C	-0.59890	-0.94190	0.47280	H	-2.95530	2.80280	1.83260
C	-3.10800	0.33070	0.86550	H	-0.88720	4.01110	2.46720
C	-3.06870	-0.99580	0.40260	H	1.32670	2.90320	2.21440
C	-1.81770	-1.62230	0.22260	H	1.48150	0.62390	1.32020
H	-4.06520	0.81280	1.00430	C	2.91090	-1.82330	-0.83210
C	3.16390	-2.99360	-0.09430	C	1.66940	-1.14800	-0.69740
C	2.17930	-3.53620	0.74990	C	1.43990	0.00510	-1.48820
C	0.94010	-2.87100	0.86780	C	2.42900	0.48890	-2.36550
C	0.68720	-1.64250	0.20740	C	3.66270	-0.17690	-2.47250
H	4.11130	-3.50420	-0.19140	C	3.90150	-1.33320	-1.70830
C	-0.66240	0.37430	1.01540	H	0.50120	0.53600	-1.43650
C	-1.92040	1.00920	1.19110	H	2.23840	1.37210	-2.95810
C	-1.99670	2.32030	1.70580	H	4.42220	0.19350	-3.14590
C	-0.82550	3.00820	2.06950	H	4.84750	-1.84660	-1.80450
C	0.42500	2.38230	1.92580	P	-1.33250	-4.11910	0.84530
C	0.50480	1.07580	1.40770	O	-1.80120	-2.93390	-0.16100

O	-0.05990	-3.44760	1.59720	C	3.14000	-4.79410	2.72990
O	-1.10640	-5.44470	0.20920	C	3.59080	-3.59490	3.33240
O	-2.47510	-4.08920	1.98510	C	4.27100	-3.62200	4.56510
H	-3.32750	-3.93100	1.61280	C	4.50880	-4.84800	5.21270
C	2.45320	-4.78210	1.48640	C	4.06450	-6.04810	4.62760
C	-4.31860	-1.72570	0.13010	H	3.41870	-2.64000	2.85880
C	2.02150	-6.00650	0.93340	H	4.60900	-2.69950	5.01430
H	1.48840	-6.01180	-0.00710	H	5.02980	-4.86850	6.15890
C	-4.70890	-1.91560	-1.21270	H	4.24840	-6.98550	5.13190
H	-4.09530	-1.51770	-2.00850	C	-6.31180	-2.97900	0.83720
C	-5.88240	-2.62070	-1.53460	C	-5.12950	-2.26320	1.16520
H	-6.16490	-2.75710	-2.56850	C	-4.77840	-2.11430	2.52860
C	2.94080	-7.23650	2.82230	C	-5.58450	-2.66660	3.54200
H	3.11820	-8.17620	3.32450	C	-6.75430	-3.37190	3.20710
C	-6.68420	-3.15200	-0.51000	C	-7.11770	-3.52670	1.85680
H	-7.58280	-3.69590	-0.76420	H	-3.88460	-1.58150	2.81960
C	2.25840	-7.22520	1.59370	H	-5.30280	-2.54910	4.57880
H	1.90990	-8.14970	1.15670	H	-7.37260	-3.79530	3.98570
C	3.38260	-6.02670	3.39300	H	-8.01710	-4.07140	1.60830

(*R*)-3,3’-Bis(2-naphthyl)-1,1’-binaphthyl-2,2’-diyl hydrogenphosphate

MM (OPLS-2005) Geometry

C	-2.50830	-1.38390	2.61410	H	-5.71020	-0.96890	5.92750
C	-5.07630	-2.08340	3.59800	H	-4.26680	0.51060	7.29410
C	-4.62570	-2.64970	2.39120	H	-1.97660	1.08380	6.50720
C	-3.33830	-2.30130	1.92120	H	-1.14070	0.21010	4.37280
H	-6.05950	-2.33720	3.96660	C	0.49140	0.60210	1.25790
C	1.43040	-0.43380	1.10790	C	-0.82350	0.31430	1.70790
C	1.06910	-1.77120	1.34750	C	-1.74940	1.38180	1.81190
C	-0.24650	-2.05650	1.77660	C	-1.36910	2.70160	1.50310
C	-1.18550	-1.02420	2.03280	C	-0.05710	2.97580	1.07850
H	2.43340	-0.21230	0.77270	C	0.87080	1.92620	0.95410
C	-2.95930	-0.88080	3.86810	H	-2.76680	1.20550	2.12800
C	-4.25230	-1.22170	4.34320	H	-2.08740	3.50390	1.59170
C	-4.72260	-0.71260	5.57190	H	0.23530	3.98850	0.84110
C	-3.90540	0.12530	6.35150	H	1.87420	2.14290	0.61670
C	-2.61210	0.44940	5.90630	P	-1.68580	-4.03040	0.87920
C	-2.14230	-0.05290	4.67800	O	-2.86030	-2.91390	0.79850

O	-0.63570	-3.35880	1.92100	H	5.74830	-6.03980	1.31000
O	-1.13520	-4.48540	-0.42550	C	-7.12580	-5.46390	0.27730
O	-2.35970	-5.20150	1.75920	C	-6.53180	-4.38290	-0.41750
H	-2.41810	-5.99160	1.24910	C	-6.75400	-4.23200	-1.80190
C	2.06440	-2.83510	1.14980	C	-7.56560	-5.15410	-2.49140
C	-5.48280	-3.60590	1.66370	C	-8.15730	-6.22890	-1.79960
C	1.99550	-3.69230	0.03160	C	-7.93800	-6.38430	-0.41670
H	1.20290	-3.56250	-0.69080	H	-6.30290	-3.41120	-2.34040
C	-5.72030	-3.46380	0.27800	H	-7.73360	-5.03650	-3.55220
H	-5.26970	-2.63930	-0.25530	H	-8.77940	-6.93520	-2.33000
C	3.99330	-4.88270	0.78530	H	-8.39520	-7.21170	0.10610
C	2.94570	-4.71650	-0.15260	C	-6.08370	-4.68900	2.34310
C	2.85600	-5.57700	-1.26580	H	-5.91000	-4.82240	3.40110
C	3.80900	-6.59850	-1.44670	C	-6.89870	-5.61170	1.65930
C	4.85320	-6.76220	-0.51550	H	-7.34450	-6.43340	2.20040
C	4.94560	-5.90610	0.59970	C	3.11490	-3.00700	2.07640
H	2.05360	-5.45830	-1.98010	H	3.18200	-2.35610	2.93590
H	3.73670	-7.25680	-2.30010	C	4.07460	-4.02260	1.89830
H	5.58310	-7.54630	-0.65560	H	4.86870	-4.13940	2.62090

(*R*)-3,3'-Bis(9-phenanthrenyl)-1,1'-binaphthyl-2,2'-diyl hydrogenphosphate

MM (OPLS-2005) Geometry

C	-0.40730	-1.06830	0.19780	C	0.87810	0.96610	0.80710
C	-2.79400	0.45560	0.41150	H	-2.41920	3.02210	0.99530
C	-2.87530	-0.92430	0.15430	H	-0.24600	4.13360	1.42320
C	-1.68280	-1.67370	0.06460	H	1.86390	2.81810	1.32120
H	-3.70510	1.03250	0.47990	H	1.81210	0.42520	0.77970
C	3.16380	-3.48710	-0.10850	C	2.98420	-2.42300	-1.01030
C	2.16350	-3.81350	0.82410	C	1.80480	-1.63450	-0.95970
C	0.98390	-3.03880	0.85810	C	1.64640	-0.59690	-1.91150
C	0.81130	-1.90560	0.02400	C	2.64620	-0.33290	-2.86680
H	4.06470	-4.08280	-0.14260	C	3.81920	-1.10750	-2.89150
C	-0.35090	0.31470	0.53600	C	3.98610	-2.15290	-1.96570
C	-1.54850	1.07230	0.62380	H	0.75480	0.01210	-1.92600
C	-1.50620	2.44740	0.93520	H	2.51040	0.46520	-3.58250
C	-0.27500	3.08090	1.18110	H	4.58690	-0.90570	-3.62470
C	0.91670	2.33750	1.12260	H	4.88510	-2.75160	-1.99810

P	-1.39060	-4.06760	1.07130	C	-6.77890	-2.34450	3.26840
O	-1.77600	-3.02490	-0.11220	C	-7.17380	-2.69810	1.96590
O	-0.03600	-3.41500	1.68440	H	-3.72430	-1.03360	2.59220
O	-1.32000	-5.49930	0.67390	H	-5.22090	-1.47370	4.48320
O	-2.48320	-3.74200	2.21080	H	-7.43530	-2.54020	4.10420
H	-3.10960	-4.44340	2.27190	H	-8.14070	-3.16100	1.86510
C	2.35730	-4.95400	1.73740	C	2.66850	-7.23500	3.42360
C	-4.19060	-1.57020	-0.01180	C	1.90450	-7.31020	2.22970
C	1.76340	-6.18340	1.40330	C	1.27130	-8.51170	1.84970
H	1.17870	-6.25710	0.49720	C	1.39350	-9.66110	2.64880
C	-4.58280	-1.94220	-1.30980	C	2.15060	-9.60790	3.83120
H	-3.91240	-1.74890	-2.13490	C	2.78060	-8.40830	4.21550
C	3.28250	-5.99760	3.77780	H	0.68350	-8.55300	0.94360
C	3.11870	-4.86050	2.93140	H	0.90590	-10.57930	2.35500
C	3.71950	-3.62890	3.29060	H	2.24700	-10.48910	4.44870
C	4.48080	-3.51070	4.46530	H	3.34330	-8.42940	5.13300
C	4.65030	-4.62610	5.29900	C	-6.71430	-2.81870	-0.47600
C	4.05700	-5.85500	4.95910	C	-5.82310	-2.55680	-1.54950
H	3.60370	-2.75240	2.67090	C	-6.16280	-2.90640	-2.87330
H	4.93240	-2.56440	4.72550	C	-7.39570	-3.52100	-3.15070
H	5.23460	-4.54040	6.20380	C	-8.29110	-3.78640	-2.10130
H	4.21980	-6.67360	5.63920	C	-7.95490	-3.43970	-0.77860
C	-6.33170	-2.45540	0.84880	H	-5.47770	-2.70520	-3.68420
C	-5.06710	-1.83360	1.07330	H	-7.65290	-3.78820	-4.16550
C	-4.68330	-1.48830	2.39250	H	-9.24010	-4.25900	-2.30990
C	-5.53110	-1.74120	3.48330	H	-8.68240	-3.66960	-0.01890

(*R*)-3,3’-Bis(9-anthracenyl)-1,1’-binaphthyl-2,2’-diyl hydrogenphosphate

MM (OPLS-2005) Geometry

C	-0.90920	-0.75980	0.78820	C	0.46580	-1.32450	0.87040
C	-3.53400	0.27330	0.46460	H	4.05020	-2.81890	1.39060
C	-3.24810	-1.03470	0.03530	C	-1.24160	0.53200	1.28890
C	-1.94500	-1.54550	0.22140	C	-2.55220	1.04670	1.10820
H	-4.53090	0.66620	0.32490	C	-2.88880	2.33350	1.57750
C	3.06240	-2.40960	1.23400	C	-1.93200	3.11610	2.24820
C	1.95240	-3.07510	1.78420	C	-0.63730	2.60950	2.45610
C	0.66370	-2.53990	1.57160	C	-0.29690	1.32760	1.98400

H	-3.88580	2.72430	1.43250	C	-5.35770	-2.81760	-2.60290
H	-2.19390	4.10000	2.60990	C	-5.39850	-2.99460	-4.00190
H	0.09810	3.20350	2.97950	C	-4.43140	-2.38650	-4.82210
H	0.70420	0.96810	2.16890	C	-3.42120	-1.59810	-4.24380
C	2.89090	-1.25210	0.45430	C	-3.37770	-1.41890	-2.84800
C	1.59400	-0.71000	0.25560	H	-6.17200	-3.59860	-4.45350
C	1.46120	0.43420	-0.56950	H	-4.46440	-2.52520	-5.89300
C	2.58970	1.03850	-1.15550	H	-2.67590	-1.13000	-4.87060
C	3.87080	0.50420	-0.93190	H	-2.58720	-0.80740	-2.43990
C	4.02000	-0.64140	-0.12990	C	2.58270	-6.73650	2.67510
H	0.49170	0.86600	-0.76850	C	2.37460	-5.55710	1.91350
H	2.47010	1.91310	-1.77850	C	2.39070	-5.65580	0.50000
H	4.73750	0.96740	-1.38130	C	2.61380	-6.89170	-0.13610
H	5.00640	-1.05350	0.02800	C	2.82640	-8.05090	0.63030
P	-1.39900	-3.99450	0.96870	C	2.80970	-7.97270	2.03440
O	-1.69690	-2.84080	-0.13500	H	2.21460	-4.79080	-0.12130
O	-0.42400	-3.23200	2.01860	H	2.61070	-6.94890	-1.21480
O	-0.91810	-5.29460	0.43240	H	2.99270	-8.99990	0.14180
O	-2.78580	-4.08290	1.78470	H	2.96640	-8.86880	2.61670
H	-3.23470	-4.88440	1.57440	C	2.33500	-5.46360	4.73880
C	2.15010	-4.31320	2.56750	C	2.13060	-4.27540	3.99030
C	-4.30680	-1.85400	-0.59290	C	1.91430	-3.06400	4.69410
C	-6.31550	-3.26970	-0.40790	C	1.89670	-3.03850	6.10180
C	-5.30550	-2.48240	0.20370	C	2.09740	-4.22390	6.83070
C	-5.31760	-2.34900	1.61470	C	2.31700	-5.43440	6.14920
C	-6.30660	-2.97960	2.39300	H	1.75630	-2.13490	4.16780
C	-7.30320	-3.75390	1.77370	H	1.72780	-2.10660	6.62160
C	-7.30670	-3.89850	0.37480	H	2.08360	-4.20520	7.91080
H	-4.56130	-1.77220	2.12620	H	2.47190	-6.34130	6.71540
H	-6.29660	-2.86950	3.46790	C	-6.33630	-3.43320	-1.80360
H	-8.06300	-4.23770	2.37050	H	-7.10680	-4.03410	-2.26410
H	-8.07470	-4.49630	-0.09430	C	2.56120	-6.68420	4.07950
C	-4.34200	-2.02670	-2.00550	H	2.71700	-7.58570	4.65340

(*R*)-3,3'-Bis(triphenylsilyl)-1,1'-binaphthyl-2,2'-diyl hydrogenphosphate

MM (OPLS-2005) Geometry

C	-0.77630	-0.83310	0.88400	C	-3.19670	-0.90730	0.36530
C	-3.33570	0.37790	0.92140	C	-1.91400	-1.51030	0.36470

H	-4.29950	0.86670	0.89800
C	3.15380	-2.61880	0.71700
C	2.10630	-3.28720	1.37720
C	0.81530	-2.71000	1.34070
C	0.57630	-1.44410	0.75270
H	4.13920	-3.06380	0.71160
C	-0.97030	0.41790	1.53660
C	-2.25140	1.02370	1.53600
C	-2.45390	2.27910	2.14600
C	-1.38690	2.93720	2.78330
C	-0.11580	2.33770	2.81320
C	0.08950	1.08860	2.19720
H	-3.43090	2.74050	2.13580
H	-1.54530	3.89660	3.25440
H	0.70550	2.83530	3.30870
H	1.07780	0.65600	2.24440
C	2.92910	-1.40780	0.03990
C	1.64070	-0.81480	0.04770
C	1.44930	0.39250	-0.66890
C	2.51700	1.00380	-1.35330
C	3.79310	0.41380	-1.33860
C	3.99760	-0.79220	-0.64440
H	0.48010	0.86670	-0.70840
H	2.35390	1.92580	-1.89250
H	4.61280	0.88130	-1.86500
H	4.97840	-1.24550	-0.64570
P	-1.29830	-4.05710	0.73430
O	-1.76630	-2.76920	-0.14860
O	-0.26150	-3.40340	1.79970
O	-0.78440	-5.20820	-0.05790
O	-2.59660	-4.36000	1.64020
H	-3.28180	-4.74780	1.11330
Si	2.51320	-4.92570	2.25360
Si	-4.71930	-1.66900	-0.48480
C	-1.33800	-6.97770	4.06610
C	-0.77120	-7.47780	2.87910
C	0.38810	-6.88140	2.34650
C	0.99630	-5.78420	2.99310
C	0.41290	-5.28500	4.17780
C	-0.74520	-5.87910	4.71570
H	-2.22960	-7.43130	4.47320
H	-1.22850	-8.31280	2.36870
H	0.80100	-7.26130	1.42280
H	0.85660	-4.43330	4.67390
H	-1.18210	-5.48660	5.62240
C	5.53310	-4.26260	5.83540
C	5.33540	-3.23480	4.89490
C	4.43020	-3.41690	3.83090
C	3.70510	-4.62180	3.69840
C	3.92150	-5.64870	4.64320
C	4.82550	-5.47200	5.70850
H	6.22700	-4.12420	6.65190
H	5.87860	-2.30550	4.98880
H	4.29110	-2.61540	3.12190
H	3.38290	-6.58190	4.54980
H	4.97580	-6.26510	6.42680
C	4.50570	-7.77150	-0.94400
C	5.09680	-7.62500	0.32500
C	4.51140	-6.77130	1.28060
C	3.33830	-6.04780	0.97540
C	2.74680	-6.21300	-0.29560
C	3.32760	-7.06640	-1.25330
H	4.95170	-8.42780	-1.67710
H	5.99780	-8.16980	0.56750
H	4.97260	-6.66980	2.25230
H	1.83420	-5.68330	-0.53620
H	2.86420	-7.18200	-2.22260
C	-4.73180	-0.44910	-5.04650
C	-5.44960	-1.58070	-4.61670
C	-5.43160	-1.95040	-3.25780
C	-4.68510	-1.20670	-2.31810
C	-3.98920	-0.05940	-2.75650
C	-4.00390	0.31420	-4.11460
H	-4.74430	-0.16340	-6.08840
H	-6.01660	-2.16350	-5.32850
H	-5.99370	-2.81530	-2.93350
H	-3.43360	0.53900	-2.04860
H	-3.45900	1.18840	-4.44060
C	-4.72610	-6.36980	0.02400
C	-5.42090	-5.56200	0.94270

C	-5.44200	-4.16360	0.77520	C	-7.56940	-0.34290	2.30200
C	-4.78170	-3.55700	-0.31580	C	-6.39260	-0.83690	1.70680
C	-4.08980	-4.37970	-1.23120	C	-6.28570	-0.95110	0.30300
C	-4.06040	-5.77750	-1.06450	C	-7.39510	-0.59290	-0.49330
H	-4.70180	-7.44280	0.15320	C	-8.57430	-0.09820	0.09720
H	-5.93370	-6.01430	1.78020	H	-9.56580	0.40790	1.94990
H	-5.96890	-3.55480	1.49590	H	-7.63600	-0.25320	3.37690
H	-3.55720	-3.93290	-2.06030	H	-5.56100	-1.12780	2.33380
H	-3.51490	-6.39340	-1.76630	H	-7.34330	-0.69130	-1.56830
C	-8.66100	0.02980	1.49600	H	-9.41350	0.18020	-0.52410

(*R*)-3,3’-Bis[4-(nitro)phenyl]-1,1’-binaphthyl-2,2’-diyl hydrogenphosphate

MM (OPLS-2005) Geometry

C	1.25650	-1.76210	-1.14500	C	6.49050	-1.89050	-1.25160
C	-0.83260	-0.76300	-2.78330	C	6.09280	-2.57060	-0.08650
C	-0.74690	-2.14800	-2.54880	H	3.43020	-1.12600	-2.52640
C	0.28900	-2.63010	-1.71580	H	5.81960	-0.85990	-3.02690
H	-1.61740	-0.38120	-3.42000	H	7.54040	-1.77290	-1.47870
C	4.34930	-3.43870	1.36750	H	6.84660	-2.97560	0.57330
C	2.99310	-3.65760	1.67360	P	-0.04300	-4.50380	0.07670
C	2.01220	-3.11980	0.81020	O	0.31890	-3.96300	-1.41130
C	2.35410	-2.34500	-0.32600	O	0.69760	-3.41730	1.02570
H	5.11050	-3.84360	2.01870	O	0.26190	-5.93320	0.34890
C	1.10740	-0.35990	-1.34310	O	-1.59920	-4.11360	0.23810
C	0.06910	0.12950	-2.17730	H	-2.09720	-4.87840	0.47340
C	-0.07830	1.51460	-2.40080	C	2.62320	-4.45580	2.85690
C	0.79310	2.42910	-1.78270	C	-1.74140	-3.05640	-3.15130
C	1.81040	1.95950	-0.93370	C	1.69500	-3.96730	3.80170
C	1.96300	0.57760	-0.71290	C	1.33770	-4.73470	4.92790
H	-0.86570	1.88460	-3.04160	H	0.62280	-4.33800	5.63450
H	0.67570	3.48970	-1.95290	C	2.83470	-6.50200	4.19350
H	2.47590	2.65940	-0.44900	H	3.27700	-7.47880	4.32720
H	2.75050	0.25400	-0.04870	C	3.18970	-5.73110	3.06890
C	4.72320	-2.73060	0.21100	H	3.89310	-6.13270	2.35340
C	3.73160	-2.19310	-0.65060	C	-3.12260	-2.79090	-3.03010
C	4.15030	-1.52810	-1.82950	H	-3.45660	-1.92050	-2.48320
C	5.51790	-1.37430	-2.12570	C	-4.07850	-3.65320	-3.60300

H	-5.12960	-3.42760	-3.49260	N	1.52820	-6.82040	6.31660
C	1.90310	-6.01310	5.13720	O	2.06770	-7.91210	6.46740
C	-3.67470	-4.80540	-4.31510	O	0.69760	-6.35940	7.09350
C	-1.34010	-4.20730	-3.86420	N	-4.67580	-5.71050	-4.91690
H	-0.28820	-4.43170	-3.97280	O	-4.27330	-6.69160	-5.53380
C	-2.29240	-5.07240	-4.43840	O	-5.86340	-5.43800	-4.77170
H	-1.95660	-5.94690	-4.97730	H	1.24880	-2.99310	3.66060

(*R*)-3,3’-Bis[4-(phenyl)phenyl]-1,1’-binaphthyl-2,2’-diyl hydrogenphosphate

MM (OPLS-2005) Geometry

C	1.71680	-3.10650	-0.37160	H	6.42610	-4.64120	-1.16850
C	0.64620	-1.62000	-2.53780	H	7.22830	-5.89600	0.82520
C	0.03840	-2.83540	-2.17230	H	5.70950	-6.26800	2.74770
C	0.57410	-3.55810	-1.08160	P	-0.89850	-4.73820	0.72500
H	0.24820	-1.06000	-3.37160	O	-0.06650	-4.69260	-0.66830
C	3.18700	-5.44690	2.95240	O	0.07960	-3.92700	1.73350
C	1.85360	-4.99690	2.93940	O	-1.34830	-6.08290	1.17270
C	1.39890	-4.26790	1.81760	O	-2.09400	-3.68690	0.46840
C	2.25480	-3.94450	0.73480	H	-2.92410	-4.13080	0.51020
H	3.54680	-6.00580	3.80410	C	-0.76890	-5.91580	6.26300
C	2.27160	-1.84170	-0.71850	C	-0.03250	-6.93150	5.61550
C	1.74030	-1.11330	-1.81430	C	0.82140	-6.63160	4.53650
C	2.29320	0.13080	-2.18380	C	0.96050	-5.30740	4.07060
C	3.36780	0.67310	-1.45670	C	0.22560	-4.29100	4.71690
C	3.88710	-0.02560	-0.35290	C	-0.62590	-4.59060	5.79790
C	3.34070	-1.26970	0.01510	H	-0.11750	-7.95510	5.94970
H	1.89070	0.68020	-3.02270	H	1.36460	-7.43120	4.05420
H	3.78700	1.62790	-1.73980	H	0.31240	-3.26880	4.37830
H	4.70590	0.39220	0.21510	H	-1.18410	-3.79270	6.26490
H	3.75940	-1.77410	0.87310	C	-3.45940	-4.23190	-4.31800
C	4.04150	-5.20480	1.86150	C	-2.35750	-5.08590	-4.09580
C	3.57900	-4.46640	0.74110	C	-1.21850	-4.63300	-3.40250
C	4.45690	-4.28350	-0.35580	C	-1.14830	-3.31350	-2.90720
C	5.76850	-4.79410	-0.32490	C	-2.24790	-2.45800	-3.13140
C	6.22190	-5.50360	0.80100	C	-3.38640	-2.91060	-3.82610
C	5.35780	-5.71090	1.89120	H	-2.37710	-6.09990	-4.46750
H	4.13860	-3.75010	-1.23910	H	-0.38940	-5.30880	-3.24920

H	-2.22660	-1.44550	-2.75530	H	-2.58420	-5.05680	10.48160
H	-4.21760	-2.23590	-3.96980	C	-6.95370	-5.62280	-6.43390
C	-3.39000	-6.83870	9.56360	C	-6.44360	-4.33540	-6.68220
C	-3.35750	-7.67430	8.43210	C	-5.30300	-3.88370	-5.98980
C	-2.50030	-7.37060	7.35630	C	-4.65400	-4.70750	-5.04190
C	-1.66490	-6.23100	7.39170	C	-5.17870	-5.99840	-4.80460
C	-1.71010	-5.40100	8.53490	C	-6.31970	-6.45490	-5.49320
C	-2.56450	-5.70040	9.61430	H	-7.82860	-5.97140	-6.96380
H	-4.04650	-7.07010	10.39000	H	-6.92520	-3.69370	-7.40570
H	-3.99140	-8.54790	8.38650	H	-4.91810	-2.89630	-6.19880
H	-2.49340	-8.01590	6.48980	H	-4.70880	-6.64640	-4.07900
H	-1.07580	-4.52860	8.59200	H	-6.70850	-7.44370	-5.29740

(*R*)-3,3’-Bis[3,5-bis(trifluoromethyl)phenyl]-1,1’-binaphthyl-2,2’-diyl hydrogenphosphate

MM (OPLS-2005) Geometry

C	0.70360	-3.24250	-0.98240	C	2.90520	-4.38230	-0.33930
C	-1.11470	-2.19980	-2.89470	C	3.35530	-4.41870	-1.68220
C	-1.49300	-3.31390	-2.12270	C	4.62610	-4.93120	-2.00490
C	-0.58500	-3.81200	-1.16050	C	5.46520	-5.42450	-0.99050
H	-1.79830	-1.80850	-3.63430	C	5.02650	-5.41490	0.34580
C	3.32260	-4.93660	2.01150	H	2.73440	-4.05520	-2.48730
C	2.03960	-4.48100	2.36980	H	4.95560	-4.94670	-3.03390
C	1.20680	-3.95250	1.35720	H	6.44030	-5.81890	-1.23800
C	1.63050	-3.85360	0.00890	H	5.67240	-5.80960	1.11700
H	3.97480	-5.34140	2.77180	P	-1.29290	-4.58440	1.23370
C	1.03640	-2.07650	-1.72890	O	-0.99260	-4.83120	-0.34360
C	0.12710	-1.57160	-2.69430	O	-0.07670	-3.60250	1.66210
C	0.45120	-0.42930	-3.45610	O	-1.47380	-5.79730	2.07340
C	1.67450	0.23410	-3.25360	O	-2.56290	-3.59100	1.21100
C	2.57370	-0.24040	-2.28290	H	-3.20280	-3.87100	1.84460
C	2.25520	-1.38290	-1.52470	C	1.57710	-4.59360	3.76620
H	-0.24000	-0.04950	-4.19480	C	-2.82430	-3.91900	-2.31110
H	1.91850	1.11130	-3.83560	C	0.98210	-3.49700	4.42720
H	3.51030	0.27250	-2.11710	H	0.86670	-2.55400	3.91140
H	2.96690	-1.71330	-0.78290	C	-5.38860	-5.07110	-2.65830
C	3.75190	-4.90620	0.67220	H	-6.36530	-5.51630	-2.78030

C	0.66220	-4.83030	6.43820
H	0.31380	-4.92910	7.45590
C	1.25380	-5.93790	5.79980
C	0.52340	-3.60500	5.75580
C	1.71130	-5.81000	4.47080
H	2.15740	-6.66440	3.98210
C	-5.26720	-3.68350	-2.44490
C	-2.96840	-5.30610	-2.53280
H	-2.09000	-5.93550	-2.56310
C	-4.24180	-5.88760	-2.71060
C	-4.38820	-7.38420	-2.93920
F	-4.69460	-7.97310	-1.78500
F	-5.36330	-7.62120	-3.81660
F	-3.25920	-7.91420	-3.41030
C	-6.50300	-2.79970	-2.37840
F	-6.25190	-1.60980	-2.92470
F	-7.52570	-3.35520	-3.02850
F	-6.84520	-2.62600	-1.10320
C	-0.12510	-2.40370	6.42560
F	0.61700	-1.31610	6.21920
F	-1.33220	-2.20810	5.89700
F	-0.25450	-2.58510	7.74010
C	1.41130	-7.24880	6.55510
F	2.47260	-7.16030	7.35510
F	0.33080	-7.48530	7.29900
F	1.58640	-8.27470	5.72210
C	-3.98640	-3.11890	-2.27150
H	-3.90190	-2.05640	-2.09130

(*R*)-3,3’-Bis[3,5-bis(*tert*-Butyl)phenyl]-1,1’-binaphthyl-2,2’-diyl hydrogenphosphate

MM (OPLS-2005) Geometry

C	-1.00210	-0.61760	0.92170
C	-3.63750	0.33070	0.45360
C	-3.24110	-0.90170	-0.09680
C	-1.92330	-1.35470	0.13740
H	-4.63990	0.69210	0.27480
C	2.90830	-2.25200	1.76460
C	1.75200	-2.99270	2.06490
C	0.49050	-2.46190	1.71600
C	0.36270	-1.17290	1.13420
H	3.87440	-2.66020	2.02460
C	-1.44990	0.59140	1.52790
C	-2.76120	1.06980	1.26810
C	-3.21070	2.27780	1.84140
C	-2.37010	3.01320	2.69580
C	-1.08040	2.53560	2.98650
C	-0.62720	1.33300	2.41200
H	-4.20720	2.64230	1.63710
H	-2.71860	3.93610	3.13670
H	-0.43570	3.09050	3.65290
H	0.36540	0.99280	2.66630
C	2.81810	-1.01740	1.09720
C	1.54860	-0.47590	0.76660
C	1.50180	0.75290	0.06270
C	2.68450	1.43670	-0.27740
C	3.93510	0.89960	0.07510
C	4.00100	-0.32730	0.75960
H	0.55830	1.18930	-0.22940
H	2.63010	2.37460	-0.81120
H	4.84330	1.42360	-0.18570
H	4.96580	-0.73990	1.01770
P	-1.41700	-3.85960	0.63600
O	-1.55590	-2.57780	-0.34510
O	-0.63090	-3.22220	1.90760
O	-0.82350	-5.07410	0.01510
O	-2.92600	-4.03480	1.18390
H	-3.55160	-3.82100	0.51010
C	1.88260	-4.29360	2.73660
C	-4.20570	-1.72740	-0.84680

C	2.13490	-4.34770	4.12140	H	-8.86610	-4.96470	0.26340
C	2.26770	-5.58410	4.79530	H	-7.80940	-5.25580	-1.10350
C	1.89870	-6.75110	2.64780	C	-8.04600	-2.48950	0.97060
C	1.77090	-5.49510	2.01040	H	-7.32640	-2.80300	1.72810
H	1.57830	-5.42350	0.95120	H	-8.00260	-1.40280	0.88860
C	-3.86790	-2.28530	-2.09580	H	-9.03600	-2.73840	1.35450
H	-2.89650	-2.06730	-2.51480	C	-8.87920	-2.70880	-1.36590
C	-4.75410	-3.13390	-2.79310	H	-9.87660	-2.90440	-0.97070
C	-5.47380	-2.01060	-0.29960	H	-8.80580	-1.63700	-1.55410
C	-6.39640	-2.84650	-0.97100	H	-8.81530	-3.21280	-2.33000
H	2.21620	-3.41050	4.64850	C	1.38030	-6.43480	6.99030
H	-5.71210	-1.58050	0.66060	H	1.28580	-7.45610	6.62190
C	1.76930	-8.07990	1.88130	H	1.52430	-6.49650	8.06930
C	-6.01760	-3.40080	-2.21530	H	0.42590	-5.93700	6.81410
H	-6.72120	-4.04300	-2.71930	C	2.64760	-4.28980	6.98400
C	-7.78320	-3.17610	-0.38760	H	2.83800	-4.38530	8.05350
C	-4.31220	-3.74310	-4.13600	H	3.46720	-3.70360	6.56670
C	2.14410	-6.77200	4.03950	H	1.72810	-3.71320	6.87610
H	2.23600	-7.72310	4.53960	C	3.85890	-6.42400	6.55470
C	2.53470	-5.67400	6.30870	H	4.09170	-6.48480	7.61820
C	-5.38810	-4.65080	-4.77080	H	3.83050	-7.44540	6.17560
H	-5.63460	-5.49540	-4.12620	H	4.69190	-5.91870	6.06440
H	-6.30730	-4.10190	-4.97900	C	1.54670	-7.88450	0.36590
H	-5.04560	-5.06650	-5.71910	H	0.62280	-7.34180	0.16160
C	-3.04740	-4.59800	-3.91800	H	2.36830	-7.33610	-0.09580
H	-3.23030	-5.39500	-3.19600	H	1.46980	-8.84090	-0.15170
H	-2.71740	-5.06700	-4.84550	C	0.56800	-8.87160	2.43310
H	-2.20900	-4.01060	-3.54370	H	-0.35480	-8.29880	2.33020
C	-4.00170	-2.61380	-5.13810	H	0.43080	-9.81270	1.90000
H	-3.70790	-3.01260	-6.10950	H	0.68470	-9.11360	3.48930
H	-4.87380	-1.97780	-5.29480	C	3.05720	-8.90930	2.05850
H	-3.18840	-1.97290	-4.79840	H	3.00730	-9.84570	1.50220
C	-7.90260	-4.69780	-0.17200	H	3.92800	-8.36000	1.69850
H	-7.12680	-5.06100	0.50330	H	3.24310	-9.16800	3.10040

(*R*)-3,3'-Bis[3,5-bis(*tert*-Butyl)-4-methoxyphenyl]-1,1'-binaphthyl-2,2'-diyl hydrogenphosphate

MM (OPLS-2005) Geometry

C	0.17240	-7.01150	3.16550
C	0.85060	-9.09250	1.35610
C	-0.39740	-8.45450	1.23520
C	-0.72190	-7.42520	2.14780
H	1.10840	-9.88580	0.66950
C	-0.84460	-3.69960	5.74770
C	-0.87000	-3.53980	4.35110
C	-0.60480	-4.65910	3.53000
C	-0.24260	-5.91570	4.08410
H	-1.05550	-2.84710	6.37720
C	1.45810	-7.62290	3.22120
C	1.77900	-8.67870	2.32780
C	3.03900	-9.31040	2.38680
C	4.00170	-8.88650	3.32000
C	3.70880	-7.82220	4.19030
C	2.45050	-7.19320	4.13710
H	3.27830	-10.11750	1.70940
H	4.96750	-9.36940	3.36020
H	4.45000	-7.48460	4.90030
H	2.26560	-6.37310	4.81450
C	-0.59340	-4.95760	6.32320
C	-0.30350	-6.07480	5.49780
C	-0.08960	-7.33210	6.11520
C	-0.13610	-7.46720	7.51580
C	-0.40370	-6.34760	8.32300
C	-0.63420	-5.09490	7.72640
H	0.11140	-8.21340	5.52460
H	0.03300	-8.43290	7.97000
H	-0.43980	-6.45030	9.39800
H	-0.84990	-4.24090	8.35230
P	-1.94860	-5.26130	1.38020
O	-1.90380	-6.76060	1.99250
O	-0.72660	-4.53770	2.17200
O	-3.27670	-4.59320	1.43010
O	-1.34790	-5.47860	-0.10280
H	-1.68900	-6.27000	-0.48720
C	-1.16560	-2.21590	3.78300
C	-1.31370	-8.81620	0.13740
C	-3.58520	-9.35010	-0.66790

C	-0.46220	0.10460	3.33180
C	-0.22460	-1.18050	3.87380
C	-2.67630	-9.05520	0.37260
C	-2.70140	-0.69830	2.59190
C	-0.85110	-8.86440	-1.18670
C	-1.69920	-9.16800	-2.27620
C	-1.68310	0.30740	2.61220
C	-3.06170	-9.50160	-1.99100
C	-2.39720	-1.95300	3.16900
C	-1.06420	-9.10100	-3.69520
C	-0.06750	-10.26460	-3.86120
H	0.73050	-10.22560	-3.11960
H	-0.56230	-11.22940	-3.75370
H	0.40460	-10.24810	-4.84410
C	-2.06050	-9.14700	-4.87680
H	-2.52880	-10.12130	-4.99520
H	-2.84640	-8.39860	-4.76730
H	-1.56270	-8.95100	-5.82690
C	-0.30090	-7.76400	-3.86930
H	0.10200	-7.66140	-4.87750
H	-0.96060	-6.91230	-3.69810
H	0.55080	-7.65890	-3.19950
C	-5.08410	-9.46700	-0.26890
C	-5.29080	-10.73970	0.57550
H	-5.01680	-11.63530	0.01890
H	-4.68910	-10.72670	1.48440
H	-6.33190	-10.85060	0.88030
C	-5.49810	-8.23610	0.57690
H	-4.97750	-8.16760	1.53070
H	-5.30950	-7.30720	0.03650
H	-6.56080	-8.25590	0.82100
C	-6.08730	-9.49880	-1.44480
H	-7.11710	-9.43430	-1.09220
H	-5.93020	-8.66260	-2.12730
H	-6.03740	-10.42250	-2.01660
C	0.62970	1.19610	3.53070
C	0.07440	2.64060	3.57050
H	-0.25060	3.00400	2.59920
H	-0.76600	2.72180	4.26090

H	0.83160	3.35430	3.89560
C	1.37090	1.02210	4.88190
H	0.66830	0.98380	5.71550
H	1.99070	0.12760	4.91930
H	2.05380	1.85020	5.07440
C	1.67400	1.07820	2.40570
H	2.46750	1.81750	2.51750
H	2.14070	0.09260	2.40180
H	1.22880	1.22510	1.42380
C	-4.10750	-0.51060	1.95030
C	-4.06150	-0.97810	0.48410
H	-3.34820	-0.40640	-0.10470
H	-3.76680	-2.02640	0.41620
H	-5.03480	-0.88140	0.00280
C	-5.18740	-1.35760	2.67270
H	-5.05970	-2.42910	2.51930
H	-5.19120	-1.16480	3.74610
H	-6.18780	-1.13560	2.30050
C	-4.63540	0.94310	2.01600
H	-5.68540	1.00230	1.72920
H	-4.55060	1.34830	3.02490
H	-4.12280	1.61870	1.33620
C	-1.40430	1.41500	0.56570
H	-2.22330	1.63540	-0.11790
H	-0.64070	2.17460	0.40350
H	-0.98290	0.44640	0.28850
C	-3.80210	-11.42750	-3.05580
H	-2.79280	-11.77990	-3.26720
H	-4.13410	-11.89370	-2.12870
H	-4.45320	-11.77530	-3.85750
O	-1.86690	1.46810	1.91050
O	-3.85750	-10.00750	-2.98040
H	0.70360	-1.41530	4.36620
H	-3.02470	-8.99820	1.39200
H	0.19250	-8.65290	-1.35850
H	-3.10500	-2.76340	3.14250

(*R*)-3,3'-Bis(2,4,6-triisopropylphenyl)-1,1'-binaphthyl-2,2'-diyl hydrogenphosphate

MM (OPLS-2005) Geometry

C	-0.90720	-0.83440	0.76310
C	-3.57370	0.08410	0.43960
C	-3.22870	-1.20130	-0.01280
C	-1.89400	-1.63830	0.13990
H	-4.58260	0.43940	0.29450
C	3.11530	-2.28910	1.35080
C	2.01940	-3.05390	1.78830
C	0.71400	-2.59000	1.50910
C	0.48750	-1.34630	0.86500
H	4.11450	-2.63110	1.57540
C	-1.30990	0.41700	1.31340
C	-2.63770	0.88050	1.12130
C	-3.04170	2.13360	1.62730
C	-2.13720	2.93020	2.35160
C	-0.82830	2.47010	2.57780
C	-0.42060	1.22330	2.06700
H	-4.05150	2.48570	1.47250
H	-2.45050	3.88760	2.74260
H	-0.13330	3.07330	3.14410
H	0.59010	0.90020	2.26720
C	2.92220	-1.11480	0.60270
C	1.60980	-0.64320	0.34230
C	1.45680	0.52620	-0.44300
C	2.57960	1.22130	-0.93110
C	3.87520	0.75550	-0.64600
C	4.04550	-0.41240	0.11900
H	0.47580	0.90740	-0.68490
H	2.44460	2.11350	-1.52540

H	4.73760	1.28880	-1.01930
H	5.04360	-0.77080	0.32580
P	-1.22720	-4.10380	0.69020
O	-1.56100	-2.88040	-0.31650
O	-0.35760	-3.36710	1.84670
O	-0.62450	-5.30380	0.05310
O	-2.65140	-4.34700	1.40440
H	-3.35620	-4.13770	0.81150
C	2.70870	-5.48390	1.90260
C	2.25760	-4.29660	2.55270
C	2.06820	-4.31310	3.96580
C	2.31890	-5.49510	4.69470
H	2.16220	-5.50850	5.76230
C	-5.27410	-2.66980	0.18570
C	-4.25390	-2.07700	-0.62040
C	-4.23780	-2.34620	-2.02060
C	-5.20900	-3.20130	-2.58340
H	-5.18850	-3.40570	-3.64350
C	2.92360	-5.54800	0.38440
H	2.38400	-4.72580	-0.08440
C	2.34670	-6.82690	-0.25080
H	1.30620	-6.97470	0.03740
H	2.37490	-6.76510	-1.33870
H	2.89790	-7.72180	0.03440
C	4.41170	-5.40190	0.03120
H	5.00020	-6.21580	0.45540
H	4.82270	-4.46820	0.41080
H	4.56110	-5.41040	-1.04860
C	1.59880	-3.07950	4.74650
H	1.52120	-2.22050	4.08390
C	0.19790	-3.29680	5.33870
H	-0.52120	-3.53450	4.55400
H	0.18440	-4.11640	6.05700
H	-0.15740	-2.40160	5.84900
C	2.61290	-2.66840	5.82690
H	2.73180	-3.43440	6.59270
H	3.59330	-2.48470	5.38620
H	2.30090	-1.75140	6.32700
C	-5.38580	-2.41000	1.69630
H	-4.43920	-2.01750	2.06590
C	-5.65050	-3.68730	2.51810
H	-4.92350	-4.46940	2.30700
H	-5.59110	-3.47520	3.58600
H	-6.64070	-4.10290	2.33470
C	-6.47480	-1.36630	1.99060
H	-7.45730	-1.71930	1.67580
H	-6.28550	-0.42880	1.47070
H	-6.52840	-1.14320	3.05640
C	-3.20370	-1.72460	-2.96760
H	-2.52580	-1.07790	-2.41530
C	-3.87420	-0.81940	-4.01330
H	-4.54640	-1.37620	-4.66600
H	-4.45640	-0.03450	-3.52950
H	-3.13050	-0.33380	-4.64550
C	-2.32730	-2.79890	-3.63230
H	-2.90990	-3.46790	-4.26530
H	-1.55650	-2.34530	-4.25560
H	-1.82220	-3.41090	-2.88450
C	2.78050	-6.65900	4.05710
C	3.06030	-7.92460	4.85560
H	3.40730	-8.68790	4.15750
C	1.78330	-8.46500	5.51600
H	1.37430	-7.76120	6.24100
H	1.01190	-8.65550	4.76890
H	1.97610	-9.40300	6.03690
C	4.18300	-7.70620	5.88080
H	3.90940	-6.95990	6.62670
H	4.41890	-8.63050	6.40840
H	5.09510	-7.36490	5.39010
C	-6.21270	-3.78220	-1.79190
C	-7.25650	-4.69280	-2.42410
H	-7.03020	-4.76930	-3.48880
C	-7.18660	-6.11420	-1.84610
H	-6.19110	-6.53930	-1.97910
H	-7.41330	-6.12980	-0.78000
H	-7.89530	-6.77630	-2.34400
C	-8.66760	-4.09830	-2.30440
H	-8.97790	-3.99880	-1.26420
H	-8.71170	-3.10820	-2.75940
H	-9.40240	-4.72540	-2.80980

C	-6.24280	-3.50280	-0.41530	C	2.97920	-6.64080	2.66650
H	-7.03140	-3.93450	0.18110	H	3.34030	-7.53340	2.18030

Transfer Hydrogenation Study

TS1

(B3LYP/6-31G**:UFF) Energy = -1966.231653

(B3LYP/6-31G**:UFF) Free Energy = -1965.344785

M06-2X/6-31G** Derived free energy = -3194.529235

M06-2X/6-31G** Derived free energy in solution = -3194.547535

Number of Imaginary Frequencies = 1 (-561.47)

ONIOM (B3LYP/6-31G**:UFF) Geometry

C	3.90480	0.08280	-0.26550	C	4.85390	-3.65760	3.32750
C	4.91740	2.70930	-0.57060	C	3.98410	-4.17950	2.45610
C	3.47130	2.50760	-0.33530	H	5.15290	-0.47600	2.10400
C	2.97050	1.25460	-0.24970	H	5.97960	-1.83330	3.90650
H	5.30530	3.71790	-0.65840	H	5.23060	-4.25840	4.14520
C	2.46820	-3.92970	0.41060	H	3.65850	-5.20560	2.57380
C	1.92740	-3.18080	-0.56760	O	1.63650	1.07310	0.03210
C	2.37230	-1.76560	-0.72290	O	1.79460	-0.96680	-1.67380
C	3.36670	-1.27110	0.04020	P	0.76030	0.14420	-1.03530
H	2.18200	-4.96770	0.53090	O	0.30120	0.95730	-2.22320
C	5.73740	1.66090	-0.70110	O	-0.26890	-0.54030	-0.16030
C	5.21430	0.27890	-0.55800	H	-1.50560	-0.56060	0.89210
C	6.18000	-0.83540	-0.74780	C	-1.04340	-4.80660	-3.25430
C	7.47210	-0.58510	-0.98990	C	0.91630	-3.76340	-1.49190
C	7.98790	0.79660	-1.08810	H	-1.79800	-5.19260	-3.92720
C	7.17880	1.85300	-0.95810	C	1.04790	6.03440	0.13290
H	5.85450	-1.86670	-0.71240	C	2.61910	3.70500	-0.17540
H	8.15830	-1.41120	-1.12630	H	0.44610	6.92570	0.25460
H	9.04230	0.95200	-1.27750	C	-3.95920	2.15440	-0.98900
H	7.59080	2.85160	-1.04300	C	-2.87000	2.13900	-1.82760
C	3.46560	-3.35750	1.33790	C	-2.90320	-0.28980	-1.92870
C	3.89060	-2.08350	1.17870	C	-4.00180	-0.33910	-1.09140
C	4.83520	-1.50760	2.17500	N	-2.34270	0.92660	-2.20080
C	5.29290	-2.25660	3.18480	H	-1.32590	0.92530	-2.47300

C	-4.40390	0.88740	-0.38140	C	-4.38490	-3.29560	3.01190
H	-3.86350	0.82830	0.73410	C	-4.77240	-0.79690	3.01370
H	-5.44280	0.88620	-0.05220	H	-5.58470	-0.72010	2.28200
C	-2.14100	3.31470	-2.41530	H	-5.23630	-0.93890	3.99430
H	-2.54000	3.54560	-3.40960	C	-3.92910	0.48480	3.02580
H	-2.25530	4.20440	-1.80160	H	-3.34010	0.53480	3.95440
H	-1.08330	3.06150	-2.52380	H	-4.56460	1.37370	3.00250
C	-2.22810	-1.44470	-2.61570	C	-2.06990	1.79830	1.76150
H	-2.93690	-1.99460	-3.23750	H	-2.68760	2.67220	1.99240
H	-1.40250	-1.08560	-3.23120	C	-0.82380	1.74480	2.67360
H	-1.82710	-2.13680	-1.87520	C	-0.00250	3.03700	2.62160
C	-4.79860	-1.53720	-0.77180	H	-0.60990	3.87770	2.98340
C	-4.63300	3.35770	-0.46520	H	0.24750	3.25310	1.57720
C	-5.13060	-3.86110	-1.03610	C	1.28720	2.93220	3.44050
H	-5.12440	-4.06560	0.03670	H	1.95160	2.17340	3.01400
H	-6.16340	-3.73240	-1.36680	H	1.07900	2.65220	4.47950
H	-4.64980	-4.67130	-1.58400	H	1.82880	3.88290	3.45940
C	-5.14240	5.64410	-0.72100	C	-0.07120	-4.55760	-1.03050
H	-4.93740	6.42710	-1.45050	H	-0.14940	-4.79460	0.02250
H	-6.21800	5.48180	-0.62200	C	-1.09710	-5.09260	-1.94930
H	-4.73540	5.91250	0.25680	H	-1.89820	-5.70810	-1.56080
O	-5.77930	-1.50890	-0.04350	C	0.97930	-3.47580	-2.95020
O	-5.27250	3.35190	0.57560	H	1.78940	-2.88460	-3.35710
O	-4.50050	4.46340	-1.23360	C	0.04980	-3.96600	-3.77980
O	-4.35360	-2.68530	-1.33740	H	0.10160	-3.75340	-4.83970
C	-3.91770	-2.00010	2.65820	C	2.94200	4.69270	0.88400
C	-2.78010	-1.86860	1.92570	H	3.77790	4.51970	1.55040
N	-2.33780	-0.60620	1.53060	C	2.19160	5.79110	1.03440
C	-2.92920	0.55800	1.88930	H	2.42230	6.50390	1.81550
H	-1.73010	1.90310	0.72630	C	1.58780	3.94310	-1.00500
C	-1.93540	-3.00510	1.43200	H	1.36450	3.26500	-1.81610
H	-2.20680	-3.23430	0.39550	C	0.76520	5.16010	-0.83950
H	-2.09210	-3.90190	2.03210	H	-0.06070	5.34770	-1.51180
H	-0.87720	-2.73200	1.43970	H	-1.12430	1.54320	3.71060
N	-4.79340	-4.34880	3.30330	H	-0.19430	0.91040	2.34810

(M06-2X/6-31G**:UFF) Energy = -1965.527272

(M06-2X /6-31G**:UFF) Free Energy = -1964.630076

M06-2X/6-31G** Derived free energy = -3194.525539

M06-2X/6-31G** Derived free energy in solution = -3194.543357

Number of Imaginary Frequencies = 1 (-695.33)

ONIOM (M06-2X /6-31G**:UFF) Geometry

C	3.77000	0.90820	-0.11110	C	0.54850	-5.23180	-3.12780
C	4.11730	3.66470	-0.67610	C	1.94920	-3.62090	-1.26230
C	2.75790	3.12510	-0.47010	H	0.00580	-5.83810	-3.84130
C	2.58300	1.80780	-0.23930	C	-0.53730	5.87800	-0.65830
H	4.24440	4.72400	-0.86850	C	1.61460	4.05400	-0.53290
C	3.31760	-3.29370	0.75780	H	-1.36530	6.57360	-0.70030
C	2.67150	-2.75310	-0.29130	C	-4.23360	0.62100	-0.95250
C	2.73430	-1.27680	-0.49490	C	-3.15940	0.93990	-1.74790
C	3.54860	-0.51570	0.26620	C	-2.49680	-1.37610	-1.87260
H	3.30950	-4.36520	0.91720	C	-3.57110	-1.76180	-1.11070
C	5.17920	2.85030	-0.65060	N	-2.32080	-0.05470	-2.15290
C	5.00630	1.39950	-0.37600	H	-1.36720	0.24380	-2.46530
C	6.22500	0.55000	-0.42580	C	-4.38660	-0.74450	-0.43340
C	7.42650	1.09320	-0.65520	H	-3.97770	-0.72300	0.75000
C	7.59080	2.54530	-0.87750	H	-5.41780	-1.02510	-0.21900
C	6.54070	3.37280	-0.88100	C	-2.79280	2.29230	-2.29510
H	6.16260	-0.52230	-0.29690	H	-3.43830	2.53250	-3.14480
H	8.30120	0.45630	-0.68870	H	-2.93660	3.07260	-1.55130
H	8.58300	2.94160	-1.05080	H	-1.75140	2.27550	-2.62480
H	6.69800	4.43040	-1.05710	C	-1.45420	-2.28670	-2.44430
C	4.07370	-2.44330	1.69990	H	-0.95710	-2.81320	-1.62810
C	4.18740	-1.11430	1.47600	H	-1.90670	-3.04120	-3.08910
C	4.90190	-0.27210	2.47390	H	-0.71280	-1.70680	-2.99700
C	5.46110	-0.83060	3.55360	C	-3.77950	-3.18060	-0.78470
C	5.36310	-2.28640	3.77070	C	-5.26570	1.57410	-0.49810
C	4.70240	-3.05170	2.89540	C	-5.05150	-4.68170	0.48120
H	4.96770	0.80010	2.34620	H	-4.19450	-5.05910	1.04630
H	5.97970	-0.21390	4.27640	H	-5.20980	-5.32670	-0.38490
H	5.82030	-2.73150	4.64490	H	-5.94070	-4.65180	1.10860
H	4.62690	-4.11830	3.06660	C	-6.12650	3.74940	-0.51830
O	1.32810	1.32650	0.03190	H	-5.92740	4.67320	-1.05790
O	2.01320	-0.69890	-1.50640	H	-7.13160	3.38130	-0.73110
P	0.74100	0.18400	-0.99390	H	-6.01160	3.90700	0.55570
O	0.17200	0.81360	-2.23290	O	-3.11620	-4.12120	-1.16780
O	-0.16920	-0.64270	-0.11170	O	-6.15850	1.25290	0.26190
H	-1.32560	0.11510	0.72030	O	-5.14810	2.81430	-0.98720

O	-4.81930	-3.33670	0.06840	H	0.25800	-3.03100	1.49280
C	-3.51970	2.12570	2.22970	C	-1.03560	-4.61190	2.21220
C	-2.43390	1.75070	1.51340	H	-1.63770	-4.58790	1.29760
N	-2.15110	0.40160	1.32110	H	-1.70430	-4.80950	3.05830
C	-2.91890	-0.58690	1.81160	H	-0.35150	-5.45950	2.12830
H	-3.08890	-2.70960	1.87930	C	1.19480	-4.65850	-0.84620
C	-1.43340	2.66880	0.88990	H	1.09860	-4.89150	0.20420
H	-1.78670	3.69950	0.89720	C	0.45790	-5.49080	-1.81920
H	-1.21570	2.35790	-0.13690	H	-0.16140	-6.30620	-1.46850
H	-0.48470	2.60510	1.43130	C	2.06240	-3.36170	-2.72330
N	-4.16250	4.62730	2.38870	H	2.70280	-2.57070	-3.09130
C	-3.85010	3.51150	2.32170	C	1.39620	-4.12100	-3.60180
C	-4.45090	1.13080	2.89340	H	1.48460	-3.92790	-4.66300
H	-4.70340	1.48600	3.89680	C	1.41320	5.04490	0.55080
H	-5.38400	1.06850	2.32710	H	2.08960	5.06930	1.39610
C	-3.78710	-0.24280	2.99760	C	0.38970	5.90660	0.49210
H	-4.52530	-1.03330	3.15160	H	0.23880	6.62590	1.28660
H	-3.10380	-0.26240	3.85950	C	0.77580	4.04010	-1.58330
C	-2.30860	-1.95670	1.73370	H	0.92430	3.34520	-2.39960
H	-1.87000	-2.08860	0.73820	C	-0.35280	4.99230	-1.64470
C	-1.18120	-2.12480	2.77000	H	-1.02980	4.97050	-2.48880
C	-0.27500	-3.30110	2.41050	H	-0.58230	-1.20810	2.79300
H	0.47910	-3.42050	3.19610	H	-1.61340	-2.26780	3.76870

TS2

(B3LYP/6-31G**:UFF) Energy = -1966.227512

(B3LYP/6-31G**:UFF) Free Energy = -1965.339854

M06-2X/6-31G** Derived free energy = -3194.523722

M06-2X/6-31G** Derived free energy in solution = -3194.541356

Number of Imaginary Frequencies = 1 (-651.01)

ONIOM (B3LYP/6-31G**:UFF) Geometry

C	3.80630	0.73610	-0.21190	C	2.48180	-2.85680	-0.25840
C	4.34970	3.45040	-0.82810	C	2.66170	-1.40350	-0.53860
C	2.95150	3.00470	-0.65740	C	3.48870	-0.65480	0.21940
C	2.68360	1.70560	-0.41140	H	2.95260	-4.43910	1.06010
H	4.55210	4.49470	-1.03700	C	5.35500	2.57040	-0.74750
C	3.04820	-3.38200	0.84290	C	5.08000	1.14170	-0.44220

C	6.24410	0.21790	-0.41250	H	-3.32630	2.58160	-3.27500
C	7.48570	0.67830	-0.60560	H	-2.85530	3.14740	-1.68270
C	7.74980	2.10940	-0.86480	H	-1.67050	2.27930	-2.70020
C	6.75500	2.99970	-0.93680	C	-1.75840	-2.32910	-2.69250
H	6.10900	-0.84360	-0.25240	H	-1.42280	-3.10440	-2.00620
H	8.31940	-0.01190	-0.58080	H	-2.30850	-2.83680	-3.49000
H	8.77130	2.43790	-1.00730	H	-0.89550	-1.81420	-3.11820
H	6.98580	4.03950	-1.13630	C	-4.08420	-3.06680	-0.91250
C	3.82310	-2.53360	1.77140	C	-5.21450	1.75040	-0.54070
C	4.03710	-1.22890	1.48460	C	-5.48030	-4.50360	0.33730
C	4.76300	-0.38260	2.47070	H	-4.65960	-5.15470	0.64820
C	5.23340	-0.91670	3.60380	H	-5.93850	-4.92480	-0.56070
C	5.02540	-2.34850	3.89150	H	-6.21770	-4.40870	1.13440
C	4.35380	-3.11550	3.02610	C	-6.06240	3.94860	-0.68120
H	4.90770	0.67440	2.29170	H	-5.90850	4.81280	-1.32660
H	5.75980	-0.29700	4.31820	H	-7.08730	3.58170	-0.76210
H	5.41020	-2.77400	4.80930	H	-5.85330	4.20980	0.35910
H	4.19600	-4.16330	3.24930	O	-3.66530	-4.04410	-1.50890
O	1.38930	1.32470	-0.16170	O	-6.06910	1.49940	0.29780
O	2.00450	-0.82890	-1.59410	O	-5.12970	2.95390	-1.14630
P	0.75250	0.13000	-1.12430	O	-5.00740	-3.17120	0.08710
O	0.19200	0.68780	-2.41140	C	-3.26430	2.35320	2.51060
O	-0.17990	-0.59480	-0.17040	C	-2.18870	1.95720	1.77700
H	-1.21040	0.31590	0.85140	N	-1.99940	0.60500	1.48070
C	0.30280	-5.35280	-3.03630	C	-2.81510	-0.39380	1.90310
C	1.72840	-3.72200	-1.20740	H	-3.02390	-2.51670	1.94470
H	-0.24610	-5.96870	-3.73680	C	-1.13620	2.87140	1.22530
C	-0.20340	5.92200	-0.85060	H	-1.31160	3.90190	1.53400
C	1.87130	4.00830	-0.73920	H	-1.12430	2.82620	0.13210
H	-1.00100	6.65270	-0.88690	H	-0.13990	2.55700	1.54920
C	-4.20860	0.76260	-0.97590	N	-3.74480	4.85710	2.95710
C	-3.14590	1.01430	-1.82080	C	-3.49800	3.73520	2.75270
C	-2.64860	-1.35270	-1.98120	C	-4.30650	1.36730	3.01290
C	-3.69580	-1.66580	-1.13940	H	-4.63300	1.65490	4.01740
N	-2.37450	-0.03680	-2.23350	H	-5.18800	1.41380	2.36630
H	-1.40710	0.19970	-2.56180	C	-3.74250	-0.05790	3.05230
C	-4.35130	-0.58160	-0.38390	H	-4.54530	-0.79820	3.10430
H	-3.79800	-0.53300	0.73670	H	-3.13640	-0.18840	3.96120
H	-5.36470	-0.80060	-0.05160	C	-2.21920	-1.78250	1.85340
C	-2.71830	2.33810	-2.39710	H	-1.72870	-1.92030	0.88560

C	-1.16130	-1.97820	2.97090	H	2.63270	-2.84320	-3.05770
C	-0.24880	-3.18780	2.73240	C	1.24530	-4.33320	-3.53570
H	0.50910	-3.20690	3.52590	H	1.39480	-4.21620	-4.60120
H	0.28950	-3.01670	1.79430	C	1.80020	5.09210	0.27000
C	-0.97220	-4.53730	2.69110	H	2.54030	5.14860	1.05850
H	-1.65650	-4.60650	1.83880	C	0.81240	5.99480	0.21970
H	-1.55560	-4.70340	3.60460	H	0.75580	6.78020	0.96220
H	-0.25890	-5.36260	2.60160	C	0.95220	3.95590	-1.71980
C	0.87630	-4.66940	-0.76530	H	1.00000	3.18890	-2.48130
H	0.71160	-4.82270	0.29110	C	-0.13340	4.95710	-1.77520
C	0.13140	-5.51580	-1.72020	H	-0.87340	4.90530	-2.56310
H	-0.55960	-6.26240	-1.35090	H	-0.53560	-1.08040	3.01820
C	1.92310	-3.56480	-2.67400	H	-1.66120	-2.07410	3.94390

(M06-2X/6-31G**:UFF) Energy = -1965.524290

(M06-2X /6-31G**:UFF) Free Energy = -1964.627019

M06-2X/6-31G** Derived free energy = -3194.524425

M06-2X/6-31G** Derived free energy in solution = -3194.540870

Number of Imaginary Frequencies = 1 (-931.73)

ONIOM (M06-2X /6-31G**:UFF) Geometry

C	3.75400	-0.14800	-0.15120	H	5.55280	-2.28270	-0.30870
C	5.04170	2.36000	-0.42160	H	7.92050	-2.05400	-0.44830
C	3.56550	2.30110	-0.36410	H	9.03650	0.21150	-0.54820
C	2.93920	1.10460	-0.28690	H	7.75360	2.24220	-0.54740
H	5.53160	3.32400	-0.49980	C	2.81720	-3.51800	1.38450
C	1.90830	-4.01580	0.33110	C	3.36080	-2.28380	1.28670
C	1.55390	-3.22940	-0.70260	C	4.20530	-1.77480	2.40160
C	2.12590	-1.85340	-0.78790	C	4.46040	-2.54770	3.46340
C	3.05820	-1.44120	0.09260	C	3.89570	-3.90830	3.54340
H	1.53300	-5.02900	0.40730	C	3.11310	-4.36820	2.56120
C	5.76820	1.23820	-0.40460	H	4.61090	-0.77210	2.37410
C	5.10270	-0.08310	-0.28350	H	5.07460	-2.17320	4.27220
C	5.97490	-1.28690	-0.32720	H	4.10900	-4.52920	4.40390
C	7.30460	-1.16440	-0.41630	H	2.69350	-5.36400	2.63240
C	7.95710	0.15980	-0.48580	O	1.57220	1.06390	-0.14180
C	7.24180	1.28900	-0.48580	O	1.71470	-0.99860	-1.77500

P	0.70990	0.16170	-1.22230
O	0.33410	0.97390	-2.42690
O	-0.38220	-0.46080	-0.38980
H	-1.50300	-0.57850	0.71240
C	-1.20160	-4.58020	-3.75160
C	0.62200	-3.73030	-1.75280
H	-1.90320	-4.89150	-4.51450
C	1.47300	6.06270	-0.40910
C	2.82150	3.57860	-0.37740
H	0.95850	7.01480	-0.42000
C	-3.68630	2.44560	-0.76070
C	-2.65570	2.38400	-1.65620
C	-2.90340	-0.01360	-1.87440
C	-3.95080	-0.01580	-0.98190
N	-2.28080	1.16480	-2.15010
H	-1.30760	1.10170	-2.53500
C	-4.24580	1.20160	-0.21800
H	-3.70170	1.05700	0.87590
H	-5.27510	1.27450	0.13870
C	-1.82910	3.52660	-2.17030
H	-2.34960	4.03090	-2.98900
H	-1.65910	4.26450	-1.38840
H	-0.87350	3.14210	-2.53270
C	-2.34840	-1.18930	-2.62620
H	-3.11160	-1.62760	-3.27170
H	-1.49100	-0.87460	-3.22320
H	-2.01940	-1.95350	-1.92390
C	-4.78540	-1.17830	-0.63990
C	-4.12940	3.66470	-0.06530
C	-5.19020	-3.47390	-0.84830
H	-5.14140	-3.64120	0.23070
H	-6.23300	-3.32750	-1.13540
H	-4.75450	-4.31320	-1.38830
C	-4.28360	5.99350	-0.02570
H	-3.99100	6.82480	-0.66430
H	-5.36350	5.99050	0.13390
H	-3.78540	6.05590	0.94400
O	-5.73050	-1.11180	0.12050
O	-4.67440	3.62990	1.02060
O	-3.88220	4.81090	-0.71900
O	-4.40790	-2.33360	-1.21750
C	-3.98490	-1.80170	2.54190
C	-2.88380	-1.78610	1.76030
N	-2.29300	-0.57490	1.40870
C	-2.69920	0.61810	1.87310
H	-1.45160	1.83350	0.63740
C	-2.21280	-2.97880	1.16030
H	-2.39710	-2.97850	0.08090
H	-2.59490	-3.90650	1.58580
H	-1.13200	-2.91690	1.31460
N	-5.13340	-4.06300	3.05690
C	-4.61310	-3.05050	2.83320
C	-4.63630	-0.52430	3.02420
H	-5.45700	-0.27700	2.34080
H	-5.06980	-0.67550	4.01580
C	-3.60980	0.60820	3.07750
H	-2.95620	0.48310	3.95370
H	-4.09590	1.58330	3.16050
C	-1.70840	1.73850	1.69950
H	-2.17160	2.67610	2.02560
C	-0.41210	1.44460	2.47280
C	0.54110	2.63370	2.47600
H	0.07820	3.47980	2.99930
H	0.70970	2.94460	1.44020
C	1.87050	2.27290	3.13170
H	2.36090	1.47030	2.57290
H	1.71950	1.92030	4.15710
H	2.54960	3.12740	3.17160
C	-0.45040	-4.48380	-1.43260
H	-0.65390	-4.75930	-0.40690
C	-1.40440	-4.91730	-2.47410
H	-2.27440	-5.49860	-2.19740
C	0.85500	-3.39640	-3.18460
H	1.73990	-2.85010	-3.48350
C	-0.01190	-3.79170	-4.12500
H	0.16450	-3.54550	-5.16400
C	3.11370	4.60460	0.65470
H	3.84770	4.40520	1.42540
C	2.46480	5.77520	0.64600
H	2.67310	6.51760	1.40550

C	1.92780	3.85360	-1.34380	H	0.51760	5.37450	-2.14730
H	1.73540	3.14350	-2.13620	H	-0.64340	1.15360	3.50650
C	1.22580	5.15430	-1.35960	H	0.08280	0.59530	1.98830

TS3

(B3LYP/6-31G**:UFF) Energy = -1966.178134

(B3LYP/6-31G**:UFF) Free Energy = -1965.096055

M06-2X/6-31G** Derived free energy = -3808.641262

M06-2X/6-31G** Derived free energy in solution = -3808.659631

Number of Imaginary Frequencies = 1 (-718.29)

ONIOM (B3LYP/6-31G**:UFF) Geometry

C	-3.92690	0.60110	0.04250	H	-5.75550	0.58020	-1.97290
C	-4.24410	3.29070	0.86620	H	-7.17560	-0.35420	-3.67030
C	-2.97950	2.85990	0.23170	H	-6.93760	-2.79920	-4.35170
C	-2.81140	1.57030	-0.12030	H	-5.26010	-4.19330	-3.31140
H	-4.36440	4.32720	1.15990	O	-1.63700	1.15140	-0.69440
C	-3.43420	-3.44640	-1.35510	O	-1.81750	-0.99280	0.87580
C	-2.57850	-2.93710	-0.45630	P	-0.75510	0.12140	0.26460
C	-2.69270	-1.51000	-0.05060	O	-0.22550	0.88740	1.45720
C	-3.73250	-0.76850	-0.49490	O	0.21380	-0.54350	-0.68750
H	-3.36130	-4.48810	-1.64660	H	1.77570	-0.95630	-0.78150
C	-5.22110	2.40330	1.09520	C	0.28420	-5.67890	1.26240
C	-5.05930	0.98550	0.67810	C	-1.56760	-3.82540	0.16190
C	-6.16610	0.04910	0.99910	H	0.99800	-6.37970	1.67950
C	-7.27600	0.48950	1.60350	C	0.15790	5.75530	-0.42610
C	-7.44190	1.90980	1.98060	C	-1.90630	3.85590	0.01920
C	-6.48090	2.81040	1.74800	H	0.94660	6.47740	-0.60300
H	-6.08860	-1.00460	0.76510	C	3.78400	1.64550	-0.77680
H	-8.07150	-0.20890	1.83050	C	2.49920	2.05620	-0.47210
H	-8.35990	2.22170	2.46190	C	2.95320	1.86080	1.90770
H	-6.63470	3.84120	2.04440	C	4.23980	1.44600	1.65500
C	-4.48210	-2.60000	-1.95680	N	2.10910	2.05820	0.84030
C	-4.62760	-1.31540	-1.55990	H	1.09120	1.93840	1.04220
C	-5.64730	-0.46420	-2.23280	C	4.57940	1.02500	0.28710
C	-6.43630	-0.97740	-3.18390	H	4.29040	-0.21350	0.22630
C	-6.29400	-2.39020	-3.58350	H	5.64760	0.95420	0.09530
C	-5.36520	-3.15960	-3.00610	C	1.46710	2.53700	-1.44660

H	1.39640	1.87380	-2.30620
H	0.49220	2.59550	-0.96670
H	1.74890	3.52160	-1.83170
C	2.31390	2.10900	3.24500
H	1.24230	1.90810	3.17320
H	2.75280	1.48340	4.01930
H	2.46040	3.15110	3.54790
C	5.27810	1.18240	2.66970
C	4.30420	1.73670	-2.15450
C	6.10420	1.59420	4.83760
H	5.81310	2.19650	5.69760
H	6.12760	0.53380	5.09970
H	7.09210	1.88990	4.47730
C	6.16660	1.33760	-3.55500
H	6.13280	2.36500	-3.92330
H	7.19710	1.00830	-3.42510
H	5.64570	0.68550	-4.25900
O	6.22320	0.43610	2.46830
O	3.70710	2.18890	-3.11740
O	5.56980	1.25390	-2.24590
O	5.10550	1.84880	3.83380
C	4.36000	-1.95290	-2.61060
C	3.11580	-1.49650	-2.29690
N	2.75740	-1.29110	-0.96510
C	3.59450	-1.49790	0.08710
H	3.74570	-1.35950	2.20200
C	2.07540	-1.10440	-3.30850
H	2.17150	-1.70170	-4.21660
H	2.22040	-0.05440	-3.58940
H	1.07020	-1.20220	-2.89480
N	5.09580	-2.25280	-5.07310
C	4.73740	-2.11600	-3.97110
C	5.41810	-2.19160	-1.54830
H	6.03130	-3.05880	-1.81200
H	6.09410	-1.32810	-1.51790
C	4.77370	-2.41800	-0.17680
H	5.50780	-2.31040	0.62590
H	4.38050	-3.44350	-0.12340
C	2.95650	-1.52940	1.46100
H	2.24050	-0.70830	1.54190

C	0.46410	-5.19760	-0.12080
C	-0.41000	-4.31070	-0.65360
C	-0.18030	-3.82130	-2.04090
C	0.86770	-4.25870	-2.74860
C	1.82240	-5.21030	-2.15230
C	1.63210	-5.65750	-0.90730
H	-0.85280	-3.10530	-2.49410
H	1.02380	-3.90810	-3.76010
H	2.67850	-5.54300	-2.72480
H	2.33990	-6.35330	-0.47430
C	-0.73670	-5.23250	2.00400
C	-1.71610	-4.26840	1.43530
C	-2.84710	-3.85690	2.30920
C	-2.93960	-4.30980	3.56490
C	-1.94180	-5.24330	4.12740
C	-0.91210	-5.68480	3.39850
H	-3.61530	-3.18490	1.95000
H	-3.76760	-3.99230	4.18580
H	-2.05530	-5.58060	5.14970
H	-0.20330	-6.37340	3.84280
C	-0.06180	5.28270	0.80680
C	-1.14000	4.28800	1.05050
C	-1.31020	3.79830	2.44350
C	-0.53400	4.26280	3.42940
C	0.52510	5.26220	3.17610
C	0.75650	5.73700	1.94830
H	-2.05860	3.05470	2.68550
H	-0.67300	3.89140	4.43660
H	1.13230	5.60520	4.00390
H	1.54800	6.46180	1.79940
C	-1.66060	4.39260	-1.35530
C	-0.67080	5.29440	-1.55760
C	-0.40190	5.81130	-2.91930
C	-1.13790	5.39430	-3.95460
C	-2.22680	4.42220	-3.74370
C	-2.47940	3.94930	-2.51780
H	0.39540	6.52590	-3.08220
H	-0.93860	5.77090	-4.94950
H	-2.82380	4.09480	-4.58510
H	-3.28820	3.24230	-2.38890

C	2.22130	-2.84580	1.78440	C	0.54810	-1.99950	3.55990
H	1.36410	-2.92200	1.10930	H	-0.30640	-2.21840	2.91350
H	2.87720	-3.70090	1.58330	H	0.77960	-0.94120	3.41210
C	1.73630	-2.91390	3.24280	H	0.22670	-2.12990	4.59860
H	2.57800	-2.68550	3.91180				
H	1.46060	-3.95180	3.46300				

(M06-2X/6-31G**:UFF) Energy = -1965.476217

(M06-2X /6-31G**:UFF) Free Energy = -1964.386166

M06-2X/6-31G** Derived free energy = -3808.640362

M06-2X/6-31G** Derived free energy in solution = -3808.658459

Number of Imaginary Frequencies = 1 (-813.60)

ONIOM (M06-2X /6-31G**:UFF) Geometry

C	3.92920	-0.46420	0.04120	C	6.19560	2.66790	-3.53290
C	4.34750	-3.15020	0.83430	C	5.22710	3.38880	-2.95830
C	3.06550	-2.75850	0.20990	H	5.77310	-0.34080	-1.95970
C	2.85030	-1.47220	-0.12710	H	7.16740	0.67400	-3.63160
H	4.50690	-4.18490	1.11510	H	6.82830	3.11340	-4.28970
C	3.26570	3.57090	-1.32830	H	5.07890	4.41980	-3.25450
C	2.42440	3.01400	-0.44470	O	1.65960	-1.08920	-0.68860
C	2.60150	1.59130	-0.04790	O	1.74680	1.03210	0.87130
C	3.68020	0.90210	-0.48500	P	0.74610	-0.12300	0.27770
H	3.14660	4.61030	-1.61290	O	0.27270	-0.91120	1.46720
C	5.29170	-2.22940	1.07080	O	-0.26100	0.48760	-0.66050
C	5.07610	-0.81320	0.67120	H	-1.77470	0.78250	-0.76940
C	6.14700	0.16020	1.00290	C	-0.60850	5.57320	1.26490
C	7.27450	-0.24580	1.59890	C	1.36270	3.84580	0.16700
C	7.49520	-1.66380	1.95640	H	-1.36640	6.22730	1.68010
C	6.56810	-2.59700	1.71480	C	-0.02840	-5.69230	-0.48090
H	6.02840	1.21330	0.78370	C	2.02390	-3.78640	-0.00930
H	8.04330	0.47920	1.83370	H	-0.81540	-6.41350	-0.66830
H	8.42580	-1.94710	2.43110	C	-3.67260	-1.71810	-0.79980
H	6.76160	-3.62500	1.99730	C	-2.37620	-2.07150	-0.50050
C	4.35910	2.77940	-1.92420	C	-2.84570	-1.95780	1.87080
C	4.55900	1.49890	-1.53710	C	-4.14280	-1.60640	1.62360
C	5.62170	0.70080	-2.20890	N	-1.99770	-2.12070	0.80680
C	6.39660	1.25870	-3.14630	H	-0.98630	-2.00270	1.01970

C	-4.51660	-1.19230	0.26830
H	-4.31450	0.05570	0.24750
H	-5.58810	-1.17700	0.07200
C	-1.30770	-2.41280	-1.49090
H	-1.21530	-1.62460	-2.23800
H	-0.35020	-2.51830	-0.98420
H	-1.56360	-3.33390	-2.01820
C	-2.19120	-2.12480	3.21120
H	-1.11570	-1.96750	3.10400
H	-2.58940	-1.40560	3.92620
H	-2.38210	-3.12450	3.60980
C	-5.14190	-1.31090	2.66460
C	-4.14740	-1.72790	-2.19280
C	-5.89150	-1.64680	4.84880
H	-5.59960	-2.24270	5.71130
H	-5.85610	-0.58120	5.08470
H	-6.90250	-1.90210	4.52600
C	-5.91770	-1.18870	-3.62550
H	-6.10900	-2.20420	-3.97950
H	-6.84460	-0.62040	-3.56440
H	-5.21590	-0.69860	-4.30190
O	-6.06060	-0.53740	2.48450
O	-3.52400	-2.12740	-3.15450
O	-5.39250	-1.22440	-2.29550
O	-4.94940	-1.96230	3.82180
C	-4.43600	1.76080	-2.49690
C	-3.18160	1.32020	-2.24050
N	-2.77680	1.09260	-0.92870
C	-3.57680	1.29050	0.13910
H	-3.65210	1.02680	2.24640
C	-2.16600	0.96470	-3.28440
H	-2.30320	1.57090	-4.18070
H	-2.29880	-0.08590	-3.56860
H	-1.15480	1.08690	-2.89010
N	-5.21340	2.12140	-4.93770
C	-4.84630	1.96030	-3.84870
C	-5.45610	1.97220	-1.39880
H	-6.09830	2.82290	-1.64080
H	-6.10200	1.08690	-1.33580
C	-4.75310	2.22070	-0.06560
H	-5.44380	2.12010	0.77560
H	-4.34480	3.24100	-0.05010
C	-2.89620	1.25210	1.48370
H	-2.15530	0.44490	1.48480
C	-0.75430	5.08130	-0.11880
C	0.18030	4.25890	-0.65260
C	-0.00750	3.76640	-2.04490
C	-1.07790	4.13900	-2.75630
C	-2.09990	5.01620	-2.15760
C	-1.94770	5.46490	-0.90800
H	0.71510	3.10230	-2.50000
H	-1.20280	3.78870	-3.77220
H	-2.97450	5.29290	-2.73210
H	-2.70450	6.10590	-0.47330
C	0.43990	5.19710	2.00700
C	1.47780	4.29410	1.44190
C	2.62660	3.94820	2.32070
C	2.68960	4.40930	3.57530
C	1.63950	5.28760	4.13170
C	0.58750	5.66610	3.39930
H	3.43200	3.31870	1.96590
H	3.53200	4.14040	4.19970
H	1.73170	5.63530	5.15260
H	-0.16060	6.31450	3.83960
C	0.19260	-5.24010	0.75950
C	1.26590	-4.24360	1.01740
C	1.43500	-3.77430	2.41760
C	0.65970	-4.25530	3.39630
C	-0.39810	-5.25200	3.12800
C	-0.62590	-5.71180	1.89380
H	2.18170	-3.03300	2.67080
H	0.79770	-3.89840	4.40890
H	-1.00630	-5.60660	3.95020
H	-1.41600	-6.43580	1.73410
C	1.78060	-4.30480	-1.39120
C	0.79620	-5.20950	-1.60600
C	0.52800	-5.70630	-2.97540
C	1.25910	-5.26760	-4.00530
C	2.34180	-4.29170	-3.78080
C	2.59390	-3.83720	-2.54800

H	-0.26480	-6.42340	-3.14840	C	-1.65770	2.55410	3.27360
H	1.06030	-5.62950	-5.00580	H	-2.48000	2.27950	3.94810
H	2.93480	-3.94670	-4.61810	H	-1.37420	3.57410	3.54980
H	3.39830	-3.12700	-2.40890	C	-0.46830	1.62000	3.48080
C	-2.18000	2.56170	1.83430	H	0.36300	1.89120	2.82350
H	-1.34170	2.67430	1.13890	H	-0.70930	0.57820	3.25260
H	-2.85710	3.41130	1.69070	H	-0.11280	1.66660	4.51380

TS4

(B3LYP/6-31G**:UFF) Energy = -1966.174911

(B3LYP/6-31G**:UFF) Free Energy = -1965.094815

M06-2X/6-31G** Derived free energy = -3808.635721

M06-2X/6-31G** Derived free energy in solution = -3808.653842

Number of Imaginary Frequencies = 1 (-831.11)

ONIOM (B3LYP/6-31G**:UFF) Geometry

C	3.88990	-0.38310	-0.33430	C	3.74780	-3.44300	1.93700
C	4.83410	1.95520	-1.62590	C	4.17400	-2.26070	1.43740
C	3.56680	1.99580	-0.86700	C	5.42420	-1.65770	1.97440
C	3.10230	0.87840	-0.27350	C	6.13560	-2.29750	2.90990
H	5.19650	2.85910	-2.10190	C	5.68000	-3.60290	3.42510
C	2.48360	-4.03550	1.45850	C	4.54640	-4.14560	2.96780
C	1.70520	-3.37120	0.59050	H	5.76650	-0.69310	1.62370
C	2.13200	-2.03580	0.08590	H	7.04420	-1.85520	3.29760
C	3.36400	-1.56950	0.38950	H	6.26210	-4.11600	4.17970
H	2.18880	-5.01630	1.81440	H	4.21420	-5.10030	3.35630
C	5.51630	0.80890	-1.73970	O	1.93000	0.90440	0.43910
C	5.01790	-0.42940	-1.08480	O	1.34210	-1.37150	-0.82310
C	5.78600	-1.67920	-1.32110	P	0.69920	0.05350	-0.27130
C	6.91000	-1.66140	-2.04730	O	0.23060	0.78000	-1.51170
C	7.42420	-0.41270	-2.64910	O	-0.28790	-0.20320	0.84780
C	6.77260	0.74690	-2.51240	H	-1.86680	-0.53770	0.54600
H	5.44340	-2.62720	-0.92820	C	-1.91850	-5.20540	-0.90390
H	7.45440	-2.58310	-2.20850	C	0.47120	-4.01370	0.07550
H	8.34260	-0.44430	-3.22100	H	-2.83850	-5.64130	-1.27600
H	7.17350	1.64020	-2.97660	C	1.41440	5.73500	-0.67760

C	2.81410	3.26750	-0.80640
H	0.86930	6.66970	-0.61450
C	-3.25280	2.95990	0.87090
C	-1.99190	2.96010	0.30720
C	-2.85020	1.90890	-1.70500
C	-4.11180	1.81860	-1.15420
N	-1.82530	2.38070	-0.91980
H	-0.87360	2.03600	-1.19040
C	-4.28270	2.10280	0.27670
H	-4.19270	0.96770	0.88500
H	-5.30270	2.33710	0.57550
C	-0.74080	3.53740	0.90510
H	-0.69220	3.35150	1.97690
H	0.13830	3.09790	0.43290
H	-0.72340	4.62220	0.76730
C	-2.42230	1.58800	-3.11140
H	-1.36460	1.31620	-3.11330
H	-3.01660	0.78650	-3.54190
H	-2.55860	2.47180	-3.74590
C	-5.33770	1.36290	-1.84910
C	-3.63880	3.64020	2.12150
C	-6.37940	0.69830	-3.85870
H	-6.54760	-0.33860	-3.55770
H	-7.26460	1.29260	-3.62520
H	-6.14500	0.75210	-4.92120
C	-3.11740	5.25400	3.75780
H	-4.07020	5.77580	3.64310
H	-3.20390	4.54050	4.58060
H	-2.31160	5.96270	3.94720
O	-6.37750	1.12720	-1.25320
O	-4.67180	3.39280	2.72270
O	-2.75990	4.58210	2.53790
O	-5.22060	1.23110	-3.18520
C	-4.63560	-2.00410	-0.57370
C	-3.33890	-1.59200	-0.52660
N	-2.89050	-0.78500	0.51570
C	-3.69980	-0.27300	1.48510
H	-2.29100	1.08880	2.32440
C	-2.29920	-1.89150	-1.56480
H	-2.63940	-2.65990	-2.25850

H	-2.05590	-0.98890	-2.13260
H	-1.36450	-2.20340	-1.09670
N	-5.53400	-3.37230	-2.57850
C	-5.10260	-2.76460	-1.68030
C	-5.65840	-1.57820	0.46700
H	-6.30750	-0.81090	0.03280
H	-6.30000	-2.42640	0.72770
C	-4.98260	-1.04560	1.73750
H	-4.68580	-1.88860	2.37900
H	-5.67460	-0.43320	2.32120
C	-2.99700	0.33290	2.68160
H	-3.74460	0.84470	3.29560
C	-1.83870	-4.77490	0.50450
C	-0.70270	-4.21440	0.98530
C	-0.65390	-3.81440	2.41910
C	-1.70820	-4.02070	3.21670
C	-2.94790	-4.60440	2.67460
C	-3.01290	-4.95910	1.38820
H	0.22930	-3.35270	2.83820
H	-1.66180	-3.73890	4.26030
H	-3.80580	-4.73990	3.32020
H	-3.92850	-5.38360	0.99550
C	-0.86780	-5.04800	-1.71720
C	0.39110	-4.43600	-1.21270
C	1.52250	-4.32930	-2.17270
C	1.38150	-4.72820	-3.44230
C	0.11180	-5.29790	-3.93920
C	-0.94350	-5.45500	-3.13420
H	2.48280	-3.93780	-1.86380
H	2.21760	-4.63960	-4.12420
H	0.04140	-5.60000	-4.97630
H	-1.85670	-5.88120	-3.53250
C	1.26890	4.93850	-1.74370
C	2.01340	3.65420	-1.82980
C	1.81560	2.83520	-3.05410
C	0.96540	3.23010	-4.00900
C	0.20850	4.49460	-3.89770
C	0.35260	5.30280	-2.84240
H	2.33920	1.89700	-3.18650
H	0.82380	2.61060	-4.88530

H	-0.47280	4.77080	-4.69220
H	-0.21630	6.22380	-2.79690
C	2.95090	4.14750	0.39370
C	2.28450	5.32530	0.44300
C	2.40230	6.19700	1.63470
C	3.16930	5.82690	2.66550
C	3.90480	4.54920	2.61900
C	3.80640	3.75420	1.54730
H	1.86450	7.13600	1.67710
H	3.25310	6.46400	3.53650
H	4.52630	4.25740	3.45580
H	4.35720	2.82280	1.53600
C	-2.19830	-0.68270	3.52040
H	-1.45440	-1.14990	2.87020
H	-2.86710	-1.46660	3.89880
C	-1.47450	-0.01810	4.69670
H	-0.80870	0.76200	4.30540
H	-2.20300	0.48800	5.34480
C	-0.65600	-1.01650	5.52060
H	-1.29590	-1.79140	5.95750
H	0.09400	-1.51600	4.89800
H	-0.13020	-0.51890	6.34180

(M06-2X/6-31G**:UFF) Energy = -1965.469618

(M06-2X /6-31G**:UFF) Free Energy = -1964.381035

M06-2X/6-31G** Derived free energy = -3808.634659

M06-2X/6-31G** Derived free energy in solution = -3808.652268

Number of Imaginary Frequencies = 1 (-959.83)

ONIOM (M06-2X /6-31G**:UFF) Geometry

C	3.84410	-0.57020	-0.36640
C	4.91370	1.71220	-1.66290
C	3.66930	1.83000	-0.87460
C	3.14640	0.73980	-0.27960
H	5.32440	2.59390	-2.14120
C	2.24810	-4.13560	1.44410
C	1.48580	-3.40580	0.61600
C	1.98160	-2.09300	0.11560
C	3.25720	-1.72050	0.37090
H	1.90210	-5.10170	1.79360
C	5.51230	0.52240	-1.80420
C	4.94650	-0.68620	-1.14690
C	5.61940	-1.98330	-1.41490
C	6.72300	-2.03510	-2.17030
C	7.30710	-0.81830	-2.77390
C	6.74120	0.38200	-2.60990
H	5.22100	-2.90950	-1.02270
H	7.19750	-2.99040	-2.35470
H	8.20620	-0.90720	-3.37010
H	7.19100	1.25030	-3.07670
C	3.56520	-3.63580	1.88450
C	4.05340	-2.47850	1.38280
C	5.35940	-1.96870	1.88220
C	6.05770	-2.66780	2.78440
C	5.53250	-3.94620	3.30080
C	4.34940	-4.40480	2.87810
H	5.75410	-1.02440	1.53090
H	7.00720	-2.29320	3.14450
H	6.10430	-4.50770	4.02840
H	3.96660	-5.33990	3.26760
O	1.98790	0.83130	0.44650
O	1.20810	-1.37930	-0.76790
P	0.70720	0.09950	-0.26180
O	0.33330	0.82180	-1.52790
O	-0.31460	-0.01450	0.84280
H	-1.84380	-0.22810	0.56880
C	-2.31760	-4.87770	-0.82830
C	0.19790	-3.95650	0.12690

H	-3.28500	-5.20510	-1.19140
C	1.64150	5.63270	-0.57870
C	2.99070	3.14250	-0.79380
H	1.11310	6.57400	-0.48190
C	-3.05750	3.00260	0.85380
C	-1.81320	2.88940	0.28200
C	-2.77250	1.93570	-1.71740
C	-4.02130	1.92090	-1.14860
N	-1.71060	2.35840	-0.96730
H	-0.77340	1.99640	-1.27260
C	-4.18660	2.28320	0.26260
H	-4.26590	1.19530	0.90850
H	-5.17270	2.66440	0.52860
C	-0.50300	3.28680	0.89620
H	-0.39500	2.84440	1.88750
H	0.31660	2.92970	0.27400
H	-0.44430	4.37040	1.00780
C	-2.39980	1.58330	-3.13210
H	-1.32040	1.42820	-3.19410
H	-2.92240	0.69330	-3.47450
H	-2.68580	2.40480	-3.79570
C	-5.25920	1.46450	-1.81570
C	-3.33960	3.62470	2.15790
C	-6.30620	0.63750	-3.73470
H	-6.58910	-0.30920	-3.27030
H	-7.12610	1.35190	-3.64430
H	-6.03890	0.47960	-4.77760
C	-2.56730	4.87380	3.97210
H	-3.42190	5.55170	3.93450
H	-2.77880	4.08000	4.69230
H	-1.66060	5.40980	4.24670
O	-6.31410	1.36240	-1.22190
O	-4.40780	3.50940	2.72340
O	-2.31370	4.31760	2.68240
O	-5.12670	1.15880	-3.11110
C	-4.58260	-1.60370	-0.71640
C	-3.29340	-1.20750	-0.60550
N	-2.87630	-0.47170	0.49860
C	-3.71060	-0.05840	1.47100
H	-2.31860	1.26890	2.35780

C	-2.19340	-1.47580	-1.58010
H	-2.53510	-2.07320	-2.42450
H	-1.75580	-0.53980	-1.93820
H	-1.37620	-1.98800	-1.06970
N	-5.39460	-2.79020	-2.86760
C	-5.00840	-2.27030	-1.90440
C	-5.62830	-1.30490	0.33930
H	-6.30880	-0.53400	-0.03220
H	-6.22570	-2.20320	0.52170
C	-4.97950	-0.85980	1.65240
H	-4.66800	-1.74110	2.23150
H	-5.68190	-0.29790	2.27210
C	-3.03840	0.51220	2.68960
H	-3.78750	1.00500	3.31680
C	-2.16350	-4.50830	0.59130
C	-0.96740	-4.07840	1.06090
C	-0.84350	-3.74880	2.50790
C	-1.89050	-3.89380	3.32830
C	-3.19310	-4.33630	2.80000
C	-3.32610	-4.62180	1.50160
H	0.08950	-3.38970	2.91900
H	-1.79110	-3.66280	4.38050
H	-4.04250	-4.42170	3.46530
H	-4.28780	-4.94100	1.11940
C	-1.27200	-4.80740	-1.66000
C	0.05180	-4.33830	-1.16840
C	1.16590	-4.30540	-2.15340
C	0.95890	-4.64590	-3.43080
C	-0.37030	-5.07600	-3.91260
C	-1.41780	-5.15820	-3.08650
H	2.16550	-4.01610	-1.85650
H	1.78400	-4.61320	-4.13080
H	-0.49230	-5.33530	-4.95640
H	-2.37660	-5.48090	-3.47470
C	1.50110	4.88920	-1.68310
C	2.21660	3.59190	-1.81260
C	2.00360	2.82140	-3.06580
C	1.17380	3.27440	-4.01280
C	0.45380	4.55620	-3.86330
C	0.60890	5.32040	-2.77740

H	2.49890	1.87260	-3.22710
H	1.02170	2.69020	-4.91140
H	-0.21000	4.88120	-4.65410
H	0.06530	6.25480	-2.70330
C	3.13300	3.97100	0.44240
C	2.48810	5.15820	0.53410
C	2.60600	5.97570	1.76360
C	3.35380	5.54830	2.78630
C	4.06920	4.26190	2.69250
C	3.96990	3.51510	1.58670
H	2.08340	6.92100	1.84050
H	3.43750	6.14580	3.68490
H	4.67740	3.92480	3.52200
H	4.50780	2.57730	1.54020
C	-2.25050	-0.53730	3.48210
H	-1.55990	-1.03960	2.79780
H	-2.93490	-1.28640	3.89990
C	-1.44480	0.11160	4.60430
H	-0.74010	0.82300	4.15590
H	-2.11470	0.69270	5.25140
C	-0.68080	-0.91450	5.43510
H	-1.36710	-1.62160	5.91130
H	0.00690	-1.48660	4.80560
H	-0.09540	-0.43430	6.22310

Additional catalysts

9-phenanthryl derived CPA *Type I* TS

(B3LYP/6-31G**:UFF) Energy = -1966.177171

(B3LYP/6-31G**:UFF) Free Energy = -1965.094867

M06-2X/6-31G** Derived free energy = -3808.657496

M06-2X/6-31G** Derived free energy in solution = -3808.677416

Number of Imaginary Frequencies = 1 (-709.76)

ONIOM (B3LYP/6-31G**:UFF) Geometry

C	-0.65040	3.83120	-0.11420
C	-3.13850	4.26100	-1.40680
C	-2.73670	2.89280	-1.02400
C	-1.53570	2.67620	-0.45530
H	-4.10090	4.41880	-1.87980
C	3.14130	3.10100	1.82570
C	2.84890	2.50470	0.65940
C	1.50640	2.70030	0.05570
C	0.62400	3.55790	0.60520
H	4.11300	2.95660	2.28360
C	-2.31790	5.29410	-1.17660
C	-1.00350	5.07750	-0.51440
C	-0.12300	6.26290	-0.34870
C	-0.53320	7.47630	-0.73640
C	-1.86160	7.68600	-1.35040
C	-2.70020	6.66660	-1.56320
H	0.86900	6.16560	0.07210
H	0.12380	8.32690	-0.60710
H	-2.15410	8.68690	-1.64110
H	-3.66230	6.85680	-2.02390
C	2.13770	3.94630	2.50670
C	0.92820	4.16260	1.93690
C	-0.09090	4.94930	2.68300
C	0.20020	5.46870	3.88130
C	1.53210	5.25610	4.47900
C	2.45050	4.53230	3.83040
H	-1.08110	5.09970	2.27420
H	-0.54490	6.03910	4.42070

H	1.76030	5.68030	5.44820
H	3.42180	4.37040	4.28100
O	-1.18270	1.40240	-0.08370
O	1.18230	2.03200	-1.09530
P	0.16170	0.77020	-0.82930
O	-0.15920	0.23700	-2.20600
O	0.72190	-0.17770	0.21500
H	-0.11290	-1.17320	1.29080
C	3.83880	1.62700	-0.01170
C	-3.66490	1.77350	-1.27520
C	-0.89280	-4.00170	-0.56420
C	-0.93570	-2.93640	-1.44250
C	1.48350	-2.90480	-1.60990
C	1.58500	-3.98450	-0.76000
N	0.24210	-2.38500	-1.86330
H	0.17590	-1.40370	-2.22990
C	0.40070	-4.39200	0.01550
H	0.48740	-3.81000	1.13590
H	0.43300	-5.41700	0.38300
C	-2.15690	-2.29850	-2.04420
H	-2.93920	-2.17240	-1.29800
H	-1.89170	-1.33060	-2.47100
H	-2.57160	-2.94110	-2.82750
C	2.59820	-2.20070	-2.33170
H	3.54830	-2.33880	-1.82400
H	2.70370	-2.59650	-3.34800
H	2.36760	-1.13480	-2.40780
C	2.81210	-4.74690	-0.46210
C	-2.06080	-4.78740	-0.11470
C	4.95300	-5.44180	-1.15840
H	4.70070	-6.50050	-1.25900
H	5.67290	-5.14890	-1.92190
H	5.35360	-5.27240	-0.15760
C	-4.40200	-5.11730	-0.18890
H	-4.58940	-4.88340	0.86190
H	-5.22380	-4.75900	-0.80850
H	-4.27100	-6.19490	-0.30340
O	2.92420	-5.46330	0.52090
O	-1.97270	-5.70810	0.68410
O	-3.23680	-4.41230	-0.66030
O	3.78980	-4.62900	-1.39140
C	-2.24040	-3.08790	2.99100
C	-1.77480	-2.02880	2.27330
N	-0.42560	-1.94250	1.93230
C	0.51060	-2.86240	2.28810
H	2.58300	-3.31010	2.11560
C	-2.61120	-0.88180	1.79640
H	-2.23860	0.06290	2.20460
H	-2.55480	-0.78100	0.70900
H	-3.65030	-1.01540	2.09290
N	-4.76940	-3.34940	3.47800
C	-3.63160	-3.20470	3.26230
C	-1.33190	-4.22140	3.43650
H	-1.61230	-4.54600	4.44370
H	-1.47750	-5.07920	2.77320
C	0.13590	-3.77660	3.43870
H	0.80980	-4.63730	3.45410
H	0.33930	-3.20000	4.35280
C	1.95610	-2.41620	2.18590
H	2.08160	-1.83470	1.26780
C	2.42920	-1.55800	3.38970
C	1.72070	-0.20540	3.55680
H	1.64820	0.28700	2.58070
H	0.68720	-0.36650	3.88790
C	2.43060	0.70940	4.55990
H	2.51210	0.23890	5.54660
H	1.88810	1.65140	4.68780
H	3.44570	0.95340	4.22650
C	4.30250	0.55190	0.63990
H	4.00340	0.36820	1.66290
C	-3.68060	1.19970	-2.48380
H	3.50180	-1.38520	3.24560
H	2.35210	-2.13790	4.31860
C	4.25900	1.86530	-1.42550
C	5.03330	0.95170	-2.07130
C	5.39090	1.19520	-3.50000
C	4.99520	2.31010	-4.12190
C	4.21140	3.32280	-3.40030
C	3.86810	3.12020	-2.12660
H	5.96490	0.48370	-4.07090

H	5.25830	2.47620	-5.15880
H	3.92210	4.23700	-3.90260
H	3.31480	3.89370	-1.61110
C	-4.55120	1.25580	-0.19570
C	-5.33680	0.16910	-0.42140
C	-6.22850	-0.31160	0.67450
C	-6.24890	0.30920	1.85850
C	-5.39030	1.47940	2.09810
C	-4.58890	1.93060	1.13050
H	-6.88310	-1.15790	0.54290
H	-6.89950	-0.04710	2.64700
H	-5.41140	1.97330	3.06090
H	-3.96820	2.79460	1.32910
H	-3.03420	1.57300	-3.27040
C	5.17750	-0.42440	-0.03250
C	5.52300	-0.25780	-1.33180
C	6.39980	-1.28400	-1.97040
C	6.83290	-2.33920	-1.27090
C	6.44900	-2.50350	0.14100
C	5.66040	-1.60070	0.72750
H	6.70840	-1.20740	-3.00020
H	7.46860	-3.07850	-1.74120
H	6.80770	-3.35730	0.70090
H	5.38440	-1.73060	1.76550
C	-4.52580	0.01960	-2.74470
C	-5.29870	-0.50380	-1.76140
C	-6.05990	-1.75570	-2.04670
C	-5.99180	-2.33360	-3.25180
C	-5.16700	-1.73810	-4.31690
C	-4.47180	-0.62310	-4.07850
H	-6.66280	-2.23740	-1.29400
H	-6.53970	-3.24650	-3.44730
H	-5.12000	-2.21080	-5.28940
H	-3.85840	-0.19760	-4.86300

(M06-2X/6-31G**:UFF) Energy = -1965.471205

(M06-2X /6-31G**:UFF) Free Energy = -1964.380474

M06-2X/6-31G** Derived free energy = -3808.656012

M06-2X/6-31G** Derived free energy in solution = -3808.675381

Number of Imaginary Frequencies = 1 (-851.90)

ONIOM (M06-2X /6-31G**:UFF) Geometry

C	-0.33650	3.81910	-0.05520
C	-2.79410	4.45710	-1.31530
C	-2.49760	3.05680	-0.95070
C	-1.31000	2.74050	-0.39990
H	-3.74570	4.69530	-1.77630
C	3.41390	2.81010	1.82730
C	3.06000	2.23110	0.66870
C	1.72030	2.51260	0.09070
C	0.91490	3.43680	0.65200
H	4.38470	2.60730	2.26470
C	-1.89180	5.41980	-1.08610
C	-0.59270	5.09350	-0.43990
C	0.38110	6.20340	-0.27350
C	0.06530	7.44920	-0.64710
C	-1.24830	7.76940	-1.24520
C	-2.16750	6.82180	-1.45740
H	1.36670	6.02360	0.13490
H	0.78940	8.24350	-0.51820
H	-1.46280	8.79290	-1.52490
H	-3.11580	7.09210	-1.90660
C	2.48140	3.71680	2.52840
C	1.28000	4.01440	1.98040
C	0.32830	4.86510	2.74520
C	0.67370	5.36050	3.93930

C	1.99860	5.05800	4.51350
C	2.85540	4.27650	3.84770
H	-0.65640	5.08220	2.35340
H	-0.02240	5.97780	4.49250
H	2.27130	5.46350	5.47930
H	3.82160	4.04940	4.28080
O	-1.04140	1.43980	-0.05520
O	1.34020	1.88830	-1.06810
P	0.21450	0.72940	-0.84900
O	-0.18140	0.29470	-2.23030
O	0.70750	-0.31430	0.12730
H	-0.15320	-1.25160	1.11420
C	3.98550	1.29730	-0.01870
C	-3.51760	2.02120	-1.20260
C	-1.26060	-3.84070	-0.52170
C	-1.23290	-2.77810	-1.39500
C	1.16740	-2.91700	-1.58390
C	1.19590	-4.02690	-0.78140
N	-0.03300	-2.33080	-1.85410
H	-0.03050	-1.36140	-2.24970
C	-0.00380	-4.41340	-0.02630
H	0.17280	-3.96930	1.12670
H	-0.06140	-5.47030	0.23940
C	-2.40930	-2.05030	-1.98290
H	-3.12090	-1.76810	-1.21080
H	-2.06120	-1.16090	-2.50940
H	-2.94210	-2.70760	-2.67520
C	2.33970	-2.24160	-2.23450
H	3.20710	-2.27180	-1.57960
H	2.60830	-2.75200	-3.16310
H	2.08200	-1.20350	-2.45680
C	2.38840	-4.83950	-0.49740
C	-2.49360	-4.49560	-0.03610
C	4.51430	-5.54180	-1.15110
H	4.22160	-6.58810	-1.26170
H	5.25130	-5.26930	-1.90420
H	4.91030	-5.39580	-0.14510
C	-4.83520	-4.53510	0.02390
H	-4.87420	-4.60890	1.11090
H	-5.64600	-3.90800	-0.34270
H	-4.88930	-5.53410	-0.41330
O	2.44960	-5.61150	0.43910
O	-2.48110	-5.50730	0.63580
O	-3.63060	-3.88230	-0.39280
O	3.39160	-4.69010	-1.37930
C	-2.41840	-3.02380	2.79620
C	-1.88900	-2.01730	2.06070
N	-0.52920	-1.99410	1.76680
C	0.32390	-2.94650	2.18640
H	2.33920	-3.58150	1.97480
C	-2.64720	-0.85040	1.52100
H	-2.41720	0.04680	2.10430
H	-2.33770	-0.63180	0.49610
H	-3.71890	-1.03980	1.55580
N	-4.96790	-3.07010	3.23750
C	-3.82480	-3.03520	3.04000
C	-1.58450	-4.16800	3.33400
H	-1.92070	-4.42520	4.34230
H	-1.73280	-5.05210	2.70690
C	-0.11170	-3.76710	3.37780
H	0.54200	-4.63810	3.47220
H	0.06810	-3.12470	4.25240
C	1.79140	-2.63680	2.04250
H	1.94450	-2.06740	1.11880
C	2.31170	-1.80990	3.23710
C	1.63320	-0.44860	3.39910
H	1.57660	0.04060	2.41940
H	0.59480	-0.59060	3.72510
C	2.36040	0.43860	4.40580
H	2.42080	-0.04390	5.38670
H	1.84750	1.39480	4.53580
H	3.38320	0.64870	4.07820
C	4.41870	0.21240	0.63720
H	4.13360	0.05460	1.66830
C	-3.61520	1.48790	-2.42570
H	3.38620	-1.66660	3.08620
H	2.22040	-2.38880	4.16500
C	4.39390	1.50560	-1.44080
C	5.12150	0.55530	-2.08830
C	5.46520	0.76900	-3.52500

C	5.10800	1.89400	-4.15180
C	4.38180	2.94720	-3.42780
C	4.04970	2.77110	-2.14730
H	5.99720	0.02710	-4.09780
H	5.36080	2.03870	-5.19450
H	4.12690	3.86950	-3.93380
H	3.54200	3.57430	-1.63050
C	-4.43110	1.56460	-0.11820
C	-5.33560	0.57810	-0.35910
C	-6.24400	0.15230	0.74600
C	-6.16070	0.71960	1.95380
C	-5.17590	1.78200	2.20860
C	-4.36130	2.18770	1.23190
H	-6.98890	-0.61380	0.60470
H	-6.82210	0.40170	2.74960
H	-5.11660	2.23580	3.18940
H	-3.64750	2.97340	1.44240
H	-2.95320	1.82080	-3.21740
C	5.24980	-0.80090	-0.03510
C	5.58010	-0.66200	-1.34150
C	6.41520	-1.72310	-1.97870
C	6.83030	-2.77930	-1.27000
C	6.46280	-2.91340	0.14940
C	5.70790	-1.98090	0.73430
H	6.70980	-1.66970	-3.01410
H	7.43720	-3.54320	-1.73910
H	6.80210	-3.77170	0.71480
H	5.44090	-2.08930	1.77750
C	-4.58560	0.41230	-2.70170
C	-5.39800	-0.05010	-1.71980
C	-6.30240	-1.19680	-2.02760
C	-6.31610	-1.74380	-3.24870
C	-5.44080	-1.21710	-4.30960
C	-4.62090	-0.19480	-4.05260
H	-6.95110	-1.62490	-1.28050
H	-6.96970	-2.58020	-3.46110
H	-5.46010	-1.66420	-5.29500
H	-3.97330	0.18080	-4.83500

9-phenanthryl derived CPA *Type II* TS

(B3LYP/6-31G**:UFF) Energy = -1966.591634

(B3LYP/6-31G**:UFF) Free Energy = -1965.513526

M06-2X/6-31G** Derived free energy = -3808.658381

M06-2X/6-31G** Derived free energy in solution = -3808.678998

Number of Imaginary Frequencies = 1 (-835.29)

ONIOM (B3LYP/6-31G**:UFF) Geometry

C	3.25280	-2.32530	-0.74610
C	5.25940	-0.68350	-1.86670
C	4.21460	-0.08090	-1.00350
C	3.19590	-0.85070	-0.56510
H	6.04800	-0.05570	-2.26540
C	0.56680	-4.70570	1.64540
C	0.07570	-3.81030	0.77640
C	1.00250	-2.96670	-0.02580
C	2.33790	-3.14870	0.08150
H	-0.10630	-5.32020	2.23280
C	5.22350	-1.98980	-2.16460
C	4.17820	-2.86300	-1.57240
C	4.22440	-4.30920	-1.90120
C	5.18390	-4.79380	-2.69850
C	6.22510	-3.91760	-3.27870
C	6.24800	-2.60280	-3.03300

H	3.47580	-4.98930	-1.51500
H	5.19940	-5.85090	-2.93100
H	6.98290	-4.35390	-3.91670
H	7.02690	-1.99230	-3.47420
C	2.02160	-4.84560	1.83750
C	2.87750	-4.08880	1.11420
C	4.33630	-4.17810	1.40620
C	4.79200	-5.03180	2.33010
C	3.85170	-5.88400	3.08100
C	2.53760	-5.79240	2.85340
H	5.04610	-3.54490	0.89220
H	5.85260	-5.09090	2.53830
H	4.22780	-6.57450	3.82480
H	1.85190	-6.41220	3.41770
O	2.15200	-0.29940	0.13320
O	0.49600	-2.09130	-0.95360
P	0.67230	-0.48550	-0.59040
O	0.65550	0.24070	-1.91360
O	-0.32350	-0.09210	0.47150
H	-1.93410	0.28510	0.41260
C	-1.38710	-3.66520	0.64730
C	4.29010	1.36510	-0.68080
C	-1.34010	4.01100	0.06160
C	-0.36300	3.32910	-0.62710
C	-1.99030	2.48000	-2.20650
C	-3.01380	3.11010	-1.53370
N	-0.73160	2.52910	-1.67090
H	-0.06750	1.76930	-1.95070
C	-2.74290	3.68520	-0.20940
H	-3.07440	2.78760	0.64980
H	-3.45450	4.44280	0.10780
C	1.11360	3.36820	-0.38610
H	1.34970	3.31880	0.67370
H	1.58720	2.53430	-0.90060
H	1.52120	4.30680	-0.76880
C	-2.03770	1.75380	-3.52220
H	-1.82450	2.46010	-4.33150
H	-3.01200	1.32170	-3.72010
H	-1.26430	0.98260	-3.53590
C	-4.41870	3.22680	-1.98690
C	-1.10720	4.98460	1.14510
C	-6.02080	2.84680	-3.68710
H	-6.60750	2.09710	-3.15440
H	-6.44470	3.83610	-3.51810
H	-5.98490	2.61550	-4.74920
C	0.45210	6.33130	2.30820
H	-0.12260	7.24710	2.16470
H	0.20110	5.90080	3.27850
H	1.51920	6.53260	2.24260
O	-5.29670	3.67120	-1.27500
O	-1.98870	5.39090	1.87380
O	0.17680	5.39330	1.25250
O	-4.64570	2.80700	-3.24470
C	-5.22070	0.36100	-0.07460
C	-3.91620	0.02860	-0.25190
N	-2.92750	0.59060	0.54820
C	-3.15700	1.57310	1.46130
H	-1.12570	2.08130	1.83520
C	-3.40560	-0.89300	-1.31930
H	-2.56370	-1.48070	-0.95320
H	-4.19010	-1.55590	-1.67990
H	-3.03350	-0.31860	-2.16950
N	-7.04530	-0.52410	-1.66320
C	-6.20990	-0.14910	-0.95270
C	-5.67080	1.36540	0.97130
H	-5.96960	2.28720	0.46670
H	-6.55690	0.98490	1.48670
C	-4.57140	1.65110	2.00300
H	-4.60990	0.89860	2.80170
H	-4.72800	2.62000	2.48020
C	-2.02230	1.86320	2.41860
H	-2.27740	2.75910	2.99090
C	-1.70130	0.68660	3.36420
C	-0.56170	0.99390	4.34870
H	-0.44700	0.12020	4.99980
H	-0.85520	1.82280	5.00520
C	0.78850	1.30610	3.69080
H	0.78270	2.27890	3.19060
H	1.58230	1.33700	4.44220
H	1.05100	0.55760	2.93930

C	-2.06230	-3.04320	1.62220
H	-1.52910	-2.61900	2.46530
C	4.34510	2.25040	-1.68560
H	-1.42040	-0.17440	2.75520
H	-2.59640	0.41940	3.93590
C	-2.12670	-4.27660	-0.49230
C	-3.48530	-4.21250	-0.53590
C	-4.19520	-4.83530	-1.69140
C	-3.51020	-5.44400	-2.66480
C	-2.04230	-5.51320	-2.60830
C	-1.38550	-4.96630	-1.58300
H	-5.26950	-4.81410	-1.77560
H	-4.03470	-5.89200	-3.49910
H	-1.49700	-6.01190	-3.39930
H	-0.30590	-5.03430	-1.56180
C	4.30760	1.85530	0.73280
C	4.28950	3.19130	0.99170
C	4.23080	3.64750	2.41190
C	4.24910	2.76350	3.41370
C	4.34520	1.32490	3.13020
C	4.37930	0.89250	1.86830
H	4.15900	4.69120	2.67080
H	4.20100	3.10430	4.44010
H	4.39370	0.61810	3.94860
H	4.47780	-0.16970	1.69180
H	4.33340	1.90750	-2.71410
C	-3.53160	-2.94080	1.57650
C	-4.22850	-3.50080	0.55790
C	-5.71370	-3.34970	0.54800
C	-6.33590	-2.67430	1.52170
C	-5.56290	-2.07520	2.62250
C	-4.23460	-2.20540	2.65270
H	-6.32180	-3.76500	-0.23900
H	-7.41230	-2.56180	1.50180
H	-6.07380	-1.52960	3.40510
H	-3.67390	-1.76600	3.46720
C	4.36600	3.70010	-1.41870
C	4.32680	4.16700	-0.14710
C	4.30990	5.64480	0.06640
C	4.32360	6.48330	-0.97640
C	4.36250	5.95890	-2.35190
C	4.38500	4.64060	-2.56320
H	4.28520	6.07690	1.05350
H	4.30850	7.55330	-0.81250
H	4.37320	6.64440	-3.18940
H	4.41130	4.26280	-3.57780

(M06-2X/6-31G**:UFF) Energy = -1965.467908

(M06-2X /6-31G**:UFF) Free Energy = -1964.376623

M06-2X/6-31G** Derived free energy = -3808.654263

M06-2X/6-31G** Derived free energy in solution = -3808.674165

Number of Imaginary Frequencies = 1 (-900.45)

ONIOM (M06-2X /6-31G**:UFF) Geometry

C	-3.22610	2.27840	-0.62990
C	-5.24280	0.74850	-1.88740
C	-4.20090	0.07090	-1.08020
C	-3.18200	0.79500	-0.57310
H	-6.03200	0.15950	-2.34040
C	-0.46690	4.52580	1.80540
C	-0.00360	3.68200	0.87130
C	-0.95400	2.83980	0.09420
C	-2.28510	3.02460	0.24130
H	0.22130	5.14250	2.37250
C	-5.20520	2.07650	-2.06450
C	-4.15330	2.89030	-1.40180

C	-4.19000	4.35930	-1.60980
C	-5.15020	4.91550	-2.35820
C	-6.20070	4.09840	-3.00380
C	-6.23020	2.76750	-2.87140
H	-3.43380	5.00010	-1.17460
H	-5.15880	5.98840	-2.50190
H	-6.95920	4.59210	-3.59760
H	-7.01520	2.20160	-3.35890
C	-1.91380	4.62840	2.07170
C	-2.79140	3.90620	1.33890
C	-4.23900	3.96750	1.68560
C	-4.66620	4.76350	2.67250
C	-3.70430	5.57900	3.43690
C	-2.39860	5.51230	3.15710
H	-4.96210	3.35980	1.15890
H	-5.71910	4.80270	2.92060
H	-4.05760	6.22340	4.23160
H	-1.69700	6.10580	3.72990
O	-2.15790	0.17990	0.09990
O	-0.47220	1.98290	-0.86620
P	-0.67340	0.38150	-0.56680
O	-0.58850	-0.28970	-1.91030
O	0.25170	-0.06980	0.53900
H	1.74000	-0.44040	0.46260
C	1.44870	3.61900	0.60790
C	-4.27590	-1.39640	-0.88490
C	1.33130	-3.80930	0.46400
C	0.39530	-3.13190	-0.27810
C	2.03990	-2.59360	-1.96100
C	3.02890	-3.20090	-1.22880
N	0.77940	-2.53250	-1.44040
H	0.13330	-1.80380	-1.82200
C	2.74890	-3.67980	0.12660
H	3.19500	-2.79050	0.91730
H	3.38910	-4.49150	0.47230
C	-1.06100	-2.98170	0.03290
H	-1.19090	-2.39510	0.94610
H	-1.55670	-2.45850	-0.78390
H	-1.51480	-3.95970	0.18580
C	2.14930	-2.01670	-3.34650

H	2.23330	-2.82800	-4.07500
H	3.03710	-1.39640	-3.45150
H	1.25550	-1.42810	-3.56770
C	4.43730	-3.35080	-1.65460
C	1.06720	-4.51670	1.73070
C	6.09790	-2.93340	-3.24460
H	6.72240	-2.39300	-2.53140
H	6.41030	-3.97830	-3.28780
H	6.15900	-2.46350	-4.22410
C	-0.47800	-5.14540	3.37020
H	-0.15300	-6.18740	3.37430
H	0.05640	-4.60370	4.15440
H	-1.55390	-5.07370	3.52060
O	5.27630	-3.87100	-0.94690
O	1.95050	-4.99290	2.41250
O	-0.22670	-4.54770	2.09850
O	4.72080	-2.84700	-2.86100
C	4.95560	-0.37400	-0.37830
C	3.62300	-0.14520	-0.43340
N	2.77930	-0.66470	0.53800
C	3.19470	-1.46110	1.54170
H	1.25810	-2.06890	2.11800
C	2.89230	0.65590	-1.46310
H	2.33450	1.45150	-0.96820
H	3.56630	1.08160	-2.20560
H	2.12740	0.04960	-1.95550
N	6.48920	0.41380	-2.30720
C	5.78850	0.08050	-1.44380
C	5.60390	-1.16750	0.74050
H	5.93060	-2.13910	0.35960
H	6.50070	-0.64150	1.08140
C	4.65210	-1.35650	1.92820
H	4.70180	-0.48610	2.59610
H	4.93400	-2.22850	2.52270
C	2.16280	-1.69770	2.61200
H	2.51940	-2.46890	3.30240
C	1.76870	-0.41310	3.35820
C	0.59520	-0.64960	4.31410
H	0.39150	0.29210	4.83580
H	0.88690	-1.37260	5.08710

C	-0.67970	-1.12080	3.60880
H	-0.58870	-2.15310	3.25170
H	-1.53380	-1.09070	4.29070
H	-0.90080	-0.49480	2.73740
C	2.25550	3.10050	1.54270
H	1.84110	2.73710	2.47600
C	-4.26990	-2.19260	-1.96240
H	1.46910	0.33350	2.61710
H	2.62950	-0.02070	3.91260
C	2.02940	4.14260	-0.66280
C	3.36910	4.05550	-0.88680
C	3.91470	4.56370	-2.17950
C	3.10560	5.10450	-3.09570
C	1.66230	5.21570	-2.83810
C	1.15100	4.76780	-1.68960
H	4.96550	4.50890	-2.41280
H	3.51130	5.46880	-4.03090
H	1.01720	5.66760	-3.58070
H	0.08800	4.87420	-1.51970
C	-4.32420	-2.00470	0.48110
C	-4.29270	-3.35720	0.62610
C	-4.22490	-3.93080	2.00270
C	-4.24100	-3.13400	3.07540
C	-4.35350	-1.67760	2.91440
C	-4.40460	-1.14160	1.69340
H	-4.13540	-4.99150	2.17170
H	-4.17900	-3.55920	4.06890
H	-4.40250	-1.04240	3.78950
H	-4.51360	-0.06920	1.60610
H	-4.22570	-1.76190	-2.95660
C	3.70740	2.99730	1.31440
C	4.25560	3.44070	0.15710
C	5.72770	3.28400	-0.03580
C	6.48250	2.71980	0.91450
C	5.87080	2.25080	2.16890
C	4.55630	2.38340	2.36070
H	6.22030	3.60670	-0.93840
H	7.54780	2.60260	0.76290
H	6.48830	1.79380	2.93130
H	4.11320	2.03270	3.28430
C	-4.27630	-3.65980	-1.82010
C	-4.28590	-4.23250	-0.59220
C	-4.29050	-5.72280	-0.50370
C	-4.26730	-6.47060	-1.61330
C	-4.24320	-5.83190	-2.94010
C	-4.25060	-4.50030	-3.04000
H	-4.32160	-6.23570	0.44390
H	-4.27190	-7.55070	-1.54010
H	-4.22330	-6.44430	-3.83230
H	-4.23480	-4.03800	-4.01920

1-naphthyl derived CPA *Type I* TS

(B3LYP/6-31G**:UFF) Energy = -1966.206822

(B3LYP/6-31G**:UFF) Free Energy = -1965.220187

M06-2X/6-31G** Derived free energy = -3501.590140

M06-2X/6-31G** Derived free energy in solution = -3501.608475

Number of Imaginary Frequencies = 1 (-715.42)

ONIOM (B3LYP/6-31G**:UFF) Geometry

C	-3.80390	-0.55570	-0.00780
C	-4.50140	-3.27100	-0.44440
C	-3.08320	-2.89460	-0.28420
C	-2.73860	-1.60090	-0.13130

H	-4.76070	-4.31390	-0.58680
C	-2.86050	3.60630	0.63320
C	-2.29380	2.93540	-0.38230
C	-2.54350	1.47580	-0.51940
C	-3.40630	0.84660	0.30220
H	-2.72280	4.67740	0.72470
C	-5.45450	-2.33190	-0.42910
C	-5.09880	-0.90340	-0.21780
C	-6.20800	0.08510	-0.26940
C	-7.47270	-0.31600	-0.44430
C	-7.81750	-1.74420	-0.60470
C	-6.87550	-2.69270	-0.60370
H	-6.01400	1.14610	-0.18660
H	-8.26500	0.42090	-0.47970
H	-8.85530	-2.02300	-0.73550
H	-7.16450	-3.72900	-0.73330
C	-3.67920	2.89540	1.63750
C	-3.93620	1.57400	1.49440
C	-4.69950	0.86690	2.55750
C	-5.16110	1.53880	3.61860
C	-4.90820	2.98670	3.74790
C	-4.20380	3.63080	2.81120
H	-4.87960	-0.19790	2.49200
H	-5.71520	1.02000	4.39020
H	-5.28660	3.52380	4.60800
H	-4.01200	4.69110	2.91890
O	-1.41920	-1.28180	0.07260
O	-1.92960	0.77900	-1.52540
P	-0.72310	-0.19140	-0.96980
O	-0.17160	-0.86430	-2.20440
O	0.23170	0.56820	-0.06660
H	1.16100	-0.26340	1.14120
C	-0.15970	5.08130	-3.46650
C	-1.51650	3.65810	-1.42060
H	0.35120	5.62170	-4.25370
C	-0.03450	-5.93370	-0.22670
C	-2.05850	-3.95680	-0.29680
H	0.74510	-6.68420	-0.18820
C	4.11650	-1.17130	-0.52490
C	3.06510	-1.40250	-1.39060

C	2.84320	0.97220	-1.83670
C	3.88970	1.26060	-0.98800
N	2.43330	-0.32890	-1.95370
H	1.46100	-0.50510	-2.30490
C	4.38660	0.20710	-0.08580
H	3.78780	0.36320	1.01640
H	5.40080	0.36860	0.27760
C	2.51110	-2.72900	-1.83790
H	2.51590	-3.45750	-1.02960
H	1.49310	-2.58620	-2.20520
H	3.12490	-3.14420	-2.64440
C	2.07740	1.93740	-2.69800
H	1.09710	1.52300	-2.94070
H	1.95560	2.89420	-2.19700
H	2.61790	2.12460	-3.63180
C	4.52020	2.58050	-0.80810
C	4.99840	-2.20640	0.05160
C	4.91030	4.74440	-1.65430
H	4.49940	5.37310	-2.44380
H	4.71610	5.17590	-0.67070
H	5.99030	4.63210	-1.77970
C	5.57840	-4.49090	0.20380
H	5.34430	-5.39870	-0.35140
H	6.63850	-4.24570	0.11900
H	5.31660	-4.61360	1.25760
O	5.24560	2.84570	0.13860
O	5.87650	-1.95100	0.86290
O	4.77060	-3.45890	-0.39620
O	4.25640	3.47210	-1.79310
C	3.04050	-2.24300	3.05100
C	2.00040	-1.85060	2.26500
N	1.90470	-0.52690	1.82910
C	2.79810	0.44710	2.14950
H	3.22450	2.51250	1.89170
C	0.88980	-2.74620	1.80640
H	0.98680	-3.73790	2.24750
H	-0.08250	-2.32180	2.07210
H	0.88710	-2.84210	0.71620
N	3.34530	-4.72000	3.73790
C	3.17610	-3.60780	3.42750

C	4.14340	-1.28700	3.47280	H	1.89730	2.31160	4.04190
H	4.42960	-1.48890	4.51000	C	-0.33850	4.51950	-1.08030
H	5.03050	-1.46450	2.85770	C	0.29080	5.20840	-2.06490
C	3.68120	0.16910	3.34990	C	1.44890	6.07430	-1.74640
H	4.53230	0.85500	3.34230	C	1.88870	6.17120	-0.48840
H	3.07300	0.43200	4.22740	C	1.23480	5.40060	0.58420
C	2.33650	1.87580	1.94210	C	0.18710	4.61460	0.30800
H	1.81000	1.94340	0.98570	H	1.94260	6.63380	-2.53110
C	1.39920	2.39400	3.06710	H	2.73190	6.80830	-0.25520
C	0.01140	1.73920	3.13080	H	1.61030	5.47320	1.59610
H	-0.40360	1.67610	2.11910	H	-0.26380	4.04730	1.10800
H	0.10430	0.70340	3.47950	C	-1.26330	-4.24980	-1.52510
C	-0.95030	2.49560	4.05240	C	-0.29000	-5.19120	-1.48040
H	-0.55210	2.58040	5.07020	C	0.50890	-5.49020	-2.69100
H	-1.91630	1.98530	4.11850	C	0.26570	-4.83930	-3.83400
H	-1.13500	3.51180	3.68530	C	-0.80380	-3.82380	-3.89490
C	-1.90090	3.55600	-2.70710	C	-1.53160	-3.54580	-2.80670
H	-2.75780	2.95270	-2.98250	H	1.29480	-6.23430	-2.65400
C	-1.19080	4.29020	-3.76990	H	0.85220	-5.05570	-4.71740
H	-1.51670	4.19450	-4.79750	H	-0.99810	-3.30470	-4.82450
C	-1.82800	-4.65840	0.82560	H	-2.31370	-2.80060	-2.87270
C	-0.77300	-5.68960	0.86020	H	-2.39440	-4.45770	1.72720
H	-0.58740	-6.23630	1.77570				
H	1.27770	3.46970	2.90000				

(M06-2X/6-31G**:UFF) Energy = -1965.502488

(M06-2X /6-31G**:UFF) Free Energy = -1964.505702

M06-2X/6-31G** Derived free energy = -3501.588406

M06-2X/6-31G** Derived free energy in solution = -3501.606264

Number of Imaginary Frequencies = 1 (-885.07)

ONIOM (M06-2X /6-31G**:UFF) Geometry

C	-3.73660	-0.77550	0.05960	C	-3.07630	3.44990	0.60710
C	-4.23840	-3.53780	-0.33720	C	-2.46520	2.79770	-0.39520
C	-2.84980	-3.05890	-0.18670	C	-2.61460	1.32130	-0.50010
C	-2.59910	-1.74090	-0.05140	C	-3.43480	0.65560	0.33680
H	-4.42270	-4.59810	-0.46690	H	-3.01430	4.52980	0.67470

C	-5.25660	-2.66960	-0.33170
C	-5.00380	-1.21700	-0.14110
C	-6.17990	-0.31040	-0.20700
C	-7.41270	-0.80270	-0.37740
C	-7.65510	-2.25410	-0.51700
C	-6.64810	-3.13320	-0.50110
H	-6.06160	0.76270	-0.13940
H	-8.25510	-0.12450	-0.42450
H	-8.67010	-2.60770	-0.64480
H	-6.86240	-4.18920	-0.61590
C	-3.83840	2.70690	1.63200
C	-4.00720	1.36930	1.51680
C	-4.71360	0.63380	2.59990
C	-5.20850	1.29390	3.65340
C	-5.04980	2.75740	3.75370
C	-4.39890	3.42850	2.79760
H	-4.82390	-0.44160	2.55560
H	-5.72030	0.75450	4.43990
H	-5.45470	3.28460	4.60780
H	-4.27660	4.50100	2.88360
O	-1.30820	-1.31340	0.13450
O	-1.96440	0.64500	-1.49740
P	-0.70380	-0.23510	-0.95360
O	-0.14420	-0.92610	-2.16210
O	0.22580	0.61930	-0.12090
H	1.23850	-0.08950	0.96510
C	-0.49750	5.04690	-3.51600
C	-1.73820	3.55210	-1.44750
H	-0.03070	5.61400	-4.31170
C	0.35830	-5.93020	-0.16440
C	-1.75600	-4.04920	-0.20440
H	1.17340	-6.64260	-0.13740
C	4.12860	-0.95830	-0.45720
C	3.09740	-1.25310	-1.31780
C	2.71340	1.09140	-1.74720
C	3.76500	1.44220	-0.94040
N	2.40710	-0.22880	-1.89190
H	1.46340	-0.46990	-2.27040
C	4.38880	0.43370	-0.07550
H	3.88610	0.60350	1.06120
H	5.42220	0.64250	0.20460
C	2.64180	-2.60950	-1.78350
H	2.64880	-3.33600	-0.97540
H	1.63650	-2.52190	-2.20050
H	3.32260	-2.97870	-2.55620
C	1.82960	2.01890	-2.52890
H	0.92810	1.49020	-2.84410
H	1.55050	2.87200	-1.91470
H	2.35660	2.39960	-3.40710
C	4.28630	2.80700	-0.76650
C	5.04280	-1.95340	0.14290
C	4.44590	4.99280	-1.56430
H	3.98440	5.59650	-2.34340
H	4.23560	5.40320	-0.57520
H	5.52850	4.95060	-1.70100
C	5.67810	-4.19420	0.37720
H	5.32990	-5.15650	0.00690
H	6.69570	-3.99050	0.03840
H	5.64210	-4.17350	1.46680
O	5.04370	3.11060	0.13400
O	5.97150	-1.63900	0.86010
O	4.77570	-3.22640	-0.17170
O	3.88130	3.68830	-1.69740
C	3.25070	-2.01800	2.79300
C	2.21310	-1.67080	1.99460
N	1.99390	-0.33710	1.65870
C	2.80230	0.65860	2.07140
H	3.16600	2.73100	1.80620
C	1.20740	-2.61410	1.42210
H	1.52570	-3.64750	1.55480
H	0.23880	-2.46180	1.90730
H	1.04580	-2.40930	0.35970
N	3.73100	-4.51450	3.27670
C	3.49410	-3.39960	3.05760
C	4.21390	-1.00520	3.37790
H	4.42940	-1.26540	4.41820
H	5.16100	-1.04200	2.83210
C	3.60440	0.39380	3.32410
H	4.36420	1.17050	3.43940
H	2.88670	0.51200	4.14890

C	2.30180	2.06100	1.84340	C	0.99620	6.22220	-1.80670
H	1.78120	2.09900	0.87960	C	1.41840	6.38150	-0.54890
C	1.33360	2.51130	2.95710	C	0.83610	5.56910	0.53430
C	0.03120	1.71140	3.02750	C	-0.13370	4.68610	0.26810
H	-0.36650	1.58650	2.01390	H	1.44010	6.81130	-2.59960
H	0.23580	0.69840	3.39680	H	2.19900	7.09670	-0.32440
C	-0.99960	2.38260	3.93130	H	1.20210	5.68790	1.54550
H	-0.60960	2.51970	4.94510	H	-0.52910	4.08800	1.07510
H	-1.91270	1.78650	4.00320	C	-0.98250	-4.32430	-1.45050
H	-1.27330	3.37000	3.54630	C	0.03620	-5.21700	-1.41970
C	-2.09940	3.38600	-2.73420	C	0.81650	-5.49470	-2.64740
H	-2.89850	2.70500	-3.00160	C	0.50970	-4.87540	-3.79260
C	-1.44780	4.15660	-3.80860	C	-0.61240	-3.91770	-3.83930
H	-1.75330	4.00790	-4.83630	C	-1.32320	-3.65860	-2.73540
C	-1.46580	-4.72640	0.91920	H	1.63990	-6.19740	-2.62120
C	-0.36350	-5.70750	0.93830	H	1.08240	-5.07620	-4.68870
H	-0.12970	-6.23380	1.85460	H	-0.85950	-3.42590	-4.77120
H	1.09960	3.56400	2.77240	H	-2.14480	-2.95640	-2.79110
H	1.84180	2.49270	3.92980	H	-2.02290	-4.54620	1.83090
C	-0.64510	4.52310	-1.11930				
C	-0.07380	5.24630	-2.11440				

1-naphthyl derived CPA *Type II* TS

(B3LYP/6-31G**:UFF) Energy = -1966.205039

(B3LYP/6-31G**:UFF) Free Energy = -1965.219557

M06-2X/6-31G** Derived free energy = -3501.590113

M06-2X/6-31G** Derived free energy in solution = -3501.609791

Number of Imaginary Frequencies = 1 (-775.60)

ONIOM (B3LYP/6-31G**:UFF) Geometry

C	3.78870	-0.94570	-0.88640	C	3.04560	-2.02120	-0.19100
C	5.31690	1.24850	-1.79140	H	1.17330	-5.03810	1.43810
C	3.97290	1.47740	-1.21100	C	5.79460	0.00560	-1.92750
C	3.19580	0.42080	-0.88880	C	5.00480	-1.15340	-1.44290
H	5.91250	2.09800	-2.10570	C	5.61400	-2.50100	-1.57780
C	1.66370	-4.17160	1.00890	C	6.83330	-2.64590	-2.11050
C	1.10180	-3.51600	-0.02050	C	7.61500	-1.48300	-2.58370
C	1.81380	-2.35380	-0.62650	C	7.13240	-0.23830	-2.50240

H	5.07730	-3.38700	-1.26390	H	-1.96160	0.41900	-3.61800
H	7.25920	-3.63660	-2.20440	H	-3.74850	0.27560	-3.59010
H	8.59810	-1.64740	-3.00590	H	-2.98030	1.67960	-4.32180
H	7.73390	0.58990	-2.85790	C	-5.36190	1.57790	-1.51200
C	2.94290	-3.71720	1.58460	C	-2.31440	4.38570	1.09720
C	3.60640	-2.68130	1.02580	C	-7.02700	0.58040	-2.85910
C	4.84920	-2.18460	1.67840	H	-7.68980	1.43430	-2.70790
C	5.32090	-2.78230	2.77860	H	-7.09260	0.21620	-3.88390
C	4.60810	-3.93540	3.36000	H	-7.28780	-0.21210	-2.15430
C	3.47900	-4.37820	2.79690	C	-1.14050	6.17800	2.08360
H	5.37690	-1.32640	1.28470	H	-2.05380	6.76080	2.22050
H	6.22290	-2.41530	3.25110	H	-0.92140	5.64140	3.01020
H	4.99700	-4.41580	4.24840	H	-0.30750	6.82540	1.81150
H	2.95500	-5.21830	3.23560	O	-6.21290	1.79590	-0.66410
O	1.91880	0.61400	-0.41480	O	-3.09450	4.39880	2.03580
O	1.25260	-1.66300	-1.67430	O	-1.28570	5.25830	0.98810
P	0.70000	-0.16340	-1.25250	O	-5.64920	0.97750	-2.68600
O	0.41920	0.57510	-2.54230	C	-5.19980	-1.15890	0.62830
O	-0.40360	-0.30530	-0.22750	C	-3.97390	-1.16190	0.03660
H	-2.00050	-0.41460	0.08310	N	-2.94370	-0.37570	0.53770
C	-2.49820	-5.24910	-1.63550	C	-3.09250	0.51680	1.55660
C	-0.17880	-4.00900	-0.57830	H	-1.21300	1.49700	1.35430
H	-3.37830	-5.72680	-2.04760	C	-3.64670	-1.91000	-1.22400
C	2.80330	5.59030	-0.64670	H	-2.57980	-2.13480	-1.28330
C	3.52810	2.87210	-0.99530	H	-4.22860	-2.82870	-1.29990
H	2.51700	6.62740	-0.52440	H	-3.90110	-1.29700	-2.09320
C	-2.41860	3.42260	-0.01600	N	-7.13810	-2.53050	-0.40070
C	-1.40710	3.10280	-0.89480	C	-6.25240	-1.92460	0.05820
C	-2.89550	1.69490	-2.19090	C	-5.53080	-0.24640	1.79560
C	-3.94160	1.95600	-1.32440	H	-6.11970	0.59520	1.41490
N	-1.65910	2.20070	-1.89200	H	-6.15850	-0.77310	2.52150
H	-0.83170	1.72100	-2.32480	C	-4.25950	0.25830	2.49200
C	-3.65840	2.63910	-0.05190	H	-3.89530	-0.50400	3.19730
H	-3.52120	1.72380	0.82990	H	-4.46450	1.15760	3.07840
H	-4.52180	3.12750	0.39700	C	-1.81070	1.05810	2.15760
C	-0.00730	3.63690	-0.89430	H	-2.06950	1.85610	2.86010
H	0.36460	3.72170	0.12390	C	-0.93520	-0.02160	2.83280
H	0.64070	2.97210	-1.46600	C	0.48380	0.48400	3.12120
H	0.01950	4.63660	-1.33860	H	0.91220	0.84990	2.18120
C	-2.90850	0.95530	-3.50140	H	0.44990	1.33510	3.81550

C	1.39300	-0.61180	3.68600
H	1.01800	-1.00100	4.63960
H	2.40830	-0.23810	3.85470
H	1.46190	-1.45400	2.98880
C	-0.23060	-4.38220	-1.86950
H	0.63510	-4.27310	-2.51170
C	-1.44090	-5.02130	-2.41970
H	-1.46090	-5.32670	-3.45780
C	3.44410	3.70240	-2.05040
C	3.06080	5.11490	-1.86820
H	2.98650	5.76570	-2.72970
H	-0.85370	-0.86290	2.14400
H	-1.41040	-0.38130	3.75530
C	-1.38550	-4.20360	0.28830
C	-2.48290	-4.81380	-0.22220
C	-3.70180	-4.96770	0.60610
C	-3.73640	-4.47640	1.84920
C	-2.55310	-3.79730	2.40900
C	-1.43850	-3.67640	1.67920
H	-4.57810	-5.45600	0.19850
H	-4.63370	-4.57270	2.44660
H	-2.58900	-3.39750	3.41420
H	-0.58510	-3.17630	2.11340
C	3.25640	3.40290	0.37910
C	2.90150	4.70290	0.53230
C	2.58560	5.23460	1.87790
C	2.66500	4.44040	2.95030
C	3.09130	3.03650	2.80090
C	3.38130	2.54510	1.59060
H	2.28120	6.26690	1.99700
H	2.42760	4.82850	3.93230
H	3.17800	2.40660	3.67680
H	3.71300	1.51880	1.51490
H	3.65430	3.34540	-3.05160

(M06-2X/6-31G**:UFF) Energy = -1965.501761

(M06-2X /6-31G**:UFF) Free Energy = -1964.505969

M06-2X/6-31G** Derived free energy = -3501.587545

M06-2X/6-31G** Derived free energy in solution = -3501.606568

Number of Imaginary Frequencies = 1 (-866.58)

ONIOM (M06-2X /6-31G**:UFF) Geometry

C	3.75840	-1.01740	-0.85080
C	5.34590	1.11410	-1.80700
C	4.02030	1.39510	-1.20780
C	3.21680	0.36890	-0.85660
H	5.96420	1.93900	-2.14220
C	1.53340	-4.15800	1.07710
C	0.97610	-3.46650	0.06940
C	1.72050	-2.32750	-0.54010
C	2.98070	-2.05890	-0.13910
H	1.01690	-5.00870	1.50680
C	5.77710	-0.14680	-1.93830
C	4.95480	-1.27340	-1.42970
C	5.50840	-2.64370	-1.57280
C	6.71000	-2.83690	-2.12990
C	7.52620	-1.70640	-2.62290
C	7.09320	-0.44380	-2.53750
H	4.94390	-3.50760	-1.24620
H	7.09500	-3.84370	-2.22980
H	8.49340	-1.90990	-3.06420
H	7.71840	0.35940	-2.90930
C	2.84760	-3.76920	1.62170
C	3.54290	-2.75810	1.05570
C	4.82340	-2.32550	1.68030
C	5.29760	-2.95860	2.75930

C	4.54980	-4.08650	3.34610
C	3.38630	-4.46980	2.81020
H	5.37760	-1.48560	1.28350
H	6.22760	-2.63850	3.21130
H	4.94110	-4.59630	4.21700
H	2.83700	-5.29130	3.25320
O	1.95420	0.60680	-0.37090
O	1.16900	-1.62210	-1.58140
P	0.71690	-0.09240	-1.21000
O	0.51450	0.61460	-2.52170
O	-0.41360	-0.11320	-0.21510
H	-2.00490	-0.27950	0.03700
C	-2.71130	-5.03710	-1.51420
C	-0.32880	-3.90710	-0.47480
H	-3.61600	-5.47320	-1.91910
C	2.91340	5.53530	-0.72190
C	3.61330	2.80480	-1.01710
H	2.63780	6.57750	-0.61960
C	-2.36530	3.37730	-0.02980
C	-1.33260	3.01830	-0.85580
C	-2.79880	1.64200	-2.19080
C	-3.86340	1.91250	-1.35980
N	-1.57500	2.15420	-1.87860
H	-0.74010	1.69180	-2.31930
C	-3.63680	2.65750	-0.11340
H	-3.58830	1.81540	0.82100
H	-4.50630	3.20310	0.25450
C	0.09670	3.45160	-0.74240
H	0.46970	3.22920	0.25960
H	0.70410	2.91290	-1.47010
H	0.18430	4.52740	-0.89840
C	-2.75120	0.87180	-3.48160
H	-1.90280	0.17970	-3.43690
H	-3.67040	0.34540	-3.70420
H	-2.54430	1.57600	-4.29380
C	-5.27280	1.50740	-1.56860
C	-2.23750	4.25340	1.14560
C	-6.88750	0.45220	-2.89880
H	-7.51430	1.34540	-2.89200
H	-6.91380	-0.03090	-3.87390

H	-7.22220	-0.23590	-2.12110
C	-0.98410	5.83190	2.32390
H	-1.87320	6.44210	2.49110
H	-0.82070	5.19780	3.19850
H	-0.11440	6.45880	2.13820
O	-6.14880	1.78060	-0.77430
O	-3.05310	4.25800	2.04580
O	-1.14170	5.03030	1.15320
O	-5.51520	0.81590	-2.69170
C	-5.22450	-0.95040	0.53810
C	-4.00150	-0.98470	-0.04010
N	-2.95010	-0.25010	0.49110
C	-3.10130	0.60710	1.52320
H	-1.25860	1.62810	1.30760
C	-3.69370	-1.70860	-1.31450
H	-2.62660	-1.93030	-1.39060
H	-4.27720	-2.62590	-1.39060
H	-3.97630	-1.07680	-2.15990
N	-7.15470	-2.27970	-0.55790
C	-6.28430	-1.68840	-0.06810
C	-5.54690	-0.07480	1.72780
H	-6.09890	0.80100	1.37000
H	-6.19910	-0.61080	2.42290
C	-4.26800	0.35050	2.44880
H	-3.92420	-0.45600	3.11370
H	-4.43480	1.23540	3.06740
C	-1.83190	1.15890	2.11350
H	-2.08990	1.92730	2.84810
C	-0.92800	0.07120	2.71640
C	0.51030	0.56370	2.85560
H	0.83710	0.91840	1.87140
H	0.55590	1.41270	3.55020
C	1.44810	-0.54810	3.31380
H	1.16730	-0.93460	4.29870
H	2.48420	-0.20460	3.36700
H	1.40670	-1.38120	2.60600
C	-0.40520	-4.28530	-1.76350
H	0.46090	-4.21950	-2.41120
C	-1.64710	-4.86930	-2.30380
H	-1.68640	-5.18160	-3.33930

C	3.53010	3.61300	-2.08910	H	-2.67300	-3.18960	3.53890
C	3.16060	5.03240	-1.93440	H	-0.66500	-3.08310	2.23000
H	3.08780	5.66590	-2.80890	C	3.35490	3.36670	0.34730
H	-0.91870	-0.77590	2.02950	C	3.00940	4.67170	0.47480
H	-1.32190	-0.27140	3.68190	C	2.71170	5.23550	1.81120
C	-1.53770	-4.03990	0.39960	C	2.78920	4.46300	2.89950
C	-2.66900	-4.59020	-0.10490	C	3.19880	3.05160	2.77680
C	-3.89160	-4.67310	0.72790	C	3.47860	2.53300	1.57550
C	-3.89190	-4.18430	1.97240	H	2.42460	6.27460	1.91090
C	-2.66690	-3.58180	2.53010	H	2.56440	4.87470	3.87490
C	-1.55060	-3.52330	1.79510	H	3.28360	2.43880	3.66500
H	-4.79700	-5.10750	0.32270	H	3.80030	1.50230	1.51890
H	-4.79070	-4.22900	2.57350	H	3.72540	3.23160	-3.08430

	ONIOM (B3LYP/6-31G**:UFF)		ONIOM (M06-2X/6-31G**:UFF)	
**Mechanism** (Catalyst)	$\Delta\Delta G^{\ddagger}$	$\Delta\Delta G_{sol}{}^{\ddagger}$	$\Delta\Delta G^{\ddagger}$	$\Delta\Delta G_{sol}{}^{\ddagger}$
Type I (Ph)	0	0	0	0
Type II (Ph)	-3.5	-3.9	-0.7	-1.6
Type I (9-anthryl)	0	0	0	0
Type II (9-anthryl)	+3.5	+3.6	+3.6	+3.9
Type I (9-phenanthryl)	0	0	0	0
Type II (9-phenanthryl)	+0.6	+1.0	+1.1	+0.8
Type I (1-naphthyl)	0	0	0	0
Type II (1-naphthyl)	0	+0.8	+0.5	-0.2

Table 1. Comparison of the relative energies derived from both ONIOM methods. All energies in kcal mol^{-1}.[21]

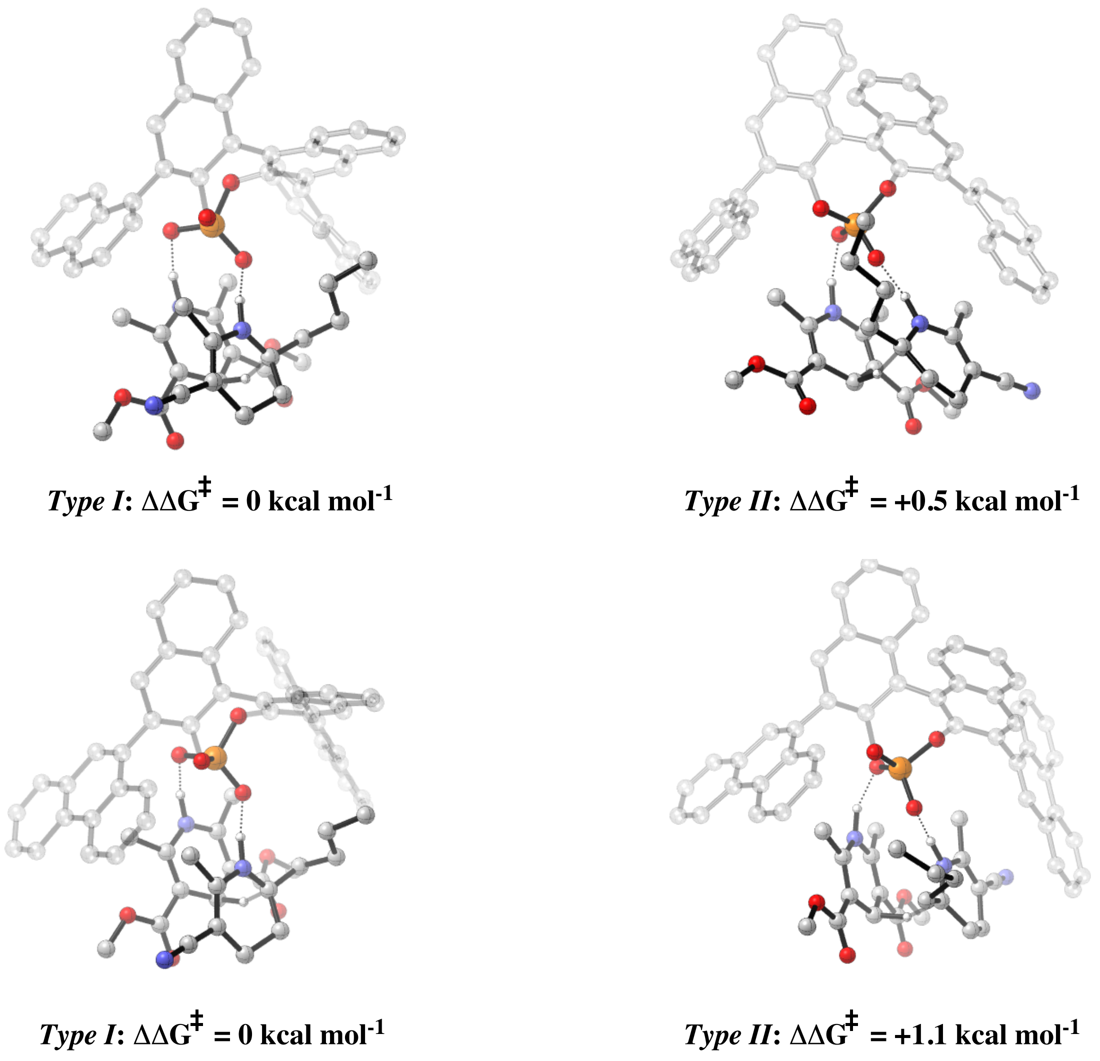

Figure 1. Competing TSs for the 9-phenanthryl and 1-naphthyl derived phosphoric acid catalyzed transfer hydrogenation reaction of cyclic imines. ONIOM (M06-2X/6-31G**: UFF), single-point energy M06-2X/6-31G**. Grayed-out regions were treated with UFF, and the full-color regions were treated M06-2X/6-31G**.[22]

Friedel-Crafts Study

TS5-*E*

(B3LYP/6-31G**:UFF) Energy = -1525.488482

(B3LYP/6-31G**:UFF) Free Energy = -1524.263670

M06-2X/6-31G** Derived free energy = -3461.015501

M06-2X/6-31G** Derived free energy in solution = -3461.039551

Number of Imaginary Frequencies = 1 (-262.04)

ONIOM (B3LYP/6-31G**:UFF) Geometry

C	2.01210	2.49010	0.49440
C	4.45030	1.41190	1.44480
C	3.72080	0.72370	0.35740
C	2.53790	1.21460	-0.06670
H	5.40890	1.02620	1.76800
C	-1.55100	4.29860	-1.12490
C	-1.57310	3.01610	-0.73060
C	-0.32270	2.37240	-0.24430
C	0.81580	3.09480	-0.14980
H	-2.46610	4.79110	-1.42940
C	3.92410	2.49300	2.03450
C	2.63530	3.05650	1.55470
C	2.09600	4.23720	2.27600
C	2.77930	4.78760	3.28660
C	4.08280	4.24530	3.72670
C	4.62270	3.16860	3.14550
H	1.13580	4.66000	2.01030
H	2.36850	5.64660	3.80160
H	4.60440	4.72880	4.54270
H	5.57620	2.79480	3.49930
C	0.86040	4.47440	-0.72420
C	-0.28150	5.04890	-1.16550
C	-0.25840	6.42350	-1.71710
C	0.89600	7.09250	-1.80610
C	2.15330	6.45930	-1.36530
C	2.14180	5.22050	-0.85970
H	-1.17570	6.89020	-2.05380
H	0.91270	8.09590	-2.21160
H	3.08760	6.99710	-1.46210
H	3.07940	4.76800	-0.56590
P	0.42200	-0.04260	-0.65230
O	1.86320	0.60250	-1.09210
O	-0.37330	1.09860	0.26380
O	-0.34240	-0.26330	-1.94010
O	0.61140	-1.19740	0.30070
H	-0.33110	-2.61640	0.63660
C	-1.93290	-3.45360	1.66360
N	-0.84590	-3.50180	0.80130
C	-1.47960	-0.89400	5.14090
C	-0.44830	-1.00430	4.21120
C	-0.59650	-1.79610	3.07220
C	-1.78340	-2.51210	2.84340
C	-2.82240	-2.37640	3.78370
C	-2.67490	-1.57870	4.91470
H	-1.36130	-0.27420	6.02460
H	0.48300	-0.46580	4.35840
H	0.20960	-1.81760	2.35380
H	-3.76400	-2.89640	3.64400
H	-3.49590	-1.49470	5.62030
C	-4.54710	-4.46250	-0.84780
C	-4.55030	-4.97490	-2.14510
C	-3.78820	-4.38290	-3.16530
C	-3.01500	-3.24800	-2.92260
H	-5.14440	-4.92880	-0.07030

H	-5.15320	-5.84870	-2.37210
H	-3.80920	-4.81020	-4.16280
H	-2.43520	-2.77160	-3.70610
C	-2.74560	-1.42200	0.14460
C	-3.48600	-2.54740	0.62520
C	-3.76780	-3.33420	-0.57780
C	-3.03270	-2.74100	-1.62660
H	-2.33400	-0.59660	0.70650
H	-4.17220	-2.46650	1.45700
N	-2.41380	-1.58620	-1.12430
H	-1.61740	-1.01300	-1.58740
C	-0.43530	-4.56260	-0.02140
O	-0.96200	-5.66190	-0.03940
C	0.77720	-4.24160	-0.87750
H	0.58120	-4.60350	-1.88940
H	1.03210	-3.18170	-0.89320
H	1.63010	-4.80970	-0.49050
C	-5.39610	0.98290	-0.57440
C	-4.79670	1.19780	-1.75340
C	-3.46770	1.85850	-1.84170
C	-2.86240	2.28290	-0.69370
C	-3.54350	2.07680	0.63150
C	-4.72310	1.42750	0.66510
H	-5.30050	0.90480	-2.66460
H	-5.22580	1.26680	1.60890
C	5.43910	-2.87230	-1.34790
C	5.39660	-1.74000	-2.06200
C	4.83720	-0.48500	-1.49500
C	4.30410	-0.50440	-0.23950
C	4.35690	-1.77140	0.57450
C	4.92330	-2.86810	0.03660
H	5.78200	-1.73440	-3.07330
H	5.00270	-3.77760	0.61530
C	-2.87400	2.09700	-3.23280
H	-1.82020	2.43750	-3.16190
C	-2.96920	2.65240	1.92740
H	-2.02090	3.19060	1.73240
C	-6.77680	0.35180	-0.51860
H	-7.13600	0.13040	-1.54900
C	-2.63550	1.54960	2.93850
H	-1.89340	0.85170	2.50290
H	-2.18680	1.99300	3.85290
H	-3.54030	0.97780	3.23280
C	-3.91790	3.69080	2.54110
H	-4.86150	3.22100	2.89010
H	-3.43100	4.18420	3.40930
H	-4.16080	4.47300	1.79020
C	-7.79410	1.31260	0.10860
H	-7.56360	1.50170	1.17830
H	-7.79030	2.28050	-0.43710
H	-8.81570	0.88130	0.04080
C	-6.74360	-0.98090	0.23510
H	-6.38990	-0.84630	1.27920
H	-7.75850	-1.43190	0.25970
H	-6.07030	-1.68850	-0.28950
C	-3.65770	3.19310	-3.96490
H	-3.66620	4.12830	-3.36820
H	-3.18160	3.41330	-4.94420
H	-4.70820	2.87890	-4.14330
C	-2.84340	0.81670	-4.08260
H	-2.24560	0.99050	-5.00270
H	-2.37540	-0.01450	-3.52490
H	-3.86210	0.50400	-4.39240
C	6.00090	-4.14490	-1.95490
H	6.32720	-3.95480	-3.00240
C	3.82400	-1.83450	2.00650
H	3.24440	-0.91850	2.24010
C	4.93030	0.78580	-2.34260
H	4.50000	1.66200	-1.81800
C	6.39410	1.14910	-2.62050
H	6.94810	1.24910	-1.66250
H	6.89220	0.37740	-3.24440
H	6.44970	2.12130	-3.15560
C	4.14530	0.63770	-3.65250
H	4.60670	-0.12150	-4.31800
H	3.09770	0.33680	-3.44150
H	4.12390	1.60820	-4.19280
C	7.23570	-4.62260	-1.18220
H	7.99650	-3.81370	-1.14360
H	6.97250	-4.91780	-0.14460

H	7.68770	-5.49930	-1.69350	C	4.97900	-1.91190	3.01280
C	4.92920	-5.24020	-2.01250	H	5.55110	-2.85620	2.88960
H	4.61140	-5.54620	-0.99320	H	5.67370	-1.05860	2.87190
H	4.04020	-4.87380	-2.56950	H	4.58350	-1.86890	4.05010
H	5.32640	-6.13300	-2.54090	C	-2.59320	-4.77770	2.00450
C	2.85670	-3.01010	2.21680	H	-3.46430	-4.62270	2.64030
H	2.09920	-3.03320	1.41070	H	-2.86460	-5.34060	1.11780
H	3.38960	-3.98390	2.22590	H	-1.86920	-5.38120	2.56320
H	2.33320	-2.89940	3.19030				

TS5-*Z*

(B3LYP/6-31G**:UFF) Energy = -1525.477503

(B3LYP/6-31G**:UFF) Free Energy = -1524.254519

M06-2X/6-31G** Derived free energy = -3461.010619

M06-2X/6-31G** Derived free energy in solution = -3461.034457

Number of Imaginary Frequencies = 1 (-256.31)

ONIOM (B3LYP/6-31G**:UFF) Geometry

C	-3.42340	-0.90320	0.78580	C	-3.75330	-3.22170	-0.35770
C	-4.59100	1.53520	1.63500	C	-3.22050	-4.41210	-0.72000
C	-3.52240	1.55390	0.61260	C	-4.08970	-5.48180	-1.26200
C	-2.94670	0.39670	0.22880	C	-5.40000	-5.26720	-1.42090
H	-5.07260	2.46290	1.91680	C	-5.98310	-3.95870	-1.06870
C	-1.76440	-4.63530	-0.61770	C	-5.20760	-2.98750	-0.57300
C	-0.95050	-3.64160	-0.23230	H	-3.66650	-6.44040	-1.53520
C	-1.52760	-2.33880	0.18710	H	-6.03420	-6.04870	-1.81900
C	-2.86650	-2.16100	0.21220	H	-7.04000	-3.78330	-1.22310
H	-1.35430	-5.59930	-0.89220	H	-5.65270	-2.02820	-0.34570
C	-4.95010	0.38410	2.21750	P	-0.49910	-0.05360	-0.25620
C	-4.32490	-0.89780	1.79750	O	-1.99290	0.40170	-0.75600
C	-4.73100	-2.12480	2.52960	O	-0.69730	-1.38210	0.70790
C	-5.66720	-2.06980	3.48440	O	0.22890	-0.42810	-1.53200
C	-6.32310	-0.79730	3.85420	O	0.11700	0.95560	0.68160
C	-5.98840	0.35630	3.26640	H	1.53520	1.78280	0.52220
H	-4.26540	-3.07840	2.31850	C	3.38140	1.78690	1.39850
H	-5.94850	-2.97510	4.00710	N	2.48290	2.22090	0.44950
H	-7.08300	-0.80240	4.62500	C	6.66910	4.28140	2.69160
H	-6.48320	1.27120	3.57020	C	5.56680	4.83470	2.04140

C	4.53940	4.01960	1.57130
C	4.58900	2.62620	1.74050
C	5.69010	2.08770	2.42570
C	6.72360	2.90150	2.88570
H	7.47260	4.91790	3.04980
H	5.50120	5.90860	1.89500
H	3.69030	4.47350	1.07980
H	5.75540	1.02200	2.60970
H	7.56780	2.45330	3.40100
C	6.06670	0.81180	-1.03440
C	6.28050	1.03980	-2.39200
C	5.28510	0.76840	-3.34560
C	4.05110	0.24090	-2.97080
H	6.83700	1.04320	-0.30620
H	7.23310	1.44300	-2.72160
H	5.48300	0.96770	-4.39420
H	3.27910	0.01760	-3.69980
C	2.99460	-0.66300	0.33100
C	4.22590	-0.02450	0.65990
C	4.83250	0.29420	-0.63460
C	3.85660	0.00360	-1.61270
H	2.24310	-1.06700	0.99210
H	4.79080	-0.31320	1.53430
N	2.74590	-0.55930	-0.96540
H	1.73830	-0.63300	-1.33880
C	2.69950	2.91330	-0.75770
O	3.78050	3.34130	-1.11420
C	1.42490	3.08100	-1.56390
H	0.62820	3.52250	-0.95760
H	1.05820	2.10750	-1.90620
H	1.64100	3.71730	-2.42140
C	3.32920	-4.33050	-0.20660
C	2.65920	-4.19600	0.94750
C	1.18890	-3.97880	0.97170
C	0.51960	-3.84910	-0.20960
C	1.27120	-3.96540	-1.51180
C	2.59310	-4.22120	-1.48080
H	3.19390	-4.27540	1.88400
H	3.14460	-4.35110	-2.40230
C	-2.28070	5.31080	-1.16060

C	-2.00600	5.04990	0.12420
C	-2.39840	3.76330	0.75680
C	-3.10030	2.85080	0.02330
C	-3.46480	3.16200	-1.40350
C	-3.04350	4.31920	-1.94760
H	-1.47350	5.78500	0.71310
H	-3.29050	4.55690	-2.97330
C	-4.33690	2.22110	-2.23540
H	-4.61330	1.32220	-1.65060
C	-1.98920	3.52860	2.21270
H	-2.16560	2.47490	2.51160
C	-1.83030	6.61460	-1.79400
H	-1.27960	7.23010	-1.04710
C	-0.86340	6.35170	-2.95410
H	0.00440	5.75730	-2.59740
H	-1.36360	5.79860	-3.77710
H	-0.48160	7.31410	-3.35670
C	-3.03300	7.44500	-2.25690
H	-3.58490	6.93390	-3.07390
H	-3.72710	7.61580	-1.40630
H	-2.69060	8.43410	-2.62910
C	-0.48960	3.78250	2.43520
H	0.10780	3.31310	1.63180
H	-0.25580	4.86770	2.45100
H	-0.17170	3.34850	3.40730
C	-2.81950	4.41240	3.15090
H	-2.62920	5.48930	2.95510
H	-3.90220	4.21560	3.01120
H	-2.56150	4.19240	4.20890
C	-3.59310	1.71820	-3.48080
H	-3.40480	2.54310	-4.19960
H	-2.61990	1.26820	-3.19280
H	-4.19650	0.93880	-3.99320
C	-5.66330	2.88950	-2.62040
H	-5.50040	3.74930	-3.30400
H	-6.32400	2.15650	-3.13100
H	-6.18520	3.24950	-1.70790
C	0.48620	-3.99570	2.33170
H	-0.60670	-3.84260	2.23000
C	0.99560	-2.87080	3.24350

H	0.42660	-2.86980	4.19760
H	2.07270	-2.99400	3.48080
H	0.84650	-1.88420	2.76010
C	0.65300	-5.36150	3.00900
H	1.71580	-5.56120	3.26130
H	0.05780	-5.39690	3.94650
H	0.28590	-6.16580	2.33590
C	0.56130	-3.86340	-2.86330
H	-0.47620	-3.49860	-2.72410
C	1.23390	-2.85550	-3.81010
H	1.38220	-1.88270	-3.30520
H	2.21850	-3.22250	-4.16750
H	0.58880	-2.68560	-4.69850
C	0.47100	-5.24120	-3.53160
H	1.48170	-5.62890	-3.78130
H	-0.02870	-5.96640	-2.85660
H	-0.12420	-5.17290	-4.46710
C	4.82220	-4.60730	-0.22980
H	5.15330	-4.72950	-1.28490
C	5.61560	-3.43150	0.35040
H	5.41540	-2.51700	-0.24450
H	5.34170	-3.24290	1.41010
H	6.70500	-3.64200	0.29730
C	5.16120	-5.91610	0.49540
H	4.95670	-5.83810	1.58410
H	4.56080	-6.75100	0.07480
H	6.23650	-6.15880	0.35730
C	2.67360	1.26070	2.64530
H	3.33680	0.63530	3.24580
H	2.39310	2.12490	3.25860
H	1.76050	0.71980	2.39180

TS6-*E*

(B3LYP/6-31G**:UFF) Energy = -1525.484905

(B3LYP/6-31G**:UFF) Free Energy = -1524.260521

M06-2X/6-31G** Derived free energy = -3461.013360

M06-2X/6-31G** Derived free energy in solution = -3461.036811

Number of Imaginary Frequencies = 1 (-256.19)

ONIOM (B3LYP/6-31G**:UFF) Geometry

C	2.29290	2.27340	0.63190
C	4.60420	0.97040	1.61440
C	3.79980	0.33310	0.54910
C	2.66370	0.92260	0.12680
H	5.51420	0.48990	1.95270
C	-1.05290	4.40390	-1.05520
C	-1.22290	3.16690	-0.56500
C	-0.04910	2.40110	-0.07060
C	1.16960	2.98130	-0.03620
H	-1.90810	4.98230	-1.38290
C	4.20630	2.12590	2.16270
C	2.98950	2.81460	1.65890
C	2.60040	4.08560	2.32080
C	3.34780	4.59550	3.30700
C	4.57640	3.91950	3.77700
C	4.98230	2.76100	3.24610
H	1.69880	4.60980	2.03120
H	3.04600	5.52140	3.77960
H	5.15350	4.37260	4.57280
H	5.88480	2.29180	3.61940
C	1.37040	4.30720	-0.69590
C	0.29840	4.98720	-1.16330
C	0.47830	6.30990	-1.80490
C	1.70350	6.82640	-1.94750

C	2.88230	6.07550	-1.47500
C	2.72980	4.88250	-0.88860
H	-0.38150	6.86190	-2.16390
H	1.83450	7.79210	-2.41860
H	3.87280	6.48940	-1.61330
H	3.61010	4.34000	-0.57090
P	0.40270	-0.11850	-0.30420
O	1.89180	0.32960	-0.84020
O	-0.24630	1.18020	0.52030
O	-0.41670	-0.38950	-1.54460
O	0.49960	-1.16220	0.78270
H	-0.44590	-2.32860	1.92000
C	-2.26360	-3.32190	2.05210
N	-1.20890	-2.55980	2.56710
C	-1.22250	-5.88620	-1.26980
C	-2.21490	-6.26370	-0.36420
C	-2.54950	-5.43060	0.69910
C	-1.89520	-4.19760	0.89120
C	-0.90060	-3.82780	-0.03470
C	-0.57010	-4.66670	-1.09760
H	-0.96270	-6.53700	-2.09950
H	-2.73160	-7.21150	-0.48200
H	-3.31840	-5.75980	1.38590
H	-0.37120	-2.88520	0.05460
H	0.20180	-4.35290	-1.79350
C	-5.48410	-3.43460	0.07980
C	-5.92550	-3.83770	-1.18220
C	-5.28580	-3.39970	-2.35130
C	-4.18900	-2.53860	-2.29420
H	-5.99990	-3.76910	0.97510
H	-6.78530	-4.49580	-1.26190
H	-5.65530	-3.72800	-3.31770
H	-3.69500	-2.18650	-3.19350
C	-2.73080	-1.08110	0.62990
C	-4.38860	-2.57270	0.16220
C	-3.76700	-2.13760	-1.03220
H	-1.99570	-0.43290	1.08010
N	-2.74290	-1.24220	-0.68140
H	-1.90720	-0.88190	-1.24090
C	-1.29510	-1.64540	3.62570

O	-2.32790	-1.41990	4.24080
C	0.01770	-0.95910	3.93570
H	0.70350	-1.66960	4.41070
H	0.49000	-0.60070	3.01610
H	-0.17550	-0.13590	4.62310
C	-5.29830	1.72940	-0.30870
C	-4.63370	2.07550	0.80220
C	-3.23080	2.56510	0.74760
C	-2.58990	2.60290	-0.45550
C	-3.31190	2.16770	-1.70390
C	-4.59510	1.77060	-1.60880
H	-5.14380	2.04830	1.75620
H	-5.14460	1.49400	-2.49760
C	5.36800	-3.28010	-1.24830
C	4.81020	-3.32190	-0.03080
C	4.21530	-2.11060	0.59270
C	4.29130	-0.92140	-0.06870
C	4.94240	-0.85800	-1.42300
C	5.42290	-1.99060	-1.96960
H	4.78840	-4.25780	0.51200
H	5.88590	-1.96650	-2.94670
C	5.94960	-4.53600	-1.87150
H	5.81760	-5.39950	-1.18100
C	5.09100	0.46080	-2.18270
H	4.68220	1.30410	-1.59280
C	3.54050	-2.26120	1.95620
H	2.98400	-1.34030	2.22860
C	-2.65250	2.25070	-3.08200
H	-1.56910	2.46190	-2.97520
C	-2.61020	3.10590	2.03930
H	-1.58040	3.48090	1.87530
C	-6.76870	1.35430	-0.23950
H	-7.13280	1.43440	0.80970
C	-7.62000	2.31560	-1.07770
H	-7.42970	3.36460	-0.76480
H	-7.39290	2.21590	-2.16010
H	-8.69890	2.09940	-0.92460
C	-6.98870	-0.09600	-0.67640
H	-6.62600	-0.26880	-1.71180
H	-6.45050	-0.77180	0.01620

H	-8.06870	-0.35170	-0.63020	H	4.09030	-2.55220	4.04460
C	-3.25720	3.39820	-3.90080	C	4.30460	0.43620	-3.49960
H	-4.32880	3.20710	-4.12260	H	3.24130	0.18200	-3.30350
H	-3.17590	4.35460	-3.34370	H	4.33790	1.43720	-3.98050
H	-2.71070	3.51070	-4.86160	H	4.72660	-0.30780	-4.20760
C	-2.74640	0.93150	-3.86500	C	6.56680	0.80380	-2.42600
H	-3.79150	0.70060	-4.15850	H	7.04440	0.07660	-3.11580
H	-2.13710	0.99950	-4.79150	H	6.65210	1.81720	-2.87340
H	-2.35310	0.09230	-3.26300	H	7.11990	0.80300	-1.46230
C	-3.40860	4.30510	2.56570	C	5.21900	-4.88940	-3.17210
H	-3.49600	5.07960	1.77380	H	4.12940	-4.99250	-2.98000
H	-4.42720	4.00390	2.88880	H	5.37480	-4.10810	-3.94590
H	-2.88600	4.75780	3.43540	H	5.59450	-5.85710	-3.56790
C	-2.49850	2.01260	3.10870	C	7.45610	-4.38200	-2.11080
H	-3.49280	1.59790	3.37560	H	7.66570	-3.59030	-2.86090
H	-1.85110	1.19120	2.74400	H	7.96720	-4.12000	-1.15970
H	-2.03180	2.42510	4.02860	H	7.88170	-5.33920	-2.48080
C	2.50630	-3.39850	1.95000	C	-3.64890	-1.96370	1.27090
H	1.86930	-3.33890	1.04460	H	-4.07930	-1.71460	2.23250
H	2.99540	-4.39490	1.96560	C	-3.16620	-3.95190	3.10250
H	1.85470	-3.32460	2.84600	H	-3.39310	-3.24120	3.89130
C	4.58600	-2.49560	3.05190	H	-4.09390	-4.31630	2.66170
H	5.14150	-3.44150	2.87700	H	-2.63410	-4.80500	3.53990
H	5.31250	-1.65710	3.07580				

TS6-*Z*

(B3LYP/6-31G**:UFF) Energy = -1525.478484

(B3LYP/6-31G**:UFF) Free Energy = -1524.255388

M06-2X/6-31G** Derived free energy = -3461.009308

M06-2X/6-31G** Derived free energy in solution = -3461.033004

Number of Imaginary Frequencies = 1 (-256.49)

ONIOM (B3LYP/6-31G**:UFF) Geometry

C	-2.60170	-1.79140	0.97900	C	0.22100	-4.74850	-0.38810
C	-4.59600	0.05280	1.76830	C	0.60120	-3.46790	-0.26180
C	-3.84140	0.28110	0.51610	C	-0.39340	-2.44000	0.14400
C	-2.85340	-0.56810	0.16820	C	-1.66180	-2.80410	0.43180
H	-5.39370	0.73320	2.03940	H	0.94930	-5.51140	-0.63390

C	-4.28830	-0.97840	2.56560
C	-3.23480	-1.94690	2.16510
C	-2.93350	-3.05630	3.10470
C	-3.62000	-3.18600	4.24610
C	-4.69240	-2.23770	4.61750
C	-5.00850	-1.20020	3.83480
H	-2.14410	-3.76560	2.89200
H	-3.38370	-3.99960	4.92000
H	-5.22640	-2.38650	5.54720
H	-5.79620	-0.52250	4.14190
C	-2.10570	-4.20470	0.15810
C	-1.19280	-5.13170	-0.21090
C	-1.61210	-6.52940	-0.46490
C	-2.89920	-6.87530	-0.35530
C	-3.90850	-5.86280	0.00870
C	-3.53880	-4.59890	0.24590
H	-0.87830	-7.27540	-0.74360
H	-3.20530	-7.89630	-0.54320
H	-4.95160	-6.14420	0.07350
H	-4.29970	-3.86910	0.48810
P	-0.54710	-0.00520	-0.67900
O	-2.12470	-0.35870	-0.97610
O	0.02520	-1.15820	0.39450
O	0.18950	-0.14970	-1.98700
O	-0.42980	1.26560	0.12730
H	0.54170	2.27960	1.10940
C	2.30910	3.24710	0.80740
N	1.34960	2.66360	1.63690
C	4.83120	6.40590	2.33800
C	3.94170	5.74970	3.18810
C	3.15040	4.70440	2.71680
C	3.22810	4.28960	1.37620
C	4.11370	4.97750	0.52510
C	4.91120	6.01490	1.00110
H	5.44810	7.21820	2.71030
H	3.85650	6.05190	4.22750
H	2.46950	4.21980	3.39970
H	4.18730	4.70200	-0.51850
H	5.58780	6.52420	0.32140
C	5.63200	2.55800	-1.15260
C	6.10290	2.54310	-2.46690
C	5.40640	1.87190	-3.48390
C	4.22490	1.18060	-3.21310
H	6.18840	3.06700	-0.37260
H	7.03210	3.05130	-2.70600
H	5.80140	1.87620	-4.49490
H	3.69320	0.63700	-3.98690
C	2.66610	0.73430	-0.02380
C	3.66360	1.67360	0.36700
C	4.43930	1.88870	-0.85840
C	3.77160	1.19580	-1.89850
H	1.87980	0.29990	0.57050
H	4.07620	1.67910	1.36820
N	2.67920	0.52250	-1.33120
H	1.80600	0.13460	-1.79150
C	1.56600	1.97290	2.83230
O	2.66140	1.87020	3.36400
C	0.29900	1.35200	3.38680
H	-0.41320	2.13640	3.66460
H	-0.18370	0.73140	2.62510
H	0.55100	0.75730	4.26420
C	-5.06350	3.59170	-1.99400
C	-4.39560	3.82180	-0.85390
C	-3.91950	2.70660	0.00540
C	-4.21700	1.42430	-0.35040
C	-4.99870	1.15900	-1.60840
C	-5.36650	2.19950	-2.38040
H	-4.19330	4.84050	-0.55340
H	-5.92300	2.02950	-3.29300
C	4.81300	-2.57750	-0.72870
C	4.04800	-2.79250	-1.80760
C	2.59020	-3.05800	-1.68680
C	2.02760	-3.10520	-0.44380
C	2.88110	-2.88050	0.77410
C	4.18850	-2.60190	0.61160
H	4.50650	-2.80770	-2.78720
H	4.82050	-2.44810	1.47540
C	2.32410	-3.05640	2.18830
H	1.24350	-3.29780	2.15800
C	1.80320	-3.35180	-2.96700

H	0.71190	-3.37330	-2.76640
C	6.31290	-2.38870	-0.87620
H	6.59840	-2.43400	-1.95150
C	-3.11520	3.05860	1.25760
H	-2.66640	2.15020	1.71100
C	-5.41050	-0.25640	-2.01540
H	-5.06720	-0.99730	-1.26750
C	-5.52730	4.73090	-2.88370
H	-6.05540	4.31000	-3.76790
C	-4.33820	5.53920	-3.41770
H	-3.80450	6.06040	-2.59490
H	-3.62430	4.86610	-3.93920
H	-4.69230	6.30010	-4.14550
C	-6.52890	5.63580	-2.15530
H	-6.04930	6.16260	-1.30320
H	-6.93290	6.39660	-2.85680
H	-7.37860	5.03130	-1.77190
C	-4.76960	-0.66210	-3.34880
H	-3.66650	-0.54410	-3.29310
H	-4.99280	-1.72800	-3.56860
H	-5.15440	-0.04290	-4.18630
C	-6.93730	-0.40130	-2.06930
H	-7.37540	0.21700	-2.88070
H	-7.21160	-1.46240	-2.25200
H	-7.38190	-0.09070	-1.09960
C	-4.02150	3.69270	2.31870
H	-4.45300	4.64930	1.95410
H	-4.85340	3.00550	2.57650
H	-3.44040	3.89420	3.24400
C	-1.94130	3.99610	0.93120
H	-2.28850	5.03160	0.73260

H	-1.23680	4.03250	1.78930
H	-1.39230	3.63710	0.03680
C	2.00950	-2.27180	-4.04050
H	3.03710	-2.29780	-4.45840
H	1.29670	-2.43290	-4.87740
H	1.82360	-1.26440	-3.62560
C	2.18930	-4.72700	-3.52520
H	3.25820	-4.74770	-3.82730
H	2.02270	-5.51540	-2.76280
H	1.56500	-4.96780	-4.41210
C	7.07790	-3.51150	-0.16550
H	6.73750	-4.50040	-0.54060
H	6.92320	-3.47110	0.93340
H	8.16580	-3.41920	-0.37100
C	6.74970	-1.01560	-0.35910
H	6.24060	-0.22370	-0.94140
H	7.84490	-0.88680	-0.49250
H	6.50540	-0.89010	0.71690
C	2.44990	-1.77170	3.01610
H	1.91130	-0.94720	2.51210
H	1.98860	-1.91590	4.01630
H	3.51080	-1.47780	3.15770
C	2.99680	-4.23610	2.90240
H	4.07370	-4.03650	3.08580
H	2.50300	-4.42080	3.88040
H	2.90080	-5.15630	2.28700
C	1.70340	3.60130	-0.54060
H	2.46520	3.78090	-1.29760
H	1.02030	2.82300	-0.88320
H	1.12930	4.52690	-0.41720

TS7-*E*

(B3LYP/6-31G**:UFF) Energy = -1525.519109

(B3LYP/6-31G**:UFF) Free Energy = -1524.434210

M06-2X/6-31G** Derived free energy = -4258.982253

M06-2X/6-31G** Derived free energy in solution = -4259.011949

Number of Imaginary Frequencies = 1 (-265.54)

ONIOM (B3LYP/6-31G**:UFF) Geometry

C	-1.06350	-2.92870	0.44500
C	-3.68510	-2.48630	1.41070
C	-3.17890	-1.68440	0.27430
C	-1.90500	-1.85330	-0.13610
H	-4.69180	-2.32020	1.77570
C	2.88140	-3.72790	-1.08820
C	2.59520	-2.52040	-0.57960
C	1.19080	-2.19360	-0.20020
C	0.26200	-3.17880	-0.17840
H	3.89340	-3.98150	-1.37620
C	-2.89900	-3.39500	2.00410
C	-1.51940	-3.63330	1.50590
C	-0.69650	-4.63890	2.22370
C	-1.20360	-5.31740	3.25990
C	-2.58730	-5.09210	3.73130
C	-3.38510	-4.19040	3.14880
H	0.32890	-4.82790	1.93360
H	-0.58740	-6.04470	3.77280
H	-2.95650	-5.66770	4.57040
H	-4.39160	-4.04910	3.52450
C	0.57220	-4.49680	-0.80900
C	1.82650	-4.74680	-1.24570
C	2.15110	-6.04990	-1.87110
C	1.19940	-6.97630	-2.02680
C	-0.17990	-6.70110	-1.58200
C	-0.48160	-5.52930	-1.01080
H	3.16020	-6.25670	-2.20550
H	1.43700	-7.92760	-2.48520
H	-0.94950	-7.44840	-1.72690
H	-1.50390	-5.34270	-0.70980
P	-0.21890	-0.03050	-0.51360
O	-1.35990	-1.05460	-1.10420
O	0.90310	-0.95190	0.31610
O	0.39780	0.61730	-1.73050
O	-0.77970	0.85000	0.58070
H	-0.63410	2.26820	1.79040
Si	-4.32460	-0.43110	-0.50280
Si	3.98290	-1.31860	-0.22860
C	4.10670	2.02360	-3.52160
C	3.21040	1.03450	-3.60640
C	3.17170	-0.02120	-2.57290
C	4.01380	0.02120	-1.52680
C	5.03250	1.09930	-1.46340
C	5.06730	2.05310	-2.40180
H	4.13460	2.79900	-4.27610
H	2.50780	1.00750	-4.42910
H	2.45040	-0.82000	-2.67840
H	5.77600	1.10410	-0.67740
H	5.81240	2.83650	-2.35320
C	3.39360	0.18800	4.17610
C	3.87610	1.02760	3.25370
C	4.05200	0.57550	1.85840
C	3.73100	-0.68060	1.50620
C	3.18840	-1.60320	2.53560
C	3.02680	-1.18990	3.79770
H	3.26260	0.51910	5.19810
H	4.13870	2.04130	3.52710
H	4.43770	1.28070	1.14220
H	2.91210	-2.61610	2.27180
H	2.62570	-1.86400	4.54340
C	8.03280	-3.68170	-0.22620
C	7.81020	-2.75330	-1.16350
C	6.53820	-2.00100	-1.17490
C	5.60290	-2.23760	-0.23840
C	5.85270	-3.27630	0.79090
C	7.00450	-3.95820	0.79630
H	8.96340	-4.23410	-0.21790
H	8.56040	-2.55210	-1.91720
H	6.37850	-1.27340	-1.95780
H	5.10330	-3.49660	1.53860
H	7.18310	-4.71690	1.54730
C	-4.15330	3.59580	1.89440
C	-4.76090	3.54950	0.70380
C	-4.82080	2.27890	-0.04750
C	-4.26200	1.16510	0.45690
C	-3.59940	1.22070	1.78250

C	-3.55120	2.37370	2.46250
H	-4.09960	4.52760	2.44220
H	-5.20060	4.44450	0.28330
H	-5.31310	2.26370	-1.01190
H	-3.15260	0.33340	2.21060
H	-3.06990	2.41610	3.43010
C	-2.99200	0.30640	-4.94050
C	-2.62240	1.15940	-3.97890
C	-3.05510	0.93360	-2.58530
C	-3.82730	-0.12060	-2.27310
C	-4.22520	-1.06740	-3.34320
C	-3.83060	-0.86230	-4.60640
H	-2.67360	0.46720	-5.96230
H	-2.00000	2.01230	-4.21700
H	-2.70490	1.60650	-1.81790
H	-4.83140	-1.93140	-3.11630
H	-4.12180	-1.55480	-5.38560
C	-8.63880	-2.26570	-0.38380
C	-8.45290	-0.99060	-0.02370
C	-7.09860	-0.39950	-0.05910
C	-6.04910	-1.13470	-0.46520
C	-6.26150	-2.55240	-0.84870
C	-7.48850	-3.08590	-0.81190
H	-9.62990	-2.69970	-0.35700
H	-9.29390	-0.38870	0.29530
H	-6.97970	0.62960	0.24640
H	-5.42230	-3.16910	-1.14470
H	-7.63910	-4.12150	-1.08820
C	0.29190	4.11050	1.99220
N	-0.15400	2.87160	2.46880
C	-2.13500	5.90160	-1.11020
C	-1.99750	4.51660	-1.04760
C	-1.22320	3.91580	-0.05480
C	-0.55370	4.70600	0.90050
C	-0.69760	6.10520	0.81770
C	-1.48270	6.69480	-0.16810
H	-2.74600	6.35690	-1.88400
H	-2.50320	3.89820	-1.78010
H	-1.15150	2.83210	-0.03860
H	-0.20950	6.75190	1.53430
H	-1.58210	7.77580	-0.19640
C	2.85930	6.00430	-0.06430
C	2.94910	6.62710	-1.31140
C	2.57900	5.95820	-2.48750
C	2.12170	4.64020	-2.45320
H	3.15640	6.53380	0.83620
H	3.31570	7.64710	-1.37340
H	2.65720	6.47010	-3.44150
H	1.84150	4.11100	-3.35790
C	1.81010	2.52680	0.41570
C	2.13300	3.74490	1.08720
C	2.39860	4.68810	-0.00440
C	2.05480	4.02750	-1.20770
H	1.55350	1.57360	0.85210
H	2.68250	3.73260	2.02020
N	1.69210	2.70900	-0.88600
H	1.22520	1.93480	-1.46090
C	0.36470	2.14850	3.55170
O	1.24230	2.56570	4.29310
C	-0.27540	0.78750	3.73470
H	-0.37020	0.26400	2.77940
H	0.33130	0.21780	4.43780
H	-1.28050	0.90340	4.15410
C	0.77380	5.08560	3.05540
H	1.30820	5.92100	2.60380
H	-0.10340	5.48000	3.58220
H	1.40800	4.58670	3.78030

TS7-Z

(B3LYP/6-31G**:UFF) Energy = -1525.511967

(B3LYP/6-31G**:UFF) Free Energy = -1524.427634

M06-2X/6-31G** Derived free energy = -4258.973813

M06-2X/6-31G** Derived free energy in solution = -4259.007267

Number of Imaginary Frequencies = 1 (-273.52)

ONIOM (B3LYP/6-31G**:UFF) Geometry

C	-1.14420	-2.94910	0.66740
C	-3.73260	-2.27960	1.59340
C	-3.21720	-1.65160	0.35650
C	-1.96580	-1.94430	-0.05760
H	-4.73160	-2.03670	1.93710
C	2.80180	-4.04710	-0.67210
C	2.52710	-2.76720	-0.38040
C	1.12230	-2.36420	-0.08900
C	0.17730	-3.31760	0.08930
H	3.81430	-4.35900	-0.89250
C	-2.96910	-3.12470	2.29820
C	-1.60320	-3.47180	1.82840
C	-0.79580	-4.37660	2.68580
C	-1.31000	-4.88470	3.81210
C	-2.68560	-4.56410	4.24950
C	-3.46640	-3.73720	3.54590
H	0.22440	-4.62660	2.42590
H	-0.70520	-5.54090	4.42490
H	-3.06230	-5.00410	5.16400
H	-4.46660	-3.51910	3.90110
C	0.47520	-4.72660	-0.30650
C	1.73170	-5.06340	-0.67480
C	2.04320	-6.45760	-1.06650
C	1.07850	-7.38360	-1.07970
C	-0.30230	-7.01830	-0.71040
C	-0.59250	-5.76290	-0.34950
H	3.05330	-6.73200	-1.34460
H	1.30660	-8.40180	-1.36740
H	-1.08200	-7.76840	-0.74000
H	-1.61540	-5.51330	-0.10060
P	-0.24160	-0.29220	-0.84100
O	-1.43540	-1.36360	-1.17420
O	0.84380	-1.04690	0.18400
O	0.37820	0.01980	-2.18210
O	-0.74260	0.84450	0.01640
H	-0.30190	1.99030	1.16350
Si	-4.33870	-0.41910	-0.49890
Si	3.92600	-1.53780	-0.19980
C	7.97320	-3.88280	0.18050
C	7.77270	-3.07300	-0.86500
C	6.50180	-2.33000	-0.99470
C	5.54410	-2.45470	-0.05880
C	5.77030	-3.36460	1.09150
C	6.92130	-4.03860	1.20420
H	8.90310	-4.42850	0.27580
H	8.54020	-2.96100	-1.61990
H	6.36300	-1.69980	-1.86130
H	5.00410	-3.50190	1.84090
H	7.08140	-4.70400	2.04270
C	4.07020	1.31220	-3.92600
C	3.21680	0.28250	-3.90480
C	3.18110	-0.62670	-2.73980
C	3.98230	-0.40900	-1.68410
C	4.97100	0.69620	-1.74450
C	5.00070	1.52010	-2.79910
H	4.09070	1.98470	-4.77370
H	2.54050	0.12040	-4.73400
H	2.48960	-1.45810	-2.75420
H	5.68970	0.82960	-0.94640
H	5.72030	2.32740	-2.83800
C	3.26810	0.69850	3.88080
C	3.04080	-0.61530	3.78300
C	3.23780	-1.30660	2.49260
C	3.65070	-0.62550	1.40840
C	3.88040	0.83690	1.52370
C	3.69950	1.46080	2.69390
H	3.12150	1.20770	4.82450
H	2.71120	-1.17540	4.64840
H	3.03420	-2.36810	2.44010
H	4.17830	1.42200	0.67040

H	3.86200	2.52780	2.77100
C	-5.59940	2.59140	2.86940
C	-6.54310	1.92800	2.19090
C	-6.16120	0.97670	1.12560
C	-4.86570	0.78350	0.82390
C	-3.82660	1.50760	1.59500
C	-4.17370	2.36790	2.55890
H	-5.87820	3.28790	3.64950
H	-7.58890	2.08580	2.42080
H	-6.94460	0.44360	0.60680
H	-2.78190	1.34280	1.38400
H	-3.41030	2.89630	3.11560
C	-2.24550	1.76330	-4.08300
C	-2.56220	2.45570	-2.98320
C	-3.24720	1.78640	-1.85710
C	-3.53240	0.47460	-1.92160
C	-3.21620	-0.27670	-3.16130
C	-2.59780	0.33260	-4.18040
H	-1.73680	2.25060	-4.90460
H	-2.30760	3.50530	-2.91100
H	-3.48950	2.37080	-0.98180
H	-3.46910	-1.32670	-3.23940
H	-2.35500	-0.22130	-5.07790
C	-8.01810	-2.62450	-2.37980
C	-7.88510	-1.30020	-2.51730
C	-6.72550	-0.60680	-1.91930
C	-5.80740	-1.29880	-1.22360
C	-5.95260	-2.76900	-1.08710
C	-7.00280	-3.39470	-1.63340
H	-8.86630	-3.13430	-2.81820
H	-8.62540	-0.73390	-3.06740
H	-6.63030	0.46440	-2.04940
H	-5.21190	-3.34970	-0.55510
H	-7.10770	-4.46740	-1.53430
C	0.15210	3.96750	1.03440
N	0.05920	2.77080	1.74710
C	1.10330	7.72650	2.94880
C	-0.01820	7.60570	2.13280
C	-0.31410	6.38570	1.52210
C	0.49420	5.25560	1.73100
C	1.62310	5.39680	2.55890
C	1.92430	6.61460	3.15640
H	1.33910	8.67610	3.42010
H	-0.66830	8.45880	1.96390
H	-1.19380	6.31990	0.89460
H	2.25990	4.54430	2.75150
H	2.80070	6.69430	3.79210
C	1.78310	5.94690	-1.68550
C	1.50880	6.37630	-2.98650
C	1.15280	5.46850	-3.99600
C	1.06970	4.09930	-3.73790
H	2.04750	6.66010	-0.91150
H	1.57190	7.43420	-3.22290
H	0.94300	5.83620	-4.99550
H	0.79980	3.38770	-4.51120
C	1.70820	2.43400	-0.63780
C	1.84180	3.78130	-0.17840
C	1.70210	4.58150	-1.40330
C	1.35570	3.68440	-2.44230
H	1.74020	1.52570	-0.05750
H	2.54470	4.02480	0.60570
N	1.37090	2.38490	-1.91310
H	0.97760	1.45730	-2.28480
C	0.29110	2.41800	3.07670
O	0.76360	3.14430	3.93350
C	-0.15180	0.99020	3.36300
H	0.24410	0.69470	4.33420
H	-1.24540	0.94150	3.39700
H	0.17830	0.29870	2.58240
C	-0.95580	4.02440	-0.00350
H	-0.81760	4.83480	-0.71660
H	-1.01960	3.07070	-0.52790
H	-1.90900	4.16520	0.51980

TS8-*E*

(B3LYP/6-31G**:UFF) Energy = -1525.520746

(B3LYP/6-31G**:UFF) Free Energy = -1524.434504

M06-2X/6-31G** Derived free energy = -4258.980985

M06-2X/6-31G** Derived free energy in solution = -4259.012376

Number of Imaginary Frequencies = 1 (-265.35)

ONIOM (B3LYP/6-31G**:UFF) Geometry

C	-1.20280	-2.52610	0.90650	P	-0.23070	0.03980	-0.61690
C	-3.84710	-1.81760	1.60600	O	-1.36530	-1.06060	-1.05230
C	-3.27090	-1.32760	0.33330	O	0.89240	-0.75320	0.33530
C	-1.98710	-1.61630	0.03460	O	0.43610	0.51400	-1.88880
H	-4.86700	-1.55780	1.86470	O	-0.81850	1.08050	0.31000
C	2.68280	-3.90740	-0.33840	H	-0.53170	2.76980	0.67280
C	2.46110	-2.59070	-0.20990	Si	-4.34890	-0.24360	-0.73790
C	1.08860	-2.09850	0.11530	Si	3.87010	-1.39660	-0.49960
C	0.11160	-2.99480	0.40030	C	2.89810	0.84160	-4.50500
H	3.66170	-4.28260	-0.60360	C	3.60380	1.46810	-3.55740
C	-3.10450	-2.54180	2.45380	C	3.90520	0.78490	-2.28220
C	-1.71220	-2.91570	2.09740	C	3.46790	-0.46650	-2.06360
C	-0.94230	-3.71180	3.08580	C	2.71050	-1.16550	-3.13230
C	-1.50530	-4.09100	4.23920	C	2.43410	-0.54180	-4.28390
C	-2.90110	-3.73410	4.57430	H	2.67190	1.34410	-5.43640
C	-3.65450	-3.00670	3.74230	H	3.95140	2.48020	-3.71940
H	0.08890	-3.98230	2.89870	H	4.46710	1.32390	-1.53800
H	-0.92710	-4.66770	4.94960	H	2.37210	-2.18330	-2.98710
H	-3.31650	-4.06700	5.51670	H	1.87420	-1.05150	-5.05730
H	-4.67200	-2.76120	4.02270	C	4.58320	1.26280	3.29890
C	0.35060	-4.45160	0.16570	C	5.33400	1.44110	2.20670
C	1.58780	-4.87770	-0.16760	C	5.12170	0.59260	1.01570
C	1.84580	-6.32010	-0.38800	C	4.13800	-0.32330	1.00040
C	0.84970	-7.20540	-0.28060	C	3.34290	-0.54760	2.23450
C	-0.51310	-6.74720	0.04710	C	3.55530	0.20450	3.32010
C	-0.75460	-5.44730	0.25250	H	4.73760	1.88190	4.17310
H	2.84200	-6.66270	-0.63890	H	6.10020	2.20530	2.19320
H	1.03870	-8.25860	-0.44320	H	5.78320	0.71510	0.16900
H	-1.32000	-7.46590	0.11080	H	2.60770	-1.33770	2.28370
H	-1.76730	-5.13440	0.46770	H	2.98670	0.02450	4.22210

C	7.89760	-3.72590	-1.02170	H	-9.35830	-0.57640	-0.32400
C	7.39970	-3.03170	-2.05190	H	-7.13470	0.61190	-0.19970
C	6.12830	-2.29270	-1.90300	H	-5.18480	-3.08320	-1.35180
C	5.46620	-2.31880	-0.73390	H	-7.31630	-4.19810	-1.45920
C	6.02470	-3.08620	0.40510	C	0.59920	4.07360	1.82180
C	7.17660	-3.75390	0.26700	N	-0.38470	3.77330	0.88570
H	8.82860	-4.26710	-1.12990	C	0.71510	1.47030	5.29120
H	7.92690	-3.00930	-2.99690	C	-0.23540	1.26810	4.29350
H	5.74880	-1.73860	-2.75070	C	-0.26420	2.08270	3.16140
H	5.50100	-3.11130	1.35270	C	0.66970	3.11910	2.99930
H	7.58240	-4.31510	1.09880	C	1.63600	3.29840	4.00610
C	-4.68250	4.01050	1.22510	C	1.65650	2.49010	5.13900
C	-3.94490	3.06210	1.81310	H	0.73130	0.83760	6.17340
C	-3.84590	1.71850	1.20690	H	-0.96880	0.47280	4.38850
C	-4.48510	1.44250	0.05610	H	-1.00490	1.88380	2.40090
C	-5.30090	2.50120	-0.58950	H	2.38590	4.07790	3.91840
C	-5.39680	3.71480	-0.03290	H	2.41330	2.65460	5.90000
H	-4.75250	4.99630	1.66600	C	2.79860	5.95720	-0.50730
H	-3.41420	3.27780	2.73150	C	2.65950	6.48740	-1.79070
H	-3.24340	0.97430	1.70730	C	2.16140	5.71270	-2.85020
H	-5.82110	2.29660	-1.51700	C	1.80830	4.37660	-2.66200
H	-5.99070	4.48480	-0.50780	H	3.18400	6.57040	0.30150
C	-2.49000	0.01240	-5.04510	H	2.93900	7.52010	-1.97430
C	-2.71400	1.13390	-4.35210	H	2.05800	6.15770	-3.83460
C	-3.32030	1.06510	-3.00670	H	1.43990	3.76110	-3.47620
C	-3.65400	-0.12110	-2.46890	C	2.08520	2.45580	0.34730
C	-3.40340	-1.36030	-3.24740	C	2.38600	3.75080	0.88600
C	-2.85050	-1.29550	-4.46480	C	2.43870	4.62460	-0.29200
H	-2.04100	0.06090	-6.02870	C	1.96960	3.85870	-1.38020
H	-2.44590	2.09470	-4.77220	H	1.95330	1.52280	0.87690
H	-3.47210	1.98730	-2.47120	H	3.04150	3.86780	1.73920
H	-3.63160	-2.32780	-2.82640	N	1.75580	2.54720	-0.92790
H	-2.65640	-2.20260	-5.02230	H	1.27260	1.73530	-1.46000
C	-8.50970	-2.40850	-0.90310	C	-1.06810	4.64960	0.03280
C	-8.45090	-1.11700	-0.56020	O	-1.04890	5.86450	0.13270
C	-7.14700	-0.42360	-0.50170	C	-1.84790	3.93490	-1.05130
C	-6.01450	-1.07880	-0.81260	H	-2.79800	4.45010	-1.19960
C	-6.08710	-2.51880	-1.16400	H	-1.28210	4.01230	-1.98580
C	-7.26890	-3.14560	-1.21090	H	-1.99810	2.87810	-0.83080
H	-9.46390	-2.91770	-0.94460	C	0.74680	5.53460	2.21210

H	1.58940	5.66600	2.89090	H	-0.16470	5.83820	2.73750
H	0.85120	6.18420	1.34900				

TS8-*Z*

(B3LYP/6-31G**:UFF) Energy = -1525.509399

(B3LYP/6-31G**:UFF) Free Energy = -1524.424979

M06-2X/6-31G** Derived free energy = -4258.971725

M06-2X/6-31G** Derived free energy in solution = -4259.004456

Number of Imaginary Frequencies = 1 (-247.70)

ONIOM (B3LYP/6-31G**:UFF) Geometry

C	1.58050	-2.62300	-1.02930	H	0.01900	-8.59230	0.25190
C	4.09580	-1.56830	-1.77810	H	2.28610	-7.51030	-0.18400
C	3.48270	-1.15740	-0.49570	H	2.45850	-5.13560	-0.51010
C	2.25340	-1.61330	-0.17380	P	0.30180	-0.23640	0.59810
H	5.06470	-1.17240	-2.05840	O	1.60300	-1.17060	0.94680
C	-2.14440	-4.47480	0.06740	O	-0.70340	-1.11480	-0.41050
C	-2.07620	-3.13890	-0.03720	O	-0.38210	0.03190	1.91690
C	-0.75160	-2.48030	-0.24830	O	0.68970	0.93390	-0.27860
C	0.33330	-3.24740	-0.51600	H	0.32200	2.55820	-0.39810
H	-3.08800	-4.96990	0.25240	Si	4.42490	0.05680	0.56580
C	3.44870	-2.40010	-2.60450	Si	-3.64430	-2.12630	0.11190
C	2.12430	-2.95460	-2.22380	C	-7.39300	-4.93120	0.17900
C	1.45440	-3.85300	-3.19820	C	-6.64170	-4.83810	-0.92450
C	2.05030	-4.16780	-4.35440	C	-5.45920	-3.95240	-0.94240
C	3.38310	-3.63440	-4.70960	C	-5.13210	-3.23890	0.14810
C	4.04130	-2.80270	-3.89520	C	-5.96840	-3.34560	1.36860
H	0.46820	-4.25220	-3.00020	C	-7.03960	-4.14870	1.38090
H	1.54470	-4.82180	-5.05330	H	-8.25950	-5.57960	0.19390
H	3.82900	-3.92070	-5.65350	H	-6.89810	-5.41090	-1.80630
H	5.01380	-2.42740	-4.19100	H	-4.86270	-3.89060	-1.84440
C	0.26210	-4.72450	-0.30360	H	-5.71960	-2.78300	2.25830
C	-0.92890	-5.30190	-0.03620	H	-7.64860	-4.22830	2.27210
C	-1.01770	-6.76730	0.16460	C	-3.42880	0.10650	4.23760
C	0.08430	-7.52210	0.10350	C	-4.27040	0.54260	3.29470
C	1.39490	-6.89670	-0.15530	C	-4.34130	-0.14450	1.98840
C	1.48400	-5.57490	-0.34280	C	-3.54660	-1.19750	1.72880
H	-1.97530	-7.23240	0.36330	C	-2.63950	-1.69470	2.79440

C	-2.58060	-1.07260	3.97840
H	-3.37130	0.61320	5.19220
H	-4.89590	1.40570	3.48260
H	-5.03800	0.22920	1.25840
H	-2.01600	-2.56250	2.62540
H	-1.91170	-1.43350	4.74890
C	-4.19930	0.68250	-3.61500
C	-5.12260	0.65900	-2.64820
C	-4.96440	-0.25060	-1.49460
C	-3.87220	-1.02670	-1.38310
C	-2.89140	-1.04770	-2.49950
C	-3.03940	-0.22640	-3.54590
H	-4.31040	1.35210	-4.45790
H	-5.98490	1.31050	-2.70510
H	-5.75110	-0.28290	-0.75520
H	-2.06320	-1.74030	-2.49690
H	-2.31960	-0.24110	-4.35410
C	2.95150	-0.38750	5.00510
C	3.40940	-1.45190	4.33800
C	3.89180	-1.30450	2.94930
C	3.88320	-0.10160	2.34770
C	3.37870	1.07450	3.09980
C	2.93480	0.93680	4.35500
H	2.59220	-0.49450	6.02050
H	3.41880	-2.42580	4.80990
H	4.20870	-2.19050	2.41960
H	3.35880	2.05190	2.64850
H	2.56340	1.79710	4.89700
C	8.94840	-1.15620	0.23970
C	8.57950	0.10020	-0.03230
C	7.15820	0.49360	0.06450
C	6.22950	-0.40170	0.44500
C	6.64510	-1.79990	0.71950
C	7.93250	-2.15440	0.62590
H	9.98880	-1.44570	0.16750
H	9.32110	0.83230	-0.32500
H	6.89360	1.50980	-0.18350
H	5.91010	-2.55230	0.96650
H	8.22990	-3.17700	0.81900
C	3.71190	4.41190	-1.04370

C	3.15550	3.37060	-1.67190
C	3.39070	1.99690	-1.18070
C	4.14610	1.78490	-0.08830
C	4.77460	2.94510	0.59120
C	4.56870	4.18780	0.13760
H	3.53470	5.41890	-1.39850
H	2.52430	3.53200	-2.53630
H	2.93330	1.17600	-1.71510
H	5.40460	2.79120	1.45830
H	5.02530	5.03150	0.63850
C	-1.00420	3.69950	-1.44320
N	-0.03760	3.53520	-0.47990
C	-1.68130	7.54110	-3.29950
C	-0.46330	7.24610	-2.68720
C	-0.27630	6.02910	-2.03690
C	-1.30540	5.07410	-1.98410
C	-2.51280	5.37060	-2.63500
C	-2.70470	6.59450	-3.27420
H	-1.82910	8.49510	-3.79670
H	0.34870	7.96640	-2.71020
H	0.67870	5.81630	-1.57590
H	-3.32180	4.65000	-2.64750
H	-3.65540	6.80260	-3.75600
C	-3.55350	5.18030	0.93670
C	-3.50050	5.67990	2.23610
C	-2.92320	4.94040	3.28210
C	-2.39540	3.66980	3.06130
H	-3.98630	5.76930	0.13490
H	-3.90730	6.66420	2.44620
H	-2.89430	5.36300	4.28150
H	-1.96350	3.08140	3.86450
C	-2.36710	1.84140	-0.02060
C	-2.85370	3.08640	-0.50780
C	-3.02220	3.91200	0.68880
C	-2.46480	3.17830	1.76040
H	-2.11780	0.94540	-0.56790
H	-3.49030	3.13930	-1.37890
N	-2.05950	1.93130	1.26550
H	-1.45530	1.17950	1.73020
C	0.28000	4.37110	0.60890

O	-0.16200	5.49390	0.75750	C	-0.90870	2.64920	-2.54430
C	1.24710	3.72830	1.58120	H	-0.11920	2.96670	-3.23580
H	2.09840	4.39760	1.72730	H	-0.63730	1.66870	-2.15000
H	0.74570	3.62780	2.54800	H	-1.83670	2.59280	-3.11600
H	1.58160	2.74560	1.25050				

TS9-*E*

(B3LYP/6-31G**:UFF) Energy = -1525.480551

(B3LYP/6-31G**:UFF) Free Energy = -1524.597411

M06-2X/6-31G** Derived free energy = -3215.934929

M06-2X/6-31G** Derived free energy in solution = -3215.971268

Number of Imaginary Frequencies = 1 (-264.00)

ONIOM (B3LYP/6-31G**:UFF) Geometry

C	1.44600	3.48580	0.60280	C	0.01090	7.50330	-2.50930
C	4.01180	2.72990	1.52090	C	1.29320	7.09610	-1.90530
C	3.29640	1.85540	0.56150	C	1.36310	5.99100	-1.15410
C	2.03090	2.17020	0.19750	H	-2.01900	7.05800	-2.77950
H	5.00500	2.45180	1.85420	H	-0.03820	8.40000	-3.11350
C	-2.22080	4.68410	-1.35350	H	2.18130	7.68910	-2.08200
C	-2.17340	3.54940	-0.64070	H	2.32150	5.70090	-0.74650
C	-0.90010	3.14460	0.01040	P	-0.08500	0.74960	-0.08010
C	0.19210	3.93130	-0.06600	O	1.33640	1.36320	-0.67090
H	-3.14090	4.98240	-1.84320	O	-0.84540	1.97310	0.71830
C	3.42890	3.83530	2.00320	O	-0.85600	0.35630	-1.32960
C	2.08250	4.24120	1.52690	C	-0.49280	-3.82050	1.56750
C	1.50310	5.48600	2.09260	N	-1.11060	-2.60280	1.77970
C	2.19040	6.21390	2.98060	C	3.78900	-3.85570	2.23670
C	3.53940	5.81140	3.43350	C	3.02620	-2.74900	2.60200
C	4.12380	4.69690	2.98050	C	1.65340	-2.71750	2.35420
H	0.51180	5.81540	1.80900	C	1.01100	-3.80520	1.74000
H	1.74980	7.11650	3.38420	C	1.79400	-4.91750	1.38070
H	4.06020	6.43320	4.15030	C	3.16520	-4.94110	1.61850
H	5.11250	4.43260	3.33640	H	4.85800	-3.87300	2.42570
C	0.14590	5.16920	-0.90590	H	3.49660	-1.89420	3.07810
C	-1.01810	5.53040	-1.49710	H	1.09260	-1.83520	2.63180
C	-1.08380	6.75900	-2.32250	H	1.33630	-5.78200	0.91090

H	3.74570	-5.80980	1.32300	C	6.21120	-2.67320	-1.64400
C	-3.23230	-5.01060	-0.69870	C	7.04240	-2.47380	-2.86130
C	-4.49560	-4.54150	-1.06000	C	7.73530	-3.49330	-3.38400
C	-4.68480	-3.22470	-1.51020	H	8.24240	-5.64770	-3.18000
C	-3.61260	-2.34070	-1.62040	H	6.89490	-5.99530	-1.19500
H	-3.10420	-6.03330	-0.35740	H	5.59720	-4.07640	-0.17720
H	-5.35100	-5.20650	-0.99290	H	7.07970	-1.50640	-3.34580
H	-5.68080	-2.89290	-1.78580	H	8.33340	-3.34390	-4.27360
H	-3.73710	-1.32480	-1.97800	C	-8.56960	-2.22040	-0.24110
C	-0.16120	-3.00720	-0.96570	C	-8.63490	-1.26260	-1.17230
C	-0.71570	-4.23450	-0.47860	C	-7.59650	-0.21240	-1.23160
C	-2.14170	-4.14020	-0.79550	C	-6.57920	-0.20690	-0.34810
C	-2.35650	-2.82700	-1.26710	C	-6.51880	-1.27850	0.68440
H	0.86820	-2.67830	-0.91740	C	-7.45930	-2.22960	0.73060
H	-0.16300	-5.16360	-0.49860	H	-9.32820	-2.99120	-0.19940
C	-2.40280	-2.35800	2.27010	H	-9.44840	-1.25470	-1.88620
O	-3.14570	-3.22120	2.70360	H	-7.68220	0.55620	-1.98910
C	-2.78850	-0.89630	2.20380	H	-5.69890	-1.31580	1.38900
H	-1.95690	-0.22990	2.43940	H	-7.40250	-3.01370	1.47460
H	-3.09340	-0.66830	1.17740	C	-3.84620	2.28510	0.63180
H	-3.63270	-0.72730	2.87170	H	-3.40450	2.64530	1.55300
C	-5.51450	0.82590	-0.41880	C	-3.98530	2.15840	-1.79980
C	-4.97480	1.33530	0.70420	H	-3.60310	2.46360	-2.76610
C	-3.36040	2.67280	-0.55870	C	-4.99560	1.28350	-1.73520
H	-5.33920	1.04180	1.68040	H	-5.40960	0.88890	-2.65430
C	5.45750	-1.53070	-1.06670	C	5.38790	0.86500	-0.54260
C	4.21620	-1.69280	-0.57150	H	5.84720	1.84580	-0.53930
C	4.01880	0.68390	-0.00020	C	3.47800	-0.54640	-0.00680
H	3.72770	-2.65900	-0.59300	H	2.50620	-0.72650	0.41790
C	-1.16230	-5.05940	2.13310	C	6.06460	-0.17550	-1.04430
H	-0.59700	-5.95090	1.85890	H	7.06740	-0.02020	-1.42170
H	-1.16110	-4.97780	3.22540	O	0.15060	-0.29470	0.98350
H	-2.19830	-5.15030	1.82090	H	-0.61010	-1.75030	1.45280
C	7.68030	-4.82680	-2.75380	N	-1.11680	-2.17540	-1.34110
C	6.93570	-5.01850	-1.65950	H	-0.98970	-1.08870	-1.45310
C	6.17000	-3.89670	-1.07750				

TS9-*Z*

(B3LYP/6-31G**:UFF) Energy = -1525.471492

(B3LYP/6-31G**:UFF) Free Energy = -1524.589768

M06-2X/6-31G** Derived free energy = -3215.930159

M06-2X/6-31G** Derived free energy in solution = -3215.967012

Number of Imaginary Frequencies = 1 (-259.09)

ONIOM (B3LYP/6-31G**:UFF) Geometry

C	-3.69520	-2.16630	-0.39430
C	-2.54190	-4.50670	-1.50190
C	-1.74340	-3.66520	-0.58120
C	-2.28540	-2.53880	-0.07330
H	-2.10430	-5.41070	-1.90970
C	-5.19310	1.51930	1.29660
C	-4.09350	1.52560	0.52600
C	-3.57440	0.24390	-0.01630
C	-4.23500	-0.91390	0.20180
H	-5.58550	2.44750	1.69570
C	-3.79110	-4.15450	-1.83410
C	-4.39720	-2.91630	-1.27700
C	-5.75290	-2.54610	-1.75860
C	-6.41130	-3.33680	-2.61460
C	-5.81870	-4.59270	-3.12190
C	-4.58970	-4.97870	-2.76310
H	-6.22680	-1.62830	-1.43700
H	-7.39890	-3.05000	-2.95250
H	-6.39150	-5.20510	-3.80650
H	-4.18380	-5.90000	-3.16360
C	-5.42530	-0.91960	1.10510
C	-5.88290	0.25000	1.61150
C	-7.06460	0.26090	2.50480
C	-7.66960	-0.88530	2.83400
C	-7.15580	-2.16770	2.31700
C	-6.09240	-2.18890	1.50530
H	-7.44170	1.19740	2.89660
H	-8.53180	-0.87600	3.48810
H	-7.63730	-3.09340	2.60470
H	-5.72130	-3.14300	1.15640
P	-1.11060	-0.27780	0.10180
O	-1.54920	-1.73340	0.76020
O	-2.41670	0.24270	-0.75310
O	-0.86280	0.61620	1.30450
C	3.40610	0.08370	-0.91440
N	2.35340	0.97200	-1.02340
C	7.42440	1.04290	-2.19540
C	6.35790	1.86850	-2.54620
C	5.06870	1.59660	-2.09020
C	4.81470	0.48740	-1.26830
C	5.89660	-0.35420	-0.95060
C	7.18560	-0.07630	-1.39680
H	8.42840	1.26260	-2.54550
H	6.52340	2.73500	-3.17910
H	4.26070	2.25150	-2.38030
H	5.74430	-1.24370	-0.34950
H	8.00060	-0.74010	-1.12430
C	4.99030	1.59460	2.14910
C	4.84340	2.79700	2.83770
C	3.58060	3.25370	3.25020
C	2.42750	2.51060	3.00390
H	5.96830	1.26050	1.81850
H	5.72080	3.39860	3.05420
H	3.50180	4.20000	3.77610
H	1.44900	2.85070	3.32690
C	2.21330	-0.68400	1.38950
C	3.59120	-0.40620	1.15740
C	3.84620	0.83950	1.88190
C	2.59030	1.30360	2.32970
H	1.61860	-1.50670	1.02280
H	4.32300	-1.19040	1.04470
C	2.28640	2.34320	-0.70040
O	3.25540	3.02240	-0.41830
C	0.87500	2.89170	-0.75140
H	0.41090	2.70290	-1.72460
H	0.24370	2.41050	0.00360
H	0.92360	3.96310	-0.56280
C	-1.97510	5.18130	-0.39790

C	-2.27020	4.86690	0.87950
C	-3.39360	2.79350	0.21740
H	-1.94600	5.49600	1.69870
C	2.40290	-4.56050	0.24930
C	1.94850	-4.52290	-1.01930
C	-0.33780	-4.03200	-0.29480
H	2.62470	-4.62070	-1.85880
C	3.03660	-1.28280	-1.48270
H	2.00220	-1.54380	-1.25630
H	3.71560	-2.05960	-1.12860
H	3.14180	-1.23040	-2.57250
C	6.61900	-4.97300	1.10330
C	6.07680	-5.65850	0.09120
C	4.63370	-5.53290	-0.20170
C	3.85080	-4.72700	0.54520
C	4.46540	-3.98170	1.67700
C	5.77410	-4.09420	1.93410
H	7.67640	-5.06240	1.31630
H	6.69440	-6.30610	-0.51770
H	4.22290	-6.11190	-1.01890
H	3.86480	-3.32260	2.29030
H	6.21660	-3.53880	2.75100
C	0.23720	8.75160	-1.41540
C	-0.65750	8.78460	-0.42210
C	-1.41760	7.56790	-0.06700
C	-1.21450	6.41370	-0.73560
C	-0.21890	6.39310	-1.84180
C	0.46700	7.49810	-2.15890
H	0.79350	9.64260	-1.67630
H	-0.82960	9.70390	0.12270
H	-2.15350	7.63400	0.72430
H	-0.02640	5.48000	-2.39000
H	1.19500	7.47420	-2.95960
C	-3.01260	3.62990	1.19820
H	-3.21700	3.39480	2.23570
C	-3.07160	3.13450	-1.18860
H	-3.38880	2.48070	-1.99150
C	-2.40740	4.25870	-1.47900
H	-2.20420	4.49650	-2.51520
C	0.14570	-4.11970	1.10420
H	-0.54590	-3.98630	1.92670
C	0.52420	-4.25390	-1.30060
H	0.20070	-4.18190	-2.33190
C	1.43460	-4.37170	1.36050
H	1.76210	-4.44350	2.38960
O	-0.00370	-0.47200	-0.90790
H	1.42290	0.50650	-1.07860
N	1.62500	0.34460	1.98410
H	0.56320	0.48450	1.91550

TS10-*E*

(B3LYP/6-31G**:UFF) Energy = -1525.482878

(B3LYP/6-31G**:UFF) Free Energy = -1524.601406

M06-2X/6-31G** Derived free energy = -3215.937736

M06-2X/6-31G** Derived free energy in solution = -3215.972448

Number of Imaginary Frequencies = 1 (-279.18)

ONIOM (B3LYP/6-31G**:UFF) Geometry

C	2.34360	3.26290	0.21160
C	4.63320	2.13950	1.44400
C	3.86340	1.33350	0.46620
C	2.76240	1.86570	-0.10710
H	5.52350	1.71870	1.89680
C	-1.24520	4.73000	-1.69670
C	-1.28480	3.61640	-0.94870
C	-0.03290	3.10510	-0.33120

C	1.12450	3.79020	-0.45840
H	-2.14950	5.11020	-2.15790
C	4.23500	3.37550	1.77280
C	3.02030	3.96420	1.15160
C	2.59110	5.30310	1.63110
C	3.31460	5.96460	2.54210
C	4.55210	5.39170	3.11330
C	4.98580	4.17710	2.75980
H	1.68270	5.76160	1.26320
H	2.98580	6.94000	2.87740
H	5.11040	5.96680	3.84070
H	5.89110	3.78490	3.20780
C	1.16960	4.99950	-1.33540
C	0.02860	5.44910	-1.90890
C	0.05570	6.65010	-2.77590
C	1.21010	7.28110	-3.01500
C	2.46440	6.77690	-2.42490
C	2.45060	5.69590	-1.63650
H	-0.85930	7.02030	-3.22140
H	1.22880	8.15750	-3.64990
H	3.39850	7.27960	-2.64020
H	3.38630	5.33270	-1.23360
P	0.55940	0.64290	-0.43390
O	2.02200	1.13380	-1.00240
O	-0.06180	1.93730	0.38980
O	-0.26160	0.36960	-1.68430
C	-1.85930	-2.91040	1.77810
N	-0.55520	-2.84600	1.30110
C	-3.17870	0.50820	4.10700
C	-4.04440	-0.55750	3.85700
C	-3.61230	-1.65100	3.11110
C	-2.30460	-1.71250	2.59560
C	-1.43960	-0.63840	2.86680
C	-1.87910	0.45940	3.60750
H	-3.51680	1.36620	4.68020
H	-5.06210	-0.53760	4.23540
H	-4.31720	-2.45380	2.92270
H	-0.42670	-0.62620	2.48540
H	-1.19200	1.28110	3.78270
C	-2.76200	-5.06620	-1.12690
C	-2.06460	-5.64690	-2.18640
C	-1.20580	-4.88770	-2.99760
C	-1.03440	-3.51970	-2.78500
H	-3.41950	-5.66800	-0.50710
H	-2.18430	-6.70700	-2.38650
H	-0.67310	-5.37180	-3.81010
H	-0.38570	-2.91880	-3.41370
C	-2.63460	-1.48750	-0.31930
C	-3.08780	-2.77010	0.13390
C	-2.59600	-3.69960	-0.88620
C	-1.74740	-2.95170	-1.73260
H	-2.77570	-0.52980	0.15810
H	-4.03440	-2.89360	0.64360
C	0.30870	-3.91330	1.00750
O	0.06120	-5.08170	1.25230
C	1.60520	-3.48940	0.34810
H	1.84470	-2.43690	0.50260
H	2.39960	-4.13910	0.71930
H	1.51100	-3.66350	-0.72980
C	-4.95580	1.38860	-0.47250
C	-4.24830	1.77500	0.60710
C	-2.55760	2.88170	-0.77630
H	-4.57950	1.52770	1.60750
C	4.96020	-2.77870	-0.31880
C	4.91000	-2.31100	0.94320
C	4.27600	-0.06560	0.19290
H	5.07920	-2.96710	1.78770
C	-2.32260	-4.25110	2.32270
H	-3.36500	-4.19950	2.63520
H	-2.18140	-5.05540	1.60770
H	-1.71440	-4.49250	3.20080
C	5.76730	-6.93550	-1.15620
C	6.45810	-6.30460	-0.20020
C	6.18890	-4.88130	0.09310
C	5.24720	-4.20790	-0.59820
C	4.48640	-4.92050	-1.65890
C	4.73280	-6.21030	-1.91940
H	5.96400	-7.97780	-1.37130
H	7.21760	-6.83450	0.36000
H	6.77640	-4.39080	0.85910

H	3.71250	-4.40700	-2.21530	C	-3.34940	2.48490	-1.96510
H	4.17060	-6.72630	-2.68690	H	-3.00440	2.74470	-2.95820
C	-8.53310	-1.00120	-0.12880	C	-4.47610	1.77780	-1.82260
C	-7.48260	-1.25860	0.65800	H	-5.02330	1.47240	-2.70540
C	-6.25880	-0.43850	0.55100	C	4.36620	-0.56950	-1.20050
C	-6.18950	0.57570	-0.33390	H	4.18950	0.09880	-2.03390
C	-7.36340	0.85190	-1.20340	C	4.55480	-0.90240	1.20780
C	-8.47070	0.10550	-1.10380	H	4.48230	-0.57010	2.23620
H	-9.42980	-1.60240	-0.05260	C	4.68010	-1.84770	-1.44060
H	-7.52400	-2.07110	1.37190	H	4.74940	-2.19180	-2.46470
H	-5.41430	-0.68690	1.17760	O	0.70020	-0.44220	0.60360
H	-7.33750	1.66910	-1.91310	H	-0.18450	-1.91880	1.02960
H	-9.32480	0.31400	-1.73510	N	-1.80170	-1.60560	-1.33360
C	-2.99630	2.54210	0.44810	H	-1.12970	-0.77030	-1.61540
H	-2.44120	2.82540	1.33410				

TS10-Z

(B3LYP/6-31G**:UFF) Energy = -1525.473960

(B3LYP/6-31G**:UFF) Free Energy = -1524.592367

M06-2X/6-31G** Derived free energy = -3215.928014

M06-2X/6-31G** Derived free energy in solution = -3215.965673

Number of Imaginary Frequencies = 1 (-250.32)

ONIOM (B3LYP/6-31G**:UFF) Geometry

C	-4.38620	0.70510	0.41350	C	-7.19550	0.77940	2.81860
C	-4.21490	3.36100	1.39560	C	-7.14480	2.19410	3.24500
C	-3.19970	2.86890	0.43880	C	-6.20820	3.02870	2.78210
C	-3.27890	1.60610	-0.02630	H	-6.39560	-0.76620	1.67420
H	-4.14970	4.37940	1.76080	H	-7.95210	0.13170	3.24280
C	-4.22650	-3.37110	-1.02430	H	-7.87610	2.55490	3.95690
C	-3.18920	-2.85030	-0.35120	H	-6.19350	4.05560	3.12760
C	-3.25940	-1.44240	0.12450	C	-5.49840	-1.27270	-0.87470
C	-4.37020	-0.70030	-0.07920	C	-5.42870	-2.55610	-1.30200
H	-4.18350	-4.39110	-1.38850	C	-6.54200	-3.13930	-2.08630
C	-5.19890	2.55370	1.81490	C	-7.60900	-2.39520	-2.39580
C	-5.27890	1.14910	1.33100	C	-7.67700	-0.98550	-1.96700
C	-6.32480	0.28210	1.93210	C	-6.67930	-0.44960	-1.25430

H	-6.49450	-4.17020	-2.41470	H	1.22050	0.97780	-2.33630
H	-8.42080	-2.82210	-2.97040	C	0.57310	-4.91480	0.15180
H	-8.53350	-0.38260	-2.24000	C	-0.06950	-4.39700	1.21630
H	-6.74220	0.59210	-0.97050	C	-1.95070	-3.63890	-0.15660
P	-1.26560	0.07350	-0.21700	H	0.39170	-4.38350	2.19540
O	-2.32850	1.13120	-0.89730	C	0.37250	5.05520	-0.56560
O	-2.18000	-0.89160	0.77010	C	0.05230	4.82230	0.72200
O	-0.71530	-0.73300	-1.37980	C	-2.04160	3.72140	0.10120
C	3.11020	0.56220	1.39320	H	0.72540	5.10170	1.52290
N	2.41390	1.13710	0.35060	C	2.31070	0.61320	2.69110
C	7.20990	1.62510	2.26130	H	1.24860	0.43170	2.51810
C	6.42940	2.42990	1.43280	H	2.41850	1.62180	3.10740
C	5.12830	2.05590	1.10380	H	2.70220	-0.08740	3.43020
C	4.57200	0.86540	1.59940	C	4.20880	6.64820	-1.73210
C	5.36130	0.08080	2.45760	C	4.13230	5.75870	-0.73620
C	6.66690	0.44750	2.77580	C	2.81980	5.22280	-0.32110
H	8.22710	1.91360	2.50850	C	1.69010	5.62620	-0.93770
H	6.83090	3.35630	1.03350	C	1.78790	6.61520	-2.04290
H	4.54530	2.69810	0.46100	C	2.97990	7.09800	-2.41590
H	4.96430	-0.83130	2.88780	H	5.17010	7.03940	-2.03910
H	7.25590	-0.18680	3.43140	H	5.03210	5.42360	-0.23620
C	5.11400	-1.97790	-0.60500	H	2.79010	4.47910	0.46530
C	5.39870	-2.15560	-1.95770	H	0.89410	6.96280	-2.54530
C	4.38130	-2.15550	-2.92580	H	3.04580	7.82170	-3.21800
C	3.04370	-1.99860	-2.56670	C	4.60090	-6.41360	0.60630
H	5.91090	-1.96480	0.13110	C	4.24750	-5.77310	-0.51270
H	6.42870	-2.29160	-2.27250	C	2.86410	-5.28390	-0.68140
H	4.64000	-2.28770	-3.97170	C	1.94730	-5.46710	0.29030
H	2.24790	-2.01460	-3.30410	C	2.35040	-6.19700	1.52430
C	1.71460	-1.65610	0.74210	C	3.60500	-6.64090	1.67110
C	3.10200	-1.56500	1.04980	H	5.61550	-6.76880	0.73240
C	3.78110	-1.80850	-0.22380	H	4.97650	-5.60210	-1.29440
C	2.77190	-1.84120	-1.21110	H	2.62040	-4.74430	-1.58650
H	0.86700	-1.53830	1.40140	H	1.62910	-6.39000	2.30790
H	3.48980	-1.87430	2.00930	H	3.89110	-7.17330	2.56900
C	2.82200	1.39390	-0.97490	C	-1.37750	-3.73090	1.05500
O	3.96380	1.26620	-1.37280	H	-1.84450	-3.28270	1.92350
C	1.67820	1.84910	-1.85680	C	-1.28100	-4.26580	-1.32120
H	0.89070	2.33910	-1.28550	H	-1.74120	-4.22220	-2.30050
H	2.07030	2.51230	-2.62850	C	-0.09280	-4.86450	-1.17750

H	0.37660	-5.31550	-2.04240	H	-0.36400	4.85890	-2.66870
C	-1.72520	4.03010	-1.31340	O	-0.30200	0.76600	0.71960
H	-2.40500	3.72560	-2.09940	H	1.38010	1.12300	0.47550
C	-1.20620	4.13330	1.06840	N	1.53100	-1.72450	-0.56850
H	-1.40500	3.89800	2.10710	H	0.58360	-1.46880	-1.01390
C	-0.59220	4.66910	-1.62770				

Addition of Enamides Study

TS11-*E*

(B3LYP/6-31G**:UFF) Energy = -1679.333830

(B3LYP/6-31G**:UFF) Free Energy = -1678.364279

M06-2X/6-31G** Derived free energy = -3522.033080

M06-2X/6-31G** Derived free energy in solution = -3522.048641

Number of Imaginary Frequencies = 1 (-273.76)

ONIOM (B3LYP/6-31G**:UFF) Geometry

C	2.06030	2.64150	-0.63980	C	5.06510	1.48810	1.42300
C	0.14000	4.39090	-1.76840	C	4.17580	2.32630	0.84050
C	-0.27260	3.41980	-0.73220	C	4.33890	3.79420	1.03180
C	0.63140	2.55500	-0.22970	C	5.37590	4.27540	1.72690
H	-0.59660	5.07040	-2.18150	C	6.36100	3.35420	2.32380
C	4.87740	0.02650	1.33250	C	6.21190	2.03270	2.18600
C	3.81200	-0.48230	0.69900	H	3.61720	4.48960	0.62540
C	2.82810	0.42360	0.05490	H	5.48950	5.34330	1.86250
C	3.03160	1.76010	0.06050	H	7.19860	3.75110	2.88270
H	5.59300	-0.63370	1.80910	H	6.93280	1.36220	2.63710
C	1.40470	4.41120	-2.21120	P	0.29920	0.06780	0.09150
C	2.41770	3.48790	-1.63420	O	0.25450	1.61050	0.69260
C	3.78850	3.53690	-2.20240	O	1.77440	-0.11340	-0.64280
C	4.09390	4.41230	-3.16760	O	0.19840	-0.83920	1.29650
C	3.08800	5.35190	-3.70850	O	-0.70710	-0.08050	-1.02500
C	1.82570	5.35390	-3.26630	H	-2.37620	-0.75800	-1.33550
H	4.55740	2.85780	-1.85790	C	-4.17800	-1.64560	-0.76070
H	5.09850	4.43170	-3.57020	N	-3.17970	-1.31650	-1.66530
H	3.38220	6.04670	-4.48460	C	-4.46530	0.73530	2.83370
H	1.11430	6.05190	-3.69140	C	-3.27130	0.69080	2.11870

C	-3.18100	-0.05610	0.94520
C	-4.27590	-0.79640	0.46740
C	-5.47950	-0.73330	1.20030
C	-5.57290	0.02660	2.35980
H	-4.54040	1.32380	3.74330
H	-2.39870	1.24390	2.45150
H	-2.25250	-0.03460	0.39500
H	-6.35710	-1.27520	0.87230
H	-6.51430	0.06170	2.89940
C	-2.95230	-1.91630	-2.91780
C	-1.76380	-1.36500	-3.67630
H	-1.21430	-0.59520	-3.13590
H	-2.12400	-0.98710	-4.63770
H	-1.09160	-2.20020	-3.89080
O	-3.64530	-2.81030	-3.37890
C	-5.49880	-2.10580	-1.35600
H	-5.33840	-2.76800	-2.19930
H	-6.12130	-2.59820	-0.61110
H	-6.02870	-1.21290	-1.71140
C	-2.27380	-3.42410	0.34470
H	-0.87080	-2.28890	1.37340
C	0.40040	-4.65460	-2.78690
C	0.78770	-3.65340	-1.89700
C	-0.04030	-3.28380	-0.84180
C	-1.31990	-3.85670	-0.70200
C	-1.69380	-4.87410	-1.60630
C	-0.83820	-5.28010	-2.62320
H	1.05980	-4.94910	-3.59800
H	1.72870	-3.13390	-2.03060
H	0.31220	-2.53160	-0.14940
H	-2.65120	-5.36940	-1.50380
H	-1.14460	-6.07470	-3.29590
C	-3.65680	-3.52990	0.12810
N	-1.76790	-2.80420	1.45330
C	-2.40650	-2.66810	2.70840
C	-1.55570	-1.97280	3.74700
H	-2.21900	-1.41740	4.41140
H	-1.04960	-2.73830	4.34590
H	-0.80820	-1.31360	3.30240
O	-3.50570	-3.12830	2.95330
H	-3.98420	-4.14810	-0.69650
H	-4.31200	-3.46500	0.98280
C	-4.41770	3.34530	0.49700
C	-1.68940	3.39130	-0.29800
H	-5.45790	3.31340	0.80000
C	3.14610	-4.74880	0.63340
C	3.60430	-1.94620	0.67170
H	2.95900	-5.81620	0.61980
C	3.99240	-2.68730	-0.39350
C	3.75680	-4.15620	-0.40000
C	4.17340	-4.93640	-1.58230
C	4.74650	-4.32710	-2.62530
C	4.98790	-2.86840	-2.62960
C	4.64220	-2.09660	-1.59220
H	4.00870	-6.00670	-1.61660
H	5.04140	-4.91090	-3.48780
H	5.45690	-2.41810	-3.49510
H	4.84340	-1.03440	-1.64940
C	2.94660	-2.61750	1.83440
C	2.72030	-3.95200	1.80050
C	2.04990	-4.62200	2.93860
C	1.67870	-3.91530	4.01090
C	1.93250	-2.46360	4.06390
C	2.52760	-1.84610	3.03740
H	1.85910	-5.68740	2.90700
H	1.18860	-4.40590	4.84190
H	1.62590	-1.89700	4.93370
H	2.69160	-0.77820	3.09540
C	-4.02480	2.77820	-0.80690
C	-2.72920	2.80560	-1.20120
C	-2.37120	2.23610	-2.52950
C	-3.31560	1.70790	-3.31650
C	-4.71760	1.65720	-2.86440
C	-5.05580	2.16440	-1.67530
H	-1.34690	2.24100	-2.87700
H	-3.05180	1.30720	-4.28600
H	-5.47070	1.20540	-3.49690
H	-6.08700	2.12010	-1.34790
C	-3.49330	3.87360	1.30790
C	-2.06250	3.90160	0.90190

C	-1.09840	4.51030	1.85590	H	-0.04610	4.58540	1.61380
C	-1.51320	4.98130	3.03780	H	-0.79390	5.41740	3.71920
C	-2.93490	4.92660	3.43840	H	-3.22350	5.31540	4.40650
C	-3.86750	4.41310	2.63010	H	-4.90030	4.39020	2.95720

TS11-Z

(B3LYP/6-31G**:UFF) Energy = -1679.328596

(B3LYP/6-31G**:UFF) Free Energy = -1678.361160

M06-2X/6-31G** Derived free energy = -3522.031515

M06-2X/6-31G** Derived free energy in solution = -3522.049036

Number of Imaginary Frequencies = 1 (-255.24)

ONIOM (B3LYP/6-31G**:UFF) Geometry

C	2.64640	2.47390	0.10020	C	6.42460	0.35080	2.71270
C	1.02650	4.72180	-0.48870	H	4.23520	3.51190	2.06440
C	0.41140	3.50610	0.08590	H	6.05340	3.60760	3.63080
C	1.17470	2.42420	0.33430	H	7.49820	1.55200	4.06050
H	0.40310	5.58410	-0.69530	H	7.03200	-0.52300	2.91360
C	4.99140	-1.01480	1.09450	P	0.58620	0.01410	-0.20440
C	3.94280	-1.11790	0.26590	O	0.61090	1.28520	0.85260
C	3.11810	0.07900	-0.03810	O	2.07350	-0.02620	-0.92330
C	3.44910	1.28170	0.48070	O	0.38830	-1.20850	0.66530
H	5.59210	-1.88800	1.32250	O	-0.38540	0.27890	-1.33280
C	2.33470	4.74760	-0.77570	H	-2.04670	-0.02500	-1.32660
C	3.18990	3.56530	-0.48990	C	-3.76740	-0.58450	-0.32950
C	4.61610	3.64160	-0.89770	N	-3.05570	-0.27820	-1.46260
C	5.10740	4.75710	-1.45030	C	-7.94960	0.35410	0.15730
C	4.25810	5.94440	-1.68570	C	-7.29500	-0.46590	1.07830
C	2.95730	5.94420	-1.37560	C	-5.95770	-0.79920	0.88960
H	5.27740	2.79470	-0.77040	C	-5.24510	-0.33990	-0.23390
H	6.14960	4.79280	-1.74110	C	-5.91820	0.48130	-1.15130
H	4.70090	6.82770	-2.12780	C	-7.25230	0.82870	-0.95280
H	2.36360	6.82920	-1.57130	H	-8.99200	0.61970	0.30490
C	5.30940	0.27420	1.74090	H	-7.82400	-0.84300	1.94820
C	4.56320	1.37190	1.47440	H	-5.46770	-1.43420	1.62000
C	4.84560	2.63200	2.21560	H	-5.40290	0.85420	-2.02350
C	5.85250	2.68900	3.09490	H	-7.74610	1.47190	-1.67470
C	6.68420	1.49760	3.34920	C	-3.42130	-0.57200	-2.79090

C	-2.38680	-0.18710	-3.82330
H	-1.53110	0.33770	-3.39830
H	-2.87080	0.42230	-4.59140
H	-2.04840	-1.11100	-4.30200
O	-4.47250	-1.11760	-3.07310
C	-3.00530	-0.23000	0.93110
H	-1.95790	-0.52590	0.88620
H	-3.46330	-0.66120	1.81940
H	-3.03970	0.85770	1.03940
C	-2.58370	-3.05740	0.16270
H	-0.59090	-2.53460	-0.00130
C	-2.40410	-4.19700	4.30470
C	-3.45150	-4.62820	3.48920
C	-3.50020	-4.23960	2.15340
C	-2.49250	-3.42460	1.60020
C	-1.42760	-3.01480	2.42610
C	-1.40090	-3.38920	3.76800
H	-2.36780	-4.49290	5.34890
H	-4.22770	-5.27350	3.88940
H	-4.30290	-4.60680	1.52320
H	-0.64030	-2.37520	2.03820
H	-0.58800	-3.04230	4.39830
C	-3.81970	-2.78640	-0.41970
N	-1.40610	-2.90740	-0.52320
C	-1.21540	-3.05420	-1.91040
C	0.19160	-2.75450	-2.36910
H	0.21890	-1.72890	-2.74870
H	0.91390	-2.80670	-1.55740
H	0.45590	-3.44360	-3.17240
O	-2.09860	-3.40950	-2.67160
H	-4.70390	-3.01530	0.15810
H	-3.93820	-2.79290	-1.49370
C	-3.87220	3.54590	0.66830
C	-1.05430	3.48030	0.30400
H	-4.94790	3.56140	0.80070
C	2.87480	-4.95480	-1.40970
C	3.59090	-2.43200	-0.31660
H	2.59290	-5.91620	-1.82310
C	3.86190	-2.71720	-1.61370
C	3.48440	-4.04170	-2.17620
C	3.77670	-4.31900	-3.59640
C	4.37470	-3.39520	-4.35570
C	4.76670	-2.08040	-3.80510
C	4.53480	-1.75730	-2.52730
H	3.50480	-5.27200	-4.03440
H	4.58080	-3.61180	-5.39600
H	5.25600	-1.36580	-4.45460
H	4.85000	-0.78270	-2.17730
C	2.90450	-3.45470	0.53350
C	2.56230	-4.65190	0.00110
C	1.85290	-5.65790	0.82550
C	1.55530	-5.39290	2.10160
C	1.93420	-4.09760	2.69480
C	2.56310	-3.17660	1.95660
H	1.56970	-6.61090	0.39650
H	1.03380	-6.12700	2.70220
H	1.69530	-3.89170	3.72980
H	2.81260	-2.22770	2.41200
C	-3.31050	3.46590	-0.69380
C	-1.96990	3.41680	-0.87760
C	-1.43870	3.28580	-2.26300
C	-2.27340	3.24430	-3.30760
C	-3.73050	3.32060	-3.09690
C	-4.22320	3.42870	-1.85920
H	-0.37500	3.20940	-2.44410
H	-1.88150	3.14350	-4.31150
H	-4.39960	3.28460	-3.94680
H	-5.29480	3.48320	-1.71240
C	-3.05890	3.57630	1.73130
C	-1.58200	3.55320	1.55110
C	-0.74740	3.63410	2.77850
C	-1.31690	3.68550	3.98850
C	-2.78560	3.67510	4.15520
C	-3.60660	3.62800	3.10110
H	0.33300	3.65390	2.71800
H	-0.69060	3.74000	4.86980
H	-3.20140	3.71240	5.15390
H	-4.67820	3.62690	3.26160

TS12-*E*

(B3LYP/6-31G**:UFF) Energy = -1679.329081

(B3LYP/6-31G**:UFF) Free Energy = -1678.359072

M06-2X/6-31G** Derived free energy = -3522.031715

M06-2X/6-31G** Derived free energy in solution = -3522.049343

Number of Imaginary Frequencies = 1 (-243.02)

ONIOM (B3LYP/6-31G**:UFF) Geometry

C	-3.31140	0.85640	0.69540	P	-0.34060	0.13090	-0.34760
C	-2.96820	3.46460	1.74550	O	-1.42230	1.28310	-0.83840
C	-2.08280	2.98740	0.66170	O	-1.19770	-0.92140	0.61810
C	-2.23260	1.74000	0.17270	O	0.10990	-0.59560	-1.59510
H	-2.84080	4.46990	2.13030	O	0.70580	0.76290	0.53830
C	-3.71510	-3.10640	-0.96590	H	2.35240	1.31470	-0.15800
C	-2.54550	-2.70930	-0.44470	C	4.43600	1.18220	-0.15510
C	-2.39410	-1.32180	0.06930	N	3.21350	1.51970	-0.69500
C	-3.45820	-0.48820	0.08150	C	4.65000	0.68730	4.13370
H	-3.82880	-4.11780	-1.33930	C	3.41410	0.67120	3.49120
C	-3.90050	2.65410	2.26270	C	3.32430	0.81590	2.10770
C	-4.07270	1.27470	1.73500	C	4.48450	0.98570	1.32820
C	-5.07380	0.40820	2.40830	C	5.72900	0.98750	1.99300
C	-5.82180	0.88420	3.41090	C	5.81060	0.84380	3.37390
C	-5.67640	2.27410	3.89410	H	4.71180	0.57920	5.21260
C	-4.77450	3.10650	3.36290	H	2.50030	0.54710	4.06370
H	-5.20650	-0.62370	2.11110	H	2.33550	0.79670	1.66350
H	-6.54670	0.23720	3.88800	H	6.65010	1.11090	1.43780
H	-6.30890	2.61830	4.70230	H	6.78350	0.85910	3.85580
H	-4.68890	4.11510	3.74960	C	2.94940	1.88900	-2.03260
C	-4.84970	-2.16700	-1.06070	C	1.52610	2.31630	-2.28820
C	-4.73160	-0.90760	-0.58000	H	1.54090	3.14640	-2.99660
C	-5.86150	0.04390	-0.76700	H	1.00040	2.58820	-1.37790
C	-6.99240	-0.35630	-1.35940	H	0.97440	1.48410	-2.73350
C	-7.12520	-1.74270	-1.84540	O	3.78650	1.86090	-2.92050
C	-6.10880	-2.60080	-1.70920	C	5.65380	1.84330	-0.78360
H	-5.77960	1.07150	-0.43950	H	5.56680	1.88030	-1.86360
H	-7.81050	0.33970	-1.49300	H	6.57600	1.33290	-0.51280
H	-8.04410	-2.05990	-2.32120	H	5.70740	2.86940	-0.39820
H	-6.20980	-3.61390	-2.07810	C	3.69140	-1.46800	-0.77840

H	1.77360	-1.25710	-1.61570
C	2.52760	-3.12150	3.00260
C	3.87440	-3.08370	2.63360
C	4.24330	-2.56230	1.39990
C	3.27120	-2.10420	0.48420
C	1.91630	-2.22750	0.84080
C	1.55550	-2.68590	2.10380
H	2.23810	-3.49800	3.97930
H	4.63890	-3.44810	3.31260
H	5.29360	-2.53810	1.13580
H	1.13380	-1.95170	0.14940
H	0.50760	-2.68740	2.37880
C	4.92970	-0.82960	-0.87760
N	2.79130	-1.34880	-1.80960
C	3.15000	-1.30960	-3.18100
C	2.03140	-0.92230	-4.11850
H	2.26270	0.08470	-4.48240
H	2.04940	-1.59320	-4.97990
H	1.05060	-0.91170	-3.64370
O	4.27070	-1.57400	-3.57050
H	5.69340	-1.04290	-0.14340
H	5.27170	-0.53300	-1.85730
C	1.07910	5.52500	-0.83130
C	-1.00600	3.87640	0.17140
H	1.87830	6.14010	-1.22810
C	0.78810	-5.44910	-0.25470
C	-1.41780	-3.66380	-0.37510
H	1.64000	-6.11770	-0.21060
C	-1.14310	-4.33480	0.76930
C	0.01340	-5.26910	0.82190
C	0.30660	-5.96470	2.09080
C	-0.45990	-5.75810	3.16670
C	-1.61910	-4.84150	3.12360
C	-1.94640	-4.17550	2.00980
H	1.14960	-6.64170	2.16080
H	-0.23010	-6.27150	4.09150
H	-2.21450	-4.70840	4.01780
H	-2.80580	-3.51770	2.03590
C	-0.57850	-3.90680	-1.58860
C	0.48060	-4.74760	-1.51630
C	1.32370	-4.98690	-2.71030
C	1.02550	-4.39820	-3.87270
C	-0.14090	-3.50120	-3.96880
C	-0.89260	-3.25540	-2.89000
H	2.18190	-5.64430	-2.64760
H	1.64110	-4.57480	-4.74520
H	-0.37570	-3.02650	-4.91280
H	-1.73140	-2.57840	-2.98250
C	-0.15920	5.34880	-1.61620
C	-1.15810	4.56310	-1.14810
C	-2.39190	4.40210	-1.96620
C	-2.50260	5.01250	-3.15170
C	-1.40400	5.85670	-3.65750
C	-0.29390	6.01910	-2.93020
H	-3.21420	3.79320	-1.61440
H	-3.40110	4.89090	-3.74280
H	-1.50030	6.34590	-4.61820
H	0.50810	6.64090	-3.30830
C	1.22070	4.91300	0.35090
C	0.12900	4.05950	0.89170
C	0.36450	3.40990	2.20800
C	1.51740	3.59180	2.86200
C	2.60410	4.42170	2.30200
C	2.46980	5.04510	1.12710
H	-0.38610	2.77380	2.65860
H	1.66690	3.11130	3.82020
H	3.52760	4.52240	2.85760
H	3.28880	5.64400	0.74700

TS12-*Z*

(B3LYP/6-31G**:UFF) Energy = -1679.324551

(B3LYP/6-31G**:UFF) Free Energy = -1678.357906

M06-2X/6-31G** Derived free energy = -3522.025866

M06-2X/6-31G** Derived free energy in solution = -3522.044371

Number of Imaginary Frequencies = 1 (-256.17)

ONIOM (B3LYP/6-31G**:UFF) Geometry

C	2.80940	2.19700	-0.57830
C	1.40280	4.40090	-1.67000
C	0.75530	3.54950	-0.65080
C	1.40710	2.47860	-0.15610
H	0.86380	5.25200	-2.07000
C	4.86320	-1.20090	1.11590
C	3.68440	-1.36050	0.49750
C	2.95160	-0.17630	-0.01680
C	3.51000	1.05080	0.05720
H	5.40200	-2.06350	1.49090
C	2.63500	4.11230	-2.10790
C	3.37420	2.94620	-1.55600
C	4.70740	2.64780	-2.13950
C	5.22980	3.43850	-3.08450
C	4.50540	4.62370	-3.59100
C	3.28720	4.94170	-3.14030
H	5.27200	1.78040	-1.82390
H	6.20270	3.20380	-3.49710
H	4.97070	5.23930	-4.35010
H	2.78210	5.81220	-3.54170
C	5.42930	0.14980	1.30810
C	4.78310	1.23420	0.81920
C	5.33560	2.58860	1.09590
C	6.47780	2.72670	1.77890
C	7.19400	1.53660	2.27600
C	6.69600	0.31480	2.05810
H	4.81700	3.47660	0.76040
H	6.87540	3.71370	1.97730
H	8.11870	1.65630	2.82570
H	7.22210	-0.55350	2.43490
P	0.45240	0.15240	0.26230
O	0.81050	1.68110	0.78990
O	1.74430	-0.34590	-0.64830
O	0.30900	-0.69420	1.50260
O	-0.70040	0.20220	-0.71990
H	-2.30500	0.21630	-0.19840
C	-4.05860	-0.74860	-0.65950
N	-3.29800	0.08870	0.11640
C	-8.30910	-0.14020	-1.10330
C	-7.71870	-1.32620	-1.54190
C	-6.35600	-1.54010	-1.35530
C	-5.55330	-0.58560	-0.70720
C	-6.15970	0.60770	-0.28270
C	-7.52040	0.82700	-0.48140
H	-9.37130	0.02980	-1.25050
H	-8.31750	-2.08560	-2.03540
H	-5.91960	-2.46330	-1.71970
H	-5.56750	1.37740	0.19120
H	-7.96210	1.76080	-0.14720
C	-3.55320	0.45360	1.45230
C	-2.49710	1.35150	2.05680
H	-1.86610	1.82430	1.30790
H	-1.83860	0.75960	2.69680
H	-2.99150	2.10160	2.67700
O	-4.53030	0.06430	2.06630
C	-3.43660	-1.01800	-2.01880
H	-2.34920	-0.97670	-1.98030
H	-3.75500	-1.97940	-2.42290
H	-3.78930	-0.24430	-2.70870
C	-2.59590	-2.85770	0.46440
H	-0.93490	-2.00660	1.41250
C	-0.54740	-4.94890	-2.67620
C	-0.09050	-3.69180	-2.27890
C	-0.70970	-3.00940	-1.23460
C	-1.83930	-3.55820	-0.60780
C	-2.30370	-4.82010	-1.02240
C	-1.65010	-5.51700	-2.03650
H	-0.04870	-5.48210	-3.48010
H	0.75600	-3.23050	-2.77780
H	-0.37090	-2.01520	-0.97730

H	-3.16040	-5.26560	-0.52640
H	-2.00730	-6.49960	-2.32970
C	-3.97350	-2.67820	0.33540
N	-1.91210	-2.34210	1.52960
C	-2.41240	-2.31450	2.86120
C	-1.44920	-1.75480	3.88390
H	-0.56580	-1.29630	3.43930
H	-1.99480	-1.02560	4.48860
H	-1.15780	-2.56830	4.55560
O	-3.50030	-2.76090	3.15990
H	-4.47440	-3.27380	-0.41580
H	-4.55380	-2.37160	1.19380
C	-3.29850	4.45390	0.53780
C	-0.62850	3.86600	-0.23340
H	-4.31990	4.66720	0.83100
C	2.04060	-5.32150	-0.01990
C	3.12920	-2.71910	0.31230
H	1.62020	-6.31300	-0.14200
C	3.27190	-3.37510	-0.86480
C	2.71720	-4.74610	-1.02140
C	2.90580	-5.44550	-2.30810
C	3.54890	-4.84540	-3.31490
C	4.09600	-3.47920	-3.17500
C	3.97440	-2.78780	-2.03550
H	2.51430	-6.44600	-2.44880
H	3.67320	-5.36750	-4.25490
H	4.60950	-3.03230	-4.01660
H	4.39880	-1.79300	-1.98890
C	2.40460	-3.38180	1.43960
C	1.86970	-4.61330	1.26370
C	1.12690	-5.26640	2.36600
C	1.00250	-4.65430	3.54790
C	1.60500	-3.32500	3.75870
C	2.25980	-2.71670	2.76360
H	0.68370	-6.24350	2.21950
H	0.45970	-5.13220	4.35310
H	1.50450	-2.83680	4.71950
H	2.67970	-1.73510	2.93930
C	-3.04100	3.74670	-0.73190
C	-1.77170	3.46410	-1.11010
C	-1.54730	2.73420	-2.38840
C	-2.58440	2.36540	-3.14860
C	-3.96260	2.67890	-2.72730
C	-4.18050	3.33520	-1.58370
H	-0.54660	2.48340	-2.71440
H	-2.41550	1.82860	-4.07310
H	-4.79700	2.37140	-3.34440
H	-5.19560	3.55940	-1.28030
C	-2.27870	4.81660	1.32600
C	-0.87690	4.51580	0.92980
C	0.20060	4.95270	1.85530
C	-0.09680	5.57600	3.00170
C	-1.49500	5.85840	3.39020
C	-2.52090	5.50700	2.60820
H	1.24240	4.77620	1.61990
H	0.70280	5.88470	3.66280
H	-1.68780	6.36300	4.32830
H	-3.53230	5.73140	2.92550

Additional catalysts

$4\text{-}NO_2C_6H_4$ derived CPA *Type II E* TS

(B3LYP/6-31G**:UFF) Energy = -1679.378801

(B3LYP/6-31G**:UFF) Free Energy = -1678.609195

M06-2X/6-31G** Derived free energy = -3316.670324

M06-2X/6-31G** Derived free energy in solution = -3316.692600

Number of Imaginary Frequencies = 1 (-208.75)

ONIOM (B3LYP/6-31G**:UFF) Geometry

C	-4.00800	-0.52280	0.00560
C	-5.05220	1.90250	1.03200
C	-3.78390	1.92240	0.26670
C	-3.27390	0.76100	-0.19630
H	-5.46240	2.83370	1.40550
C	-2.12690	-4.01660	-1.70550
C	-1.46830	-3.26520	-0.80850
C	-2.13770	-2.07720	-0.21810
C	-3.40400	-1.75700	-0.56570
H	-1.64890	-4.88400	-2.14630
C	-5.68770	0.74620	1.26800
C	-5.13650	-0.53450	0.75240
C	-5.85420	-1.78240	1.11670
C	-6.98210	-1.73360	1.83540
C	-7.54860	-0.44990	2.30210
C	-6.94430	0.71470	2.04280
H	-5.47390	-2.75030	0.81730
H	-7.49200	-2.65420	2.08940
H	-8.46790	-0.45820	2.87360
H	-7.38370	1.63480	2.40930
C	-3.49760	-3.65840	-2.12600
C	-4.10770	-2.56690	-1.60670
C	-5.44730	-2.17550	-2.12630
C	-6.05690	-2.91960	-3.05630
C	-5.40010	-4.12860	-3.58720
C	-4.18490	-4.47700	-3.15180
H	-5.94000	-1.28080	-1.77120
H	-7.03060	-2.63020	-3.43010
H	-5.90040	-4.72660	-4.33800
H	-3.70420	-5.35960	-3.55530
P	-0.83720	0.07680	0.00840
O	-2.07170	0.74590	-0.85990
O	-1.46000	-1.29410	0.68270
C	-1.63700	5.65240	-0.16930
C	-1.86000	4.87000	-1.24100
C	-2.60470	3.60210	-1.10160
C	-3.06120	3.20710	0.10110
C	-2.82870	4.07500	1.28050
C	-2.15650	5.22530	1.15420
H	-1.49390	5.14850	-2.22050
H	-2.77750	3.00060	-1.98560
H	-3.18780	3.77240	2.25640
H	-1.98880	5.83830	2.03020
C	2.63580	-4.10520	0.25150
C	2.27980	-3.97190	-1.03910
C	0.87370	-3.70440	-1.40460
C	-0.06660	-3.57980	-0.45120
C	0.32300	-3.72340	0.97150
C	1.59360	-3.98280	1.30150
H	3.01370	-4.04880	-1.83050
H	0.61960	-3.58380	-2.45080
H	-0.42420	-3.63690	1.75060
H	1.85350	-4.10710	2.34310
O	0.22720	-0.26650	-1.00590
O	-0.47990	0.96670	1.18270
H	1.15190	1.27040	1.40340
N	-0.89780	6.87870	-0.29310
O	-0.66350	7.66330	0.81240
O	-0.39560	7.28180	-1.50960
N	4.00410	-4.35030	0.61690
O	4.35340	-4.50710	1.93810
O	4.99050	-4.41640	-0.34070
C	3.10170	0.69260	1.85860
N	2.15490	1.57900	1.43690
C	1.87190	-2.37200	4.64570
C	3.19480	-2.30260	4.20380
C	3.57400	-1.33670	3.27910
C	2.64720	-0.39870	2.77810
C	1.31370	-0.49140	3.22350
C	0.93740	-1.47000	4.14170
H	1.57420	-3.12860	5.36520
H	3.93310	-3.00760	4.57320

H	4.60380	-1.32810	2.94300	C	5.08530	0.39180	-1.36890
H	0.55000	0.18210	2.85520	C	5.96170	-0.69920	-1.48430
H	-0.09880	-1.51850	4.46090	C	7.31640	-0.49700	-1.74490
C	2.36230	2.85080	0.85320	H	8.87130	0.96050	-2.06440
C	1.08790	3.64360	0.66290	H	7.33770	2.90260	-1.82960
H	0.21810	3.00860	0.48890	H	4.93410	2.54450	-1.39340
H	0.89930	4.21720	1.57840	H	5.57050	-1.70830	-1.40180
H	1.24110	4.35370	-0.15080	H	7.97800	-1.35130	-1.85220
O	3.45990	3.30080	0.57490	C	3.36160	-0.70660	0.04400
C	4.52110	1.18070	2.03080	N	2.61840	0.72600	-1.66790
H	5.19890	0.35930	2.24860	C	2.61180	1.40460	-2.90550
H	4.87860	1.73490	1.17150	C	1.22420	1.86250	-3.30830
H	4.53430	1.86190	2.89190	H	0.46220	1.11790	-3.06530
C	3.66510	0.12560	-1.02650	H	0.97100	2.77690	-2.75830
H	1.67140	0.41760	-1.34040	H	1.22950	2.08930	-4.37440
C	7.81530	0.79940	-1.86820	O	3.60600	1.60890	-3.57310
C	6.95260	1.89150	-1.73960	H	4.15880	-1.29310	0.47980
C	5.59590	1.69430	-1.50280	H	2.34790	-1.07710	0.14200

4-$NO_2C_6H_4$ derived CPA *Type I E* TS

(B3LYP/6-31G**:UFF) Energy = -1679.375068

(B3LYP/6-31G**:UFF) Free Energy = -1678.601765

M06-2X/6-31G** Derived free energy = -3316.665299

M06-2X/6-31G** Derived free energy in solution = -3316.688535

Number of Imaginary Frequencies = 1 (-253.13)

ONIOM (B3LYP/6-31G**:UFF) Geometry

C	3.14330	-1.87390	0.33690	H	4.89810	2.76400	-1.80030
C	1.96870	-4.24870	1.33660	C	3.21390	-3.91330	1.70160
C	1.19020	-3.37070	0.43450	C	3.83250	-2.65900	1.19760
C	1.73870	-2.22580	-0.01970	C	5.18600	-2.31460	1.70200
H	1.51840	-5.16280	1.70640	C	5.83020	-3.13760	2.53810
C	4.56000	1.83430	-1.35660	C	5.22320	-4.40510	2.99860
C	3.49330	1.82480	-0.54440	C	3.99620	-4.77190	2.61300
C	3.02310	0.54240	0.04060	H	5.66970	-1.39140	1.41070
C	3.68850	-0.60640	-0.21160	H	6.81710	-2.87000	2.89350

H	5.78450	-5.04300	3.66930
H	3.58020	-5.70330	2.97820
C	5.27370	0.58010	-1.67360
C	4.85460	-0.59470	-1.14700
C	5.53740	-1.85250	-1.55830
C	6.57680	-1.81470	-2.40010
C	7.04830	-0.52570	-2.93990
C	6.42870	0.60960	-2.60080
H	5.19760	-2.81240	-1.19450
H	7.06920	-2.73220	-2.69570
H	7.89090	-0.50300	-3.61880
H	6.77400	1.55100	-3.00990
P	0.54880	0.02290	-0.00010
O	1.00910	-1.36210	-0.80310
O	1.89560	0.52060	0.82680
C	-2.90490	-4.29890	-0.54160
C	-2.53930	-4.08670	0.73610
C	-1.12930	-3.80420	1.07420
C	-0.20260	-3.72890	0.10640
C	-0.59250	-3.99130	-1.29740
C	-1.86510	-4.27360	-1.60210
H	-3.27020	-4.09520	1.53400
H	-0.86120	-3.62110	2.10720
H	0.15280	-3.95570	-2.08230
H	-2.13300	-4.45850	-2.63420
C	1.28860	5.45680	0.18430
C	1.94810	4.84810	1.18720
C	2.69820	3.59830	0.94290
C	2.75380	3.07440	-0.29100
C	2.05180	3.75340	-1.40460
C	1.35240	4.87180	-1.17920
H	1.92660	5.24900	2.19210
H	3.20250	3.11460	1.77080
H	2.08680	3.33450	-2.40270
H	0.83520	5.34710	-2.00240
O	0.21080	1.03380	-1.07200
O	-0.48920	-0.33110	1.03780
H	-2.30240	-0.57580	0.94260
N	-4.28140	-4.52560	-0.88610
O	-5.25930	-4.54020	0.08250
O	-4.65060	-4.68550	-2.20170
N	0.52840	6.65300	0.42170
O	-0.14860	7.26210	-0.60950
O	0.44250	7.19740	1.68300
C	-4.19460	-0.29450	0.12430
N	-3.31840	-0.63410	1.13480
C	-2.86510	-1.01970	-3.92260
C	-1.99380	-1.21800	-2.85570
C	-2.42070	-0.99450	-1.54720
C	-3.72070	-0.53730	-1.27390
C	-4.58460	-0.32990	-2.36990
C	-4.16740	-0.57730	-3.67110
H	-2.53690	-1.20150	-4.94160
H	-0.97300	-1.54620	-3.01930
H	-1.72540	-1.19880	-0.74590
H	-5.58720	0.05030	-2.21920
H	-4.85610	-0.40810	-4.49280
C	-3.60520	-0.80240	2.50480
C	-2.42350	-1.27460	3.32430
H	-2.54080	-2.35050	3.49660
H	-1.46380	-1.08600	2.84140
H	-2.46870	-0.78210	4.29700
O	-4.70670	-0.62280	2.99810
C	-5.67110	-0.53110	0.38100
H	-6.28330	-0.09960	-0.40750
H	-5.83700	-1.61600	0.38130
H	-5.98060	-0.15260	1.34830
C	-2.98380	2.25970	0.20510
H	-1.19090	1.97740	-0.82340
C	-1.16950	2.80770	4.06350
C	-2.53240	2.52730	3.95970
C	-3.11260	2.34270	2.71010
C	-2.35040	2.44760	1.52670
C	-0.97920	2.75250	1.65310
C	-0.39810	2.91200	2.90600
H	-0.71010	2.93920	5.03860
H	-3.14590	2.44130	4.85110
H	-4.16450	2.09410	2.67060
H	-0.35360	2.84050	0.77420
H	0.66600	3.09990	2.98430

C	-4.32980	1.90400	0.08120
N	-2.17260	2.33000	-0.89890
C	-2.56450	2.67410	-2.20790
C	-1.40670	2.72150	-3.18160
H	-0.95030	3.71740	-3.13670
H	-0.63820	1.98590	-2.93760
H	-1.79730	2.57160	-4.18850
O	-3.70500	2.97070	-2.51770
H	-4.98630	2.04510	0.92830
H	-4.78430	1.99580	-0.89250

4-$NO_2C_6H_4$ derived CPA *Type II Z* TS

(B3LYP/6-31G**:UFF) Energy = -1679.373222

(B3LYP/6-31G**:UFF) Free Energy = -1678.599869

M06-2X/6-31G** Derived free energy = -3316.663390

M06-2X/6-31G** Derived free energy in solution = -3316.689204

Number of Imaginary Frequencies = 1 (-241.69)

ONIOM (B3LYP/6-31G**:UFF) Geometry

C	-3.77420	-0.95800	0.59670
C	-4.92910	1.48360	1.45230
C	-3.90300	1.49500	0.38590
C	-3.33040	0.33470	0.00290
H	-5.37980	2.41830	1.76550
C	-1.83490	-4.57980	-0.73330
C	-1.10630	-3.58350	-0.20140
C	-1.78100	-2.31600	0.19040
C	-3.12550	-2.20260	0.10510
H	-1.36270	-5.51490	-1.01190
C	-5.29560	0.33160	2.03010
C	-4.68230	-0.95330	1.60130
C	-5.08430	-2.17910	2.33740
C	-6.00170	-2.11930	3.31010
C	-6.63890	-0.84230	3.69700
C	-6.30900	0.30970	3.10360
H	-4.63560	-3.13720	2.11050
H	-6.28150	-3.02400	3.83450
H	-7.38140	-0.84310	4.48470
H	-6.79000	1.22770	3.42010
C	-3.29050	-4.42600	-0.93390
C	-3.91540	-3.29050	-0.54580
C	-5.37030	-3.13690	-0.82230
C	-6.05590	-4.12580	-1.40760
C	-5.37240	-5.37390	-1.79580
C	-4.06110	-5.51630	-1.57600
H	-5.88920	-2.22340	-0.56500
H	-7.11350	-4.00920	-1.60620
H	-5.93400	-6.17040	-2.26670
H	-3.56470	-6.43220	-1.87200
P	-0.83880	-0.00550	-0.32630
O	-2.33410	0.32900	-0.94100
O	-1.03650	-1.28380	0.70600
C	-2.59970	5.27590	-1.24320
C	-3.05670	4.30770	-2.05890
C	-3.50700	3.01340	-1.50720
C	-3.47350	2.79140	-0.18180
C	-2.99800	3.86490	0.72140
C	-2.57900	5.03330	0.22140
H	-3.08620	4.45000	-3.13120
H	-3.86200	2.24860	-2.18720
H	-2.96790	3.69960	1.79130
H	-2.22060	5.79900	0.89710
C	3.15050	-4.01990	0.43720

C	2.60060	-4.36920	-0.73910	C	0.75780	4.07920	1.31800
C	1.14710	-4.22540	-0.96090	H	0.66670	4.61290	0.36630
C	0.35110	-3.75460	0.01610	H	-0.06550	3.37090	1.41400
C	0.95060	-3.40900	1.32820	H	0.72470	4.82510	2.11570
C	2.26760	-3.53770	1.52770	O	3.16310	4.02920	1.38230
H	3.21240	-4.74050	-1.55050	C	2.72240	-0.28340	0.97760
H	0.74120	-4.47870	-1.93270	H	1.95380	-0.39940	0.21440
H	0.32260	-3.06160	2.13890	H	3.57500	-0.91410	0.73040
H	2.68390	-3.27910	2.49270	H	2.30790	-0.63610	1.92970
O	0.00370	-0.39730	-1.51990	C	3.26600	1.05160	-1.62150
O	-0.37800	1.13200	0.55970	H	1.25440	0.72950	-2.02000
H	1.13100	1.59870	0.98870	C	4.63110	-2.63180	-3.38610
N	-2.13050	6.52620	-1.77420	C	3.26430	-2.37230	-3.28620
O	-1.65350	7.50350	-0.93150	C	2.80220	-1.19990	-2.69350
O	-2.12730	6.76190	-3.13030	C	3.71220	-0.24600	-2.19360
N	4.57090	-4.10120	0.64030	C	5.09080	-0.51960	-2.30900
O	5.12560	-3.70020	1.83370	C	5.54390	-1.69900	-2.89170
O	5.40620	-4.52290	-0.36930	H	4.98250	-3.54940	-3.84830
C	3.13780	1.15780	1.17870	H	2.54450	-3.09300	-3.66180
N	2.06860	2.00630	1.24460	H	1.73230	-1.05900	-2.58950
C	6.48540	1.37350	3.88580	H	5.82000	0.21260	-1.98210
C	6.56850	0.60850	2.72110	H	6.61120	-1.87950	-2.97630
C	5.50130	0.57680	1.82910	C	4.06720	1.78230	-0.75110
C	4.33460	1.32630	2.06630	N	1.98870	1.45810	-1.92630
C	4.26010	2.07970	3.24870	C	1.51860	2.78010	-1.99490
C	5.32390	2.09970	4.14770	C	0.04760	2.88180	-2.32600
H	7.31550	1.39740	4.58530	H	-0.28510	2.10990	-3.02270
H	7.46260	0.03020	2.50890	H	-0.51960	2.74530	-1.39950
H	5.58190	-0.03290	0.93520	H	-0.14590	3.87540	-2.73060
H	3.36390	2.63940	3.47860	O	2.22470	3.75740	-1.81240
H	5.24050	2.68670	5.05720	H	5.09580	1.48080	-0.62020
C	2.11500	3.41290	1.33300	H	3.85530	2.82420	-0.56040

4-$NO_2C_6H_4$ derived CPA *Type I Z* TS

(B3LYP/6-31G**:UFF) Energy = -1679.373327

(B3LYP/6-31G**:UFF) Free Energy = -1678.602467

M06-2X/6-31G** Derived free energy = -3316.662067

M06-2X/6-31G** Derived free energy in solution = -3316.688388

Number of Imaginary Frequencies = 1 (-214.55)

ONIOM (B3LYP/6-31G**:UFF) Geometry

C	-3.20450	-1.99720	-0.67760	C	0.23920	-3.67840	-0.34830
C	-1.81270	-4.20390	-1.78050	C	0.59120	-3.78930	1.08860
C	-1.14900	-3.35970	-0.75900	C	1.84340	-4.08260	1.45780
C	-1.80470	-2.29260	-0.25490	H	3.32230	-4.34830	-1.63490
H	-1.28850	-5.06290	-2.18340	H	0.96490	-3.81590	-2.32920
C	-5.17850	1.45140	1.01290	H	-0.16990	-3.65580	1.84720
C	-4.00670	1.59670	0.36960	H	2.07440	-4.18350	2.50910
C	-3.31040	0.38940	-0.15340	C	-2.24860	5.51060	-0.21230
C	-3.88540	-0.82990	-0.05560	C	-2.74940	5.16100	0.98660
H	-5.70940	2.31900	1.38690	C	-3.35340	3.82780	1.18770
C	-3.04800	-3.91130	-2.20770	C	-3.41930	2.94650	0.17250
C	-3.77850	-2.74290	-1.65140	C	-2.88470	3.34000	-1.15530
C	-5.11270	-2.43640	-2.22840	C	-2.32790	4.54320	-1.33460
C	-5.64490	-3.22440	-3.17020	H	-2.70480	5.83950	1.82830
C	-4.93020	-4.41410	-3.67960	H	-3.71850	3.56700	2.17340
C	-3.71130	-4.73840	-3.23550	H	-2.95470	2.66000	-1.99490
H	-5.67110	-1.56640	-1.90920	H	-1.94300	4.80710	-2.31120
H	-6.61880	-2.98440	-3.57740	O	-0.62140	0.87870	1.31720
H	-5.40320	-5.02760	-4.43560	O	0.30690	-0.09120	-0.93380
H	-3.21340	-5.61200	-3.63930	H	1.66610	0.70070	-1.23350
C	-5.76660	0.11160	1.21840	N	4.25520	-4.54760	0.84590
C	-5.14910	-0.98630	0.72500	O	5.25520	-4.70940	-0.08600
C	-5.72480	-2.32880	1.01320	O	4.58000	-4.60420	2.18150
C	-6.86020	-2.44230	1.71200	N	-1.63640	6.79580	-0.40930
C	-7.54570	-1.23690	2.21460	O	-1.54300	7.70040	0.62400
C	-7.02650	-0.02610	1.98570	O	-1.10980	7.13390	-1.63450
H	-5.22940	-3.22810	0.67290	C	3.69300	0.52880	-0.97620
H	-7.27470	-3.42070	1.91860	N	2.58340	1.19280	-1.40760
H	-8.46530	-1.33630	2.77690	C	7.53940	0.86070	-2.89990
H	-7.53060	0.85360	2.36630	C	6.40870	1.41990	-3.49430
P	-0.80730	0.02170	0.08330	C	5.17200	1.36130	-2.85610
O	-1.19830	-1.47460	0.66560	C	5.03690	0.73800	-1.60450
O	-2.10750	0.52750	-0.79920	C	6.18210	0.15400	-1.03170
C	2.90250	-4.28340	0.43860	C	7.41910	0.22290	-1.66430
C	2.57630	-4.20560	-0.86410	H	8.50330	0.91460	-3.39670
C	1.19150	-3.89300	-1.27280	H	6.48440	1.90540	-4.46230

H	4.30950	1.79080	-3.34530	C	1.69970	-0.51920	3.02270
H	6.11250	-0.35750	-0.07820	C	1.66500	-1.60350	3.89630
H	8.28800	-0.22640	-1.19330	H	2.81150	-3.00830	5.06540
C	2.47040	2.55620	-1.74720	H	5.00050	-2.06290	4.34970
C	1.09050	2.94760	-2.23650	H	5.08110	-0.14560	2.83370
H	1.11250	2.99350	-3.33130	H	0.75870	-0.12050	2.65830
H	0.31530	2.23900	-1.94030	H	0.70330	-2.01050	4.19220
H	0.86200	3.95050	-1.87110	C	4.14810	1.53170	0.99390
O	3.39900	3.33760	-1.66370	N	1.87080	1.97960	1.58490
C	3.39840	-0.90120	-0.57560	C	1.83720	3.38080	1.43440
H	4.14130	-1.29440	0.11790	C	0.43350	3.94790	1.45250
H	3.43850	-1.51130	-1.48590	H	0.43840	4.89240	0.90730
H	2.40220	-1.00090	-0.14570	H	-0.29990	3.24970	1.04490
C	3.00700	1.21600	1.71980	H	0.14880	4.15780	2.48990
H	0.93650	1.52270	1.64570	O	2.83230	4.07530	1.35880
C	2.84570	-2.16690	4.37980	H	5.07360	1.02710	1.22650
C	4.07330	-1.64120	3.97330	H	4.23110	2.50490	0.53130
C	4.11560	-0.56150	3.09740				
C	2.93120	0.02350	2.60300				

$SiPh_3$ derived CPA *Type I Z* TS

(B3LYP/6-31G**:UFF) Energy = -1679.358711

(B3LYP/6-31G**:UFF) Free Energy = -1678.228373

M06-2X/6-31G** Derived free energy = -4412.704864

M06-2X/6-31G** Derived free energy in solution = -4412.721814

Number of Imaginary Frequencies = 1 (-236.41)

ONIOM (B3LYP/6-31G**:UFF) Geometry

C	0.84740	3.13330	0.38660	H	5.47900	0.78580	1.75660
C	-1.38330	4.60350	-0.54770	C	-0.18760	5.18910	-0.47630
C	-1.55350	3.17940	-0.18530	C	0.99210	4.42390	-0.01190
C	-0.48260	2.44630	0.21290	C	2.30500	5.12750	-0.04470
H	-2.22880	5.17910	-0.89820	C	2.38650	6.41040	-0.41590
C	4.49360	1.18480	1.54630	C	1.19000	7.17580	-0.81970
C	3.79910	0.75210	0.48290	C	-0.01820	6.60670	-0.85620
C	2.45070	1.31470	0.21460	H	3.22310	4.61360	0.20540
C	2.01450	2.37980	0.90820	H	3.35200	6.89970	-0.43420

H	1.30040	8.21460	-1.10370
H	-0.87250	7.19520	-1.16970
C	3.93990	2.23360	2.42780
C	2.75860	2.81820	2.12480
C	2.20390	3.85090	3.03970
C	2.88420	4.22040	4.13110
C	4.18670	3.59860	4.43970
C	4.68760	2.65490	3.63470
H	1.24330	4.30670	2.83790
H	2.47770	4.97240	4.79500
H	4.73270	3.90490	5.32270
H	5.64140	2.19950	3.87030
P	0.38360	-0.06010	0.04630
O	-0.67160	1.12550	0.57310
O	1.62140	0.72640	-0.70340
O	0.89180	-0.75420	1.29550
O	-0.31270	-0.89240	-1.00060
H	-1.55850	-2.01780	-0.83670
C	-3.18700	-2.76970	0.18390
N	-2.24670	-2.81020	-0.80640
C	-7.19240	-4.22000	-0.46110
C	-6.75220	-3.92930	0.83110
C	-5.44220	-3.51310	1.04840
C	-4.53340	-3.39620	-0.01850
C	-4.99460	-3.68210	-1.31470
C	-6.30900	-4.08640	-1.53180
H	-8.21590	-4.53960	-0.63180
H	-7.43090	-4.01990	1.67360
H	-5.12970	-3.28220	2.06000
H	-4.33550	-3.56110	-2.16330
H	-6.64150	-4.29220	-2.54460
C	-1.88990	-3.93440	-1.57990
C	-0.87930	-3.62100	-2.66100
H	-0.26040	-4.50310	-2.82910
H	-0.26740	-2.75250	-2.41010
H	-1.41890	-3.40310	-3.59030
O	-2.37300	-5.03550	-1.39200
C	-3.22520	-1.44520	0.90120
H	-2.23750	-1.00770	1.02820
H	-3.72730	-1.51250	1.86310

H	-3.80660	-0.77430	0.26910
C	-1.14660	-3.70030	1.93080
H	0.15160	-2.09810	2.17010
C	1.64500	-6.18270	-0.17280
C	1.81260	-4.79900	-0.18420
C	0.93630	-3.96140	0.50410
C	-0.15750	-4.51800	1.19160
C	-0.32080	-5.91820	1.19390
C	0.57650	-6.74360	0.52850
H	2.34670	-6.81940	-0.70340
H	2.64120	-4.36990	-0.73010
H	1.09020	-2.88660	0.48380
H	-1.14210	-6.36040	1.74570
H	0.44080	-7.82020	0.55580
C	-2.49110	-4.05340	1.88930
N	-0.68290	-2.57410	2.56300
C	-1.32300	-1.84990	3.58410
C	-0.52790	-0.65380	4.05640
H	-1.02660	-0.22940	4.92670
H	0.49720	-0.93730	4.31140
H	-0.45490	0.08070	3.24860
O	-2.40570	-2.16220	4.05350
H	-2.75390	-4.99940	1.43640
H	-3.15380	-3.63730	2.62960
Si	-3.26880	2.44620	-0.38520
Si	4.60080	-0.45800	-0.68680
C	4.87040	-4.68270	1.33680
C	4.25840	-3.67840	1.97500
C	4.15600	-2.34830	1.33890
C	4.67750	-2.13910	0.11720
C	5.33300	-3.26470	-0.59400
C	5.43190	-4.46620	-0.01170
H	4.94330	-5.65770	1.80070
H	3.83540	-3.83770	2.95840
H	3.65880	-1.55620	1.88110
H	5.73810	-3.11720	-1.58740
H	5.91290	-5.28420	-0.53200
C	2.06260	-0.62920	-4.63810
C	2.62550	0.52120	-4.25400
C	3.45230	0.56970	-3.03010

C	3.64950	-0.53990	-2.29510
C	3.03040	-1.81300	-2.73830
C	2.27790	-1.85380	-3.84450
H	1.44890	-0.66470	-5.52880
H	2.46590	1.42290	-4.83100
H	3.84600	1.52440	-2.71740
H	3.17160	-2.71660	-2.17170
H	1.82410	-2.78480	-4.15890
C	8.92210	1.21920	-1.45260
C	7.85220	1.99530	-1.65430
C	6.49520	1.46360	-1.41200
C	6.32200	0.19200	-1.00980
C	7.51810	-0.65240	-0.75930
C	8.74670	-0.16610	-0.97560
H	9.91710	1.60760	-1.62740
H	7.97750	3.01590	-1.99200
H	5.65380	2.12930	-1.53860
H	7.41850	-1.65810	-0.38210
H	9.61590	-0.78360	-0.78900
C	-6.28780	5.80210	-1.63950
C	-5.79850	5.76870	-0.39450
C	-4.84560	4.71010	-0.00170
C	-4.46810	3.77540	-0.89050
C	-5.00950	3.82100	-2.27100
C	-5.87250	4.78200	-2.62340
H	-6.98570	6.57710	-1.92890
H	-6.09940	6.51650	0.32770
H	-4.45820	4.70210	1.00970
H	-4.70460	3.08950	-3.00700
H	-6.26420	4.81710	-3.63180
C	-4.88980	0.81820	3.71210
C	-5.67840	0.82870	2.63260
C	-5.17140	1.35980	1.35070
C	-3.90380	1.79740	1.24700
C	-3.05250	1.82820	2.46390
C	-3.51970	1.35880	3.62730
H	-5.25840	0.42840	4.65200
H	-6.68870	0.44570	2.69410
H	-5.84330	1.39060	0.50610
H	-2.06000	2.25210	2.43620
H	-2.90080	1.39150	4.51400
C	-3.20200	-0.82980	-3.75660
C	-2.10740	-0.10230	-3.51140
C	-2.12990	0.94010	-2.46400
C	-3.23660	1.14310	-1.72720
C	-4.46150	0.36510	-2.04380
C	-4.43990	-0.57520	-2.99600
H	-3.18590	-1.59800	-4.51870
H	-1.19890	-0.28180	-4.07200
H	-1.23320	1.52150	-2.30440
H	-5.38360	0.55220	-1.51630
H	-5.33340	-1.14450	-3.21740

$SiPh_3$ derived CPA *Type II Z* TS

(B3LYP/6-31G**:UFF) Energy = -1679.370756

(B3LYP/6-31G**:UFF) Free Energy = -1678.232211

M06-2X/6-31G** Derived free energy = -4412.704122

M06-2X/6-31G** Derived free energy in solution = -4412.721398

Number of Imaginary Frequencies = 1 (-217.46)

ONIOM (B3LYP/6-31G**:UFF) Geometry

C	-2.74650	1.95650	-0.88740
C	-4.88900	0.27800	-1.64900
C	-4.06250	-0.07650	-0.47310
C	-2.99810	0.69070	-0.15970
H	-5.72030	-0.35860	-1.92890
C	0.18050	4.80680	0.46260
C	0.57230	3.56210	0.15120
C	-0.45060	2.51950	-0.16950
C	-1.74870	2.89270	-0.31060
H	0.90280	5.56850	0.72330
C	-4.59430	1.35820	-2.38480
C	-3.46970	2.24630	-1.99350
C	-3.20330	3.43110	-2.84710
C	-3.97060	3.68650	-3.91350
C	-5.10060	2.80670	-4.28370
C	-5.39580	1.71290	-3.57270
H	-2.37580	4.09510	-2.63260
H	-3.75870	4.55310	-4.52660
H	-5.69660	3.05490	-5.15260
H	-6.22960	1.09050	-3.87520
C	-1.24750	5.16370	0.48960
C	-2.17900	4.25320	0.13610
C	-3.61970	4.61940	0.24140
C	-3.97990	5.84580	0.63670
C	-2.95200	6.84430	0.98460
C	-1.65630	6.52120	0.92020
H	-4.39300	3.89760	0.01540
H	-5.02790	6.10580	0.71220
H	-3.25010	7.83550	1.30100
H	-0.90720	7.25660	1.18650
P	-0.64470	-0.07700	0.22080
O	-2.11900	0.32180	0.82150
O	-0.02090	1.26530	-0.54570
O	0.19470	-0.44770	1.41640
O	-0.78340	-1.08890	-0.90110
H	0.27870	-2.24270	-0.99460
C	2.14060	-2.50400	-1.78880
N	1.09750	-2.92450	-1.03260
C	5.09950	-5.13240	-3.47580
C	3.84530	-5.62110	-3.10960
C	2.91170	-4.78360	-2.50520
C	3.21120	-3.43200	-2.26110
C	4.46660	-2.94720	-2.66430
C	5.40580	-3.78990	-3.25110
H	5.83000	-5.79040	-3.93660
H	3.58890	-6.66010	-3.29250
H	1.94010	-5.17820	-2.23920
H	4.72580	-1.90830	-2.50230
H	6.37700	-3.39490	-3.53220
C	1.08640	-3.92350	-0.03330
C	-0.23400	-3.98870	0.69540
H	-1.05810	-4.13390	-0.00720
H	-0.20550	-4.80790	1.41280
H	-0.41380	-3.03690	1.20420
O	2.03610	-4.64410	0.21070
C	1.77450	-1.41180	-2.77280
H	1.25510	-1.89410	-3.61130
H	2.67010	-0.94330	-3.17500
H	1.10110	-0.67180	-2.34060
C	3.50150	-1.70430	0.77970
H	1.64600	-1.31050	1.54360
C	7.44200	-3.38370	0.95110
C	6.33250	-4.21320	1.13640
C	5.04510	-3.68700	1.11680
C	4.85160	-2.31460	0.89340
C	5.96900	-1.49070	0.68360
C	7.25840	-2.01990	0.72750
H	8.44450	-3.80080	0.98040
H	6.47130	-5.27740	1.30080
H	4.18640	-4.33260	1.24800
H	5.82720	-0.42770	0.51680
H	8.11410	-1.36610	0.58740
C	3.18280	-0.98700	-0.36470
N	2.52710	-1.83800	1.72930
C	2.62720	-2.39070	3.02340
C	1.32550	-2.33540	3.79860
H	0.64260	-1.56650	3.43330
H	1.55990	-2.18120	4.85310
H	0.83150	-3.30950	3.70820
O	3.64720	-2.86960	3.47630

H	3.99090	-0.71350	-1.02660
H	2.31170	-0.34280	-0.35990
Si	-4.50090	-1.63540	0.45490
Si	2.40120	3.16310	0.14170
C	4.81320	7.16940	0.41670
C	4.81630	6.32770	1.45710
C	4.06180	5.05860	1.38690
C	3.37530	4.74230	0.27560
C	3.37120	5.68190	-0.87150
C	4.05360	6.83140	-0.80380
H	5.36430	8.09950	0.46790
H	5.36960	6.57510	2.35380
H	4.06630	4.40670	2.24910
H	2.81330	5.44320	-1.76850
H	4.04900	7.51620	-1.64190
C	3.34920	0.65180	3.99300
C	4.31120	0.87270	3.09160
C	4.01260	1.63370	1.86120
C	2.76760	2.08040	1.62000
C	1.70550	1.82630	2.62650
C	1.98300	1.15170	3.74860
H	3.56700	0.09830	4.89710
H	5.31370	0.50410	3.26600
H	4.82810	1.86080	1.19500
H	0.70010	2.19290	2.47320
H	1.20530	0.96910	4.47900
C	3.69990	1.49030	-4.05230
C	4.58450	1.54830	-3.05180
C	4.17390	2.06160	-1.72870
C	2.89960	2.42660	-1.50240
C	1.93100	2.38190	-2.62880
C	2.31080	1.93600	-3.83240
H	4.00000	1.11890	-5.02350
H	5.60580	1.22750	-3.21070
H	4.92950	2.16050	-0.96670
H	0.92080	2.74330	-2.50520
H	1.60310	1.91210	-4.65080
C	-2.99940	-5.31920	-2.03840
C	-3.66270	-5.51340	-0.89290
C	-4.14390	-4.35610	-0.11040
C	-3.91690	-3.10270	-0.54080
C	-3.19310	-2.89670	-1.82050
C	-2.75440	-3.94710	-2.52490
H	-2.64310	-6.16560	-2.61100
H	-3.84480	-6.51810	-0.53420
H	-4.67660	-4.53550	0.81530
H	-3.01050	-1.89990	-2.19690
H	-2.22100	-3.79330	-3.45400
C	-9.18210	-1.64320	0.82020
C	-8.47050	-0.62040	1.30550
C	-6.99760	-0.62160	1.18930
C	-6.35990	-1.65590	0.61400
C	-7.16030	-2.77570	0.05560
C	-8.49530	-2.76960	0.15890
H	-10.26130	-1.64310	0.90270
H	-8.97170	0.21220	1.78190
H	-6.45310	0.23530	1.55640
H	-6.68290	-3.59790	-0.45560
H	-9.07560	-3.58560	-0.25210
C	-2.52280	-1.52070	4.71830
C	-2.33530	-2.58310	3.92890
C	-2.96460	-2.63500	2.59350
C	-3.73980	-1.62370	2.16440
C	-3.94380	-0.44900	3.05020
C	-3.36420	-0.40030	4.25600
H	-2.05580	-1.47830	5.69370
H	-1.71690	-3.40580	4.26350
H	-2.77250	-3.49770	1.97750
H	-4.52420	0.39760	2.71380
H	-3.50100	0.46620	4.89000

$SiPh_3$ derived CPA *Type I E* TS

(B3LYP/6-31G**:UFF) Energy = -1679.374764

(B3LYP/6-31G**:UFF) Free Energy = -1678.233894

M06-2X/6-31G** Derived free energy = -4412.703274

M06-2X/6-31G** Derived free energy in solution = -4412.720214

Number of Imaginary Frequencies = 1 (-288.56)

ONIOM (B3LYP/6-31G**:UFF) Geometry

C	2.02050	2.39910	0.88330
C	4.54490	1.28400	1.48300
C	3.78300	0.78230	0.31690
C	2.55970	1.28930	0.05940
H	5.52440	0.87180	1.69680
C	-1.56560	4.43470	-0.37190
C	-1.60390	3.11070	-0.16190
C	-0.35500	2.37900	0.21050
C	0.79550	3.07840	0.39290
H	-2.45240	4.97620	-0.67100
C	4.00930	2.19920	2.30150
C	2.67740	2.78290	2.00130
C	2.13790	3.78560	2.95300
C	2.85050	4.15890	4.02260
C	4.18870	3.59200	4.29830
C	4.73600	2.67210	3.49610
H	1.15570	4.21660	2.80690
H	2.43950	4.88910	4.70780
H	4.73180	3.92840	5.17210
H	5.71680	2.27630	3.73150
C	-0.29780	5.17670	-0.28290
C	0.84260	4.53300	0.04470
C	2.12750	5.28850	0.01320
C	2.14070	6.59290	-0.28390
C	0.88260	7.29410	-0.59850
C	-0.27340	6.62300	-0.60510
H	3.06830	4.79340	0.21170
H	3.07720	7.13510	-0.30630
H	0.89980	8.34990	-0.83580
H	-1.19190	7.14160	-0.85030
P	0.50280	-0.08050	-0.22280
O	1.74920	0.74460	-0.90110
O	-0.44880	1.05010	0.56550
O	-0.28630	-0.72230	-1.33070
O	1.03780	-0.94700	0.90140
H	0.32560	-1.79450	2.02560
C	-1.09280	-3.26270	2.24530
N	-0.28180	-2.26990	2.74740
C	0.69160	-5.87140	-0.69360
C	1.47040	-4.92190	-0.03490
C	0.89570	-4.04670	0.88470
C	-0.47770	-4.11520	1.17700
C	-1.25210	-5.07340	0.49630
C	-0.67850	-5.93960	-0.42810
H	1.14500	-6.54940	-1.41060
H	2.53420	-4.86080	-0.23440
H	1.51800	-3.31040	1.37050
H	-2.32290	-5.12970	0.65890
H	-1.30630	-6.65520	-0.94890
C	-0.53330	-1.45290	3.86130
C	0.38610	-0.25020	3.92600
H	0.14210	0.43370	3.10580
H	0.24600	0.24510	4.88620
H	1.43210	-0.53840	3.79000
O	-1.40820	-1.66540	4.68560
C	-2.00060	-3.99450	3.21110
H	-2.63420	-4.70790	2.68670
H	-1.35840	-4.56370	3.89490
H	-2.58800	-3.31360	3.82000
C	-3.10190	-2.49070	0.07410
H	-1.55920	-1.84990	-1.11120
C	-7.24840	-3.52440	0.39680
C	-6.72300	-3.10730	-0.82760
C	-5.36650	-2.83070	-0.95280

C	-4.50420	-2.94360	0.15340
C	-5.05250	-3.34990	1.38540
C	-6.40570	-3.65000	1.50230
H	-8.30740	-3.74550	0.48980
H	-7.37130	-2.99940	-1.69140
H	-4.97590	-2.51440	-1.90840
H	-4.41880	-3.43030	2.25880
H	-6.80320	-3.97030	2.46030
C	-2.44180	-1.96580	1.19530
N	-2.38860	-2.48980	-1.09110
C	-2.45090	-3.41010	-2.15950
C	-1.34310	-3.21810	-3.17090
H	-0.83000	-2.26130	-3.06760
H	-0.61560	-4.02190	-3.01530
H	-1.75800	-3.33810	-4.17390
O	-3.27670	-4.30020	-2.23370
H	-3.03540	-1.69070	2.05430
H	-1.61830	-1.30420	0.97510
Si	4.54580	-0.60540	-0.67130
Si	-3.20180	2.18970	-0.46970
C	-4.68440	0.07040	3.44700
C	-5.39730	-0.02840	2.31980
C	-4.91310	0.60970	1.07810
C	-3.73960	1.26680	1.06100
C	-2.96800	1.39270	2.32480
C	-3.41490	0.82300	3.45080
H	-5.03780	-0.39610	4.35720
H	-6.33340	-0.57140	2.31510
H	-5.54700	0.57310	0.20520
H	-2.05810	1.97330	2.36240
H	-2.85280	0.92400	4.37010
C	-6.64690	5.26730	-1.24550
C	-6.21300	4.49800	-2.25100
C	-5.12690	3.52190	-2.02280
C	-4.57280	3.40160	-0.80420
C	-5.05640	4.25850	0.30510
C	-6.03950	5.14210	0.09480
H	-7.44160	5.98290	-1.41200
H	-6.65540	4.58970	-3.23450
H	-4.78750	2.92230	-2.85590
H	-4.61390	4.17440	1.29000
H	-6.39070	5.76730	0.90540
C	-2.62820	-0.31020	-4.40290
C	-3.71480	-0.50090	-3.64770
C	-3.88510	0.25170	-2.38680
C	-2.94570	1.12350	-1.98310
C	-1.74980	1.34090	-2.83950
C	-1.59690	0.65420	-3.97790
H	-2.50290	-0.86140	-5.32570
H	-4.47570	-1.20260	-3.96320
H	-4.79320	0.10780	-1.82480
H	-0.99000	2.05630	-2.55780
H	-0.71980	0.80980	-4.59300
C	8.82720	0.95240	-1.79480
C	8.69090	-0.25530	-1.23630
C	7.34910	-0.75850	-0.87480
C	6.25550	-0.01700	-1.12510
C	6.41940	1.33410	-1.71680
C	7.63640	1.78960	-2.03800
H	9.80950	1.32250	-2.05840
H	9.56340	-0.86710	-1.04690
H	7.27900	-1.72650	-0.40460
H	5.56120	1.97570	-1.86250
H	7.75200	2.77880	-2.46180
C	2.02360	-1.43720	-4.54550
C	2.14720	-2.38820	-3.61400
C	2.94390	-2.13480	-2.39680
C	3.57760	-0.96070	-2.22940
C	3.44380	0.08560	-3.27420
C	2.70070	-0.13850	-4.36520
H	1.43030	-1.61810	-5.43250
H	1.66010	-3.34480	-3.74650
H	2.98160	-2.90490	-1.64290
H	3.91690	1.04780	-3.15090
H	2.59220	0.63280	-5.11660
C	4.75790	-4.49290	1.95520
C	4.20940	-3.38170	2.45880
C	4.13690	-2.15360	1.64020
C	4.62760	-2.14250	0.38800
C	5.22290	-3.38380	-0.16700

C	5.28960	-4.49380	0.57800	H	3.67220	-1.27850	2.07110
H	4.80690	-5.39410	2.55240	H	5.60200	-3.39810	-1.18120
H	3.81080	-3.38060	3.46500	H	5.72310	-5.39720	0.16920

$SiPh_3$ derived CPA *Type II E* TS

(B3LYP/6-31G**:UFF) Energy = -1679.367827

(B3LYP/6-31G**:UFF) Free Energy = -1678.231175

M06-2X/6-31G** Derived free energy = -4412.702132

M06-2X/6-31G** Derived free energy in solution = -4412.719161

Number of Imaginary Frequencies = 1 (-223.49)

ONIOM (B3LYP/6-31G**:UFF) Geometry

C	-1.77560	-2.78110	-0.32880	C	-0.08800	-5.31850	-4.60730
C	-4.35500	-2.54150	0.79420	C	1.00040	-4.89550	-3.95450
C	-3.66440	-1.33610	0.28220	H	-2.56080	-3.93190	-2.68550
C	-2.39100	-1.43050	-0.16880	H	-2.30860	-5.28590	-4.66080
H	-5.34760	-2.45630	1.21800	H	0.01420	-5.92670	-5.49680
C	2.06230	-3.60480	-2.01420	H	1.98360	-5.16510	-4.31960
C	1.95490	-2.79930	-0.94360	P	-0.23400	0.01910	0.02540
C	0.60760	-2.31020	-0.53440	O	-1.72790	-0.31580	-0.63010
C	-0.48750	-2.90130	-1.04910	O	0.47620	-1.39970	0.48530
H	3.02900	-3.94990	-2.35590	O	0.56010	0.65650	-1.09280
C	-3.75380	-3.73390	0.76620	O	-0.42840	0.79540	1.30940
C	-2.39910	-3.87110	0.18200	H	0.10350	2.34980	1.43230
C	-1.78520	-5.22590	0.20180	C	1.66310	3.67090	1.81700
C	-2.45670	-6.27240	0.69640	N	0.36910	3.36730	1.50530
C	-3.82080	-6.12690	1.24610	C	3.85310	0.83290	4.22470
C	-4.43190	-4.93860	1.28590	C	2.49260	0.64740	3.99090
H	-0.77830	-5.38790	-0.15930	C	1.77760	1.54490	3.19900
H	-1.98970	-7.24900	0.70540	C	2.41900	2.65400	2.61540
H	-4.32910	-7.00190	1.63050	C	3.79850	2.82080	2.85510
H	-5.43030	-4.86810	1.70120	C	4.50540	1.92470	3.64740
C	0.86060	-4.06170	-2.73820	H	4.40490	0.13500	4.84660
C	-0.36590	-3.74280	-2.27370	H	1.97240	-0.20080	4.42420
C	-1.56770	-4.20380	-3.01910	H	0.72630	1.35830	3.03020
C	-1.43220	-4.95220	-4.12020	H	4.33850	3.64520	2.40390

H	5.56840	2.07500	3.80820
C	-0.59540	4.26260	1.00920
C	-1.88810	3.61820	0.57190
H	-2.05150	3.88260	-0.47600
H	-1.89340	2.53570	0.68750
H	-2.70230	4.07160	1.14230
O	-0.42240	5.46840	0.93520
C	2.05560	5.11500	2.03100
H	1.79290	5.75530	1.19630
H	1.50510	5.48340	2.90550
H	3.11850	5.19660	2.24820
C	2.02470	3.90120	-1.16930
H	0.80330	2.26490	-1.38540
C	3.35120	7.84770	-2.20310
C	4.24330	6.77630	-2.19510
C	3.79230	5.49810	-1.86790
C	2.43830	5.27060	-1.57270
C	1.54560	6.35580	-1.59080
C	2.00550	7.63360	-1.89260
H	3.70080	8.84600	-2.44940
H	5.28920	6.93140	-2.44230
H	4.48520	4.66360	-1.87310
H	0.50440	6.20030	-1.33840
H	1.30850	8.46580	-1.89000
C	2.70850	3.25440	-0.14670
N	0.95710	3.24970	-1.71920
C	0.26650	3.55430	-2.91600
C	-0.64150	2.42970	-3.36290
H	-1.34260	2.14890	-2.57240
H	-1.17470	2.75130	-4.25650
H	-0.05680	1.52860	-3.56870
O	0.39930	4.59450	-3.52840
H	3.65120	3.67870	0.16990
H	2.56360	2.18700	-0.02900
Si	-4.60920	0.27400	0.33810
Si	3.49160	-2.40370	0.04880
C	6.85850	-5.44780	-1.11350
C	5.72010	-5.88010	-0.55890
C	4.66170	-4.91600	-0.19400
C	4.83350	-3.60300	-0.42300

C	6.10100	-3.12870	-1.03180
C	7.05850	-4.00530	-1.35930
H	7.63500	-6.15260	-1.38110
H	5.57150	-6.93650	-0.37640
H	3.74740	-5.28510	0.25450
H	6.26670	-2.07580	-1.21350
H	7.98300	-3.65910	-1.80310
C	5.33150	1.67430	-1.28290
C	5.34830	1.33980	0.01240
C	4.67230	0.10790	0.46920
C	4.06230	-0.69650	-0.41680
C	4.01540	-0.29990	-1.84550
C	4.61410	0.82530	-2.25390
H	5.83400	2.57140	-1.61900
H	5.86690	1.96530	0.72720
H	4.73280	-0.15040	1.51320
H	3.51180	-0.92750	-2.56960
H	4.58450	1.10900	-3.29780
C	3.08760	-3.15930	4.66640
C	2.03240	-3.36500	3.87100
C	2.14490	-3.13560	2.41560
C	3.29060	-2.67030	1.88670
C	4.46980	-2.49310	2.77110
C	4.37080	-2.71870	4.08660
H	3.00750	-3.32960	5.73210
H	1.09450	-3.70540	4.29040
H	1.29680	-3.38190	1.79720
H	5.42630	-2.20430	2.35450
H	5.23380	-2.58880	4.72660
C	-3.36140	2.71170	4.14980
C	-2.73130	1.57010	3.85220
C	-3.13790	0.78690	2.66730
C	-4.11430	1.23460	1.85900
C	-4.83370	2.48240	2.21800
C	-4.47450	3.18160	3.30170
H	-3.06390	3.28730	5.01670
H	-1.91880	1.21920	4.47530
H	-2.62190	-0.14050	2.46270
H	-5.66970	2.82260	1.62130
H	-5.00330	4.08920	3.56240

C	-9.16320	-0.82740	0.46120
C	-8.58820	-0.28100	1.53870
C	-7.15430	0.07610	1.51350
C	-6.42440	-0.13300	0.40400
C	-7.06550	-0.75120	-0.78190
C	-8.36340	-1.07760	-0.75430
H	-10.21270	-1.09150	0.47950
H	-9.17100	-0.10270	2.43320
H	-6.71020	0.48580	2.40930
H	-6.48700	-0.94910	-1.67460
H	-8.82850	-1.52980	-1.62080
C	-4.09730	2.55800	-3.72600
C	-4.32420	3.24870	-2.60340
C	-4.46820	2.54150	-1.31390
C	-4.34430	1.20550	-1.25450
C	-4.09310	0.44630	-2.50560
C	-3.97640	1.08780	-3.67440
H	-3.99470	3.07450	-4.67150
H	-4.40940	4.32710	-2.63740
H	-4.68060	3.12460	-0.43280
H	-4.01260	-0.63290	-2.48420
H	-3.79360	0.53080	-4.58420

<u>Data for delocalisation bonding model</u>

TS1

M06-2X/6-31G** Derived free energy = -3194.583783

M06-2X/6-31G** Derived free energy in solution = -3194.601744

ONIOM (B3LYP/6-31G**:UFF) Geometry

C	3.90140	0.10720	-0.21070
C	4.89380	2.71850	-0.54070
C	3.52910	2.52060	-0.30360
C	3.01490	1.21260	-0.19670
H	5.27710	3.72920	-0.63260
C	2.50180	-3.90340	0.44990
C	1.96140	-3.12640	-0.58630
C	2.36760	-1.78130	-0.71540
C	3.37370	-1.25120	0.11200
H	2.20140	-4.93960	0.55690
C	5.75870	1.63080	-0.68910
C	5.26800	0.31000	-0.53910
C	6.15950	-0.76840	-0.73430
C	7.50400	-0.53550	-1.03190
C	7.98160	0.76900	-1.14960
C	7.11460	1.84910	-0.98270
H	5.82620	-1.79550	-0.67180
H	8.17750	-1.37070	-1.17480
H	9.02480	0.94350	-1.37940
H	7.50250	2.85540	-1.08920
C	3.41910	-3.35630	1.35580
C	3.85740	-2.01700	1.20150
C	4.75240	-1.48100	2.15210
C	5.22340	-2.26730	3.20580
C	4.80670	-3.59190	3.33490
C	3.90710	-4.13590	2.41720
H	5.08390	-0.45270	2.09650
H	5.91200	-1.84640	3.92710
H	5.17560	-4.19660	4.15330
H	3.58680	-5.16370	2.54130
O	1.68270	1.03690	0.09790
O	1.78610	-0.96970	-1.65340
P	0.78130	0.15680	-0.99090
O	0.34050	1.00290	-2.16200
O	-0.26340	-0.52010	-0.12860
H	-1.51640	-0.55590	0.90210
C	-0.96270	-4.82570	-3.24660

C	0.95870	-3.72350	-1.51120	H	-1.74990	1.90520	0.72230
H	-1.70730	-5.24170	-3.91270	C	-1.94450	-3.00050	1.44240
C	1.04200	5.97550	0.16890	H	-2.19990	-3.23100	0.40220
C	2.65920	3.70330	-0.13790	H	-2.10720	-3.89730	2.04100
H	0.41600	6.85020	0.28840	H	-0.88750	-2.72360	1.46590
C	-3.95630	2.14710	-1.02010	N	-4.82020	-4.35040	3.28280
C	-2.85200	2.14300	-1.83900	C	-4.41370	-3.29640	2.99130
C	-2.86000	-0.28620	-1.94270	C	-4.81360	-0.79940	2.97460
C	-3.97240	-0.34670	-1.12470	H	-5.61450	-0.72900	2.22980
N	-2.30690	0.93600	-2.20410	H	-5.29250	-0.93930	3.94820
H	-1.28630	0.94570	-2.45770	C	-3.97620	0.48600	2.99450
C	-4.39900	0.87540	-0.42140	H	-3.40110	0.54200	3.93150
H	-3.87680	0.82130	0.70350	H	-4.61520	1.37210	2.95850
H	-5.44320	0.86350	-0.10970	C	-2.10190	1.80120	1.75360
C	-2.12370	3.32640	-2.41170	H	-2.72330	2.67450	1.97640
H	-2.51760	3.56560	-3.40600	C	-0.86670	1.74980	2.68050
H	-2.24460	4.21010	-1.79040	C	-0.03940	3.03800	2.63010
H	-1.06450	3.07770	-2.51480	H	-0.65020	3.88550	2.96980
C	-2.16360	-1.43410	-2.62040	H	0.23270	3.24050	1.58840
H	-2.85540	-1.98300	-3.26200	C	1.23270	2.93640	3.47670
H	-1.32580	-1.06780	-3.21500	H	1.90340	2.17220	3.07020
H	-1.77700	-2.12920	-1.87540	H	1.00170	2.66560	4.51340
C	-4.76310	-1.55260	-0.81940	H	1.77670	3.88560	3.49970
C	-4.64920	3.34360	-0.50610	C	-0.06710	-4.54980	-1.01440
C	-5.06720	-3.87960	-1.08880	H	-0.13890	-4.76420	0.04360
H	-5.08280	-4.08240	-0.01580	C	-1.02620	-5.08860	-1.87700
H	-6.09370	-3.76150	-1.44240	H	-1.82150	-5.70730	-1.48180
H	-4.56660	-4.68580	-1.62470	C	1.02450	-3.48570	-2.89770
C	-5.17260	5.62610	-0.76670	H	1.82450	-2.89100	-3.31950
H	-4.96120	6.41240	-1.49080	C	0.06550	-4.03060	-3.75710
H	-6.24840	5.45470	-0.68730	H	0.12040	-3.83640	-4.82040
H	-4.78560	5.89600	0.21890	C	2.90270	4.61210	0.90500
O	-5.75820	-1.53350	-0.11070	H	3.71150	4.43900	1.60440
O	-5.30690	3.33060	0.52320	C	2.09040	5.73920	1.06170
O	-4.51170	4.45190	-1.26990	H	2.27390	6.42900	1.87510
O	-4.29540	-2.69660	-1.37500	C	1.61470	3.95590	-1.04050
C	-3.94810	-2.00030	2.63770	H	1.44330	3.29380	-1.87710
C	-2.80080	-1.86640	1.92090	C	0.80980	5.08910	-0.88590
N	-2.35870	-0.60400	1.52700	H	0.01390	5.28830	-1.59020
C	-2.96000	0.55920	1.87260	H	-1.18030	1.55590	3.71510

H	-0.23610	0.91130	2.36800

TS2

M06-2X/6-31G** Derived free energy = -3194.578725

M06-2X/6-31G** Derived free energy in solution = -3194.596078

ONIOM (B3LYP/6-31G**:UFF) Geometry

C	3.78000	0.77870	-0.16260
C	4.28720	3.47890	-0.78440
C	2.97120	3.04080	-0.61210
C	2.70900	1.68530	-0.34680
H	4.47630	4.52620	-0.99440
C	3.09250	-3.34180	0.86150
C	2.51610	-2.79330	-0.29430
C	2.66010	-1.41160	-0.53980
C	3.48070	-0.61620	0.28240
H	2.98630	-4.40150	1.06370
C	5.35280	2.57490	-0.71840
C	5.10850	1.20910	-0.42310
C	6.20130	0.31430	-0.41070
C	7.49930	0.77330	-0.64540
C	7.73110	2.12320	-0.90590
C	6.66450	3.02140	-0.94670
H	6.06340	-0.74350	-0.23250
H	8.32860	0.07800	-0.62810
H	8.73940	2.47300	-1.08600
H	6.86380	4.06430	-1.16330
C	3.79540	-2.53550	1.76570
C	3.99440	-1.15970	1.48570
C	4.68230	-0.36660	2.42860
C	5.18250	-0.93390	3.60260
C	5.00060	-2.29250	3.85980
C	4.30950	-3.09170	2.94820
H	4.82610	0.69440	2.27390
H	5.71000	-0.31610	4.31800
H	5.39040	-2.72630	4.77160
H	4.17040	-4.14310	3.17080
O	1.41700	1.29920	-0.09270
O	1.99680	-0.82580	-1.58600
P	0.76160	0.14770	-1.09670
O	0.21470	0.74860	-2.36930
O	-0.18700	-0.58720	-0.16660
H	-1.21830	0.31320	0.84820
C	0.36800	-5.34480	-3.01500
C	1.76570	-3.67070	-1.23570
H	-0.17370	-5.98470	-3.69930
C	-0.24180	5.85960	-0.82920
C	1.86840	4.01800	-0.69400
H	-1.05920	6.56710	-0.87880
C	-4.20480	0.73560	-0.99190
C	-3.14120	1.00770	-1.82940
C	-2.60630	-1.35080	-1.99680
C	-3.65250	-1.68350	-1.16140
N	-2.35160	-0.02960	-2.24270
H	-1.38740	0.22350	-2.56220
C	-4.33120	-0.61330	-0.40730
H	-3.78820	-0.56170	0.72050
H	-5.34350	-0.84970	-0.08350
C	-2.73100	2.34000	-2.39850
H	-3.33980	2.57860	-3.27730
H	-2.88130	3.14440	-1.68120
H	-1.68150	2.29710	-2.69810
C	-1.69830	-2.31030	-2.70820
H	-1.35030	-3.08010	-2.02220
H	-2.23860	-2.82640	-3.50700
H	-0.84350	-1.78060	-3.13190
C	-4.01800	-3.09180	-0.94030
C	-5.22900	1.70580	-0.55960
C	-5.39270	-4.55570	0.30150

H	-4.56110	-5.19220	0.61380
H	-5.84030	-4.98370	-0.59870
H	-6.13400	-4.47570	1.09640
C	-6.10900	3.89180	-0.69610
H	-5.96590	4.75970	-1.33890
H	-7.12730	3.50830	-0.78300
H	-5.90920	4.15410	0.34570
O	-3.57990	-4.05990	-1.53770
O	-6.08620	1.43730	0.27080
O	-5.15770	2.91330	-1.15850
O	-4.94330	-3.21450	0.05500
C	-3.29720	2.33430	2.49690
C	-2.21480	1.94670	1.76870
N	-2.01260	0.59670	1.47440
C	-2.82100	-0.40980	1.89310
H	-2.99910	-2.53600	1.95450
C	-1.16590	2.86810	1.22250
H	-1.34910	3.89670	1.53260
H	-1.14960	2.82520	0.12910
H	-0.16900	2.55900	1.55010
N	-3.80000	4.83430	2.94080
C	-3.54310	3.71440	2.73770
C	-4.33400	1.33980	2.99380
H	-4.66760	1.62460	3.99670
H	-5.21280	1.37920	2.34320
C	-3.75930	-0.08120	3.03570
H	-4.55680	-0.82750	3.08180
H	-3.15850	-0.20800	3.94870
C	-2.20540	-1.79090	1.85540
H	-1.71390	-1.93070	0.88840
C	-1.14280	-1.95880	2.97310
C	-0.20720	-3.15230	2.74340
H	0.55390	-3.14760	3.53400
H	0.32420	-2.98000	1.80160
C	-0.90380	-4.51630	2.72040
H	-1.59090	-4.60860	1.87260
H	-1.47930	-4.68350	3.63860
H	-0.17470	-5.32840	2.63660
C	0.88680	-4.65880	-0.75190
H	0.73240	-4.78960	0.30880
C	0.19080	-5.48680	-1.63740
H	-0.48930	-6.23670	-1.25460
C	1.94820	-3.55100	-2.62710
H	2.64660	-2.83050	-3.03300
C	1.24790	-4.37980	-3.50900
H	1.39250	-4.27720	-4.57660
C	1.72980	5.00760	0.29170
H	2.43040	5.06110	1.11600
C	0.67420	5.92250	0.22490
H	0.56460	6.67830	0.99170
C	0.95180	3.96670	-1.75570
H	1.05950	3.22540	-2.53630
C	-0.09990	4.88570	-1.82150
H	-0.80460	4.84350	-2.64170
H	-0.53400	-1.04910	3.01280
H	-1.63960	-2.05660	3.94740

TS3

M06-2X/6-31G** Derived free energy = -3808.695170

M06-2X/6-31G** Derived free energy in solution = -3808.713161

ONIOM (B3LYP/6-31G**:UFF) Geometry

C	3.89580	-0.63900	0.00550
C	4.16590	-3.31110	0.84030
C	2.97760	-2.88570	0.23710
C	2.82470	-1.54050	-0.13990
H	4.27050	-4.34970	1.13470
C	3.49620	3.39530	-1.41630
C	2.59960	2.88500	-0.47090
C	2.68220	1.53470	-0.08170

C	3.73800	0.72880	-0.56080
H	3.41860	4.43520	-1.71480
C	5.20140	-2.40300	1.09010
C	5.07090	-1.04970	0.68550
C	6.11260	-0.14730	0.98950
C	7.26460	-0.58760	1.64480
C	7.39450	-1.92580	2.01560
C	6.36790	-2.83100	1.74370
H	6.04160	0.90180	0.73520
H	8.05870	0.11290	1.86900
H	8.28990	-2.26090	2.52310
H	6.48260	-3.86410	2.05020
C	4.46990	2.57380	-1.99270
C	4.59420	1.22430	-1.57790
C	5.55840	0.40780	-2.20820
C	6.39660	0.93100	-3.19510
C	6.28400	2.26740	-3.57680
C	5.32400	3.08690	-2.98200
H	5.66420	-0.63860	-1.95520
H	7.13430	0.29590	-3.66850
H	6.93650	2.66730	-4.34220
H	5.24380	4.11950	-3.30060
O	1.65640	-1.11810	-0.72470
O	1.79710	1.03050	0.84260
P	0.75640	-0.11120	0.24220
O	0.25210	-0.88620	1.43980
O	-0.23430	0.53090	-0.70290
H	-1.78930	0.96160	-0.79240
C	-0.22720	5.68580	1.18170
C	1.59020	3.80080	0.12030
H	-0.92890	6.40450	1.59220
C	-0.17860	-5.73860	-0.43960
C	1.88930	-3.86730	0.00440
H	-0.97620	-6.45400	-0.61100
C	-3.79070	-1.62900	-0.75340
C	-2.50470	-2.03670	-0.44980
C	-2.94720	-1.81380	1.92940
C	-4.23590	-1.40450	1.67820
N	-2.10830	-2.02480	0.86070
H	-1.08790	-1.90840	1.05530

C	-4.58350	-0.99920	0.30740
H	-4.30010	0.23780	0.23310
H	-5.65300	-0.93530	0.11990
C	-1.47710	-2.52710	-1.42440
H	-1.39940	-1.86340	-2.28300
H	-0.50310	-2.59430	-0.94410
H	-1.76800	-3.50840	-1.81050
C	-2.30180	-2.03990	3.26770
H	-1.22770	-1.85830	3.18430
H	-2.72460	-1.38710	4.02870
H	-2.46480	-3.07050	3.59920
C	-5.26850	-1.13040	2.69560
C	-4.31500	-1.73280	-2.12860
C	-6.07890	-1.51600	4.87430
H	-5.77860	-2.10390	5.74110
H	-6.10670	-0.45200	5.12080
H	-7.06740	-1.82250	4.52510
C	-6.18040	-1.34530	-3.52790
H	-6.14900	-2.37610	-3.88670
H	-7.21010	-1.01320	-3.39900
H	-5.66000	-0.70080	-4.23920
O	-6.21770	-0.39040	2.49030
O	-3.72080	-2.19330	-3.08920
O	-5.58080	-1.25030	-2.22090
O	-5.08540	-1.77980	3.86780
C	-4.37390	1.95190	-2.62430
C	-3.13040	1.49470	-2.30930
N	-2.76920	1.30190	-0.97600
C	-3.60190	1.52330	0.07600
H	-3.75010	1.42320	2.19360
C	-2.09380	1.08920	-3.31950
H	-2.19040	1.67790	-4.23310
H	-2.24280	0.03690	-3.58930
H	-1.08720	1.18790	-2.90930
N	-5.11430	2.23230	-5.08780
C	-4.75390	2.10400	-3.98530
C	-5.42880	2.20270	-1.56170
H	-6.04180	3.06790	-1.83240
H	-6.10550	1.34010	-1.52100
C	-4.78070	2.44180	-0.19420

H	-5.51290	2.34290	0.61130
H	-4.38630	3.46740	-0.15150
C	-2.96050	1.56940	1.44790
H	-2.25620	0.73990	1.54150
C	-0.41780	5.19490	-0.11280
C	0.48650	4.24630	-0.65620
C	0.24960	3.76210	-1.96240
C	-0.84330	4.21680	-2.70330
C	-1.73230	5.13730	-2.15330
C	-1.52300	5.62620	-0.86470
H	0.89790	3.02650	-2.41960
H	-1.00560	3.84770	-3.70720
H	-2.58560	5.47370	-2.72780
H	-2.22700	6.34030	-0.45400
C	0.84650	5.24490	1.96050
C	1.76920	4.30330	1.43720
C	2.85550	3.90240	2.24900
C	2.99900	4.40070	3.54530
C	2.07670	5.31320	4.05330
C	1.00810	5.73910	3.26530
H	3.60100	3.20320	1.89570
H	3.83140	4.07890	4.15780
H	2.19330	5.69610	5.05890
H	0.30530	6.45380	3.67710
C	0.03950	-5.24090	0.84830
C	1.07390	-4.29980	1.08390
C	1.25570	-3.81220	2.39800
C	0.44880	-4.26440	3.44360
C	-0.55940	-5.19530	3.20080
C	-0.77030	-5.67710	1.90950
H	2.01580	-3.07830	2.63000
H	0.60000	-3.88350	4.44540
H	-1.18600	-5.53710	4.01440
H	-1.56480	-6.39440	1.74080
C	1.66310	-4.38540	-1.29870
C	0.61830	-5.32120	-1.50980
C	0.37520	-5.82590	-2.79760
C	1.16050	-5.41660	-3.87500
C	2.19710	-4.50610	-3.67820
C	2.45250	-3.99460	-2.40430
H	-0.42430	-6.53660	-2.97140
H	0.96680	-5.80890	-4.86500
H	2.80700	-4.19500	-4.51660
H	3.27060	-3.29480	-2.29750
C	-2.20460	2.88040	1.74350
H	-1.35040	2.92900	1.06250
H	-2.84880	3.74110	1.52880
C	-1.70790	2.97120	3.19650
H	-2.54810	2.77220	3.87690
H	-1.41180	4.00860	3.39130
C	-0.53260	2.04390	3.52430
H	0.31920	2.23240	2.86500
H	-0.78460	0.98660	3.40480
H	-0.19950	2.19280	4.55680

TS4

M06-2X/6-31G** Derived free energy = -3808.689752

M06-2X/6-31G** Derived free energy in solution = -3808.707488

ONIOM (B3LYP/6-31G**:UFF) Geometry

C	3.87580	-0.31910	-0.28530
C	4.75980	2.01040	-1.59450
C	3.56870	2.03160	-0.86290
C	3.10960	0.86180	-0.23280
H	5.10050	2.91780	-2.08140
C	2.56850	-3.97020	1.54840

C	1.75050	-3.30860	0.62440
C	2.12580	-2.04030	0.13880
C	3.39060	-1.50850	0.47020
H	2.26980	-4.94390	1.92130
C	5.49060	0.82510	-1.72940
C	5.04450	-0.36060	-1.09080
C	5.76790	-1.55500	-1.30130
C	6.92380	-1.55750	-2.08500
C	7.37130	-0.37940	-2.68140
C	6.65740	0.80690	-2.51020
H	5.44310	-2.49510	-0.87690
H	7.47230	-2.47880	-2.23410
H	8.26780	-0.38790	-3.28790
H	7.01180	1.70850	-2.99580
C	3.75060	-3.37820	2.00660
C	4.17190	-2.13400	1.47410
C	5.35930	-1.55120	1.96600
C	6.11810	-2.20240	2.94120
C	5.70640	-3.43650	3.44400
C	4.52790	-4.02380	2.98130
H	5.70200	-0.58800	1.61140
H	7.02800	-1.74680	3.30990
H	6.29940	-3.93710	4.19850
H	4.22140	-4.98000	3.38880
O	1.94340	0.88310	0.49060
O	1.31950	-1.38750	-0.76450
P	0.70120	0.05620	-0.22820
O	0.25580	0.78240	-1.47740
O	-0.30120	-0.17410	0.88280
H	-1.86840	-0.54500	0.56010
C	-1.81470	-5.25150	-0.83950
C	0.52280	-3.98710	0.12670
H	-2.71650	-5.72500	-1.21390
C	1.36190	5.72460	-0.66010
C	2.79410	3.29440	-0.78470
H	0.80640	6.65530	-0.60900
C	-3.29980	2.93250	0.83210
C	-2.03100	2.93860	0.28640
C	-2.84980	1.85670	-1.72550
C	-4.11820	1.75980	-1.19190

N	-1.84090	2.34710	-0.93130
H	-0.88090	2.01150	-1.18480
C	-4.31220	2.05770	0.23340
H	-4.21720	0.93030	0.85440
H	-5.33860	2.28400	0.51600
C	-0.79430	3.53360	0.89660
H	-0.75320	3.34680	1.96860
H	0.09540	3.10750	0.43220
H	-0.79180	4.61860	0.76050
C	-2.39910	1.52420	-3.12200
H	-1.34000	1.25790	-3.10530
H	-2.98320	0.71610	-3.55390
H	-2.52920	2.40130	-3.76690
C	-5.32930	1.28240	-1.89780
C	-3.71030	3.62150	2.07000
C	-6.34040	0.59270	-3.91390
H	-6.50340	-0.44220	-3.60320
H	-7.23400	1.18120	-3.69850
H	-6.09290	0.63680	-4.97390
C	-3.22480	5.25130	3.70140
H	-4.17960	5.76490	3.56830
H	-3.31810	4.54380	4.52870
H	-2.42730	5.96760	3.89720
O	-6.37260	1.03610	-1.31240
O	-4.75120	3.37270	2.65690
O	-2.84390	4.57230	2.49260
O	-5.19550	1.14380	-3.23170
C	-4.60050	-2.07430	-0.56700
C	-3.31080	-1.64120	-0.51250
N	-2.88740	-0.80930	0.52110
C	-3.71460	-0.29670	1.47500
H	-2.33600	1.09660	2.31320
C	-2.25710	-1.94160	-1.53650
H	-2.57470	-2.73590	-2.21160
H	-2.03520	-1.04860	-2.12810
H	-1.31650	-2.21800	-1.05820
N	-5.45150	-3.49740	-2.55410
C	-5.04170	-2.86400	-1.66370
C	-5.64210	-1.64350	0.45270
H	-6.29300	-0.88990	-0.00250

H	-6.27840	-2.49420	0.71790	C	1.78000	2.86970	-3.00380
C	-4.98930	-1.08380	1.72330	C	0.90250	3.24720	-4.02210
H	-4.68830	-1.91340	2.38010	C	0.18240	4.43610	-3.92360
H	-5.69660	-0.47320	2.29010	C	0.34160	5.25980	-2.80950
C	-3.03510	0.33500	2.67170	H	2.31040	1.93330	-3.11670
H	-3.79770	0.84310	3.26990	H	0.77620	2.61120	-4.88880
C	-1.76590	-4.80660	0.48380	H	-0.49960	4.72130	-4.71400
C	-0.59650	-4.17630	0.98380	H	-0.22500	6.18190	-2.75380
C	-0.58890	-3.74850	2.33040	C	2.91340	4.13450	0.35310
C	-1.69570	-3.96530	3.15370	C	2.19140	5.35430	0.40300
C	-2.84460	-4.56580	2.64490	C	2.29840	6.18940	1.52710
C	-2.88400	-4.98170	1.31550	C	3.11540	5.82800	2.59830
H	0.26460	-3.24380	2.76160	C	3.83410	4.63440	2.55800
H	-1.66780	-3.65010	4.18840	C	3.73940	3.79280	1.44770
H	-3.70660	-4.71080	3.28310	H	1.74760	7.12120	1.57840
H	-3.78680	-5.44790	0.93870	H	3.19240	6.47590	3.46190
C	-0.71470	-5.09190	-1.68610	H	4.46830	4.35930	3.39090
C	0.46830	-4.46910	-1.21100	H	4.31590	2.87720	1.45770
C	1.56990	-4.35980	-2.09160	C	-2.23100	-0.65730	3.53230
C	1.48220	-4.82310	-3.40570	H	-1.47350	-1.12120	2.89590
C	0.30620	-5.41150	-3.86620	H	-2.89280	-1.44550	3.91380
C	-0.78690	-5.55010	-3.01180	C	-1.52720	0.03020	4.70750
H	2.50860	-3.92500	-1.77650	H	-0.87330	0.81930	4.31440
H	2.33210	-4.72760	-4.06920	H	-2.26840	0.52730	5.34800
H	0.24390	-5.76820	-4.88620	C	-0.69530	-0.94860	5.54170
H	-1.68930	-6.01850	-3.38690	H	-1.32320	-1.73400	5.97740
C	1.21730	4.89290	-1.77460	H	0.06790	-1.43640	4.92560
C	1.94260	3.67700	-1.85530	H	-0.18420	-0.43770	6.36400

TS5-*E*

M06-2X/6-31G** Derived free energy = -3461.068467

M06-2X/6-31G** Derived free energy in solution = -3461.092197

ONIOM (B3LYP/6-31G**:UFF) Geometry

C	2.06080	2.46540	0.47730	C	-1.46840	4.32920	-1.12100
C	4.45640	1.33880	1.42710	C	-1.51210	3.00520	-0.67130
C	3.73970	0.69130	0.41450	C	-0.33290	2.37890	-0.22490
C	2.52620	1.23940	-0.04020	C	0.87750	3.10350	-0.17100
H	5.40630	0.93160	1.75090	H	-2.38510	4.81680	-1.43000

C	3.94340	2.48680	2.04130
C	2.72520	3.05130	1.58500
C	2.20580	4.17840	2.25740
C	2.89990	4.74960	3.32620
C	4.11320	4.20580	3.74730
C	4.63370	3.07790	3.11150
H	1.25900	4.61760	1.97280
H	2.49360	5.61620	3.83160
H	4.64710	4.65380	4.57540
H	5.57050	2.66150	3.46270
C	0.93910	4.40300	-0.73790
C	-0.25380	5.01840	-1.19390
C	-0.21680	6.31930	-1.72080
C	0.99540	7.00250	-1.82390
C	2.17920	6.39350	-1.40890
C	2.15740	5.10350	-0.87420
H	-1.12410	6.80350	-2.06250
H	1.01840	8.00290	-2.23640
H	3.11900	6.92210	-1.50430
H	3.09940	4.65890	-0.58280
P	0.42550	-0.03210	-0.63860
O	1.85440	0.64040	-1.07510
O	-0.38710	1.10140	0.27450
O	-0.33060	-0.26130	-1.92980
O	0.62600	-1.18550	0.31350
H	-0.33830	-2.60310	0.65010
C	-1.94850	-3.42360	1.67680
N	-0.86190	-3.48280	0.81480
C	-1.46870	-0.86920	5.15380
C	-0.43900	-0.98970	4.22370
C	-0.59650	-1.77850	3.08400
C	-1.79080	-2.48210	2.85520
C	-2.82820	-2.33530	3.79550
C	-2.67180	-1.54000	4.92690
H	-1.34310	-0.25220	6.03840
H	0.49850	-0.46220	4.37130
H	0.20870	-1.80710	2.36500
H	-3.77560	-2.84480	3.65540
H	-3.49180	-1.44710	5.63260
C	-4.57770	-4.41410	-0.83290
C	-4.58430	-4.93130	-2.12830
C	-3.81200	-4.35320	-3.14880
C	-3.02530	-3.22720	-2.90860
H	-5.18310	-4.86960	-0.05520
H	-5.19830	-5.79770	-2.35370
H	-3.83600	-4.78410	-4.14480
H	-2.43740	-2.76130	-3.69240
C	-2.74350	-1.39080	0.15100
C	-3.49700	-2.50570	0.63450
C	-3.78490	-3.29470	-0.56530
C	-3.04030	-2.71450	-1.61480
H	-2.32440	-0.56690	0.70940
H	-4.18200	-2.41520	1.46630
N	-2.41030	-1.56460	-1.11630
H	-1.60680	-1.00010	-1.58000
C	-0.45770	-4.55060	-0.00250
O	-0.99430	-5.64510	-0.01750
C	0.76100	-4.24460	-0.85520
H	0.56650	-4.61080	-1.86580
H	1.02560	-3.18710	-0.87590
H	1.60690	-4.81810	-0.46100
C	-5.36020	1.05770	-0.55590
C	-4.71370	1.27270	-1.77650
C	-3.44280	1.87210	-1.84140
C	-2.81760	2.29860	-0.63200
C	-3.48700	2.11790	0.61180
C	-4.73330	1.46600	0.62740
H	-5.21640	0.98260	-2.68930
H	-5.24300	1.31210	1.56850
C	5.30130	-2.92420	-1.35040
C	5.28250	-1.73080	-2.07700
C	4.80210	-0.53490	-1.51410
C	4.28240	-0.55410	-0.18740
C	4.31320	-1.76540	0.56600
C	4.84400	-2.92350	-0.02850
H	5.66500	-1.72990	-3.08940
H	4.89470	-3.84150	0.53980
C	-2.81510	2.11100	-3.21780
H	-1.75800	2.43090	-3.11390
C	-2.93750	2.67710	1.92720

H	-1.97680	3.20620	1.77060	C	4.90530	0.75140	-2.33770
C	-6.74620	0.43540	-0.53270	H	4.50010	1.61960	-1.78230
H	-7.07970	0.22860	-1.57430	C	6.37030	1.09860	-2.63180
C	-2.65030	1.56070	2.93740	H	6.94170	1.16580	-1.68130
H	-1.90070	0.85990	2.52020	H	6.84500	0.33500	-3.28320
H	-2.22860	1.98980	3.87120	H	6.43230	2.08300	-3.14340
H	-3.57000	0.99520	3.19500	C	4.09420	0.65290	-3.63730
C	-3.89150	3.72120	2.52240	H	4.53510	-0.08970	-4.33480
H	-4.84990	3.26010	2.84130	H	3.04740	0.35620	-3.41730
H	-3.42410	4.20220	3.40820	H	4.07400	1.64020	-4.14640
H	-4.10440	4.51210	1.77150	C	7.06000	-4.72420	-1.26620
C	-7.77480	1.39530	0.07830	H	7.83930	-3.93350	-1.22300
H	-7.57280	1.56970	1.15630	H	6.81430	-5.04330	-0.23120
H	-7.75000	2.36970	-0.45500	H	7.47990	-5.59570	-1.81240
H	-8.79670	0.97130	-0.02270	C	4.71970	-5.26650	-2.05930
C	-6.74060	-0.90750	0.20440	H	4.41850	-5.59520	-1.04210
H	-6.41350	-0.78880	1.25910	H	3.82680	-4.86390	-2.58420
H	-7.75840	-1.35230	0.19670	H	5.08370	-6.15230	-2.62240
H	-6.05790	-1.61230	-0.31160	C	2.83760	-3.01770	2.22190
C	-3.55790	3.23010	-3.95890	H	2.07090	-3.02590	1.42500
H	-3.57210	4.15720	-3.35040	H	3.35820	-3.99790	2.21690
H	-3.04750	3.45560	-4.91950	H	2.32680	-2.91020	3.20260
H	-4.60700	2.93530	-4.17500	C	4.99770	-1.97460	2.98110
C	-2.78470	0.83960	-4.08220	H	5.55280	-2.92210	2.81200
H	-2.15170	1.01000	-4.97920	H	5.70070	-1.12730	2.84730
H	-2.35720	-0.00970	-3.51950	H	4.63270	-1.95880	4.03030
H	-3.79950	0.55530	-4.43050	C	-2.62220	-4.74060	2.01880
C	5.81820	-4.19770	-1.99570	H	-3.49210	-4.57560	2.65370
H	6.12460	-3.98620	-3.04460	H	-2.89940	-5.30140	1.13250
C	3.81580	-1.85110	2.01150	H	-1.90520	-5.35140	2.57870
H	3.25570	-0.93470	2.28830				

TS6-*E*

M06-2X/6-31G** Derived free energy = -3461.066456

M06-2X/6-31G** Derived free energy in solution = -3461.089568

ONIOM (B3LYP/6-31G**:UFF) Geometry

C	2.27300	2.27470	0.58670	C	4.54900	0.95590	1.57850

C	3.79540	0.36890	0.55620
C	2.62220	0.99770	0.10430
H	5.45940	0.47260	1.91370
C	-1.03360	4.42940	-1.11440
C	-1.22000	3.14500	-0.59170
C	-0.11500	2.41560	-0.11800
C	1.16580	3.00580	-0.09380
H	-1.89270	4.99590	-1.45380
C	4.13400	2.15430	2.17090
C	2.98290	2.82430	1.68390
C	2.57100	4.01570	2.31830
C	3.30360	4.54120	3.38500
C	4.44930	3.88870	3.84010
C	4.86420	2.69970	3.23870
H	1.67790	4.54050	2.00580
H	2.97970	5.45710	3.86240
H	5.01360	4.30190	4.66610
H	5.75160	2.20270	3.61240
C	1.36760	4.26310	-0.71940
C	0.24870	4.97890	-1.21540
C	0.42690	6.24010	-1.80610
C	1.70570	6.78440	-1.93050
C	2.81580	6.07530	-1.47270
C	2.65400	4.82340	-0.87520
H	-0.42200	6.80070	-2.17950
H	1.83730	7.75480	-2.39160
H	3.80690	6.49600	-1.58350
H	3.54170	4.29700	-0.55080
P	0.39600	-0.09920	-0.33820
O	1.86450	0.40980	-0.87690
O	-0.31230	1.18730	0.45900
O	-0.40560	-0.42460	-1.57720
O	0.53050	-1.12120	0.76540
H	-0.41740	-2.30080	1.91020
C	-2.22280	-3.31660	2.04930
N	-1.17800	-2.53650	2.55830
C	-1.13610	-5.89100	-1.24960
C	-2.11970	-6.27960	-0.33910
C	-2.47020	-5.44290	0.71640
C	-1.84040	-4.19540	0.89550
C	-0.85550	-3.81410	-0.03580
C	-0.50880	-4.65660	-1.09080
H	-0.86360	-6.54480	-2.07290
H	-2.61690	-7.23900	-0.44680
H	-3.23120	-5.78090	1.40760
H	-0.34510	-2.86030	0.04310
H	0.25550	-4.33350	-1.79070
C	-5.43870	-3.49290	0.06990
C	-5.86770	-3.91260	-1.19100
C	-5.22740	-3.47640	-2.36060
C	-4.14280	-2.60000	-2.30500
H	-5.95490	-3.82670	0.96530
H	-6.71830	-4.58270	-1.26970
H	-5.58720	-3.81810	-3.32600
H	-3.64850	-2.24790	-3.20420
C	-2.72200	-1.09520	0.61300
C	-4.35490	-2.61640	0.15060
C	-3.73340	-2.18280	-1.04420
H	-2.00060	-0.43010	1.06090
N	-2.72440	-1.26930	-0.69680
H	-1.88750	-0.91040	-1.25780
C	-1.27280	-1.62130	3.61510
O	-2.30730	-1.40550	4.23060
C	0.03400	-0.92290	3.92420
H	0.72070	-1.62410	4.41160
H	0.50980	-0.57150	3.00390
H	-0.16800	-0.09380	4.60190
C	-5.27660	1.70660	-0.32540
C	-4.58430	2.09570	0.82500
C	-3.25970	2.56480	0.76320
C	-2.59510	2.59400	-0.49660
C	-3.28490	2.17420	-1.67220
C	-4.61830	1.74120	-1.56040
H	-5.10050	2.07580	1.77590
H	-5.16430	1.45790	-2.44910
C	5.36260	-3.20620	-1.27200
C	4.77290	-3.26100	-0.00570
C	4.20700	-2.12010	0.59140
C	4.28960	-0.87190	-0.08870
C	4.91330	-0.80210	-1.36680

C	5.41840	-1.98000	-1.94440
H	4.74680	-4.20790	0.51720
H	5.88190	-1.93940	-2.92070
C	5.93550	-4.46610	-1.89670
H	5.79140	-5.32470	-1.20330
C	5.08750	0.51660	-2.12460
H	4.67970	1.37090	-1.54980
C	3.53060	-2.26460	1.95530
H	2.97690	-1.33920	2.21620
C	-2.65680	2.26030	-3.06650
H	-1.57310	2.48540	-2.99510
C	-2.61700	3.10940	2.04270
H	-1.59290	3.48580	1.85200
C	-6.74190	1.31490	-0.23160
H	-7.08560	1.40680	0.82310
C	-7.62200	2.25470	-1.06550
H	-7.43590	3.31030	-0.77290
H	-7.41810	2.14050	-2.15110
H	-8.69490	2.02960	-0.88510
C	-6.95450	-0.14510	-0.64060
H	-6.60890	-0.33130	-1.67950
H	-6.39680	-0.80370	0.05310
H	-8.03070	-0.41100	-0.57050
C	-3.30060	3.39400	-3.87500
H	-4.37390	3.18400	-4.07030
H	-3.22360	4.35450	-3.32500
H	-2.77970	3.51150	-4.84920
C	-2.75690	0.93730	-3.84180
H	-3.80590	0.69510	-4.11120
H	-2.16990	1.00720	-4.78250
H	-2.34240	0.10390	-3.24700
C	-3.40240	4.31100	2.58470
H	-3.51110	5.08190	1.79180
H	-4.41180	4.01090	2.93630
H	-2.85730	4.76790	3.43830
C	-2.47710	2.01940	3.11260
H	-3.46480	1.60940	3.41000
H	-1.84430	1.19440	2.73100
H	-1.98190	2.43360	4.01660
C	2.49120	-3.39780	1.95960
H	1.85480	-3.34240	1.05370
H	2.97600	-4.39620	1.98370
H	1.84040	-3.31330	2.85520
C	4.57290	-2.49010	3.05620
H	5.12560	-3.43990	2.89290
H	5.30220	-1.65390	3.07250
H	4.07470	-2.53420	4.04820
C	4.32660	0.49490	-3.45630
H	3.25770	0.24960	-3.28060
H	4.37710	1.49450	-3.93860
H	4.75570	-0.25360	-4.15510
C	6.57150	0.83930	-2.34260
H	7.05260	0.10270	-3.01970
H	6.67810	1.84930	-2.79320
H	7.10660	0.83650	-1.36880
C	5.20500	-4.81810	-3.19830
H	4.11360	-4.90720	-3.00960
H	5.37290	-4.04330	-3.97600
H	5.57050	-5.79240	-3.58740
C	7.44510	-4.33090	-2.13010
H	7.66730	-3.54540	-2.88320
H	7.95520	-4.07030	-1.17810
H	7.86090	-5.29470	-2.49380
C	-3.62930	-1.98670	1.25750
H	-4.06760	-1.73720	2.21540
C	-3.11640	-3.95200	3.10410
H	-3.35580	-3.23860	3.88670
H	-4.03740	-4.33470	2.66450
H	-2.57160	-4.79290	3.54950

TS6-*Z*

M06-2X/6-31G** Derived free energy = -3461.062765

M06-2X/6-31G** Derived free energy in solution = -3461.086195

ONIOM (B3LYP/6-31G**:UFF) Geometry

C	-2.61100	-1.80650	0.93780	O	0.19180	-0.15770	-1.99480
C	-4.57850	0.04850	1.70460	O	-0.46770	1.23950	0.12020
C	-3.85770	0.24970	0.52220	H	0.51640	2.26680	1.09290
C	-2.83590	-0.64890	0.16600	C	2.27650	3.24910	0.78930
H	-5.37700	0.73340	1.96460	N	1.32130	2.65860	1.61890
C	0.21370	-4.76730	-0.37640	C	4.77630	6.43100	2.31060
C	0.61030	-3.43590	-0.20970	C	3.89680	5.76650	3.16470
C	-0.33980	-2.46290	0.15020	C	3.11190	4.71480	2.69660
C	-1.67570	-2.83700	0.40360	C	3.18620	4.30140	1.35520
H	0.95610	-5.51700	-0.62240	C	4.06150	4.99800	0.50030
C	-4.27000	-1.01980	2.55460	C	4.85240	6.04200	0.97300
C	-3.27210	-1.95590	2.18290	H	5.38860	7.24780	2.68060
C	-2.95760	-3.00370	3.07440	H	3.81470	6.06690	4.20480
C	-3.64090	-3.13380	4.28550	H	2.43960	4.22360	3.38320
C	-4.63940	-2.22320	4.63050	H	4.13230	4.72410	-0.54390
C	-4.95370	-1.16900	3.77160	H	5.52110	6.55760	0.29010
H	-2.17720	-3.71890	2.85040	C	5.60140	2.59450	-1.17690
H	-3.39290	-3.94280	4.96040	C	6.06360	2.58610	-2.49440
H	-5.16620	-2.33000	5.57000	C	5.36670	1.91000	-3.50800
H	-5.72510	-0.46570	4.06220	C	4.19410	1.20640	-3.23020
C	-2.09460	-4.16470	0.13110	H	6.15750	3.10840	-0.40000
C	-1.12960	-5.13350	-0.24300	H	6.98600	3.10380	-2.73910
C	-1.52160	-6.46060	-0.48100	H	5.75430	1.92040	-4.52180
C	-2.86370	-6.82740	-0.37550	H	3.66210	0.65950	-4.00140
C	-3.82500	-5.87530	-0.03810	C	2.66130	0.73960	-0.03180
C	-3.44960	-4.55310	0.20990	C	3.65030	1.69010	0.35340
H	-0.79120	-7.21180	-0.75780	C	4.41720	1.91340	-0.87600
H	-3.16060	-7.85090	-0.56520	C	3.75010	1.21450	-1.91240
H	-4.86660	-6.16190	0.02930	H	1.88470	0.29450	0.56730
H	-4.22760	-3.84100	0.45020	H	4.06660	1.70290	1.35290
P	-0.54680	-0.03470	-0.68530	N	2.66850	0.52910	-1.33940
O	-2.11090	-0.44560	-0.98170	H	1.79390	0.13520	-1.79580
O	0.06200	-1.17260	0.38630	C	1.54010	1.97680	2.81900

O	2.63590	1.88170	3.35120
C	0.27480	1.35770	3.37910
H	-0.43120	2.14420	3.66700
H	-0.21670	0.74360	2.61780
H	0.53050	0.75780	4.25190
C	-5.07320	3.52720	-2.00150
C	-4.36960	3.76990	-0.81630
C	-3.91480	2.71720	-0.00280
C	-4.22830	1.37660	-0.36890
C	-4.96910	1.12310	-1.55860
C	-5.35760	2.20790	-2.36520
H	-4.16740	4.79250	-0.52960
H	-5.91050	2.02510	-3.27750
C	4.81030	-2.56980	-0.65770
C	4.01360	-2.80240	-1.78260
C	2.63320	-3.04790	-1.66900
C	2.04290	-3.08370	-0.37100
C	2.85280	-2.87370	0.78120
C	4.21900	-2.58790	0.61100
H	4.48170	-2.81610	-2.75770
H	4.84240	-2.42800	1.47980
C	2.31280	-3.04390	2.20430
H	1.23280	-3.29120	2.19940
C	1.83480	-3.35070	-2.94040
H	0.74710	-3.36760	-2.72330
C	6.30710	-2.36410	-0.81940
H	6.57640	-2.41550	-1.89820
C	-3.11010	3.05710	1.25300
H	-2.66520	2.13990	1.69160
C	-5.39490	-0.28690	-1.97560
H	-5.05610	-1.04530	-1.24340
C	-5.53270	4.67330	-2.88550
H	-6.06870	4.26790	-3.77270
C	-4.33780	5.47520	-3.41550
H	-3.79670	5.98420	-2.58990
H	-3.63180	4.80020	-3.94510
H	-4.68690	6.24590	-4.13540
C	-6.52170	5.58150	-2.14460
H	-6.03340	6.09590	-1.29000
H	-6.92140	6.35240	-2.83740

H	-7.37570	4.98200	-1.76270
C	-4.76930	-0.68050	-3.31980
H	-3.66510	-0.56990	-3.27360
H	-5.00110	-1.74250	-3.54980
H	-5.15800	-0.05010	-4.14690
C	-6.92320	-0.41400	-2.02070
H	-7.36060	0.22070	-2.81970
H	-7.21080	-1.46920	-2.21650
H	-7.35720	-0.11260	-1.04320
C	-4.01410	3.67730	2.32460
H	-4.44240	4.64090	1.97500
H	-4.84800	2.98880	2.57220
H	-3.43180	3.86260	3.25250
C	-1.93140	3.99450	0.94170
H	-2.27420	5.03340	0.75340
H	-1.23050	4.01940	1.80300
H	-1.38070	3.64270	0.04550
C	2.02770	-2.28130	-4.02780
H	3.04830	-2.31630	-4.46250
H	1.30040	-2.44710	-4.85120
H	1.85390	-1.26920	-3.61940
C	2.20820	-4.73290	-3.49150
H	3.27100	-4.75840	-3.81420
H	2.05640	-5.51340	-2.71810
H	1.56660	-4.98200	-4.36350
C	7.09670	-3.47260	-0.11220
H	6.76210	-4.46840	-0.47400
H	6.95930	-3.42530	0.98880
H	8.18000	-3.36910	-0.33570
C	6.73590	-0.98080	-0.32180
H	6.20490	-0.20000	-0.89970
H	7.82650	-0.83910	-0.47790
H	6.51170	-0.85000	0.75790
C	2.44850	-1.75330	3.02020
H	1.89560	-0.93540	2.52100
H	2.00770	-1.89240	4.03040
H	3.51020	-1.45250	3.13880
C	3.00350	-4.21500	2.91520
H	4.08150	-4.00830	3.08230
H	2.52500	-4.39720	3.90120

H	2.90420	-5.13940	2.30670	H	0.98870	2.81060	-0.89900
C	1.66790	3.59400	-0.55990	H	1.08930	4.51720	-0.44010
H	2.42750	3.77420	-1.31880				

TS5-Z

M06-2X/6-31G** Derived free energy = -3461.064613

M06-2X/6-31G** Derived free energy in solution = -3461.088103

ONIOM (B3LYP/6-31G**:UFF) Geometry

C	-3.39030	-0.95550	0.73520	H	-5.53630	-2.07900	-0.35160
C	-4.56630	1.45890	1.57910	P	-0.50230	-0.07220	-0.30690
C	-3.56170	1.48070	0.60620	O	-2.02160	0.29650	-0.80000
C	-2.95590	0.27750	0.20270	O	-0.62120	-1.42640	0.63740
H	-5.05190	2.38450	1.86320	O	0.24290	-0.38990	-1.58810
C	-1.73200	-4.66610	-0.68400	O	0.05840	0.95870	0.64220
C	-0.89010	-3.63130	-0.26220	H	1.48100	1.80290	0.49310
C	-1.44340	-2.40160	0.13610	C	3.32610	1.85260	1.37330
C	-2.84270	-2.21690	0.14800	N	2.42030	2.25730	0.41890
H	-1.30350	-5.62260	-0.95750	C	6.56870	4.42850	2.62340
C	-4.92360	0.26070	2.20750	C	5.44900	4.95240	1.97840
C	-4.31900	-0.95890	1.80830	C	4.43540	4.11140	1.52450
C	-4.65440	-2.14060	2.50390	C	4.51650	2.72070	1.70460
C	-5.59720	-2.11530	3.53400	C	5.63410	2.21220	2.38580
C	-6.21190	-0.91680	3.89510	C	6.65380	3.05200	2.82960
C	-5.87580	0.26800	3.23920	H	7.36190	5.08510	2.96790
H	-4.18520	-3.08630	2.26800	H	5.35940	6.02320	1.82270
H	-5.85000	-3.02910	4.05630	H	3.57270	4.54300	1.03650
H	-6.94260	-0.90390	4.69340	H	5.72430	1.14970	2.57770
H	-6.35190	1.19200	3.54560	H	7.51190	2.62660	3.34140
C	-3.67980	-3.22180	-0.40330	C	6.04890	0.90020	-1.02960
C	-3.11210	-4.46200	-0.79310	C	6.26740	1.12390	-2.38740
C	-3.93310	-5.48240	-1.29940	C	5.28200	0.83270	-3.34550
C	-5.30460	-5.27340	-1.44820	C	4.05370	0.28920	-2.97510
C	-5.86710	-4.04530	-1.10180	H	6.81160	1.14690	-0.29860
C	-5.06530	-3.02340	-0.58850	H	7.21600	1.53910	-2.71350
H	-3.51210	-6.43740	-1.59100	H	5.48330	1.02920	-4.39390
H	-5.93150	-6.06180	-1.84450	H	3.28890	0.05080	-3.70670
H	-6.92900	-3.88270	-1.23420	C	2.98910	-0.61180	0.32460

C	4.20860	0.04680	0.65770
C	4.82000	0.36660	-0.63430
C	3.85490	0.05660	-1.61690
H	2.23830	-1.02290	0.98270
H	4.77030	-0.22550	1.53940
N	2.74830	-0.51840	-0.97430
H	1.74520	-0.59980	-1.35860
C	2.62460	2.94740	-0.79260
O	3.69970	3.38630	-1.15350
C	1.34570	3.09530	-1.59520
H	0.54150	3.51620	-0.98440
H	0.99870	2.11620	-1.94250
H	1.54750	3.74100	-2.44920
C	3.37870	-4.27580	-0.17940
C	2.65580	-4.14950	1.01220
C	1.26180	-3.96390	1.01110
C	0.58130	-3.84380	-0.23530
C	1.31440	-3.95760	-1.45500
C	2.70000	-4.19210	-1.39790
H	3.18230	-4.23110	1.95310
H	3.26050	-4.31720	-2.31460
C	-2.39770	5.25120	-1.14420
C	-2.07890	4.96070	0.18490
C	-2.42100	3.72900	0.77100
C	-3.15290	2.77580	0.00210
C	-3.51710	3.08290	-1.34040
C	-3.11190	4.31020	-1.89400
H	-1.54790	5.70180	0.76760
H	-3.37260	4.54630	-2.91660
C	-4.37400	2.14600	-2.19470
H	-4.63220	1.22090	-1.64330
C	-2.00790	3.47190	2.22250
H	-2.16620	2.40740	2.49080
C	-1.97450	6.57670	-1.75250
H	-1.42740	7.17810	-0.99240
C	-1.01130	6.36180	-2.92610
H	-0.13400	5.76760	-2.59310
H	-1.50970	5.82850	-3.76320
H	-0.64420	7.34110	-3.30070
C	-3.19240	7.40380	-2.18180
H	-3.74410	6.90910	-3.00900
H	-3.88170	7.54050	-1.32120
H	-2.86640	8.40790	-2.52780
C	-0.51320	3.74530	2.45820
H	0.09460	3.31060	1.64350
H	-0.29890	4.83370	2.50650
H	-0.19120	3.28890	3.41850
C	-2.85690	4.31460	3.18210
H	-2.68530	5.39960	3.01630
H	-3.93560	4.10290	3.03430
H	-2.59760	4.07040	4.23450
C	-3.62610	1.70120	-3.45940
H	-3.46520	2.55130	-4.15510
H	-2.63900	1.26930	-3.19180
H	-4.21220	0.92160	-3.99130
C	-5.71430	2.80090	-2.55290
H	-5.57100	3.68700	-3.20640
H	-6.36220	2.07320	-3.08690
H	-6.24010	3.11840	-1.62700
C	0.52430	-3.97380	2.35310
H	-0.56620	-3.83780	2.21430
C	0.98920	-2.82960	3.26560
H	0.38720	-2.82160	4.19920
H	2.05880	-2.93760	3.54170
H	0.84690	-1.85180	2.76260
C	0.68450	-5.32910	3.05430
H	1.74010	-5.51210	3.34600
H	0.05770	-5.35970	3.97120
H	0.35080	-6.14670	2.37990
C	0.64240	-3.88900	-2.83000
H	-0.40830	-3.54780	-2.73750
C	1.32290	-2.87950	-3.76880
H	1.43020	-1.89620	-3.27560
H	2.32740	-3.22660	-4.08830
H	0.70460	-2.73840	-4.68110
C	0.61210	-5.27720	-3.48240
H	1.64040	-5.63830	-3.69730
H	0.11470	-6.00960	-2.81390
H	0.04350	-5.23810	-4.43600
C	4.87610	-4.53210	-0.16280

H	5.24590	-4.65010	-1.20590	H	4.63340	-6.67850	0.13670
C	5.63270	-3.34570	0.44340	H	6.29160	-6.06400	0.46990
H	5.43940	-2.43390	-0.15780	C	2.62910	1.32510	2.62540
H	5.32100	-3.16140	1.49340	H	3.30400	0.71750	3.23100
H	6.72620	-3.54050	0.42660	H	2.33320	2.18920	3.23140
C	5.20950	-5.83530	0.57440	H	1.72620	0.76440	2.37770
H	4.97070	-5.75930	1.65620				

TS8-*E*

M06-2X/6-31G** Derived free energy = -4259.080428

M06-2X/6-31G** Derived free energy in solution = -4259.111087

ONIOM (B3LYP/6-31G**:UFF) Geometry

C	-1.22170	-2.53650	0.87110	C	-0.73690	-5.36090	0.20190
C	-3.82790	-1.76910	1.58160	H	2.82210	-6.57230	-0.75680
C	-3.28020	-1.32390	0.37260	H	0.98210	-8.19360	-0.55660
C	-1.95160	-1.65960	0.04960	H	-1.29510	-7.43200	0.03540
H	-4.84180	-1.48880	1.84520	H	-1.75110	-5.06840	0.43800
C	2.64130	-3.93580	-0.38420	P	-0.22600	0.01200	-0.63610
C	2.42180	-2.56570	-0.20050	O	-1.33710	-1.12160	-1.05110
C	1.12490	-2.09890	0.10730	O	0.91850	-0.75320	0.31510
C	0.08980	-3.03070	0.36680	O	0.42740	0.48730	-1.91440
H	3.62240	-4.29510	-0.66550	O	-0.83000	1.04900	0.28470
C	-3.07090	-2.54260	2.46930	H	-0.54910	2.74590	0.65460
C	-1.75290	-2.93130	2.12340	Si	-4.35150	-0.26740	-0.73460
C	-1.00040	-3.68460	3.04930	Si	3.85850	-1.39050	-0.45100
C	-1.55620	-4.06300	4.27340	C	3.05010	0.89760	-4.44210
C	-2.86170	-3.69350	4.59680	C	3.74400	1.53580	-3.41210
C	-3.61740	-2.93510	3.70160	C	3.97510	0.87130	-2.20380
H	0.02120	-3.97410	2.84150	C	3.51290	-0.44060	-2.01740
H	-0.97060	-4.64230	4.97560	C	2.83930	-1.08450	-3.06870
H	-3.28710	-3.99000	5.54690	C	2.60020	-0.41320	-4.27130
H	-4.62670	-2.65030	3.97460	H	2.86530	1.41710	-5.37340
C	0.30580	-4.41080	0.11670	H	4.09650	2.55020	-3.54630
C	1.59840	-4.85520	-0.25000	H	4.49820	1.39660	-1.42250
C	1.83430	-6.22020	-0.48320	H	2.49560	-2.10530	-2.96180
C	0.79470	-7.14350	-0.37260	H	2.06960	-0.91140	-5.07240
C	-0.48810	-6.71410	-0.03660	C	4.60410	1.22630	3.34660

C	5.40210	1.39160	2.21260
C	5.18420	0.59960	1.08130
C	4.13500	-0.33300	1.05950
C	3.36810	-0.52450	2.22080
C	3.60090	0.25530	3.35600
H	4.77820	1.83340	4.22550
H	6.20100	2.12160	2.21600
H	5.84890	0.70360	0.23450
H	2.60310	-1.28560	2.26680
H	3.01070	0.09600	4.24770
C	7.83310	-3.77840	-0.94080
C	7.34570	-3.02100	-2.00950
C	6.15370	-2.30240	-1.87430
C	5.44070	-2.34210	-0.66590
C	5.93790	-3.10010	0.40510
C	7.12960	-3.81740	0.26600
H	8.75580	-4.33390	-1.04750
H	7.89240	-2.99000	-2.94310
H	5.79560	-1.72160	-2.71330
H	5.40240	-3.13870	1.34580
H	7.50800	-4.40300	1.09380
C	-4.71510	3.99630	1.15130
C	-3.94400	3.01780	1.78280
C	-3.84070	1.73980	1.22620
C	-4.50070	1.43320	0.02460
C	-5.27230	2.42370	-0.60400
C	-5.38160	3.69820	-0.03980
H	-4.79280	4.98580	1.58250
H	-3.42350	3.25000	2.70300
H	-3.23990	1.00040	1.73580
H	-5.78770	2.21070	-1.53230
H	-5.97660	4.45730	-0.53070
C	-2.47430	-0.07120	-5.01720
C	-2.71610	1.10820	-4.31030
C	-3.29840	1.05800	-3.04060
C	-3.64180	-0.17530	-2.46300
C	-3.39650	-1.35600	-3.18410
C	-2.81540	-1.30260	-4.45440
H	-2.01960	-0.03110	-5.99850
H	-2.44870	2.06300	-4.74460

H	-3.46780	1.98450	-2.51820
H	-3.61680	-2.32200	-2.75610
H	-2.61950	-2.21770	-4.99820
C	-8.50090	-2.41810	-0.88000
C	-8.43410	-1.06690	-0.53370
C	-7.19840	-0.41390	-0.48850
C	-6.01780	-1.10650	-0.80550
C	-6.09330	-2.47140	-1.12770
C	-7.33020	-3.12120	-1.17090
H	-9.45790	-2.92260	-0.91150
H	-9.34020	-0.52580	-0.29360
H	-7.17740	0.62410	-0.19610
H	-5.19820	-3.04540	-1.31900
H	-7.37970	-4.17310	-1.42110
C	0.57430	4.05840	1.80200
N	-0.40880	3.74990	0.86700
C	0.70300	1.47290	5.28430
C	-0.23310	1.24800	4.27790
C	-0.26490	2.05620	3.14120
C	0.65110	3.10900	2.98340
C	1.60550	3.30910	3.99770
C	1.62860	2.50750	5.13530
H	0.72100	0.84570	6.17050
H	-0.95300	0.44010	4.36960
H	-0.99370	1.83940	2.37390
H	2.34320	4.10040	3.91280
H	2.37520	2.68900	5.90250
C	2.75820	5.94840	-0.53250
C	2.61520	6.47440	-1.81730
C	2.12040	5.69450	-2.87450
C	1.77380	4.35730	-2.68220
H	3.14170	6.56560	0.27410
H	2.88950	7.50800	-2.00380
H	2.01470	6.13620	-3.86020
H	1.40950	3.73670	-3.49450
C	2.06490	2.44460	0.33080
C	2.35880	3.74260	0.86690
C	2.40520	4.61460	-0.31320
C	1.93780	3.84450	-1.39890
H	1.94180	1.51080	0.86100

H	3.01590	3.86400	1.71840	H	-1.28280	3.97540	-2.00760
N	1.73100	2.53290	-0.94340	H	-2.00610	2.84150	-0.85760
H	1.25720	1.71810	-1.47770	C	0.71190	5.52210	2.18640
C	-1.09650	4.62010	0.01080	H	1.55630	5.66330	2.86100
O	-1.09140	5.83500	0.11290	H	0.80800	6.16910	1.32050
C	-1.85960	3.89850	-1.07990	H	-0.19980	5.82080	2.71410
H	-2.81020	4.40830	-1.24180				

TS7-*E*

M06-2X/6-31G** Derived free energy = -4259.081315

M06-2X/6-31G** Derived free energy in solution = -4259.110350

ONIOM (B3LYP/6-31G**:UFF) Geometry

C	-0.98500	-2.97230	0.40830	H	3.36780	-6.07750	-2.18800
C	-3.58580	-2.55810	1.39150	H	1.66470	-7.82940	-2.48570
C	-3.12430	-1.79940	0.31010	H	-0.66660	-7.44350	-1.75570
C	-1.79960	-1.96410	-0.13450	H	-1.31480	-5.32220	-0.72830
H	-4.59460	-2.40870	1.75940	P	-0.20210	-0.06360	-0.51800
C	2.97630	-3.67380	-1.09090	O	-1.28320	-1.15270	-1.10870
C	2.64130	-2.43370	-0.53600	O	0.96370	-0.92770	0.31590
C	1.30110	-2.16000	-0.19390	O	0.38340	0.60870	-1.73740
C	0.34760	-3.20420	-0.21810	O	-0.80760	0.79570	0.56910
H	3.99710	-3.87210	-1.39390	H	-0.72290	2.24040	1.77070
C	-2.75260	-3.49500	2.01540	Si	-4.29040	-0.58300	-0.49590
C	-1.43770	-3.71010	1.52990	Si	4.00840	-1.20900	-0.17430
C	-0.60880	-4.63560	2.19910	C	4.09270	2.10710	-3.46460
C	-1.08670	-5.34810	3.30110	C	3.19050	1.04560	-3.56290
C	-2.38870	-5.14590	3.75820	C	3.16500	0.05360	-2.57770
C	-3.21930	-4.22240	3.12190	C	4.02760	0.12900	-1.47380
H	0.41310	-4.80580	1.88740	C	4.95980	1.17710	-1.40470
H	-0.44270	-6.05720	3.80530	C	4.98080	2.16970	-2.38860
H	-2.75310	-5.70100	4.61290	H	4.11030	2.87540	-4.22650
H	-4.22460	-4.07260	3.49780	H	2.51090	0.99010	-4.40340
C	0.67810	-4.44170	-0.82560	H	2.47130	-0.76940	-2.68380
C	2.00560	-4.66520	-1.26590	H	5.68970	1.21510	-0.60710
C	2.35300	-5.89100	-1.85670	H	5.69390	2.98120	-2.32310
C	1.39150	-6.88760	-2.02740	C	3.35580	0.29420	4.20500
C	0.07770	-6.67060	-1.61350	C	3.82340	1.18360	3.23550
C	-0.28190	-5.45860	-1.01980	C	4.01020	0.75290	1.91890

C	3.73580	-0.57670	1.56220
C	3.26000	-1.46220	2.54430
C	3.06810	-1.02660	3.85790
H	3.20750	0.63110	5.22270
H	4.03630	2.21040	3.50320
H	4.35780	1.46750	1.19260
H	3.02930	-2.49040	2.29680
H	2.69680	-1.71400	4.60680
C	8.08310	-3.49310	-0.19470
C	7.82880	-2.52060	-1.16460
C	6.61270	-1.83040	-1.16440
C	5.64330	-2.10480	-0.18560
C	5.90220	-3.09240	0.77800
C	7.11890	-3.78070	0.77420
H	9.02420	-4.02770	-0.19760
H	8.57250	-2.30480	-1.92080
H	6.43290	-1.09720	-1.93760
H	5.16440	-3.33880	1.52870
H	7.31280	-4.54000	1.52080
C	-4.26520	3.46790	1.82610
C	-4.89230	3.37460	0.58160
C	-4.90110	2.15900	-0.10850
C	-4.28010	1.02700	0.44270
C	-3.65870	1.12560	1.69710
C	-3.65320	2.34360	2.38500
H	-4.25160	4.41130	2.35620
H	-5.36480	4.24620	0.14760
H	-5.38690	2.10280	-1.07470
H	-3.17730	0.26760	2.14630
H	-3.17130	2.41850	3.35050
C	-2.90270	0.15150	-4.89610
C	-2.58790	1.07460	-3.89650
C	-3.01800	0.85760	-2.58500
C	-3.76960	-0.28180	-2.26150
C	-4.06720	-1.21350	-3.26800
C	-3.63980	-0.99300	-4.58130
H	-2.56790	0.31800	-5.91180
H	-2.00120	1.95260	-4.13440
H	-2.73000	1.55700	-1.81630
H	-4.62320	-2.11170	-3.04670
H	-3.87530	-1.71300	-5.35430
C	-8.56000	-2.47560	-0.41470
C	-8.38650	-1.14090	-0.04040
C	-7.11250	-0.56480	-0.06510
C	-6.00230	-1.32050	-0.47520
C	-6.18360	-2.66540	-0.83590
C	-7.45830	-3.23820	-0.80910
H	-9.54650	-2.92020	-0.39290
H	-9.23950	-0.55260	0.27240
H	-7.00440	0.46630	0.23730
H	-5.33820	-3.27830	-1.12180
H	-7.59090	-4.27540	-1.08870
C	0.14570	4.10950	1.98300
N	-0.26400	2.85660	2.45220
C	-2.32420	5.84510	-1.11530
C	-2.13210	4.46590	-1.07060
C	-1.34460	3.88140	-0.07840
C	-0.71440	4.68500	0.89260
C	-0.91330	6.07830	0.82790
C	-1.71340	6.65040	-0.15610
H	-2.94590	6.28630	-1.88870
H	-2.60660	3.84050	-1.81770
H	-1.23230	2.80090	-0.07370
H	-0.45800	6.73330	1.55830
H	-1.85640	7.72670	-0.16960
C	2.64700	6.08460	-0.09250
C	2.71750	6.70210	-1.34330
C	2.37680	6.01220	-2.51630
C	1.96820	4.67860	-2.47480
H	2.92160	6.63060	0.80530
H	3.04590	7.73470	-1.41080
H	2.44010	6.52040	-3.47330
H	1.71220	4.13270	-3.37680
C	1.71990	2.57480	0.40680
C	2.00180	3.80650	1.07120
C	2.23460	4.75250	-0.02510
C	1.91780	4.07270	-1.22510
H	1.49380	1.61630	0.84840
H	2.55050	3.81950	2.00440
N	1.59900	2.74490	-0.89630

H	1.15990	1.95120	-1.46820	H	-1.37370	0.86800	4.10250
C	0.25940	2.14900	3.54390	C	0.60120	5.09290	3.04990
O	1.11670	2.59000	4.29470	H	1.10490	5.94810	2.60010
C	-0.35080	0.77410	3.72210	H	-0.28520	5.45490	3.58470
H	-0.39830	0.23910	2.76960	H	1.25500	4.61110	3.76880
H	0.24640	0.22850	4.45210				

TS7-Z

M06-2X/6-31G** Derived free energy = -4259.074258

M06-2X/6-31G** Derived free energy in solution = -4259.106950

ONIOM (B3LYP/6-31G**:UFF) Geometry

C	-1.06120	-2.97290	0.65750	H	3.26600	-6.56340	-1.27930
C	-3.63460	-2.32620	1.58690	H	1.53710	-8.31550	-1.27720
C	-3.16410	-1.74650	0.40320	H	-0.79740	-7.76810	-0.67080
C	-1.85880	-2.04300	-0.03610	H	-1.42350	-5.48360	-0.06370
H	-4.63390	-2.09030	1.93650	P	-0.22400	-0.31970	-0.84870
C	2.90150	-3.99270	-0.65120	O	-1.35360	-1.46380	-1.16530
C	2.57850	-2.66650	-0.34400	O	0.91280	-1.00750	0.16980
C	1.23840	-2.31850	-0.08170	O	0.36360	0.01440	-2.19910
C	0.26720	-3.33410	0.07710	O	-0.77660	0.79450	0.00580
H	3.92350	-4.25710	-0.89320	H	-0.33760	1.94790	1.15750
C	-2.82250	-3.18090	2.34130	Si	-4.30760	-0.55590	-0.48670
C	-1.51770	-3.50260	1.89010	Si	3.95410	-1.40510	-0.19540
C	-0.70260	-4.32260	2.70000	C	8.03160	-3.64880	0.21550
C	-1.18740	-4.83720	3.90440	C	7.79270	-2.84210	-0.89950
C	-2.48230	-4.53750	4.32610	C	6.57500	-2.16720	-1.02950
C	-3.29710	-3.71050	3.55210	C	5.58790	-2.29000	-0.03760
H	0.31430	-4.56140	2.41930	C	5.83180	-3.11260	1.07370
H	-0.55430	-5.46780	4.51520	C	7.05020	-3.78620	1.19990
H	-2.85240	-4.93910	5.26060	H	8.97420	-4.17150	0.31390
H	-4.29530	-3.47770	3.90400	H	8.55010	-2.74290	-1.66630
C	0.58510	-4.66600	-0.28870	H	6.40900	-1.56380	-1.91040
C	1.91550	-4.98550	-0.65780	H	5.08150	-3.24340	1.84010
C	2.24990	-6.30430	-1.00580	H	7.23220	-4.41760	2.05990
C	1.27370	-7.30140	-1.00570	C	4.03200	1.31910	-3.99170
C	-0.04200	-6.99290	-0.66130	C	3.16500	0.22590	-3.93120
C	-0.38920	-5.68720	-0.30790	C	3.15110	-0.59600	-2.79990

C	3.98760	-0.31530	-1.70960
C	4.89670	0.75080	-1.80290
C	4.90590	1.57440	-2.93260
H	4.03830	1.95780	-4.86540
H	2.50140	0.01580	-4.75990
H	2.48320	-1.44660	-2.78050
H	5.61130	0.93730	-1.01210
H	5.59760	2.40470	-2.99070
C	3.23750	0.87350	3.82860
C	3.01780	-0.50240	3.74210
C	3.22780	-1.17040	2.53270
C	3.66310	-0.46740	1.39650
C	3.86690	0.91890	1.49120
C	3.65590	1.58390	2.70220
H	3.07090	1.39110	4.76430
H	2.67900	-1.05130	4.61120
H	3.03710	-2.23460	2.48460
H	4.17640	1.50010	0.63980
H	3.81660	2.65190	2.76640
C	-5.63230	2.51790	2.77090
C	-6.59960	1.78970	2.07300
C	-6.21440	0.86790	1.09410
C	-4.85500	0.67670	0.80030
C	-3.89030	1.39750	1.52060
C	-4.27720	2.31930	2.49640
H	-5.93240	3.23060	3.52800
H	-7.64910	1.93700	2.29310
H	-6.98030	0.30690	0.57860
H	-2.83940	1.24790	1.33000
H	-3.52570	2.87640	3.04120
C	-2.28300	1.57570	-4.11510
C	-2.62710	2.31590	-2.98170
C	-3.25570	1.69100	-1.89990
C	-3.52080	0.31300	-1.93590
C	-3.20600	-0.41660	-3.09340
C	-2.58080	0.21200	-4.17460
H	-1.79250	2.06020	-4.94930
H	-2.40430	3.37440	-2.94000
H	-3.51850	2.28580	-1.03760
H	-3.42280	-1.47620	-3.14970
H	-2.32230	-0.35970	-5.05640
C	-7.95470	-2.81860	-2.31230
C	-7.81870	-1.43930	-2.48940
C	-6.72660	-0.76650	-1.93370
C	-5.76800	-1.46930	-1.18790
C	-5.90240	-2.85720	-1.02560
C	-6.99590	-3.52700	-1.58310
H	-8.79970	-3.33900	-2.74430
H	-8.55790	-0.89180	-3.05960
H	-6.62980	0.30180	-2.08440
H	-5.16560	-3.42810	-0.47810
H	-7.09770	-4.59670	-1.45360
C	0.05340	3.94040	1.04180
N	-0.00210	2.73520	1.74670
C	1.00720	7.69500	2.95800
C	-0.10900	7.58100	2.13370
C	-0.41100	6.36120	1.52480
C	0.38680	5.22600	1.74350
C	1.50920	5.35990	2.58160
C	1.81650	6.57640	3.17750
H	1.24820	8.64450	3.42680
H	-0.74980	8.43950	1.95670
H	-1.28630	6.30020	0.89050
H	2.13740	4.50230	2.77990
H	2.68820	6.65080	3.82030
C	1.58340	5.96870	-1.69100
C	1.27450	6.39260	-2.98620
C	0.93260	5.47660	-3.99320
C	0.90030	4.10460	-3.73880
H	1.83520	6.68820	-0.91860
H	1.29860	7.45270	-3.21990
H	0.69370	5.84000	-4.98770
H	0.64120	3.38640	-4.50960
C	1.63790	2.45180	-0.65500
C	1.73280	3.80160	-0.19180
C	1.55200	4.60070	-1.41230
C	1.22130	3.69590	-2.44950
H	1.71010	1.54320	-0.07850
H	2.43800	4.06450	0.58390
N	1.28770	2.39590	-1.92630

H	0.91780	1.45530	-2.29660	H	0.13400	0.27590	2.58240
C	0.17610	2.39730	3.08870	C	-1.06930	3.98270	0.02000
O	0.57240	3.14590	3.96460	H	-0.95670	4.79570	-0.69460
C	-0.22420	0.95410	3.36200	H	-1.12450	3.02880	-0.50520
H	0.16940	0.66650	4.33660	H	-2.01610	4.10710	0.55900
H	-1.31640	0.87430	3.38570				

TS8-Z

M06-2X/6-31G** Derived free energy = -4259.075118

M06-2X/6-31G** Derived free energy in solution = -4259.106832

ONIOM (B3LYP/6-31G**:UFF) Geometry

C	1.54400	-2.64200	-1.01610	H	-2.12420	-7.09680	0.38380
C	4.02850	-1.56380	-1.76750	H	-0.12520	-8.52280	0.21980
C	3.46080	-1.20530	-0.53970	H	2.08700	-7.52390	-0.25540
C	2.19000	-1.70370	-0.19210	H	2.32160	-5.11260	-0.57230
H	4.99330	-1.15820	-2.05060	P	0.30930	-0.27460	0.64410
C	-2.19230	-4.44440	0.08200	O	1.56160	-1.28750	0.95160
C	-2.09870	-3.05380	-0.04870	O	-0.74810	-1.08610	-0.37220
C	-0.83600	-2.45420	-0.25050	O	-0.35290	0.00000	1.97190
C	0.29000	-3.27200	-0.51250	O	0.74910	0.89270	-0.21200
H	-3.14840	-4.90610	0.29130	H	0.41350	2.52780	-0.35290
C	3.35300	-2.41720	-2.64710	Si	4.41640	-0.03400	0.55900
C	2.09520	-2.95990	-2.28300	Si	-3.66150	-2.03020	0.11670
C	1.41850	-3.78450	-3.20750	C	-7.39400	-4.82300	0.15250
C	1.99470	-4.08730	-4.44310	C	-6.59730	-4.72320	-0.99120
C	3.24400	-3.56860	-4.78190	C	-5.47930	-3.88410	-0.99470
C	3.92080	-2.73410	-3.89160	C	-5.15080	-3.14100	0.14920
H	0.43890	-4.18910	-2.99090	C	-5.95530	-3.24330	1.29470
H	1.46790	-4.72380	-5.14240	C	-7.07320	-4.08340	1.29420
H	3.68550	-3.80690	-5.74100	H	-8.25940	-5.47290	0.15430
H	4.88550	-2.33200	-4.17780	H	-6.84610	-5.29600	-1.87510
C	0.20070	-4.67300	-0.31190	H	-4.86950	-3.81640	-1.88730
C	-1.05650	-5.25260	-0.01710	H	-5.72360	-2.68190	2.18960
C	-1.16550	-6.64100	0.16610	H	-7.69080	-4.16140	2.17950
C	-0.03540	-7.45400	0.07460	C	-3.44590	0.17100	4.23740
C	1.21110	-6.89080	-0.19570	C	-4.28300	0.66360	3.23460
C	1.33410	-5.51270	-0.38470	C	-4.34390	0.02280	1.99330

C	-3.55750	-1.11310	1.74080
C	-2.74250	-1.61930	2.76730
C	-2.68050	-0.97340	4.00560
H	-3.39470	0.67270	5.19490
H	-4.87960	1.54830	3.41570
H	-4.99560	0.43050	1.24020
H	-2.15070	-2.51240	2.61670
H	-2.03930	-1.36180	4.78620
C	-4.30030	0.78410	-3.56650
C	-5.25020	0.73440	-2.54460
C	-5.06720	-0.12730	-1.45910
C	-3.91250	-0.92110	-1.36760
C	-2.99620	-0.91210	-2.43370
C	-3.18220	-0.05050	-3.51850
H	-4.44170	1.45250	-4.40590
H	-6.13420	1.35630	-2.59820
H	-5.84080	-0.18670	-0.70680
H	-2.14170	-1.57110	-2.44330
H	-2.46050	-0.03350	-4.32490
C	2.90440	-0.49270	4.96470
C	3.33270	-1.61060	4.24640
C	3.79970	-1.46560	2.93680
C	3.84390	-0.19790	2.33250
C	3.40990	0.91950	3.06420
C	2.94230	0.77130	4.37310
H	2.53900	-0.60630	5.97710
H	3.29280	-2.59230	4.70050
H	4.08720	-2.35040	2.38870
H	3.42390	1.90690	2.63570
H	2.60640	1.63790	4.92800
C	8.89490	-1.34960	0.27510
C	8.54030	-0.03150	-0.01970
C	7.20390	0.37150	0.06420
C	6.21080	-0.54080	0.45860
C	6.57470	-1.86980	0.73000
C	7.91150	-2.26950	0.64430
H	9.92930	-1.66060	0.20660
H	9.30080	0.67780	-0.31990
H	6.95810	1.39060	-0.19150
H	5.82790	-2.60770	0.98310

H	8.18350	-3.29550	0.85590
C	3.84140	4.33180	-1.02110
C	3.22880	3.26480	-1.68140
C	3.41040	1.95750	-1.22030
C	4.19500	1.70860	-0.08170
C	4.81890	2.78660	0.56700
C	4.64230	4.09200	0.09810
H	3.69660	5.34350	-1.37730
H	2.60940	3.45080	-2.54940
H	2.92910	1.14920	-1.75280
H	5.44070	2.61780	1.43730
H	5.12060	4.91840	0.60730
C	-0.89100	3.67500	-1.41620
N	0.07010	3.50880	-0.44660
C	-1.51850	7.50040	-3.32240
C	-0.31070	7.20590	-2.69030
C	-0.13850	5.99490	-2.02450
C	-1.17340	5.04610	-1.97540
C	-2.37040	5.34180	-2.64590
C	-2.54730	6.55930	-3.30100
H	-1.65450	8.44960	-3.83190
H	0.50530	7.92180	-2.70990
H	0.80890	5.78250	-1.54820
H	-3.18270	4.62500	-2.66200
H	-3.49000	6.76680	-3.79840
C	-3.43550	5.21790	0.93260
C	-3.38810	5.73110	2.22690
C	-2.82490	4.99800	3.28490
C	-2.30570	3.72080	3.08180
H	-3.85870	5.80090	0.12120
H	-3.78920	6.72040	2.42360
H	-2.80100	5.43060	4.28020
H	-1.88600	3.13730	3.89490
C	-2.28070	1.85570	0.02110
C	-2.74890	3.10060	-0.48330
C	-2.91350	3.94250	0.70260
C	-2.36970	3.21600	1.78590
H	-2.04510	0.94780	-0.51220
H	-3.38220	3.14970	-1.35700
N	-1.97560	1.95880	1.30690

H	-1.39580	1.20000	1.78480
C	0.39320	4.34920	0.63690
O	-0.03140	5.48040	0.77250
C	1.34400	3.69970	1.62140
H	2.22140	4.34010	1.74140
H	0.84680	3.64880	2.59400
H	1.63960	2.69630	1.31700
C	-0.80280	2.61060	-2.50480
H	-0.00340	2.90860	-3.19340
H	-0.54950	1.63050	-2.09760
H	-1.72710	2.56210	-3.08280

TS10-*E*

M06-2X/6-31G** Derived free energy = -3216.017786

M06-2X/6-31G** Derived free energy in solution = -3216.051742

ONIOM (B3LYP/6-31G**:UFF) Geometry

C	2.19460	3.32980	0.19710
C	4.48640	2.27580	1.44640
C	3.79520	1.50050	0.50560
C	2.63570	2.02140	-0.10130
H	5.38030	1.87680	1.91330
C	-1.40160	4.69400	-1.74020
C	-1.41180	3.53670	-0.95140
C	-0.21650	3.09260	-0.35810
C	0.97560	3.83310	-0.50140
H	-2.32240	5.03160	-2.20340
C	4.02800	3.54980	1.80330
C	2.86230	4.08270	1.19610
C	2.38790	5.34040	1.62810
C	3.07920	6.06450	2.60180
C	4.24220	5.54580	3.16980
C	4.71390	4.29250	2.77800
H	1.48030	5.77000	1.22650
H	2.70910	7.03070	2.91970
H	4.77340	6.11160	3.92420
H	5.61010	3.89960	3.24370
C	0.99400	4.97100	-1.34740
C	-0.21380	5.40560	-1.95210
C	-0.21560	6.54090	-2.77860
C	0.97010	7.23390	-3.02590
C	2.16730	6.79680	-2.46000
C	2.18560	5.67200	-1.63230
H	-1.13260	6.88710	-3.24080
H	0.96260	8.10590	-3.66700
H	3.08660	7.32950	-2.66650
H	3.13720	5.35450	-1.22820
P	0.51480	0.66850	-0.44880
O	1.94080	1.26360	-1.01200
O	-0.20300	1.92270	0.36020
O	-0.27320	0.33450	-1.70580
C	-1.74700	-2.98050	1.77240
N	-0.44640	-2.85860	1.29730
C	-3.22610	0.37720	4.09340
C	-4.03980	-0.72950	3.84730
C	-3.55630	-1.80290	3.10390
C	-2.24790	-1.80250	2.58620
C	-1.43550	-0.68690	2.85260
C	-1.92630	0.39000	3.59180
H	-3.60420	1.21930	4.66520
H	-5.05680	-0.75770	4.22690
H	-4.22210	-2.63920	2.91900
H	-0.42470	-0.62650	2.47000
H	-1.27960	1.24440	3.76410
C	-2.55430	-5.18630	-1.12500
C	-1.82780	-5.74090	-2.17890
C	-0.99770	-4.94940	-2.98910

C	-0.88560	-3.57450	-2.78140
H	-3.18890	-5.81290	-0.50600
H	-1.90150	-6.80590	-2.37520
H	-0.44050	-5.41370	-3.79670
H	-0.25970	-2.94890	-3.40920
C	-2.58290	-1.60160	-0.33280
C	-2.98220	-2.90040	0.12450
C	-2.44750	-3.81280	-0.88920
C	-1.62760	-3.03300	-1.73510
H	-2.76870	-0.64810	0.13820
H	-3.92210	-3.06250	0.63550
C	0.46290	-3.88790	1.00180
O	0.26440	-5.06620	1.24350
C	1.74120	-3.40810	0.34580
H	1.93070	-2.34470	0.49460
H	2.56350	-4.01780	0.72430
H	1.66040	-3.59370	-0.73130
C	-5.00470	1.20930	-0.53030
C	-4.29780	1.63920	0.60680
C	-2.66380	2.76000	-0.80080
H	-4.64470	1.38410	1.60000
C	5.05960	-2.52790	-0.34570
C	4.95590	-2.07530	0.98100
C	4.25950	0.12270	0.20680
H	5.14750	-2.74630	1.80890
C	-2.15090	-4.34020	2.31730
H	-3.19490	-4.33470	2.62840
H	-1.97340	-5.13760	1.60270
H	-1.53380	-4.55400	3.19640
C	6.13610	-6.60390	-1.16940
C	6.80270	-5.89460	-0.16770
C	6.45620	-4.56700	0.10140
C	5.43730	-3.93370	-0.63460
C	4.77250	-4.65890	-1.64150
C	5.12160	-5.98690	-1.90480
H	6.40530	-7.63180	-1.37520
H	7.59050	-6.37310	0.39980
H	6.99460	-4.03110	0.87300
H	3.97040	-4.20580	-2.21050
H	4.60230	-6.53910	-2.67730
C	-8.47980	-1.28480	-0.14100
C	-7.36190	-1.51150	0.66510
C	-6.23320	-0.69670	0.54240
C	-6.20990	0.35500	-0.39160
C	-7.34110	0.57260	-1.20020
C	-8.46940	-0.24360	-1.07230
H	-9.35350	-1.91630	-0.04470
H	-7.36760	-2.32160	1.38290
H	-5.37150	-0.90000	1.16150
H	-7.35820	1.38200	-1.91920
H	-9.33720	-0.06730	-1.69480
C	-3.13530	2.40280	0.47240
H	-2.60510	2.71860	1.36250
C	-3.37970	2.35080	-1.93620
H	-3.02600	2.60490	-2.92790
C	-4.53680	1.57960	-1.80290
H	-5.05000	1.24870	-2.69710
C	4.38920	-0.32040	-1.11960
H	4.19360	0.35180	-1.94570
C	4.55660	-0.76470	1.25400
H	4.45250	-0.45130	2.28550
C	4.77790	-1.63420	-1.39310
H	4.87030	-1.94820	-2.42530
O	0.71950	-0.39910	0.59610
H	-0.11460	-1.91630	1.02920
N	-1.74110	-1.68890	-1.34320
H	-1.10030	-0.82820	-1.62750

TS9-*E*

M06-2X/6-31G** Derived free energy = -3216.015855

M06-2X/6-31G** Derived free energy in solution = -3216.051432

ONIOM (B3LYP/6-31G**:UFF) Geometry

C	1.45290	3.47670	0.57150
C	3.98450	2.71160	1.52230
C	3.31580	1.89060	0.60340
C	2.00540	2.22760	0.20080
H	4.98030	2.43880	1.85440
C	-2.18230	4.69290	-1.40160
C	-2.14050	3.51880	-0.64190
C	-0.93860	3.13500	-0.02170
C	0.21220	3.93890	-0.12050
H	-3.10850	4.98020	-1.88740
C	3.37760	3.86710	2.02830
C	2.10150	4.26230	1.55590
C	1.51040	5.42830	2.08760
C	2.18270	6.19130	3.04480
C	3.44650	5.80470	3.49040
C	4.04340	4.64800	2.98690
H	0.52540	5.75140	1.77720
H	1.72070	7.08500	3.44400
H	3.96360	6.40090	4.23110
H	5.02420	4.36240	3.34840
C	0.18120	5.10290	-0.93310
C	-1.03640	5.48320	-1.55660
C	-1.08780	6.64070	-2.35010
C	0.05870	7.41080	-2.54720
C	1.26690	7.02840	-1.96610
C	1.33470	5.88170	-1.17180
H	-2.01290	6.94460	-2.82580
H	0.01320	8.29990	-3.16280
H	2.15710	7.62030	-2.13580
H	2.29660	5.60900	-0.76060
P	-0.07700	0.75780	-0.08990
O	1.32920	1.41310	-0.67570
O	-0.88060	1.96600	0.68960
O	-0.82230	0.33600	-1.34610
C	-0.44340	-3.81150	1.58700
N	-1.07360	-2.59800	1.78360
C	3.83070	-3.80170	2.29790
C	3.05050	-2.70860	2.66700
C	1.68020	-2.69140	2.40390
C	1.05840	-3.78010	1.77070

C	1.85850	-4.87910	1.40880
C	3.22720	-4.88820	1.66150
H	4.89790	-3.80800	2.49800
H	3.50490	-1.85340	3.15770
H	1.10420	-1.82000	2.68530
H	1.41670	-5.74390	0.92490
H	3.82180	-5.74630	1.36300
C	-3.17230	-5.02610	-0.67650
C	-4.43660	-4.55700	-1.03460
C	-4.62680	-3.24090	-1.48680
C	-3.55400	-2.35860	-1.60490
H	-3.04390	-6.04790	-0.33280
H	-5.29210	-5.22130	-0.96280
H	-5.62380	-2.90850	-1.75810
H	-3.67800	-1.34370	-1.96540
C	-0.10200	-3.02260	-0.95370
C	-0.65590	-4.24770	-0.46120
C	-2.08150	-4.15630	-0.77850
C	-2.29700	-2.84500	-1.25480
H	0.92730	-2.69340	-0.90750
H	-0.10220	-5.17630	-0.47120
C	-2.37220	-2.36240	2.26290
O	-3.10830	-3.23080	2.69730
C	-2.77390	-0.90590	2.18340
H	-1.94790	-0.22740	2.40270
H	-3.09310	-0.69410	1.15760
H	-3.61360	-0.73870	2.85740
C	-5.48120	0.82690	-0.41630
C	-4.93510	1.38380	0.75380
C	-3.33430	2.64960	-0.56300
H	-5.32400	1.11300	1.72730
C	5.41290	-1.46460	-1.11420
C	4.14020	-1.63700	-0.54500
C	4.03360	0.72690	0.02020
H	3.66090	-2.60800	-0.54620
C	-1.10280	-5.05350	2.15750
H	-0.52350	-5.93960	1.89510
H	-1.11110	-4.96310	3.24910
H	-2.13500	-5.16020	1.83800
C	7.49100	-4.80380	-2.84710

C	6.77610	-4.96650	-1.65850
C	6.10050	-3.88160	-1.09200
C	6.13380	-2.61760	-1.71170
C	6.85870	-2.46640	-2.90910
C	7.53220	-3.55520	-3.47150
H	8.01310	-5.64510	-3.28420
H	6.74770	-5.93360	-1.17320
H	5.56780	-4.02710	-0.16170
H	6.88930	-1.51230	-3.42000
H	8.08350	-3.43130	-4.39470
C	-8.53150	-2.19460	-0.22350
C	-8.59210	-1.20350	-1.20560
C	-7.60760	-0.21240	-1.26480
C	-6.54910	-0.20240	-0.33670
C	-6.50210	-1.20540	0.65110
C	-7.48780	-2.19510	0.70400
H	-9.29270	-2.96280	-0.18190
H	-9.40300	-1.20290	-1.92240
H	-7.68070	0.55200	-2.02800
H	-5.68940	-1.24270	1.36420
H	-7.43730	-2.96770	1.46020
C	-3.86480	2.27930	0.68130
H	-3.44090	2.67400	1.59660
C	-3.90820	2.13610	-1.73480
H	-3.50500	2.40310	-2.70410
C	-4.96920	1.22990	-1.66280
H	-5.35760	0.80940	-2.58170
C	5.30870	0.90020	-0.54330
H	5.77310	1.87870	-0.55710
C	3.46380	-0.55430	0.02050
H	2.50410	-0.72590	0.47300
C	5.99100	-0.18300	-1.10550
H	6.97750	-0.01990	-1.52070
O	0.17020	-0.27240	0.98420
H	-0.57730	-1.74360	1.45570
N	-1.05810	-2.19320	-1.33320
H	-0.93540	-1.10470	-1.45240

TS9-*Z*

M06-2X/6-31G** Derived free energy = -3216.008825

M06-2X/6-31G** Derived free energy in solution = -3216.044536

ONIOM (B3LYP/6-31G**:UFF) Geometry

C	-3.59230	-2.29820	-0.36200
C	-2.33910	-4.58760	-1.42090
C	-1.62610	-3.74490	-0.55950
C	-2.24450	-2.58710	-0.05200
H	-1.85800	-5.47580	-1.81600
C	-5.25170	1.33660	1.24900
C	-4.11500	1.37100	0.43210
C	-3.57410	0.16740	-0.05270
C	-4.19820	-1.06650	0.22520
H	-5.66360	2.26540	1.62850
C	-3.65600	-4.28670	-1.78940
C	-4.29110	-3.12550	-1.27820
C	-5.59470	-2.81530	-1.72300
C	-6.26130	-3.65930	-2.61390
C	-5.63980	-4.81450	-3.08650
C	-4.34150	-5.12660	-2.68200
H	-6.10500	-1.91980	-1.39560
H	-7.26310	-3.41400	-2.94210
H	-6.16150	-5.46430	-3.77710
H	-3.86970	-6.02010	-3.07360
C	-5.31610	-1.10230	1.09640
C	-5.84850	0.11620	1.59290
C	-6.96570	0.09820	2.44340
C	-7.54420	-1.11440	2.82060
C	-7.00980	-2.31790	2.36150
C	-5.90150	-2.31750	1.51170

H	-7.38630	1.02170	2.82350
H	-8.40300	-1.12150	3.47930
H	-7.45380	-3.25620	2.66840
H	-5.50570	-3.27220	1.19250
P	-1.10460	-0.30610	0.08540
O	-1.54070	-1.74740	0.77630
O	-2.41290	0.18640	-0.78410
O	-0.87580	0.61790	1.26980
C	3.41190	0.16580	-0.91300
N	2.32920	1.01350	-1.05370
C	7.41200	1.21520	-2.18190
C	6.32680	2.00580	-2.55490
C	5.04100	1.70650	-2.10590
C	4.80960	0.60430	-1.26800
C	5.91160	-0.20240	-0.92760
C	7.19630	0.10280	-1.36720
H	8.41290	1.45670	-2.52670
H	6.47450	2.86630	-3.20020
H	4.21890	2.33540	-2.41270
H	5.77750	-1.08600	-0.31360
H	8.02640	-0.53430	-1.07710
C	4.91820	1.83200	2.10540
C	4.71870	3.05170	2.74880
C	3.43570	3.47330	3.13580
C	2.31450	2.67700	2.90900
H	5.91120	1.52510	1.79400
H	5.57010	3.69480	2.94930
H	3.31560	4.43450	3.62580
H	1.32050	2.98980	3.21130
C	2.23670	-0.57750	1.40810
C	3.60500	-0.25530	1.17560
C	3.80660	1.02330	1.85810
C	2.53020	1.45350	2.28100
H	1.67650	-1.43440	1.06610
H	4.36720	-1.01410	1.09240
C	2.20820	2.39170	-0.77930
O	3.14790	3.11650	-0.51210
C	0.77760	2.88450	-0.86210
H	0.32360	2.62960	-1.82470
H	0.16240	2.41900	-0.08420

H	0.78580	3.96500	-0.72740
C	-2.15490	5.10000	-0.44900
C	-2.42740	4.73000	0.88100
C	-3.46230	2.66490	0.12720
H	-2.11680	5.36000	1.70470
C	2.52070	-4.47020	0.33190
C	2.06250	-4.46400	-0.99810
C	-0.20960	-4.04500	-0.24620
H	2.75550	-4.57180	-1.82260
C	3.09000	-1.22750	-1.44310
H	2.06190	-1.51280	-1.21700
H	3.78840	-1.97290	-1.06090
H	3.20290	-1.20410	-2.53320
C	6.72770	-4.82010	1.14980
C	6.15100	-5.57440	0.12600
C	4.78110	-5.47280	-0.13490
C	3.97040	-4.61210	0.63010
C	4.56380	-3.86450	1.66580
C	5.93470	-3.96660	1.91910
H	7.78900	-4.89780	1.34770
H	6.76600	-6.23950	-0.46650
H	4.35530	-6.07690	-0.92590
H	3.97430	-3.18270	2.26490
H	6.38300	-3.37950	2.71030
C	-0.14120	8.79980	-1.31800
C	-1.08410	8.74220	-0.28970
C	-1.74160	7.54120	-0.00640
C	-1.46080	6.37980	-0.75210
C	-0.51070	6.45340	-1.78970
C	0.14470	7.65670	-2.06720
H	0.36700	9.73040	-1.53500
H	-1.30880	9.63020	0.28690
H	-2.48190	7.52660	0.78340
H	-0.26020	5.57840	-2.37550
H	0.87740	7.70190	-2.86260
C	-3.07990	3.52710	1.16520
H	-3.26440	3.25650	2.19770
C	-3.19250	3.02940	-1.20070
H	-3.48930	2.38280	-2.01730
C	-2.54850	4.23570	-1.48670

H	-2.37230	4.49810	-2.52200	H	1.90140	-4.32620	2.40610
C	0.23620	-4.08720	1.08370	O	0.01150	-0.51010	-0.91160
H	-0.46140	-3.94890	1.90050	H	1.41670	0.51430	-1.10190
C	0.71130	-4.25440	-1.28330	N	1.60510	0.44700	1.96330
H	0.39040	-4.21580	-2.31710	H	0.53730	0.54610	1.88060
C	1.58730	-4.29980	1.37050				

TS10-*Z*

M06-2X/6-31G** Derived free energy = -3216.008906

M06-2X/6-31G** Derived free energy in solution = -3216.045568

ONIOM (B3LYP/6-31G**:UFF) Geometry

C	-4.36210	0.76630	0.34670	H	-6.39430	-4.11150	-2.42090
C	-4.12960	3.38590	1.36100	H	-8.33580	-2.72340	-3.02170
C	-3.18790	2.91250	0.44140	H	-8.43390	-0.34820	-2.34040
C	-3.30510	1.60760	-0.06790	H	-6.61740	0.65250	-1.04570
H	-4.02760	4.39060	1.75690	P	-1.26560	0.12210	-0.29500
C	-4.25000	-3.31070	-1.04450	O	-2.37390	1.13910	-0.96340
C	-3.17010	-2.76790	-0.33670	O	-2.14050	-0.86260	0.71190
C	-3.22520	-1.42700	0.08600	O	-0.70880	-0.68510	-1.45520
C	-4.37090	-0.63940	-0.15790	C	3.09530	0.66460	1.20740
H	-4.20510	-4.34230	-1.37610	N	2.42090	1.15070	0.10310
C	-5.17400	2.56330	1.80120	C	7.20400	1.76150	1.99230
C	-5.28400	1.23190	1.32040	C	6.43740	2.48690	1.08160
C	-6.29000	0.40010	1.85890	C	5.13550	2.09320	0.78120
C	-7.19070	0.89440	2.80500	C	4.56350	0.96360	1.38820
C	-7.09600	2.21440	3.24350	C	5.33970	0.25870	2.32310
C	-6.08910	3.04500	2.75120	C	6.64670	0.64410	2.61410
H	-6.38620	-0.63520	1.56270	H	8.22150	2.06530	2.21930
H	-7.96280	0.24880	3.20340	H	6.85050	3.36550	0.59550
H	-7.79600	2.59090	3.97820	H	4.56350	2.67200	0.07170
H	-6.01830	4.06090	3.12180	H	4.93140	-0.60380	2.83710
C	-5.43460	-1.17480	-0.92640	H	7.22540	0.07210	3.33320
C	-5.37200	-2.52810	-1.35010	C	5.05440	-2.11280	-0.46610
C	-6.42660	-3.07800	-2.09670	C	5.35430	-2.44290	-1.78680
C	-7.52810	-2.29430	-2.44280	C	4.35280	-2.51380	-2.76840
C	-7.58390	-0.95540	-2.05700	C	3.01540	-2.27900	-2.45340
C	-6.54540	-0.39380	-1.31080	H	5.84010	-2.04240	0.27870

H	6.38420	-2.64290	-2.06570	H	5.20000	7.15580	-1.74720
H	4.62360	-2.76360	-3.78940	H	5.03140	5.41170	0.00500
H	2.23150	-2.34760	-3.20020	H	2.84630	4.43510	0.58000
C	1.64540	-1.55750	0.77950	H	0.96000	6.98900	-2.35300
C	3.03190	-1.46160	1.09810	H	3.16190	7.93400	-2.92250
C	3.72200	-1.86530	-0.13030	C	4.37320	-6.71480	0.84750
C	2.72800	-1.97500	-1.12660	C	4.09300	-6.01650	-0.32830
H	0.79170	-1.34750	1.40700	C	2.82860	-5.45520	-0.52590
H	3.39590	-1.68350	2.09120	C	1.82920	-5.57560	0.45940
C	2.85470	1.24150	-1.23600	C	2.12370	-6.28840	1.63790
O	3.99490	1.01430	-1.59280	C	3.38830	-6.85400	1.82760
C	1.75280	1.67050	-2.18060	H	5.35270	-7.14980	0.99840
H	1.28190	2.59170	-1.83070	H	4.85730	-5.90600	-1.08690
H	2.18580	1.82860	-3.16760	H	2.64230	-4.90970	-1.44030
H	0.96690	0.90830	-2.23230	H	1.37380	-6.42070	2.40730
C	0.49970	-4.93500	0.27080	H	3.60480	-7.40090	2.73620
C	-0.15970	-4.32720	1.35480	C	-1.37030	-3.65650	1.16520
C	-1.94780	-3.57670	-0.11140	H	-1.84580	-3.17880	2.01290
H	0.28240	-4.32750	2.34280	C	-1.31350	-4.21440	-1.18850
C	0.41360	5.04260	-0.50430	H	-1.73750	-4.16100	-2.18370
C	0.08040	4.75460	0.83080	C	-0.10200	-4.88570	-1.00030
C	-2.00240	3.73220	0.11670	H	0.36670	-5.36010	-1.85280
H	0.75390	5.01700	1.63700	C	-1.69120	4.04640	-1.21390
C	2.29870	0.88990	2.48970	H	-2.35960	3.75610	-2.01530
H	1.23230	0.71640	2.33820	C	-1.12000	4.11160	1.13830
H	2.43310	1.93790	2.78240	H	-1.33960	3.86730	2.17060
H	2.67620	0.27240	3.30640	C	-0.49830	4.70630	-1.52150
C	4.23560	6.73460	-1.49430	H	-0.26300	4.90160	-2.56010
C	4.14010	5.75340	-0.50510	O	-0.30150	0.84670	0.61540
C	2.89560	5.20810	-0.17660	H	1.38880	1.18610	0.22570
C	1.73030	5.64010	-0.83840	N	1.48090	-1.75760	-0.52010
C	1.83940	6.62790	-1.83480	H	0.55870	-1.51270	-1.00490
C	3.08660	7.17180	-2.15770				

TS12-*E*

M06-2X/6-31G** Derived free energy = -3522.076623

M06-2X/6-31G** Derived free energy in solution = -3522.093832

ONIOM (B3LYP/6-31G**:UFF) Geometry

C	-3.27860	0.94910	0.63800
C	-2.85010	3.51350	1.71580
C	-2.02360	3.04260	0.69080
C	-2.22050	1.75260	0.16640
H	-2.68590	4.50830	2.11570
C	-3.82760	-2.96740	-1.05360
C	-2.60640	-2.60530	-0.47630
C	-2.41940	-1.29970	0.01360
C	-3.48790	-0.37810	-0.00610
H	-3.95750	-3.97510	-1.43320
C	-3.85250	2.69800	2.25330
C	-4.06400	1.39540	1.73230
C	-5.03770	0.57310	2.33940
C	-5.80620	1.04920	3.40400
C	-5.61140	2.34220	3.88850
C	-4.63670	3.16350	3.32080
H	-5.20600	-0.44230	2.00690
H	-6.55340	0.41090	3.85760
H	-6.20980	2.70520	4.71420
H	-4.48910	4.15910	3.72240
C	-4.86400	-2.03450	-1.17140
C	-4.69840	-0.72130	-0.66170
C	-5.74150	0.21250	-0.84310
C	-6.92830	-0.16240	-1.47680
C	-7.09230	-1.46440	-1.94850
C	-6.06520	-2.39730	-1.80140
H	-5.64610	1.23670	-0.50840
H	-7.72290	0.56120	-1.60550
H	-8.01460	-1.74890	-2.43850
H	-6.20640	-3.40010	-2.18710
P	-0.34290	0.12860	-0.36480
O	-1.41740	1.29150	-0.84910
O	-1.21560	-0.93740	0.57240
O	0.11430	-0.57750	-1.62170
O	0.69960	0.73770	0.54080
H	2.36690	1.27170	-0.15090
C	4.44800	1.09940	-0.15240
N	3.23070	1.46630	-0.68640
C	4.64840	0.49810	4.12310
C	3.41430	0.49980	3.47680

C	3.32890	0.67980	2.09710
C	4.49170	0.86840	1.32590
C	5.73420	0.85270	1.99420
C	5.81140	0.67360	3.37130
H	4.70690	0.36220	5.19900
H	2.49860	0.36290	4.04320
H	2.34130	0.67530	1.64960
H	6.65700	0.99040	1.44530
H	6.78280	0.67620	3.85650
C	2.97470	1.85500	-2.02000
C	1.55920	2.30760	-2.27460
H	1.59020	3.15420	-2.96270
H	1.03040	2.56440	-1.36190
H	1.00020	1.49370	-2.74350
O	3.81360	1.82350	-2.90610
C	5.67470	1.76050	-0.76370
H	5.58860	1.82560	-1.84250
H	6.59020	1.23150	-0.50620
H	5.74110	2.77570	-0.35220
C	3.67130	-1.52600	-0.83330
H	1.75430	-1.27190	-1.66010
C	2.50590	-3.26440	2.90850
C	3.85040	-3.23920	2.53020
C	4.21910	-2.68850	1.30920
C	3.24830	-2.18780	0.41510
C	1.89370	-2.29650	0.77860
C	1.53490	-2.78540	2.03080
H	2.21710	-3.66410	3.87610
H	4.61350	-3.63650	3.19230
H	5.26760	-2.67460	1.03690
H	1.11140	-1.98450	0.10250
H	0.48970	-2.77480	2.31610
C	4.91580	-0.89750	-0.92160
N	2.77030	-1.37620	-1.85930
C	3.12620	-1.32090	-3.23070
C	2.00760	-0.91420	-4.16030
H	2.24960	0.09140	-4.52100
H	2.01250	-1.58160	-5.02470
H	1.02940	-0.89390	-3.68050
O	4.24360	-1.58940	-3.62660

H	5.67990	-1.13610	-0.19560
H	5.25680	-0.58150	-1.89570
C	1.19970	5.49580	-0.77830
C	-0.92150	3.90390	0.19390
H	2.01810	6.09930	-1.15700
C	0.56960	-5.50410	-0.21640
C	-1.52120	-3.61400	-0.39260
H	1.38110	-6.22090	-0.14460
C	-1.30330	-4.32580	0.81560
C	-0.25040	-5.27360	0.89190
C	-0.01570	-5.96940	2.08920
C	-0.81570	-5.73690	3.20780
C	-1.85830	-4.81410	3.14230
C	-2.10600	-4.11420	1.95960
H	0.79120	-6.68900	2.16170
H	-0.62690	-6.27410	4.12820
H	-2.47750	-4.63880	4.01260
H	-2.92560	-3.40780	1.95330
C	-0.70800	-3.88660	-1.52430
C	0.34890	-4.82650	-1.41880
C	1.17400	-5.08490	-2.52560
C	0.94430	-4.44100	-3.74090
C	-0.10660	-3.53430	-3.86330
C	-0.92020	-3.24690	-2.76600
H	1.99150	-5.79240	-2.45320
H	1.58070	-4.64790	-4.59150
H	-0.28360	-3.03990	-4.80970
H	-1.71130	-2.52140	-2.90120
C	0.02040	5.38400	-1.52150
C	-1.05130	4.58660	-1.04420
C	-2.22630	4.49720	-1.82450
C	-2.32420	5.17040	-3.04400
C	-1.26170	5.94510	-3.50620
C	-0.09470	6.05430	-2.75030
H	-3.07690	3.91160	-1.50180
H	-3.22910	5.09150	-3.63260
H	-1.34340	6.46420	-4.45250
H	0.71960	6.66170	-3.12750
C	1.33940	4.82810	0.44200
C	0.27700	4.03500	0.94670
C	0.45700	3.37680	2.18470
C	1.65250	3.50650	2.89370
C	2.69520	4.27410	2.37930
C	2.54190	4.93380	1.16030
H	-0.31500	2.75070	2.61130
H	1.77460	2.99920	3.84170
H	3.62480	4.35830	2.92710
H	3.36440	5.52680	0.77810

TS11-*E*

M06-2X/6-31G** Derived free energy = -3522.087155

M06-2X/6-31G** Derived free energy in solution = -3522.10237

ONIOM (B3LYP/6-31G**:UFF) Geometry

C	1.85580	2.74960	-0.61940
C	-0.15820	4.31170	-1.81260
C	-0.49530	3.37710	-0.82790
C	0.50450	2.56670	-0.26280
H	-0.93670	4.92890	-2.24770
C	4.83040	0.40030	1.42470
C	3.78600	-0.21860	0.73200
C	2.79740	0.55940	0.10620
C	2.88590	1.96830	0.12430
H	5.58310	-0.21220	1.90910
C	1.16420	4.43220	-2.25740
C	2.18580	3.64020	-1.67230
C	3.50060	3.75020	-2.17280
C	3.79590	4.64880	-3.20030
C	2.78990	5.44240	-3.75100
C	1.47860	5.33450	-3.28590
H	4.30430	3.13910	-1.78430

H	4.80890	4.72740	-3.57330	H	-5.95420	-2.99340	-0.60110
H	3.02530	6.13700	-4.54700	H	-5.93530	-1.64720	-1.75360
H	0.70880	5.95070	-3.73530	C	-2.07040	-3.56030	0.39720
C	4.88900	1.79490	1.52470	H	-0.75030	-2.32560	1.41920
C	3.90290	2.59500	0.89150	C	0.70910	-4.66420	-2.69070
C	3.95270	3.99510	1.06740	C	1.02840	-3.63610	-1.80450
C	4.97810	4.58210	1.81190	C	0.16580	-3.30360	-0.76470
C	5.96070	3.78870	2.40290	C	-1.08120	-3.94690	-0.63510
C	5.91610	2.40090	2.26580	C	-1.38470	-4.99170	-1.53450
H	3.19910	4.64490	0.64380	C	-0.49470	-5.35670	-2.53730
H	5.00840	5.65720	1.93420	H	1.39410	-4.92780	-3.49120
H	6.75290	4.24970	2.97860	H	1.94010	-3.06530	-1.93040
H	6.67950	1.80050	2.74660	H	0.46600	-2.52510	-0.07670
P	0.28840	0.07550	0.10420	H	-2.31370	-5.53990	-1.44010
O	0.17300	1.62470	0.67880	H	-0.74760	-6.17240	-3.20700
O	1.78830	-0.05640	-0.59360	C	-3.44210	-3.75540	0.17240
O	0.20260	-0.80970	1.32660	N	-1.61340	-2.89650	1.50110
O	-0.68970	-0.14060	-1.02570	C	-2.27170	-2.77520	2.74750
H	-2.33030	-0.93410	-1.33950	C	-1.46800	-2.01970	3.78180
C	-4.07450	-1.92690	-0.76340	H	-2.16520	-1.50690	4.44550
N	-3.09030	-1.55130	-1.66530	H	-0.90870	-2.74660	4.38170
C	-4.54840	0.52650	2.76040	H	-0.76730	-1.31470	3.33130
C	-3.33500	0.51040	2.07730	O	-3.34770	-3.28880	2.98970
C	-3.18510	-0.26450	0.92860	H	-3.72440	-4.40890	-0.64140
C	-4.23840	-1.05780	0.44290	H	-4.10670	-3.71340	1.02130
C	-5.46190	-1.02460	1.14350	C	-4.60630	3.08650	0.44130
C	-5.61460	-0.23850	2.27940	C	-1.90980	3.26150	-0.38530
H	-4.67060	1.13690	3.65030	H	-5.64000	3.01150	0.76300
H	-2.49390	1.10730	2.41540	C	3.55750	-4.51790	0.60970
H	-2.24230	-0.22720	0.40500	C	3.71870	-1.70020	0.69450
H	-6.30940	-1.60750	0.80680	H	3.48710	-5.60000	0.57240
H	-6.57040	-0.22610	2.79400	C	4.24080	-2.41050	-0.41710
C	-2.81640	-2.14980	-2.90900	C	4.15990	-3.82660	-0.44580
C	-1.66360	-1.52760	-3.66800	C	4.66310	-4.53750	-1.54770
H	-1.16550	-0.72160	-3.13030	C	5.24440	-3.85840	-2.61840
H	-2.04580	-1.17660	-4.63120	C	5.33180	-2.46740	-2.59990
H	-0.93960	-2.31940	-3.87860	C	4.83780	-1.74520	-1.51150
O	-3.44680	-3.09220	-3.36290	H	4.60050	-5.61870	-1.58380
C	-5.35460	-2.49020	-1.35770	H	5.62750	-4.41240	-3.46560
H	-5.14070	-3.17090	-2.17390	H	5.78410	-1.94520	-3.43310

H	4.92610	-0.66700	-1.53810	C	-5.17340	1.87580	-1.57800
C	3.13100	-2.41410	1.77170	H	-1.50990	2.08180	-2.83170
C	3.04290	-3.82810	1.71130	H	-3.21290	1.00210	-4.20510
C	2.43960	-4.54070	2.76040	H	-5.54620	0.82300	-3.41510
C	1.93770	-3.86630	3.87320	H	-6.20310	1.78980	-1.25140
C	2.03460	-2.47830	3.95150	C	-3.66970	3.71780	1.26450
C	2.62150	-1.75410	2.91260	C	-2.31300	3.81190	0.86100
H	2.35960	-5.62070	2.72150	C	-1.39900	4.46570	1.71890
H	1.47460	-4.42140	4.67860	C	-1.81910	4.98670	2.94440
H	1.64580	-1.95920	4.81800	C	-3.15200	4.87560	3.33520
H	2.66340	-0.67720	3.00960	C	-4.07550	4.24770	2.50020
C	-4.22050	2.53100	-0.78120	H	-0.35650	4.58200	1.45460
C	-2.87140	2.61950	-1.21170	H	-1.10700	5.48020	3.59330
C	-2.52470	2.05170	-2.45830	H	-3.47150	5.28030	4.28690
C	-3.48970	1.42290	-3.24790	H	-5.10760	4.17280	2.82200
C	-4.80680	1.32530	-2.80500				

TS11-Z

M06-2X/6-31G** Derived free energy = -3522.084806

M06-2X/6-31G** Derived free energy in solution = -3522.101877

ONIOM (B3LYP/6-31G**:UFF) Geometry

C	2.59830	2.48060	0.06090	H	5.19160	2.81520	-0.76330
C	0.97300	4.67740	-0.61370	H	6.11460	4.81030	-1.83100
C	0.39710	3.53360	-0.05060	H	4.67200	6.75730	-2.32490
C	1.20170	2.42390	0.25560	H	2.27630	6.70590	-1.76160
H	0.34230	5.52680	-0.85240	C	5.22610	0.30600	1.81690
C	4.96130	-0.92920	1.21410	C	4.44030	1.44080	1.48770
C	3.89910	-1.06840	0.31530	C	4.68740	2.65480	2.16460
C	3.11340	0.04760	-0.01990	C	5.71430	2.74670	3.10670
C	3.41730	1.31830	0.51180	C	6.50160	1.63370	3.39940
H	5.55830	-1.79810	1.46910	C	6.25790	0.41640	2.76260
C	2.34190	4.71640	-0.90400	H	4.08650	3.53520	1.98200
C	3.16770	3.60730	-0.58620	H	5.89740	3.68470	3.61490
C	4.53190	3.65280	-0.94600	H	7.29560	1.71130	4.13090
C	5.06580	4.78530	-1.56470	H	6.86920	-0.44150	3.01670
C	4.25210	5.88250	-1.84530	P	0.58500	0.00630	-0.21100
C	2.89520	5.84940	-1.52150	O	0.63350	1.30510	0.81170

O	2.07870	-0.08840	-0.91250
O	0.35810	-1.18410	0.69520
O	-0.37350	0.25320	-1.35400
H	-2.03370	-0.08590	-1.36320
C	-3.76080	-0.62220	-0.36590
N	-3.03660	-0.35870	-1.50250
C	-7.95610	0.29650	0.04520
C	-7.29540	-0.45840	1.01610
C	-5.95380	-0.78670	0.85170
C	-5.24120	-0.38630	-0.29460
C	-5.92140	0.36780	-1.26310
C	-7.26050	0.71010	-1.08980
H	-9.00190	0.55890	0.17360
H	-7.82280	-0.78850	1.90580
H	-5.46030	-1.37190	1.62020
H	-5.40880	0.69420	-2.15530
H	-7.75900	1.30210	-1.85120
C	-3.38000	-0.70750	-2.82290
C	-2.33460	-0.35260	-3.85540
H	-1.49280	0.20300	-3.44170
H	-2.81540	0.21550	-4.65620
H	-1.97640	-1.29140	-4.28900
O	-4.42140	-1.27480	-3.09900
C	-3.01170	-0.21230	0.88610
H	-1.96310	-0.50660	0.86260
H	-3.47510	-0.60870	1.78740
H	-3.05020	0.87890	0.95000
C	-2.57440	-3.06980	0.24000
H	-0.58510	-2.53910	0.06600
C	-2.42460	-4.02640	4.42990
C	-3.46920	-4.48850	3.62790
C	-3.50820	-4.15990	2.27570
C	-2.49400	-3.37390	1.69290
C	-1.43290	-2.93190	2.50700
C	-1.41470	-3.24740	3.86410
H	-2.39560	-4.27620	5.48640
H	-4.25070	-5.11190	4.05160
H	-4.30860	-4.55210	1.65770
H	-0.64270	-2.31080	2.09690
H	-0.60290	-2.87700	4.48240

C	-3.80600	-2.82640	-0.36380
N	-1.39230	-2.94610	-0.44390
C	-1.19320	-3.15080	-1.82240
C	0.20920	-2.84340	-2.29110
H	0.22250	-1.82720	-2.69650
H	0.93480	-2.86460	-1.48120
H	0.48180	-3.54880	-3.07730
O	-2.06670	-3.55530	-2.57010
H	-4.69520	-3.03080	0.21540
H	-3.91440	-2.88020	-1.43760
C	-3.85970	3.54620	0.63320
C	-1.06920	3.50030	0.19100
H	-4.93150	3.56290	0.80270
C	2.96770	-4.95070	-1.30980
C	3.58730	-2.40920	-0.24280
H	2.72350	-5.92420	-1.72220
C	3.93350	-2.72430	-1.58350
C	3.61510	-4.00320	-2.10860
C	3.94260	-4.31970	-3.43720
C	4.58700	-3.38420	-4.24630
C	4.91430	-2.12820	-3.73840
C	4.59580	-1.79720	-2.41970
H	3.69900	-5.29140	-3.85050
H	4.83600	-3.63480	-5.26940
H	5.41860	-1.40720	-4.36890
H	4.87440	-0.81360	-2.06560
C	2.92770	-3.38000	0.55910
C	2.61840	-4.65110	0.01030
C	1.94470	-5.60840	0.78670
C	1.58190	-5.32070	2.10210
C	1.88800	-4.07920	2.65500
C	2.54680	-3.11210	1.89390
H	1.69650	-6.57960	0.37500
H	1.06200	-6.06230	2.69480
H	1.60800	-3.86160	3.67720
H	2.74330	-2.15540	2.35860
C	-3.36720	3.40700	-0.66720
C	-1.96890	3.36820	-0.90070
C	-1.51190	3.19060	-2.22650
C	-2.41720	3.07790	-3.28330

C	-3.78830	3.13450	-3.04290
C	-4.26340	3.30120	-1.74270
H	-0.45720	3.12700	-2.45850
H	-2.05300	2.94000	-4.29320
H	-4.48550	3.04270	-3.86550
H	-5.33350	3.33920	-1.57640
C	-2.98490	3.64840	1.71900
C	-1.58160	3.63450	1.50920
C	-0.73130	3.76810	2.63050
C	-1.26140	3.88630	3.91700
C	-2.64140	3.88290	4.11150
C	-3.50130	3.76800	3.01960
H	0.34530	3.78300	2.52710
H	-0.59810	3.98260	4.76700
H	-3.04630	3.97470	5.11110
H	-4.57120	3.77390	3.19160

TS12-Z

M06-2X/6-31G** Derived free energy = -3522.079997

M06-2X/6-31G** Derived free energy in solution = -3522.098022

ONIOM (B3LYP/6-31G**:UFF) Geometry

C	-2.77590	-2.19950	-0.57180
C	-1.35530	-4.35600	-1.69290
C	-0.74790	-3.55050	-0.72530
C	-1.44120	-2.45220	-0.18860
H	-0.80690	-5.19640	-2.10470
C	-4.86990	1.13070	1.17190
C	-3.65390	1.31950	0.50740
C	-2.94950	0.21230	0.00570
C	-3.50090	-1.08180	0.09810
H	-5.40160	1.99190	1.56180
C	-2.64490	-4.06640	-2.15230
C	-3.36380	-2.97080	-1.60810
C	-4.63810	-2.66860	-2.13560
C	-5.19590	-3.45900	-3.14300
C	-4.49340	-4.55180	-3.64940
C	-3.22170	-4.85390	-3.16140
H	-5.20710	-1.81820	-1.78520
H	-6.17610	-3.22020	-3.53520
H	-4.93110	-5.16040	-4.43010
H	-2.68530	-5.69800	-3.57860
C	-5.38350	-0.15780	1.36240
C	-4.69390	-1.28220	0.83910
C	-5.20910	-2.57170	1.09200
C	-6.39560	-2.73390	1.81070
C	-7.08100	-1.62090	2.29680
C	-6.57790	-0.33770	2.07830
H	-4.69720	-3.46050	0.74820
H	-6.78320	-3.72770	1.99440
H	-8.00010	-1.75340	2.85280
H	-7.11790	0.51350	2.47580
P	-0.45100	-0.14590	0.25360
O	-0.84300	-1.67050	0.76960
O	-1.74580	0.39540	-0.62920
O	-0.27190	0.67730	1.50520
O	0.68760	-0.20430	-0.74420
H	2.30140	-0.20290	-0.22680
C	4.05320	0.77120	-0.67870
N	3.29310	-0.07380	0.09000
C	8.30550	0.17500	-1.12680
C	7.71140	1.36040	-1.56170
C	6.34800	1.56930	-1.37460
C	5.54810	0.61030	-0.72930
C	6.15860	-0.58200	-0.30810
C	7.52000	-0.79650	-0.50780
H	9.36830	0.00890	-1.27420
H	8.30780	2.12340	-2.05250

H	5.90900	2.49260	-1.73570
H	5.56960	-1.35390	0.16570
H	7.96460	-1.72980	-0.17590
C	3.54650	-0.45020	1.42320
C	2.48770	-1.34970	2.01960
H	1.87650	-1.83750	1.26420
H	1.80760	-0.75470	2.63340
H	2.97570	-2.08660	2.65980
O	4.52390	-0.06740	2.04100
C	3.42900	1.05070	-2.03510
H	2.34150	1.01310	-1.99450
H	3.74970	2.01310	-2.43490
H	3.77690	0.27910	-2.73000
C	2.59680	2.86750	0.47350
H	0.94740	1.99650	1.42370
C	0.51340	5.00270	-2.61390
C	0.07010	3.73320	-2.24140
C	0.70150	3.03390	-1.21560
C	1.82990	3.57950	-0.58390
C	2.28190	4.85330	-0.97500
C	1.61590	5.56620	-1.96970
H	0.00450	5.54910	-3.40250
H	-0.77510	3.27530	-2.74530
H	0.37080	2.03250	-0.97600
H	3.13840	5.29480	-0.47520
H	1.96310	6.55770	-2.24410
C	3.97350	2.69130	0.33160
N	1.92280	2.34010	1.53850
C	2.43300	2.30260	2.86620
C	1.48230	1.72340	3.88980
H	0.59420	1.27210	3.44760
H	2.03530	0.98270	4.47360
H	1.19870	2.52390	4.58010
O	3.51890	2.75580	3.16160
H	4.46770	3.29520	-0.41770
H	4.56080	2.37840	1.18300
C	3.27200	-4.45450	0.53520
C	0.63690	-3.86040	-0.29030
H	4.28460	-4.67720	0.85540
C	-2.08010	5.30110	0.02750
C	-3.11770	2.69410	0.34580
H	-1.67320	6.29920	-0.09850
C	-3.30330	3.39140	-0.87650
C	-2.79050	4.70610	-1.01920
C	-2.97830	5.40800	-2.22140
C	-3.66140	4.81630	-3.28350
C	-4.16790	3.52410	-3.15580
C	-3.99620	2.81500	-1.96510
H	-2.58710	6.41130	-2.34290
H	-3.79740	5.36090	-4.20890
H	-4.69840	3.06960	-3.98260
H	-4.41120	1.81740	-1.90760
C	-2.42510	3.32220	1.41430
C	-1.89070	4.62370	1.23530
C	-1.17690	5.24040	2.27560
C	-1.00990	4.58970	3.49770
C	-1.55410	3.32210	3.69410
C	-2.24860	2.68650	2.66370
H	-0.75430	6.22980	2.14600
H	-0.46180	5.07170	4.29680
H	-1.42820	2.82340	4.64650
H	-2.63850	1.69560	2.85480
C	3.06050	-3.77650	-0.66890
C	1.74190	-3.47340	-1.09480
C	1.57000	-2.77200	-2.30950
C	2.67420	-2.38770	-3.07240
C	3.96500	-2.69210	-2.64380
C	4.15980	-3.38550	-1.45020
H	0.58740	-2.50340	-2.67380
H	2.52810	-1.84440	-3.99700
H	4.81750	-2.38510	-3.23580
H	5.17160	-3.61030	-1.13380
C	2.19230	-4.83550	1.33790
C	0.86430	-4.54430	0.93310
C	-0.20070	-4.94790	1.77010
C	0.05100	-5.60860	2.97430
C	1.36050	-5.88310	3.36480
C	2.42770	-5.50110	2.55200
H	-1.23240	-4.75810	1.50490
H	-0.77390	-5.90940	3.60730

H	1.54930	-6.39510	4.29960
H	3.43810	-5.72470	2.87350

	Kekulé ONIOM (B3LYP/6-31G**:UFF)		Delocalised ONIOM (B3LYP /6-31G**:UFF)	
**Mechanism** (Catalyst)	$\Delta\Delta G^{\ddagger}$	$\Delta\Delta G_{sol}^{\ddagger}$	$\Delta\Delta G^{\ddagger}$	$\Delta\Delta G_{sol}^{\ddagger}$
**TS1**	0	0	0	0
**TS2**	+3.5	+3.9	+3.2	+3.6
**TS3**	0	0	0	0
**TS4**	+3.5	+3.6	+3.4	+3.6
**TS5-*E***	0	0	0	0
**TS6-*E***	+1.3	+1.7	+1.3	+1.6
**TS5-*Z***	+3.1	+3.2	+3.6	+3.8
**TS6-*Z***	+3.9	+4.1	+2.4	+2.6
**TS8-*E***	0	0	0	0
**TS7-*E***	-0.8	+0.3	-0.6	+0.5
**TS7-*Z***	+4.5	+3.2	+3.9	+2.6
**TS8-*Z***	+5.8	+5.0	+3.3	+2.7
**TS10-*E***	0	0	0	0
**TS9-*E***	+1.8	+0.8	+1.2	+0.2
**TS9-*Z***	+4.8	+3.4	+5.6	+4.5
**TS10-*Z***	+6.1	+4.3	+5.6	+3.9
**TS12-*E***	0	0	+6.6	+5.4
**TS11-*Z***	+0.1	+0.2	+1.5	+0.3
**TS11-*E***	-0.9	+0.4	0	0
**TS12-*Z***	+3.7	+3.1	+4.5	+2.7

Table 2. Comparison of the relative energies derived from both ONIOM methods. All energies in kcal mol^{-1}

References

[1] MacroModel, version 9.9, Schrodinger, LLC, New York, NY, 2009.

[2] Kaminski, G.A.; Friesner, R. A.; Tirado-Rives, J.; Jorgensen, E. *J. Phys. Chem. B* **2001**, *105*, 6474.

[3] Jorgensen, W. L.; Maxwell, D. S.; Tirado-Rives, J. *J. Am. Chem. Soc.* **1996**, *118*, 11225.

[4] Jorgensen, W. L; Tirado-Rives, J. *J. Am. Chem. Soc.* **1988**, *110*, 1657.

[5] Frisch, M. J. et al. Gaussian 09, Revision D.01, Gaussian, Inc., Wallingford, CT, 2013.

[6] Becke, A. D. *Phys. Rev. A* **1988**, *38*, 3098.

[7] Lee, C.; Yang, W.; Parr, R. G. *Phys. Rev. B* **1988**, *37*, 785

[8] Krishnan R.; Binkley J. S.; Seeger R.; Pople J. A. *J. Chem. Phys.* **1980**, *72*, 650.

[9] Gill, P. M. W.; Johnson, B. G.; Pople, J. A.; Frisch, M. J. *Chem. Phys. Lett.* **1992**, *197*, 499.

[10] Rappe, A. K.; Casewit, C. J.; Colwell, K. S.; Goddard, W. A.; Skiff, W. M. *J. Am. Chem. Soc.* **1992**, *114*, 10024.

[11] Simón, L.; Goodman, J. M. *J. Am. Chem. Soc.* **2009**, *131*, 4070.

[12] Simón, L.; Goodman, J. M. *J. Org. Chem.* **2010**, *75,* 589.

[13] Grayson, M. N.; Pellegrinet, S. C.; Goodman, J. M. *J. Am. Chem. Soc.* **2012**, *134,* 2716.

[14] Overvoorde, L. M.; Grayson, M. N.; Luo, Y.; Goodman, J. M. *J. Org. Chem.* **2015**, *80*, 2634.

[15] Reid, J. P.; Goodman, J. M. *J. Am. Chem. Soc.* **2016**, *138*, 7910.

[16] We have re-optimized the lowest energy TS structures using the delocalized bonding arrangement and have concluded that it does not affect the relative energies to an appreciable extent.

[17] Zhao, Y.; Truhlar, D. *Theor. Chem. Acc.* **2008**, *120*, 215.

[18] Jaguar, version 7.9, Schrodinger, LLC, New York, NY, 2012.

[19] Mennucci, B.; Tomasi, J. *J. Chem. Phys.* **1997**, *106*, 5151.

[20] CYL view, 1.0b; Legault, C. Y., Université de Sherbrooke, 2009.

[21] Superposition of the TS geometries showed no obvious structural origin for the difference in the two methods.

[22] Reagents take similar arrangements with both catalysts. The small energy difference between TSs leading to competing products with the 1-naphthyl derived catalyst can be traced back to C-H$\cdots$O interactions with the alkyl group and the phosphate in the *Type II* TS. These interactions are diminished in the corresponding TS involving the 9-phenanthryl as the alkyl group rotates away from the phosphate.
